# Ambiphilic Reactivity and Switchable Methyl Transfer at a T‑Shaped Bi(NNN) Complex Enabled by a Redox-Active Pincer Ligand

**DOI:** 10.1021/jacs.5c18955

**Published:** 2026-01-06

**Authors:** Sotirios Pavlidis, Eric W. Fischer, Amanda Opis-Basilio, Ayan Bera, Ana Guilherme Buzanich, María Álvarez-Sánchez, Severin Wittek, Franziska Emmerling, Kallol Ray, Michael Roemelt, Josh Abbenseth

**Affiliations:** † Institut für Chemie, 9373 Humboldt-Universität zu Berlin, Brook-Taylor-Str. 2, 12489 Berlin, Germany; ‡ Department of Materials Chemistry, Federal Institute for Materials Research and Testing, Richard-Willstätter-Str. 11, 12489 Berlin, Germany; § Department of Chemistry, 5292 University of Manchester, Oxford Road, Manchester M13 9PL, U.K.

## Abstract

We report the transition-metal-like reactivity of a geometrically constrained, ambiphilic bismuth­(III) trisamide. Planarization of the Bi­(III) center unlocks Bi–C bond formation when reacted with mild electrophiles (alkyl iodides and triflates) accompanied by two-electron oxidation of the utilized NNN pincer ligand. The preservation of the bismuth oxidation state is confirmed by single-crystal X-ray diffraction and X-ray absorption spectroscopy and corroborated by theoretical calculations. Sequential reduction of the oxidized ligand framework alters the reactivity of a generated Bi–Me unit, enabling controlled access to methyl cation, radical, and anion equivalents. The full [Bi­(Me)­(NNN)]^+/•/–^ redox series was comprehensively characterized using NMR and EPR spectroscopy as well as spectro-electrochemistry. This work represents the first example of ligand-assisted, redox-neutral C–X bond splitting at bismuth, establishing a new paradigm for synthetic bismuth chemistry.

## Graphical abstract

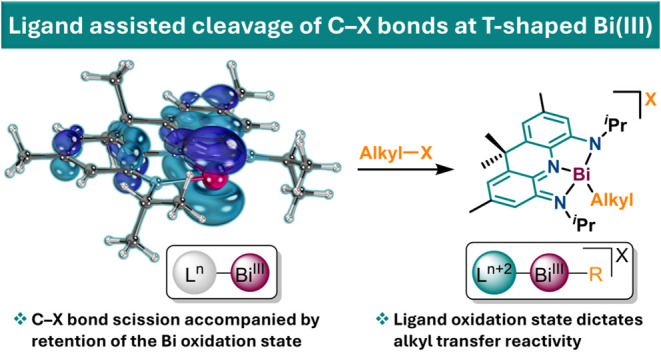

## Introduction

Noble transition metals are indispensable in synthetic chemistry due to their ability to undergo catalytic bond formation reactions via reversible two-electron elementary steps, e.g., oxidative addition or reductive elimination.^1,2^ However, their high cost has motivated chemists to develop more sustainable catalysts based on 3d metals. Metal–ligand cooperativity is a cornerstone in facilitating catalytic transformations, with the use of redox-active ligands being particularly effective.^3–8^ Pushing this sustainability concept further, heavy p-block element compounds have recently garnered attention as potential substitutes for traditional transition metals. When bonded to lighter p-block elements, these species exhibit comparably low bond dissociation free energies (BDFEs).^9^ This, in combination with their inherently lower Lewis acidity and resistance to β-hydride elimination in alkyl compounds when compared to transition metal catalysts, further distinguishes them as promising candidates for catalytic applications.^2^ Until now, the chemistry of p-block elements supported by redox-active ligands remains in its infancy, with the majority of reported systems based on group 13 elements and Si­(IV).^10,11^ More recently, transition-metal-like bond activation by group 15 elements was reported when coordinated to redox-active scaffolds. These include oxygen activation by P, Sb, and Bi or phenyl hydrazine disproportionation by phosphorus.^12–20^ Notably, the latter transformation mirrors reactivity observed with Zr­(IV) complexes using the identical ligand platform, underscoring the untapped potential of repurposing established redox-active ligand platforms for main-group catalysis.^21^ Bismuth represents a particularly intriguing element in this context due to its low price, widespread availability, and low toxicity, in addition to its unique reactivity profiles arising from relativistic effects.^22–24^ Its vast potential in catalytic carbon-element bond-forming reactions was recently demonstrated by several studies establishing bismuth’s ability to cycle between redox states. In contrast, redox-neutral applications using Bi catalysts in which the ligand acts as an electron reservoir to drive coupling reactions remain elusive.^19,23–27^ A highly promising compound class exhibiting transition metal-like, ambiphilic reactivity are geometrically constrained pnictogen species of the type PnR_3_ (Pn = Pnictogen) in VSEPR noncompliant *C*_s_ or *C*_2v_ geometries. In particular, phosphines were recently shown to unlock transition metal mimetic bond activation chemistry.^10,18,19,29–33^ The geometric perturbation away from typical *C*_3v_ symmetric structures results in decreased HOMO/LUMO gaps, with the LUMO featuring pronounced empty Pn­(p) orbital character enabling ambiphilic reactivity. Recent works by Chitnis, Hwang, and us have shown that Bi­(III) centers planarized by trianionic, redox-active NNN pincer ligands can undergo adduct formation with Lewis acids and bases as well as oxygen binding, suggesting great potential for small molecule activation (Figure 1A,B).^18,19,34–42^

**Figure 1:**
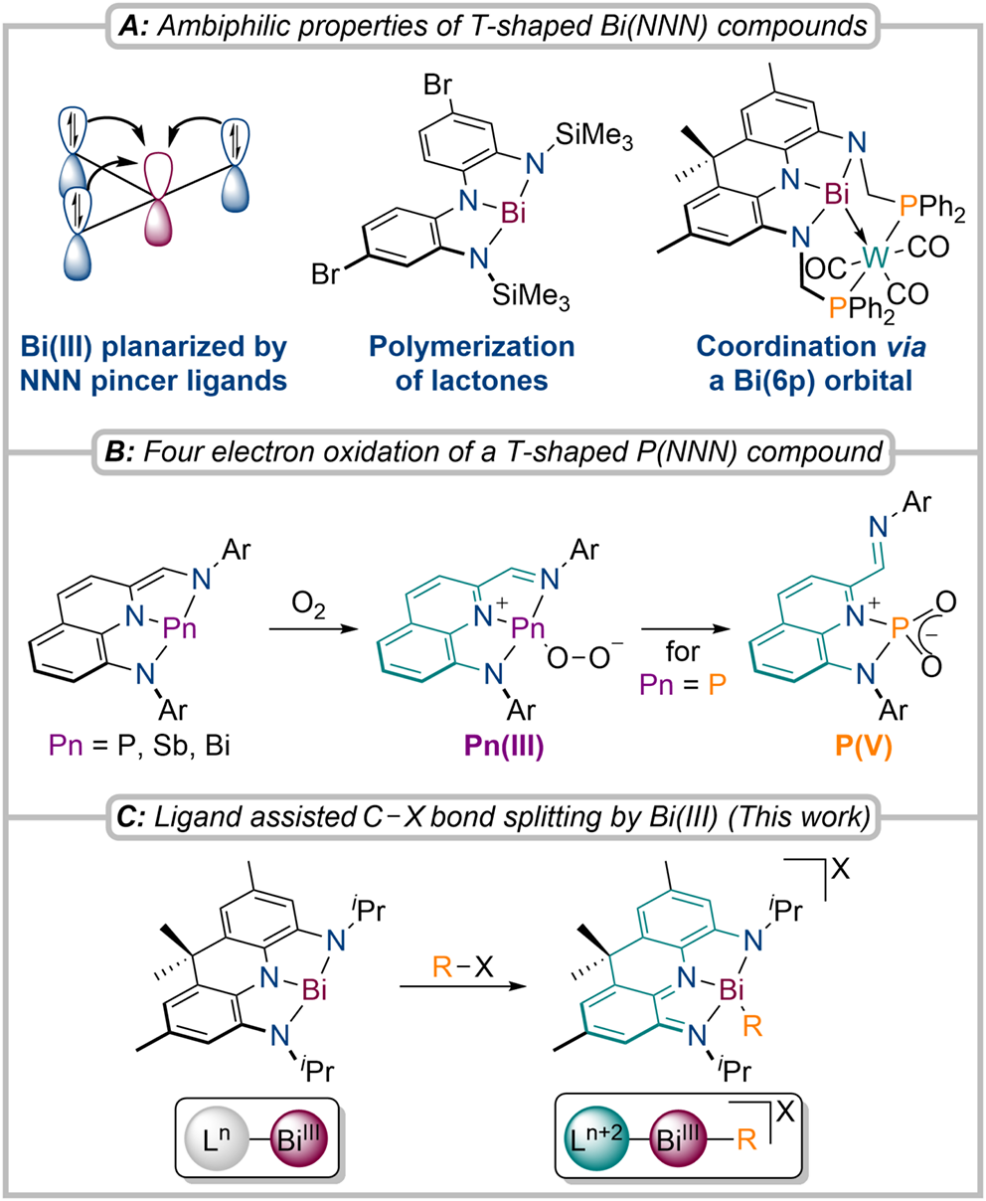
Recently reported examples and properties of T-shaped pnictogens planarized by redox-active NNN pincer ligands (A, B);^18,19,36,37^ This work: Ligand participation in C–X bond scission at Bi­(III) (C).

Yet, the realization of transition metal-like splitting of C–X bonds by Bi­(III) under active participation of redox-active ligands remains elusive but would open up a new design principle for Bi catalysis since highly reactive oxidation states like Bi­(I/II/V) can be efficiently circumvented by outsourcing the redox-shuttling to the supporting ligand scaffold. Herein, we demonstrate that a Bi­(III) center planarized by a rigid redox-active NNN pincer ligand can cleave C–X bonds, a hallmark of transition metal chemistry. The electronic flexibility of the ligand framework allows the decoupling of electron transfer and Bi–element bond formation, resulting in the redox-neutral addition of methyl triflate and alkyl iodides at the bismuth center, while the ligand is oxidized by two electrons. We further show that all available redox states of the ligand are accessible and can be utilized to steer the reactivity of the Bi-bound alkyl group. This provides a new design strategy for redox-driven catalysis using main-group elements and paves the way for new sustainable bismuth-based catalytic cycles.

## Results and Discussion

### Synthesis and Electronic Structure of 1^Bi^

The T-shaped bismuth trisamide [Bi­(NNN)] (**1** ^**Bi**^) forms quantitatively and instantaneously upon reaction of the protioligand **H**_**3**_ **NNN** ^43,44^ with Bi­(NMe_2_)_3_ in benzene at 25 °C (Figure 2). In contrast to the structurally related constrained Bi trisamide Bi­{N­[*o*-N­(SiMe_3_)-C_6_H_4_]_2_} (**1** ^**Bi_TMS**^)^39^ reported by Chitnis and co-workers, no coordination of *in situ* formed dimethylamine was observed, which we ascribe to more pronounced van-der-Waals interactions being present in **1** ^**Bi_TMS**^. The ^1^H and ^13^C­{^1^H} nuclear magnetic resonance (NMR) spectra confirm the presence of a diamagnetic reaction product exhibiting *C*_2v_ symmetry on the NMR time scale. The molecular structure of **1** ^**Bi**^ derived by single-crystal X-ray diffraction (scXRD) reveals a planar, T-shaped Bi center in which the Bi–N bond lengths compare well with structurally related Bi­(NNN) pincer compounds (Figure 2).^36,37,39,41^ We first compared the structural properties of the recently reported phosphorus trisamide [P­(NNN)] (**1** ^**P**^) and **1** ^**Bi**^, as accessing T-shaped P and Bi compounds has been highly challenging upon utilization of an identical ligand system.^45^ This is likely due to phosphorus’ pronounced s/p-orbital mixing, which strongly favors pyramidalization, while heavy pnictogens can be readily planarized by flexible pincer ligands.^33^ In fact, T-shaped phosphines remain particularly rare, with **1** ^**P**^ being one of the only four examples reported in literature that all leverage highly rigid pincer scaffolds.^18,45–47^ For **1** ^**Bi**^, significantly longer Pn–N bonds are observed when compared to **1** ^**P**^. In addition, the ligand in **1** ^**Bi**^ displays more localized C–C single and double bonds, suggesting increased delocalization of electron density from the NNN ligand toward the Bi center (Figure 2).^43,44,48^ Characteristic bond lengths of fully planar **1** ^**Bi**^ and Chitnis’ **1** ^**Bi_TMS**^, which features a canted ligand backbone, display pronounced structural similarities (see SI for a detailed comparison). The Bi–N bond to the central N donor and the N1/3–C2/12 bonds are slightly shorter in **1** ^**Bi**^ (Δ*d* ≈ 0.01 Å). The Bi–N bonds to the flanking nitrogen doors are inequivalent in **1** ^**Bi_TMS**^ (2.303(4) and 2.282(3) Å), in contrast to fully symmetric **1** ^**Bi**^. Theoretical evaluation of different conformers of **1** ^**P**^ and **1** ^**Bi**^ shows that a bent conformer of **1** ^**Bi**^ is not a local minimum in contrast to **1** ^**P**^, which is only Δ*E*_CC_ = 0.8 kcal/mol lower in energy than the T-shaped geometry previously observed by scXRD (DLPNO–CCSD­(T)/Def2-TZVPP). Consequently, a Boltzmann-distributed mixture of both conformers of **1** ^**P**^ is present in solution. This extremely flat potential energy surface and a transition state of ∼1 kcal/mol explain why **1** ^**P**^ appears strictly *C*_2v_ symmetric in solution (see SI for details). This contrasts with the less constrained analogue P­{N­[*o*-N^*i*^Pr–C_6_H_4_]_2_} reported by Radosevich and co-workers.^49^ Here, a barrier of 5.7 kcal/mol was computed to interconvert the bent shape into its T-shaped conformer (Δ*G*_DFT_ = 4.4 kcal/mol, B3LYP/6–311+G**). Therefore, ligand rigidity is of tremendous importance for lighter Group 15 elements to steer reactivity, while heavier congeners can utilize highly rigid, as well as more flexible platforms to achieve fully planarized geometries.^35^ The frontier molecular orbitals of **1** ^**P**^ and **1** ^**Bi**^ display pronounced similarities, with a ligand-based HOMO while the LUMO and HOMO–1 are best described as bonding and antibonding combinations of the pnictogen’s empty p-orbital and the ligand’s π-sphere, respectively (Figure 2). The HOMO–2 reveals further conjugation between the pnictogen’s vacant p-orbital and the NNN pincer ligand, which is more pronounced in the case of **1** ^**P**^. A comparable ordering and overall shape of the frontier molecular orbitals is likewise observed for **1** ^**Bi_TMS**^ (see SI for comparative density functional theory (DFT) calculations). The electronic structures of **1** ^**P**^ and **1** ^**Bi**^ were further probed by UV/vis spectroscopy and time-dependent density functional theory calculations (TD-DFT, see SI for computational details).

**Figure 2:**
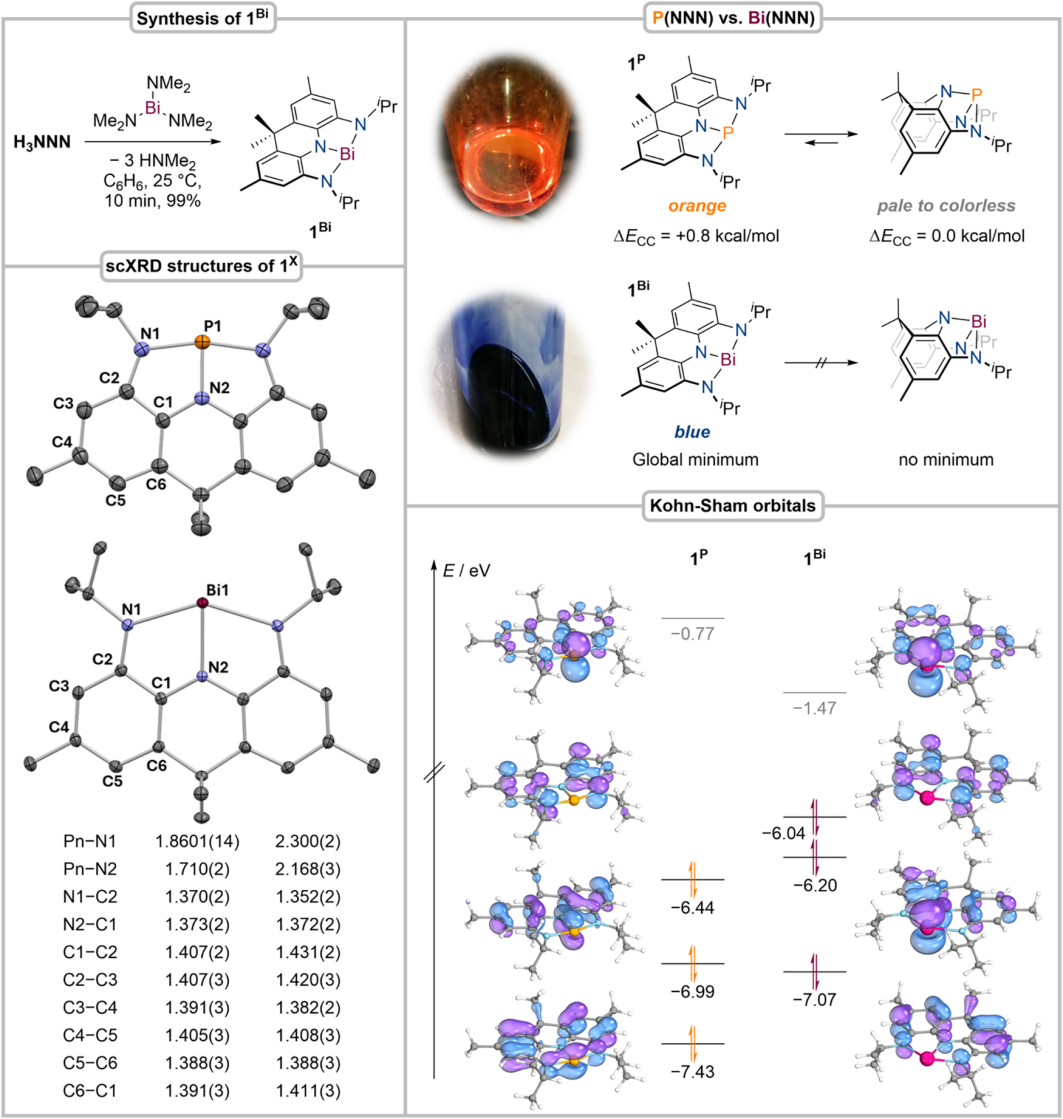
Synthesis of **1** ^**Bi**^ (top left); molecular structure and selected bond lengths of **1** ^**P**^ and **1** ^**Bi**^ in the solid state derived from scXRD, bond lengths in Å, hydrogen atoms were omitted for clarity (bottom left); color and calculated relative stability of different conformers of **1** ^**P**^ (DLPNO–CCSD­(T)/Def2-TZVPP, top right) and **1** ^**Bi**^ ZORA-CAM-B3LYP­(D4)/Def2-TZVPP/CPCM­(Hexane); selected calculated frontier molecular orbitals and corresponding energies of **1** ^**P**^ (CAM-B3LYP/Def2-TZVP/CPCM­(Hexane)) and **1** ^**Bi**^ (ZORA-CAM-B3LYP­(D4)/Def2-TZVPP/CPCM­(Hexane), bottom right); see SI for a detailed comparison of scXRD data and DFT between **1** ^**Bi**^ and **1** ^**Bi_TMS**^ reported by Chitnis and co-workers.^39^

The intense orange and blue colors of **1** ^**P**^ and **1** ^**Bi**^, respectively, originate from two bands in the visible range (λ = 443, 377 nm (**1** ^**P**^); 657, 519 nm (**1** ^**Bi**^)). In line with the observed colors in solution, **1** ^**P**^ possesses a HOMO–LUMO gap larger than that of **1** ^**Bi**^. TD-DFT calculations reveal that the intense colors stem from HOMO–LUMO and HOMO–1–LUMO transitions. Similar optical properties and nature of the frontier molecular orbitals were reported for **1** ^**Bi_TMS**^ (λ = 618 and 522 nm).^39^ Since the experimentally determined Pn–N bond lengths as well as the calculated frontier molecular orbitals indicate increased donation of electron density from the NNN pincer ligand to the bismuth compared to the phosphorus center, we determined the Lewis acidities of **1** ^**P**^ and **1** ^**Bi**^.

Surprisingly, the Gutmann–Beckett acceptor number (AN) of **1** ^**P**^ is just marginally higher (AN_**1P**_ = 12) than that of **1** ^**Bi**^ (AN_**1Bi**_ = 11). This confirms that although a low-lying empty p-orbital results from planarization, this does not necessarily induce Lewis acidic behavior. Nevertheless, Chitnis and co-workers have shown that Bi­(NNN) compounds can act as polymerization catalysts when electron-deficient substituents are introduced, which dramatically alters the Lewis acidity.^37,38^

### Reactivity of 1^Bi^ toward Electrophiles

With **1** ^**Bi**^ in hand, its reactivity toward simple electrophiles was investigated. The nucleophilicity of T-shaped Bi­(NNN) compounds has been indicated by successful coordination of molybdenum(0), tungsten(0), and gold­(I) in recent reports; however, substrate activation involving electron transfer and bond cleavage, e.g., C–X bond splitting reactions reminiscent of Bi­(I) pincer compounds, remains elusive.^36,39,50,51^

The reaction of **1** ^**Bi**^ with the strong acid [H­(OEt_2_)_2_]­[B­(3,5–CF_3_–C_6_H_3_)_4_] (HBArF)^52^ results in an immediate color change from dark blue to purple and affords dark purple crystals in 82% yield after crystallization. The ^1^H NMR spectrum reveals the presence of a *C*_1_ symmetric reaction product on the NMR time scale, featuring a broad resonance of an amino group (δ_H_ = 7.76 ppm), in line with a sharp IR-band at ṽ = 3233 cm^–1^. Consequently, protonation occurs at a ligand side arm to yield the T-shaped Bi­(III) cation [Bi­(^H^NNN)]­[BArF] (**2**, Figure 3). This is confirmed by scXRD, revealing a T-shaped Bi­(III) cation. Ligand-centered protonation results in considerable elongation of the Bi1–N3 bond distance, while the Bi1–N1 bond distance significantly contracts when compared to parent **1** ^**Bi**^. This further leads to pronounced elongation of the N3–C12 bond while the N1–C2 bond retains its partial double-bond character. The preservation of pronounced coupling between the Bi­(III) center and the NNN ligand is evidenced by theoretical calculations. In contrast to **1** ^**Bi**^, the computed HOMO is not ligand centered but rather is best described as a bonding combination between the Bi­(6p) orbital and the pincer ligand, while the LUMO represents the corresponding antibonding combination. The HOMO–1 is ligand centered, and electronic excitation into the LUMO from both of these occupied orbitals causes intense purple color in solution (λ = 866, 796 nm, see SI). To efficiently compare the change in Lewis acidity upon protonation, a modified Gutmann–Beckett method previously reported by Lichtenberg and co-workers, using Me_3_P=S as a reference in dichloromethane, was employed.^53^ Upon protonation, the acceptor number only increases by one unit, demonstrating that transfer of electron density from the redox-active ligand can compensate for the cationic charge, thereby providing valuable insights for the future design of geometrically constrained Bi Lewis acids, as recently investigated by Chitnis and co-workers.^37,38^ To test the stability toward weaker proton sources, we reacted **1** ^**Bi**^ with water, which resulted in a clean and quantitative demetalation and regeneration of **H**_**3**_ **NNN** (Figure 3).

**Figure 3:**
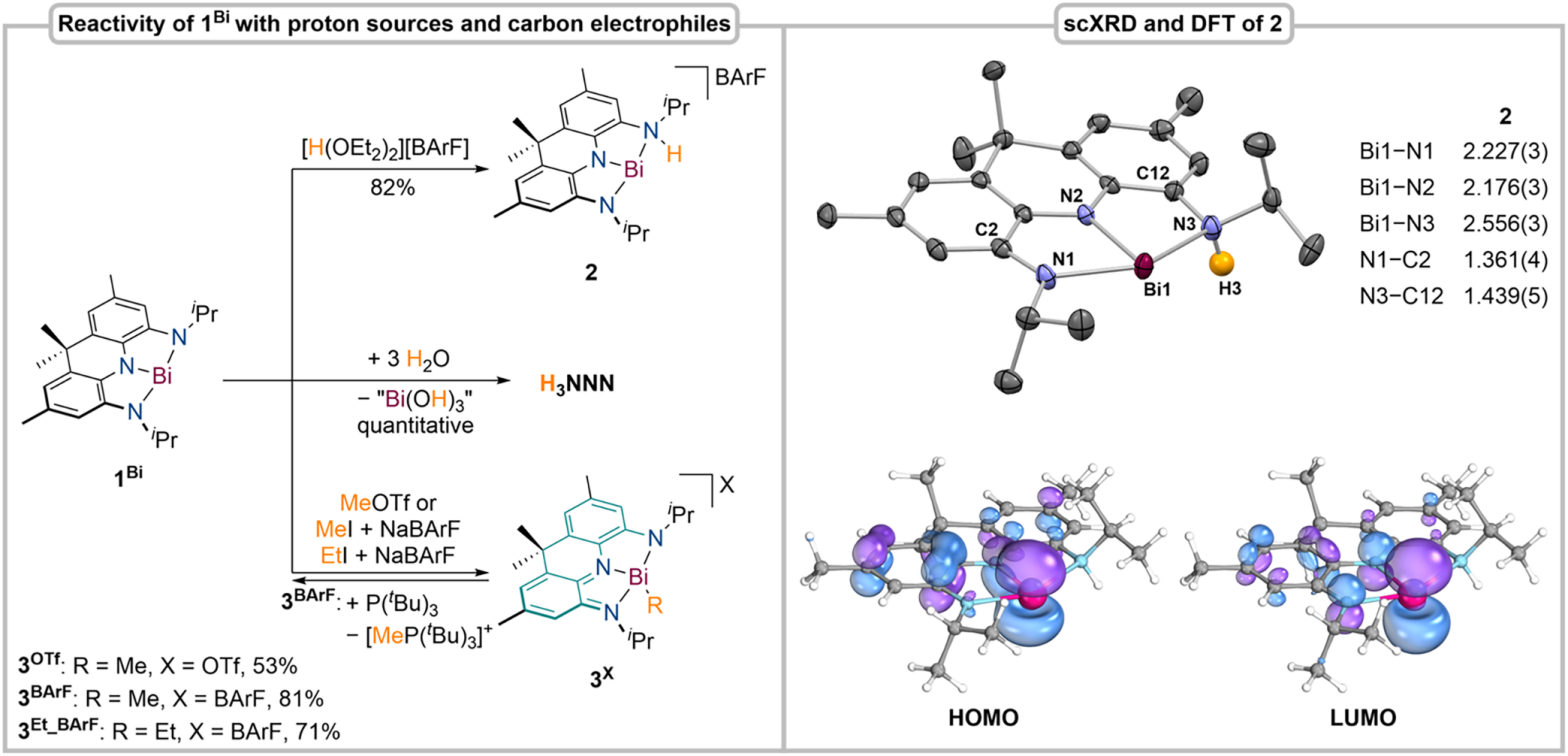
Reactivity of **1** ^**Bi**^ toward electrophiles (left); molecular structure and selected bond lengths of **2** in the solid state derived from scXRD and selected calculated frontier molecular orbitals (ZORA-CAM-B3LYP­(D4)/Def2-TZVPP/CPCM­(Hexane)), bond lengths in Å, nonessential H atoms, and the anion were omitted for clarity (right).

Next, we were interested in whether bismuth-centered reactivity can be realized when carbon electrophiles are employed instead. **1** ^**Bi**^ reacts with methyl triflate at 80 °C for 2.5 h, yielding a dark green compound that can be isolated in moderate yields (Figure 3). The ^1^H and ^13^C­{^1^H} NMR spectra reveal the presence of a *C*_s_ symmetric compound on the NMR time scale with the Bi–Me moiety showing resonances at δ_H_ = 1.60 and δ_C_ = 46.7 ppm.

scXRD confirms Bi-centered methylation, resulting in the distorted bisphenoidal Bi compound [Bi­(Me)­(OTf)­(NNN)] (**3** ^**OTf**^) in which the methyl group occupies the apical position (Figure 3). The alterations in bond distances of the Bi­(NNN) unit and the ligand when compared to **1** ^**Bi**^ give the first indications for the active involvement of the redox-active ligand in these processes. The Bi1–N1 and Bi1–N2 bond lengths elongate by Δ*d* = 0.08 and 0.13 Å, respectively, when compared to **1** ^**Bi**^. This is accompanied by a further contraction of the N1–C2 bond by Δ*d* = 0.05 Å. This reactivity could further be extended to the addition of methyl iodide in the presence of NaBArF to produce the four-coordinate, cationic Bi methyl trisamide [Bi­(Me)­(NNN)]­[BArF] (**3** ^**BArF**^), which features a slightly elongated Bi–Me bond when compared to **3** ^**OTf**^ (Δ*d* = 0.04 Å, Figures 3 and 4) and a shorter Bi1–N2 bond length (Δ*d* = 0.04 Å). **3** ^**BArF**^ features shorter Bi–N bonds when compared to recently reported four-coordinate Bi­(III) cations ligated by monoanionic N,C,N pincer ligands, e.g., [Bi­(CH_2_SiMe_3_)­(2,6-(^t^BuN=CH)_2_C_6_H_3_)]­[OTf] (2.517(8) and 2.495(8) Å) or [Bi­(Cl)­(2,6-(^t^BuN=CH)_2_C_6_H_3_)]­[CB_11_H_12_] (2.465(3) and 2.498(2) Å).^51,54^ Interestingly, when **1** ^**Bi**^ is reacted with methyl iodide in the absence of NaBArF, only sluggish reactivity is observed. Both methylation products are deep green in solution, reminiscent of our recently reported Ta­(V) complexes that featured the employed NNN pincer ligand in its two-electron oxidized state.^43,44^ The presence of a weak Bi–C bond in **3** ^**BArF**^ was confirmed by quantitative methylation of P­(^*t*^Bu)_3_ by **3** ^**BArF**^, regenerating **1** ^**Bi**^ accompanied by the formation of [P­(Me)­(^*t*^Bu)_3_]­[BArF] (Figure 3). We further attempted to deprotonate **3** ^**BArF**^ with potassium bis­(trimethylsilyl)­amide to generate a parent bismuth ylide, which gave no isolable product (see SI). The scope of C–I bond cleavage could further be extended to the activation of Et–I to yield **3** ^**Et_BArF**^ in 71% isolated yield (Figure 3). The bond distances around the Bi center and the NNN pincer ligand are almost identical to the values obtained for methyl analogue **3** ^**BArF**^ (see SI). While the methylation of **1** ^**Bi**^ with Me–I proceeds at room temperature overnight, heating to 60 °C for 2 days was required to drive the ethylation reaction to completion. We further tried to oxidize **1** ^**Bi**^ with Ph_2_Se_2_, which did not result in any reaction even when heated overnight (see SI).

**Figure 4:**
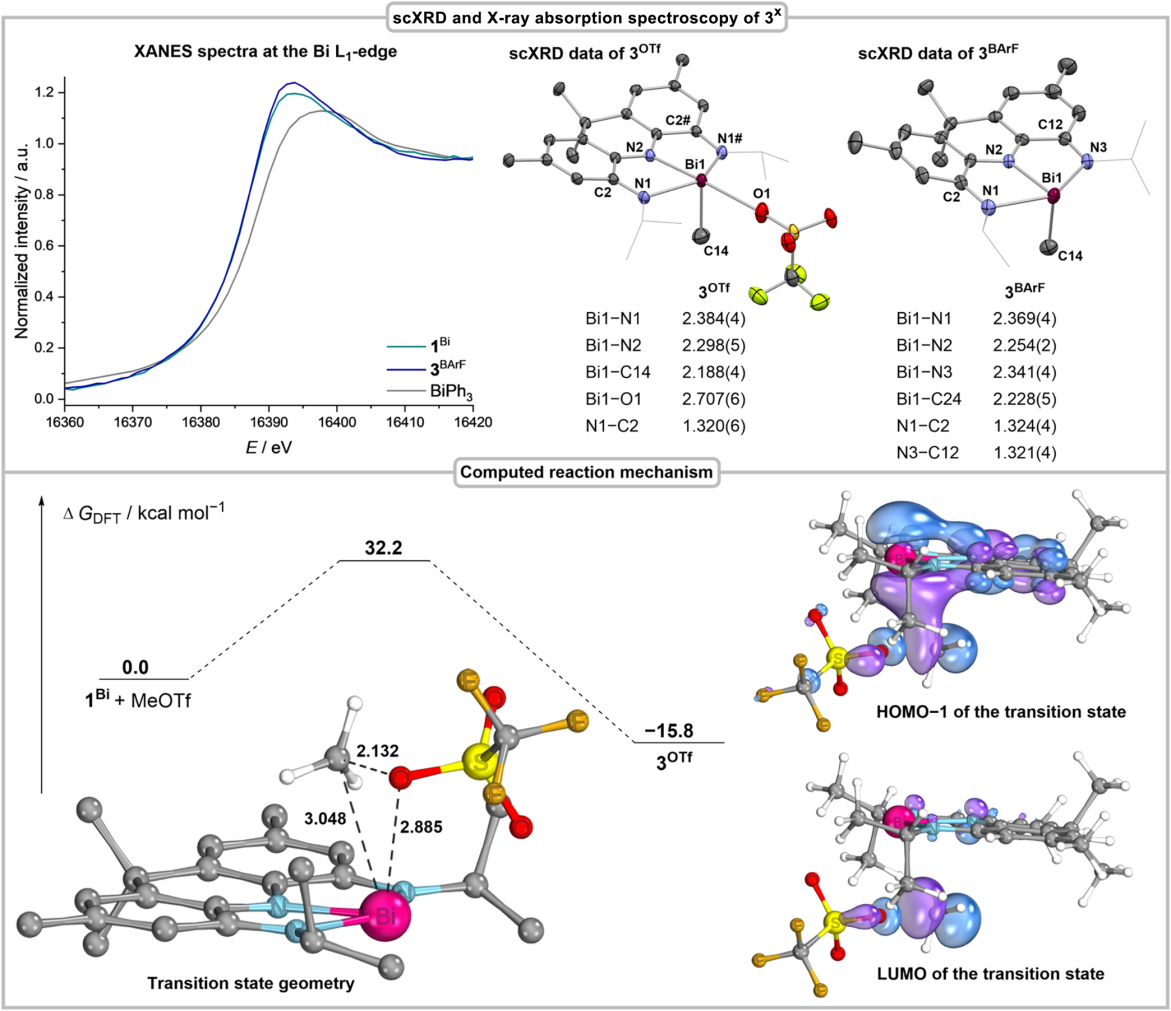
Bi L_1_-edge XANES spectra of **1** ^**Bi**^, **3** ^**BArF**^, and BiPh_3,_ ^36^ as well as molecular structures of **3** ^**BArF**^ and **3** ^**OTf**^ in the solid state derived from scXRD, bond lengths in Å, hydrogen atoms, and the BArF anion were omitted for clarity (top); computed reaction pathway for the methylation of **1** ^**Bi**^ by methyl triflate (ZORA-TPSSh­(D4)/Def2-TZVPP) and selected frontier molecular orbitals of the transition state (ZORA-CAM-B3LYP­(D4)/Def2-TZVP, bottom).

**1** ^**Bi**^ and **3** ^**BArF**^ were further examined by X-ray absorption spectroscopy at the Bi L_1_ and L_3_ edges. Both complexes display identical edge (16388.1 eV) and whiteline (16393.8 eV) energies at the Bi L_1_-edge (Figure 4, see SI). These energies are very close to the XANES data of our recently reported PBiP pincer ligand, which features CH_2_PPh_2_ tethers instead of the N^*i*^Pr groups (Bi L_1_-edge energy: 16388.6 eV).^36^ When compared to the unconstrained Bi­(III) reference compound BiPh_3_, a significant shift to lower energies is observed throughout, in line with recent XANES reports on geometrically constrained P and Bi species.^41,55^ We attribute the identical edge and whiteline energies of **1** ^**Bi**^ and **3** ^**BArF**^ at the Bi L_1_-edge to two contrary effects. While the two-electron oxidation of the ligand reduces the electron density at the Bi center, the introduced electron-donating methyl ligand in **3** ^**BArF**^ compensates for this change. A change of the Bi oxidation state from + III to + V would, in contrast, result in a significant shift of the Bi L_1_-edge energy upon methylation of **1** ^**Bi**^. Fitting the EXAFS spectra of **1** ^**Bi**^ and **3** ^**BArF**^ at the more intense Bi L_3_-edge revealed pronounced differences between both compounds. The Bi–N bond lengths elongate upon methylation, and a new scattering pathway to the methyl group is detected, fully in line with our crystallographic results (see SI).

The reaction of **1** ^**Bi**^ with Me–OTf was further analyzed by DFT calculations, yielding an overall exergonic reaction enthalpy (Δ*G* = −15.8 kcal/mol) with an activation barrier of Δ*G* ^‡^ = 32.2 kcal/mol in line with the need to heat the reaction to 80 °C. The HOMO–1 of the transition state is centered on the Bi­(NNN) fragment, exhibiting large orbital contributions from the Bi­(6p) orbital, while the LUMO is best described as the C–O π*-antibonding orbital of MeOTf (Figure 4). Consequently, the NNN pincer ligand can induce Bi­(I)-like reactivity due to extensive electron donation to the Bi­(III) center to enable C–X bond cleavage in an SN_1_-type reaction under retention of the Bi oxidation state. Upon methylation, the Loewdin atomic charge of the Bi center decreases from −0.16 to −0.11, while the charge of the flanking and central nitrogen atoms of the pincer ligand increases by 0.05 and 0.10, respectively, further supporting ligand-centered redox chemistry. This reactivity profile closely mirrors a recent report on oxidative addition of authentic Bi­(I) pincer compounds that undergo oxidative addition of MeI and MeOTf, forming the corresponding Bi­(III) products.^51^ While MeOTf is sufficiently reactive to oxidize Bi­(III) species to Bi­(V), as shown by Seppelt and co-workers,^56^ in our system, the redox-active ligand appears to be more readily oxidized than the relativistically contracted Bi­(6s) orbital of **1** ^**Bi**^. This represents the first report of transition metal mimetic C–X bond splitting at a Bi­(III) center under active participation of a redox-active ligand, resulting in overall preservation of the Bi­(III) oxidation state.

### Stepwise Reduction of 3^BArF^ and Umpolung of the Bi–Me Moiety

The viability to reduce the oxidized NNN pincer ligand back to its trianionic state was initially assessed by cyclic voltammetry of **3** ^**BArF**^, showing two reversible reductive events in THF at 25 °C (*E*_1/2_ = −0.79, −1.45 V vs Fc^0/+^, Figure 5). We recently reported on a redox series of Ta­(V) complexes with the NNN ligand in all three accessible oxidation states that displayed drastically different colors depending on the ligand redox state. Similar to [TaCl_2_(NPh)­(NNN)] (λ = 900 nm),^43^ the intense green color of **3** ^**BArF**^ arises from a HOMO/LUMO transition at λ = 890 nm that is best described as a ligand-centered π–π* transition (see SI). We therefore performed spectro-electro UV/vis spectroscopy (SEC-UV/vis) to follow the two reductive processes (Figure 5). The first reduction leads to a bleaching of the intense band at λ = 890 nm with an isosbestic point at λ = 480 nm and emergence of a new band at λ = 432 nm that is ascribed to the radical complex [Bi­(Me)­(NNN)]^•^ (**4**). Upon two-electron reduction, the anionic Bi methyl compound [Bi­(Me)­(NNN)]^−^ (**5**) is formed, associated with a rising band at λ = 391 nm. This gradual blue shift upon stepwise ligand reduction is reproduced by TD-DFT calculations (see SI). The computed structures of **3** ^**+**^ **–5** further reveal distinct bond alteration in the Bi–N/Me and ligand bond lengths (Figure 5). Stepwise reduction of **3** ^**+**^ leads to a gradual increase in the Bi–Me bond lengths, while the Bi–N lengths contract. The bond lengths within the pincer ligand suggest that the reductive processes are mainly ligand centered due to successive contraction of the C2/12–N1/3 bonds, whereas the C1/7–C2/12 bonds gradually shorten due to more pronounced double-bond character. This strongly indicates that redox chemistry occurs exclusively at the NNN pincer ligand. Going from **3** ^**+**^ to **5**, the computed Loewdin atomic charge of Bi only changes from −0.11 to −0.31, with the charge of carbon of the bismuth-bound methyl group remaining almost constant (−0.01 to – 0.08).

**Figure 5:**
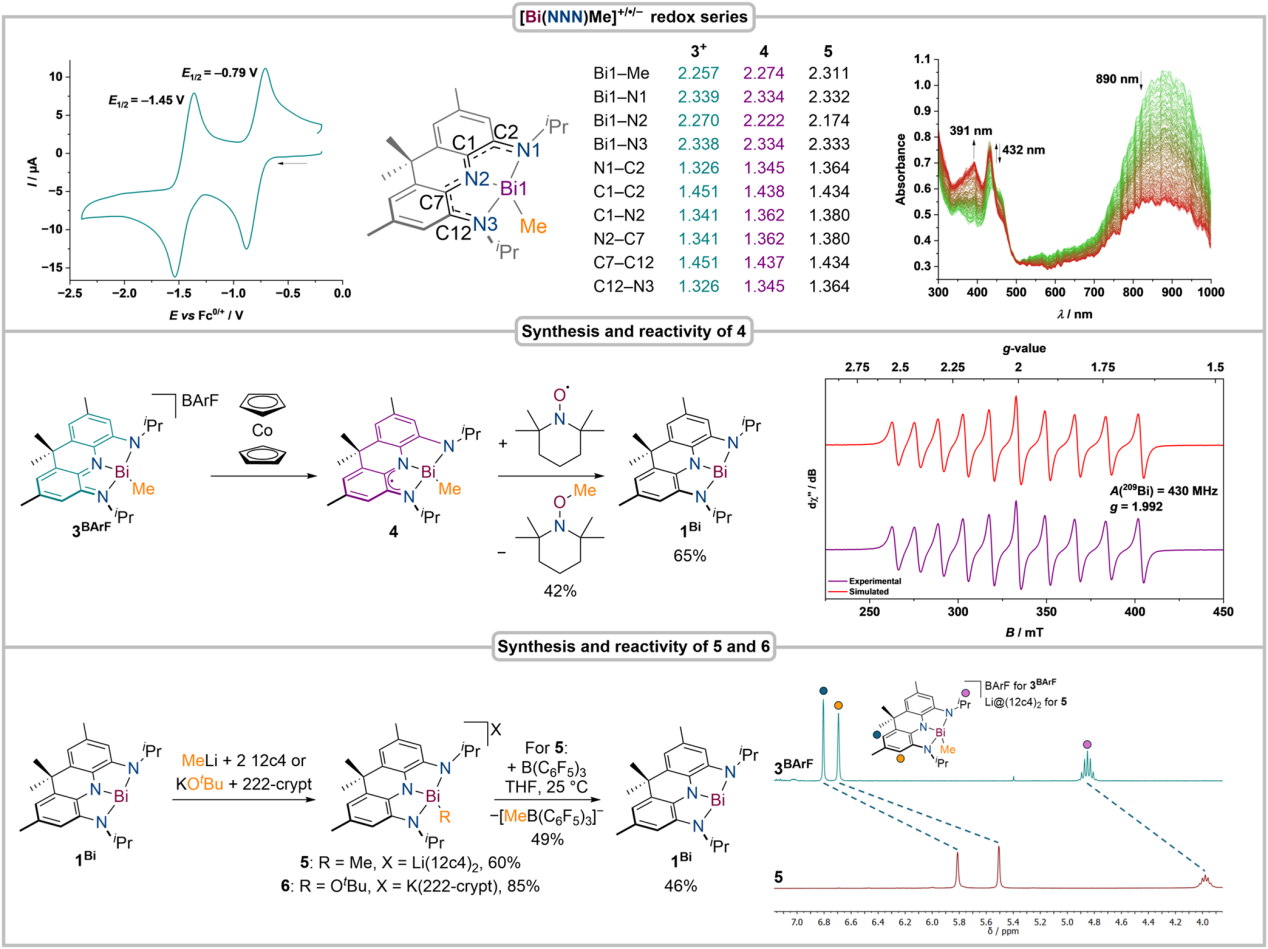
Cyclic voltammogram of **3** ^**BArF**^, THF, 1 mM, 0.1 [NBu_4_]­[PF_6_], 25 °C; computed bond lengths of **3** ^**+**^, **4**, and **5** (ZORA-TPSSh­(D4)/Def2-TZVPP); SEC-UV/vis of **3** ^**BArF**^ in THF, 1 mM, 0.1 M [NBu_4_]­[PF_6_], 25 °C, 100 mV/s (top); synthesis and methyl radical transfer reactivity of **4**; EPR spectrum of **4**, toluene, 25 °C (middle); synthesis of **5** and **6** as well as methyl anion transfer reactivity of **5**; ^1^H NMR spectra of **3** ^**BArF**^ and **5**, THF-*d*_8_, 25 °C (bottom).

**4** can be generated *in situ* upon reduction of **3** ^**BArF**^ with cobaltocene. The X-band electron paramagnetic resonance (EPR) spectrum features a broad signal with a *g-*value (1.992) close to the value of the free electron, indicative of the presence of a ligand-centered radical (Figure 5). Bi hyperfine interactions (HFIs) produce a 10-line pattern (^209^Bi: 100% natural abundance, *I* = −9/2) with a coupling constant of *A*(^209^Bi) = 430 MHz, suggesting minimal localization of the unpaired electron at the bismuth center (Figure 5). This is further corroborated by spin-density calculations that suggest 2% spin localization on the bismuth center (see SI for the spin density plot). This is in stark contrast to authentic Bi radicals, which typically exhibit coupling constants in the GHz range only observable at extremely low temperatures.^23,24,41,57–66^ When solutions of **4** are kept at 25 °C, partial regeneration of **1** ^**Bi**^ is detected by ^1^H NMR spectroscopy due to the loss of a methyl radical. The lability of Bi–Me bonds toward homolysis has recently also been demonstrated by the Lichtenberg group by Me radical generation directly from BiMe_3_.^67,68^

No experimental evidence of the presence of methane or ethane originating from hydrogen atom abstraction or radical dimerization could be detected. Lastly, we aimed to access the anionic bismuth methyl species **5**. This highly reactive, deep red compound can be cleanly prepared upon reaction of **1** ^**Bi**^ with MeLi in the presence of two equivalents of 12c4 (1,4,7,10-tetraoxacyclododecane) in 60% isolated yield (Figure 5). This further confirms the unique amphiphilicity of T-shaped Bi­(NNN) compounds and a suitable description of such systems as Bi­(III) trisamides. While we succeeded in designing a synthetic route to yield pure **5**, as confirmed by elemental analysis, high-quality single crystals suitable for scXRD could not be obtained due to the tremendous sensitivity of **5** toward oxygen and moisture. Nevertheless, we were able to obtain a data set that confirms the overall connectivity of atoms within the **5** (see SI). Comparing the ^1^H NMR data of **5** and **3** ^**BArF**^ reveals how ligand oxidation directly impacts the chemical shift of ligand protons. The aromatic C–H resonances shift by Δδ_H_ ∼1 ppm between **3** ^**BArF**^ and **5**, while the C–H protons of the isopropyl groups display a shift of Δδ_H_ ∼ 0.8 ppm (Figure 5). The cyclic voltammogram of **5** in THF at room temperature shows the same redox events as **3** ^**BArF**^ (see SI). With methyl cation and radical transfer from the Bi­(NNN) platform realized, we last evaluated if **5** can be a source of a methyl anion. Mixing **5** with Lewis acid B­(C_6_F_5_)_3_ leads to regeneration of **1** ^**Bi**^ (46%) accompanied by formation of [Li­(12c4)_2_]­[B­(Me)­(C_6_F_5_)_3_]^−^ (49%, Figure 5). We ascribe the modest yield of this reaction to the high sensitivity of **5** in solution. This transformation nevertheless establishes the anion transfer reactivity of **5** and thereby completes a unique series of redox-switchable Bi–Me compounds.

The reactivity of **1** ^**Bi**^ toward nucleophiles could further be extended to Bi–O bond formation upon reaction of **1** ^**Bi**^ with KO^*t*^Bu in the presence of the sequestering agent 222-crypt (4,7,13,16,21,24-hexaoxa-1,10-diazabicyclo[8.8.8]­hexacosane) to give [K@222-crypt]­[Bi­(O^*t*^Bu)­(NNN)] (**6**) in 85% isolated yield (Figure 5). The ^1^H and ^13^C­{^1^H} NMR spectra are consistent with the presence of a *C*_S_ symmetric species on the NMR time scale, with the *tert*-butoxide ligand showing resonance at δ_H_ = 0.61 ppm in the proton NMR. **6** readily hydrolyzes to give **1** ^**Bi**^ when exposed to trace amounts of water (see SI). The molecular structure of **6** derived by scXRD shows pronounced differences when compared to that of **3** ^**BArF**^ (Figure 6). The flanking Bi–N bonds are shortened by Δ*d* ≈ 0.05 Å, while the central Bi–N bond is 0.08 Å shorter. In contrast, N1–C2 and N3–C12 are the longest among the series of **1** ^**Bi**^, **3** ^**OTf**^, **3** ^**BArF**^, and **3** ^**Et_BArF**^, in line with the ligand being in its trianionic, fully reduced oxidation state.

**Figure 6:**
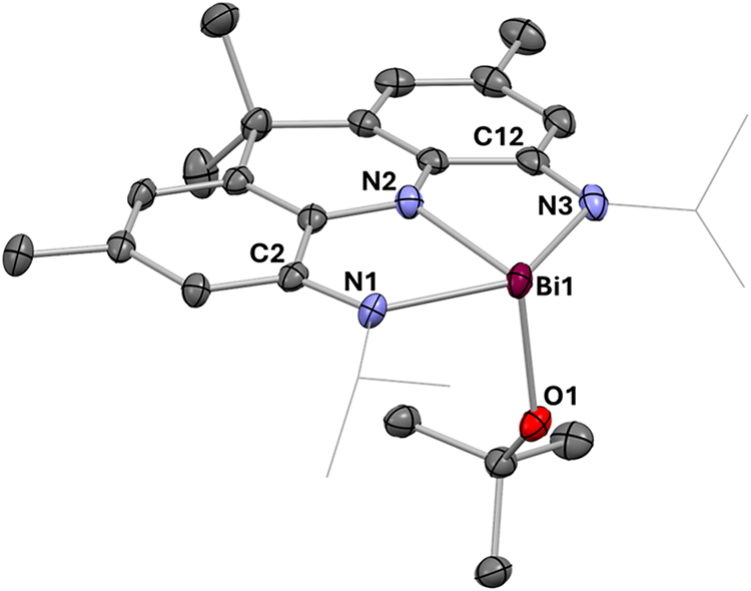
Molecular structure of **6** in the solid state derived from scXRD, H atoms, solvent molecules, and the cation were omitted for clarity. Bond lengths of **6** in Å: Bi1–N1 2.319(3); Bi–N2 2.176(3); Bi1–N3 2.294(4); Bi1–O1 2.154(3); N1–C2 1.370(4); N3–C12 1.376(5).

## Conclusion

In conclusion, we report the first example of an ambiphilic Bi­(III) compound that undergoes the cleavage of C–I and C–OTf bonds mediated by a redox-active pincer ligand. While bond formation occurs exclusively at the Bi center, the required electrons for this reaction are delivered by the pincer ligand, which was confirmed via scXRD, X-ray absorption spectroscopy, and DFT calculations. Starting from the cationic Bi­(III) methyl compound **3** ^**BArF**^, stepwise ligand-centered reduction gives the corresponding radical **4** as well as the anionic species **5**. Depending on the ligand oxidation state, the [BiMe­(NNN)] platform can serve as a source of methyl cations, radicals, and anions, which represents a rare case of redox-induced reactivity control in p-block chemistry. By mimicking transition metal-like reactivities through ligand-centered redox events, this work underscores the powerful synergy between redox-active ligands and VSEPR noncompliant pnictogen geometries, leading to the transfer of established transition metal chemistry to main group metals. This new reactivity vector in Bi-mediated substrate activation opens up new design principles for future applications of Bi compounds in catalysis. Steering the reactivity of metal-bound alkyl groups via ligand-centered redox chemistry has been tremendously successful in transition metal chemistry, and similar transformations based on Bi­(NNN) species are currently being investigated by us.^3,6,69–71^

## Supplementary Material





## Notes

Coordinates of computed species (XYZ)
Synthetic details; recorded NMR, IR, UV/vis, XAS, and EPR spectra; SQUID and CV data; scXRD details; computational details (PDF)

## Abbreviations

EXAFS: extended X-ray absorption fine structure
HOMO: highest occupied molecular orbital
LUMO: lowest unoccupied molecular orbital
OTf: trifluoromethanesulfonate
VSEPR: valence shell electron pair repulsion
XANES: X-ray absorption near edge structure
