# Supplementary material for: Ambiphilic Reactivity and Switchable Methyl Transfer at a T‑Shaped Bi(NNN) Complex Enabled by a Redox-Active Pincer Ligand
Source: J Am Chem Soc. 2026 Jan 6;148(2):2683–92. doi: 10.1021/jacs.5c18955 (PMC12833801; doi:10.1021/jacs.5c18955)
Supplement: Supplementary file 2 [file ja5c18955_si_002.pdf]

Electronic Supporting Information

Ambiphilic Reactivity and Switchable Methyl Transfer at a T-shaped Bi(NNN) Complex Enabled by a Redox-Active Pincer Ligand

Sotirios Pavlidis,[a] Eric W. Fischer,[a] Amanda Opis-Basilio,[a] Ayan Bera,[a] Ana Guilherme Buzanich,[b] María Álvarez-Sánchez,[a] Severin Wittek,[a] Franziska Emmerling,[b] Kallol Ray,[a] Michael Roemelt,[a*] Josh Abbenseth[a,c,*]

[a] S. Pavlidis, Dr. E. W. Fischer, A. Opis-Basilio, A. Bera, María Álvarez-Sánchez,[a] Severin Wittek, Prof. K. Ray, Prof. M. Roemelt, Dr. J. Abbenseth
Institut für Chemie Humboldt-Universität zu Berlin Brook-Taylor-Str. 2, 12489 Berlin, Germany.
E-Mail: michael.roemelt@hu-berlin.de

[b] Dr. A. Guilherme Buzanich, Prof F. Emmerling
Department of Materials Chemistry Federal Institute for Materials Research and Testing Richard-Willstätter-Str. 11, 12489 Berlin, Germany

[c] Department of Chemistry, The University of Manchester, Oxford Road, Manchester, M13 9PL, U.K.
E-Mail: josh.abbenseth@manchester.ac.uk

Table of Contents

Materials and Methods S4

Synthetic Procedures S6

Improved Synthesis of 1^{P} S6

Synthesis of 1^{Bi} S7

Synthesis of 2 ..... S8

Synthesis of 3^{OTf} S9

Synthesis of 3^{BArF} S10

Synthesis of 3^{Et_BArF} S11

Generation of 4 *in situ* S12

Synthesis of 5 ..... S13

Synthesis of 6 ..... S14

Me^{+}, $Me^{•}$, and Me^{-} Transfer S15

Transfer of Me^{+} from 3^{BArF} to $P(^{t}Bu_3)_3$ S15

Transfer of $Me^{•}$ from 4 to TEMPO S15

Transfer of Me^{-} from 5 to $B(C_6F_5)_3$ S16

Additional Reactions S16

Reaction of 1^{Bi} with Water S16

Reaction of 1^{Bi} with Diphenyl diselenide S17

Reaction of 3^{BArF} with KHMDS S17

NMR, IR and UV/Vis Spectroscopy S18

Determination of Lewis Acidities *via* the Gutmann-Beckett Method S18

Spectroscopy of 1^{P} S21

Spectroscopy of 1^{Bi} S22

Spectroscopy of 2 ..... S26

Spectroscopy of 3^{OTf} S31

Spectroscopy of 3^{BArF} S35

Spectroscopy of 3^{Et_BArF} S40

NMR Spectroscopy of the One-Electron Reduction of 3^{BArF} by $CoCp_2$ S44

NMR and IR Spectroscopy of 5 ..... S46

NMR and IR Spectroscopy of 6 ..... S50

Spectroscopic Data for the Me^{+}, $Me^{•}$, and Me^{-} Transfer Reactivity S54

Spectroscopy of Me^+ Transfer from 3^{BArF} to $P(^tBu_3)_3$ S54
Spectroscopy of $Me^•$ Transfer from 4 to TEMPO S58
Spectroscopy of Me^- Transfer from 5 to $B(C_6F_5)_3$.... S62
Spectroscopic Data for Additional Reactions S66
Spectroscopy of 1^{Bi} with Water S66
Spectroscopy of 1^{Bi} with Diphenyl Diselenide S68
Spectroscopy of 3^{BArF} with KHMDS S70
Single Crystal X-ray Crystallography S72
1^{Bi} S72
2 .... S74
3^{OTf} S78
3^{BArF} S80
3^{Et_BArF} S85
5 .... S91
6 .... S93
EPR Spectroscopy S99
X-ray Absorption Spectroscopy S100
Density Functional Theory Calculations S104
1^P S104
1^P S105
1^{Bi} S109
Comparison Between 1^{Bi} and 1^{Bi_TMS} S111
2 .... S113
3^+ S115
Transition State of the Methylation of 1^{Bi} by MeOTf S117
4 .... S118
5 .... S121
Spin Properties / Charges of 1 and 3^+ – 5 .... S123
Comparison of Structural Parameters of the Redox Series of 3^+ - 5 .... S123
Electrochemistry S125
References S128

Materials and Methods

NMR spectra were recorded on AVANCE II 300 MHz, Bruker Avance 400 MHz, Bruker Avance III 500 MHz and Bruker Avance 600 MHz NMR spectrometers. Chemical shifts (δ) are referenced to the signal of the deuterated solvent.

IR spectra were recorded on a Bruker ALPHA spectrometer with an ATR sampling unit.

Elemental analyses were performed with a HEKA Euro 3000EA elemental analyzer.

XAS measurements were performed at the BAMline located at BESSY-II storage ring operated by Helmholtz-Zentrum Berlin (HZB).[1] The incident energy was tuned by a double crystal monochromator in a Si(111) arrangement (delivering an intrinsic resolution of $\Delta E/E = 2x10^{-4}$). The measurements were carried out in transmission using three ionization chambers to measure the signal before the sample (I_0), after the samples (I_1) and after a Bi metal reference foil (I_2). The energy range scanned comprised in total 1000 eV, starting at –200 eV below and ending at 800 eV above the Bi L_1- and L_3-edges in a continuous mode. For XANES measurements we used equidistant 0.25 eV energy steps, and for EXAFS 0.5 eV. The samples were prepared and diluted with boron nitride in 2 mm thick sample holders, to produce an absorption edge jump of 2. A total of 3 repetitions were collected for each sample. The acquired spectra were extracted, calibrated, and normalized using the IFFEFIT software package containing ATHENA and ARTEMIS software.[2] The Fourier Transformed EXAFS data were made in k-space (between 1.5-12 $Å^{-1}$)

Continuous wave (CW) X-band electron paramagnetic resonance (EPR) spectra were collected on a Bruker EMXplus Instrument at a frequency of ~9.35 GHz (X-Band), equipped with the Bruker ER4119-HS probehead, in perpendicular polarization mode. Measurements were carried out at 25 °C. EPR simulation of the experimental features of **4** was performed using the software EasySpin (version 6.0.0) supported by MATLAB.[3]

Cyclic Voltammetry (CV) was carried out with a CHI 600E potentiostat using 3 mm diameter glassy carbon disk electrodes (ALS Co Ltd.) as working and Platinum wire (length 5 cm, diameter 0.5 mm; ALS Co Ltd.) as counter electrodes. Prior to use, electrodes were polished with 0.05 µm alumina suspensions (CH Instruments Inc., USA). $Ag/AgNO_3$ (10 mM $AgNO_3$ and 0.1 M $[NBu_4][PF_6]$ in THF) was used as the reference electrode. CV measurements were performed in a three-necked glass cell under an Ar atmosphere. Two milliliters of 1 mM compound solution in THF was taken in the presence of 100 mM $[NBu_4][PF_6]$ as the supporting electrolyte. Unless noted otherwise, a scan rate of 100 mV/s was applied. Ferrocene (Fc) was used as an internal reference, and the potential scale is normalized with respect to the potential of the $Fc^{+/0}$ couple.

Spectro-electrochemical measurements were performed with a Metrohm Autolab PGSTAT 204 potentiostat/galvanostat equipped with a Pt net as a working electrode, glassy carbon as counter electrode and Ag wire pseudo-reference electrode. The supporting electrolyte tetrabutylammonium hexafluorophosphate ($[NBu_4][PF_6]$) (>99%, Sigma) was used as received.

UV/Vis absorption spectro-electrochemistry measurements in parallel configuration (the light beam passes parallel and close to the electrode surface) were performed using an AUTOLAB spectro-photometer UA in the UV/Vis/NIR wavelength range (from 200 nm to 1100 nm). UV/Vis spectrometer was properly synchronized with the Metrohm AUTOLAB PGSTAT 204 potentiostat. All UV/Vis data were supported by AVANTES AVASOFT 8.11 and the electrochemical data were handled by NOVA 2.1.5 software. The light beam, supplied by a light source (AUTOLAB D/HAL Light Source, METROHM), was conducted to the spectro-electrochemical cell by a 200 μm bare optical fiber (METROHM), and collected from the spectroel-ectrochemical cell to the spectrometer by a 200 μm bare optical fiber. (The standard dimensions of the optical fibers is 2 m in length and 200 μm in diameter. The fibers are fitted with SMA-905 connectors on both ends).

All experiments with air-sensitive compounds were carried out in a glovebox or in a fume hood employing Schlenk techniques under a dry Ar atmosphere. Traces of water and oxygen were removed *via* heating of glassware under vacuum prior to use.

All solvents except THF were dried and degassed by a MBraun solvent purification system. THF was dried over sodium, distilled and stored over molecular sieves. Deuterated solvents were degassed *via* three freeze-pump-thaw cycles and stored over molecular sieves.

All commercially available chemicals were used without purification unless otherwise noted. Boron nitride was dried at 120 °C for seven days under a dynamic vacuum. $BiCl_3$ was sublimed and grinded under an Ar atmosphere prior to use. 2,4,6-Tris-tert-butylphenoxyl radical ($TEMPO^•$) was sublimed under an Ar atmosphere. Tetrabutylammonium hexafluorophosphate ($[NBu_4][PF_6]$) was dried at 120 °C for three days under a dynamic vacuum prior to use. Sodium tetrakis[3,5-bis(trifluoromethyl)phenyl]borate (NaBArF) was purified according to literature.[4]

Brookhart's acid ($[H(OEt_2)_2][BArF_{24}]$),[5] KC_8,[6] $Bi(NMe_2)_3$[7] and (**H_3NNN**)[8,9] were synthesized according to literature procedures.

Synthetic Procedures

Improved Synthesis of 1^P

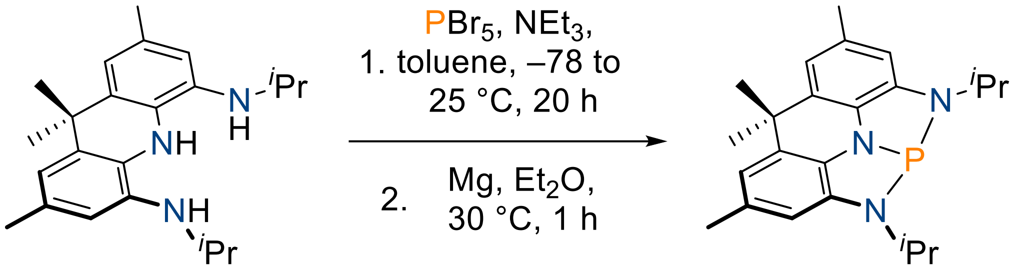

A suspension of PBr_5 (370 mg, 859 µmol, 1.01 eq.) in toluene (10 mL) is cooled to −78 °C and NEt_3 (0.6 mL, 4.32 mmol, 5.06 eq.) is added. A solution of **H_3NNN** (300 mg, 853 µmol, 1.00 eq.) in toluene (10 mL) is added dropwise under intense stirring. The green reaction mixture is slowly warmed to 25 °C and stirred for 20 h under the exclusion of light. The resulting brown reaction suspension is filtered and extracted with toluene (2 x 10 mL). The solvent is removed *in vacuo* and the dark brown solid is washed with Et_2O (3 x 2 mL) at 0 °C affording a bright orange powder. After removing residual solvent *in vacuo*, **1^{PBr2}** is obtained as a bright orange powder (323 mg, 599 µmol, 70%).

1^{PBr2} (90.0 mg, 167 µmol, 1.00 eq.) and Mg powder (81.0 mg, 3.33 mmol, 19.9 eq.) are suspended in Et_2O (10 mL) and stirred at 30 °C for 1 h. The solvent of the bright orange solution is removed *in vacuo*. The orange residue is extracted with hexane (3 x 5 mL) followed by removal of the solvent *in vacuo* and lyophilization out of a concentrated benzene solution at 50 °C. **1^P** is obtained as a bright orange powder (59.4 mg, 157 µmol, 94%).

^{1}H NMR spectra matched published spectra.[10]

For the determination of the acceptor number of **1^P** *via* Gutmann-Beckett method see

Figure S1. For the UV/Vis spectrum see **Figure S7**.

UV/Vis: (hexane, 1 × 10^{-5} M, 25 °C): λ_{max} (nm) = 443, 377, 311.

Synthesis of 1^{Bi}

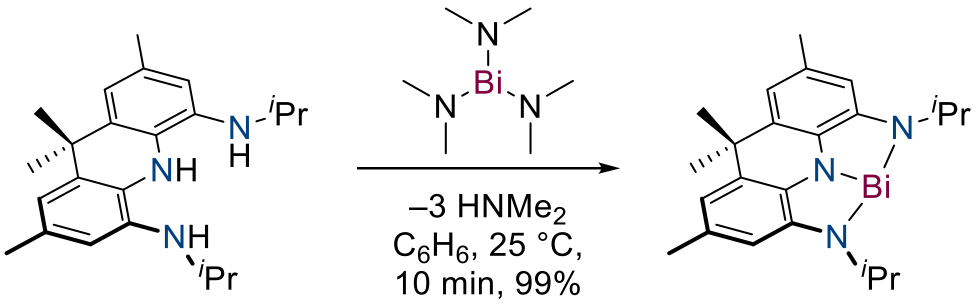

H_3NNN **1^{Bi}**

H_3NNN (150 mg, 427 µmol, 1.00 eq.) is dissolved in benzene (15 mL) and added to a Schlenk flask containing $Bi(NMe_2)_3$ (147 mg, 431 µmol, 1.01 eq.) dissolved in benzene (5 mL). The dark blue reaction solution is stirred for 10 min at 25 °C, followed by lyophilization overnight. **1^{Bi}** is obtained as a fine purple/blue powder (236 mg, 423 µmol, 99%).

See **Figure S8-Figure S12**, **Figure S13** and **Figure S14** for NMR, IR and UV/Vis spectra, respectively. For the determination of the acceptor number (AN) *via* Gutmann-Beckett method see **Figure S2+Figure S4**.

NMR: (CD_2Cl_2, 25 °C) ^{1}H NMR (500 MHz) δ(ppm) = 6.83 (m, 2H, $C^{Ar}H$), 6.59 (d, $^3J_{HH}$ = 1.5 Hz, 2H, $C^{Ar}H$), 5.84 (hept, $^3J_{HH}$ = 6.2 Hz, 2H, $NCH(CH_3)_2$), 2.80 (s, 6H, $C^{Ar}CH_3$), 1.75 (s, 6H, $C^{quart.}(CH_3)_2$), 1.68 (d, $^3J_{HH}$ = 6.2 Hz, 12H, $NCH(CH_3)_2$).

$^{13}C\{^1H\}$ NMR (126 MHz) δ(ppm) = 153.58 (C^{Ar}), 139.89 (C^{Ar}), 137.93(C^{Ar}), 134.48 (C^{Ar}), 114.95($C^{Ar}H$), 111.14 ($C^{Ar}H$), 51.88 ($CH(CH_3)_2$), 37.30 ($C^{quart.}(CH_3)_2$), 36.85 ($C^{quart.}(CH_3)_2$), 27.64 ($NCH(CH_3)_2$), 21.28 ($C^{Ar}CH_3$).

ATR-IR (solid): $\tilde{\nu}$ (cm^{-1}) = 2954 (m), 1599 (m), 1454 (m), 1291 (s), 821 (s).

UV/Vis: (hexane, 1 × 10^{-5} M, 25 °C): λ_{max} (nm) = 657, 519, 278.

Elem. Anal. found (calcd) for ($C_{44}H_{58}BiN_3$): C: 49.38 (49.55), H: 5.50 (5.42), N: 7.39 (7.54).

Synthesis of 2

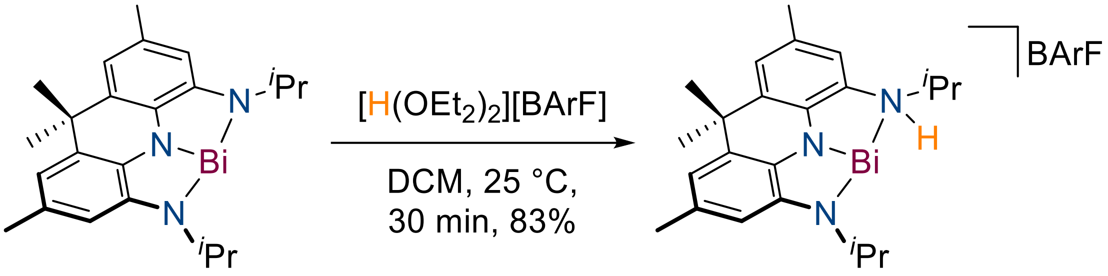

1^{Bi} **2**

1^{Bi} (54.0 mg, 96.9 µmol, 1.00 eq.) is dissolved in DCM (10 mL) and [H(OEt$_2$)$_2$][BArF] (98.0 mg, 96.8 µmol, 1.00 eq.) is added. The reaction solution is stirred at 25 °C for 1 h. After removing all volatiles *in vacuo*, the purple solid is washed with hexane (3 x 5 mL), followed by extraction with DFB (3 mL) and layering with hexane (17 mL). After recrystallization for 4 d at –30 °C, the supernatant is decanted and the violet crystalline residue is washed with hexane (3 x 5 mL). Removing residual solvent *in vacuo* yields **2** (113.7, 80.0 µmol, 83%) as dark-violet crystals.

See **Figure S15-Figure S22**, **Figure S23** and **Figure S24** for NMR, IR and UV/Vis spectra, respectively. For the determination of the acceptor number (AN) of **2** *via* Gutmann-Beckett method see **Figure S5**.

NMR: (THF-d$_8$, 25 °C) ^{1}H NMR (500 MHz) δ(ppm) = 7.77 – 7.80 (m superimposed, 8H, C^{BArF}*H*), 7.76 (hept superimposed, $^{3}J_{HH}$ = 6.3 Hz, 1H, NC*H*(CH$_3$)$_2$), 7.63 (m, 1H, C^{Ar}*H*), 7.58 (s, 4H, C^{BArF}*H*), 7.45 (s, 1H, C^{Ar}*H*), 6.83 (s, 1H, C^{Ar}*H*), 6.42 (d, $^{4}J_{HH}$ = 1.3 Hz, 1H, C^{Ar}*H*), 5.49 (br. s, 1H, N*H*), 4.41 (dhept, $^{3}J_{HH}$ = 6.4, $^{3}J_{HH}$ = 2.4 Hz, 1H, NHC*H*(CH$_3$)$_2$), 3.16 (s, 3H, C^{Ar}C*H*$_3$), 2.83 (s, 3H, C^{Ar}C*H*$_3$), 1.80 – 1.84 (two d superimposed, $^{3}J_{HH}$ = 6.4 Hz, 6H, NCH(C*H*$_3$)$_2$), 1.69 (s, 6H, C$^{quart.}$(C*H*$_3$)$_2$), 1.59 (d, $^{3}J_{HH}$ = 6.3 Hz, 3H, NHCH(C*H*$_3$)$_2$), 1.03 (d, $^{3}J_{HH}$ = 6.3 Hz, 3H, NHCH(C*H*$_3$)$_2$).

^{13}C{^{1}H} NMR (126 MHz) δ(ppm) = 161.83 ($^{1}J_{BC}$ = 50.0 Hz, BC^{BArF}), 155.81 (C^{Ar}), 151.21 (C^{Ar}), 140.98 (C^{Ar}), 140.71 (C^{Ar}), 139.86 (C^{Ar}), 138.55 (C^{Ar}), 138.30 (C^{Ar}), 136.38 (C^{Ar}), 135.77 (m, *C*^{BArF}H), 130.20 (qq, $^{2}J_{CF}$ = 31.5 Hz, $^{4}J_{CF}$ = 2.9 Hz, *C*^{BArF}CF$_3$), 125.85 (*C*^{Ar}H), 125.69 (q, $^{1}J_{CF}$ = 272.3 Hz, C^{BArF}*C*F$_3$), 125.43 (*C*^{Ar}H), 125.33 (*C*^{Ar}H), 118.36 – 117.10 (hept, $^{3}J_{CF}$ = 3.89 Hz *C*^{BArF}(CF$_3$)$_2$), 114.98 (*C*^{Ar}H), 52.82 (NH*C*H(CH$_3$)$_2$), 52.05 (N*C*H(CH$_3$)$_2$), 36.86 (C$^{quart.}$(*C*H$_3$)$_2$), 36.40 (C$^{quart.}$(*C*H$_3$)$_2$), 36.22 (*C*$^{quart.}$(CH$_3$)$_2$), 29.40 (NCH(*C*H$_3$)$_2$), 24.33 (NHCH(*C*H$_3$)$_2$), 20.15 (C^{Ar}*C*H$_3$), 20.13 (C^{Ar}*C*H$_3$), 20.00 (NHCH(*C*H$_3$)$_2$).

^{19}F{^{1}H} NMR (470 MHz) δ(ppm)= −63.75.

^{11}B NMR (160 MHz) δ(ppm)= −6.85 (br).

UV/Vis: (DCM, 5 × 10^{-5} M, 25 °C): λ_{max} (nm) = 866, 796, 615, 532, 457, 403, 305.

ATR-IR (solid): $\tilde{\nu}$ (cm^{-1}) = 3233 (w), 1353 (s), 1273 (s), 1115 (s), 885 (s), 667(s).

Elem. Anal. found (calcd) for ($C_{55}H_{43}BBiF_{24}N_3$): C: 46.33 (46.47), H: 2.98 (3.05), N: 2.88 (2.96).

Synthesis of 3^{OTf}

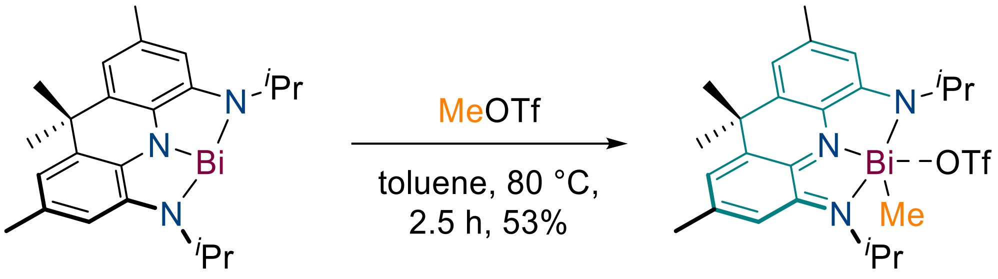

1^{Bi} **3^{OTf}**

1^{Bi} (54.3 mg, 97.4 μmol, 1.00 eq.) is dissolved in toluene (5 mL) followed by addition of methyl trifluoromethanesulfonate (MeOTf) (11 μL, 101 μmol, 1.04 eq). The reaction is heated to 80 °C for 2.5 h. After cooling to 25 °C, all volatiles are removed *in vacuo*, followed by extraction with DCM (5 mL). The extract is layered with hexane (20 mL) and stored at −30 °C for 4 d. After filtration at −78 °C the dark green solid is washed with hexane (2 x 5 mL) at 25 °C. After removing residual solvent *in vacuo*, **3^{OTf}** is obtained as dark green crystals (37.5 mg, 52.0 μmol, 53%).

See **Figure S25-Figure S30**, **Figure S31** and **Figure S32** for NMR, IR and UV/Vis spectra, respectively.

NMR: (CD_2Cl_2, 25 °C) ^{1}H NMR (500 MHz) δ(ppm) = 6.70 (d, $^3J_{HH}$ = 1.1 Hz, 2H, $C^{Ar}H$), 6.64 (m, 2H, $C^{Ar}H$), 4.83 (hept, $^3J_{HH}$ = 6.5 Hz, 2H, NCH(CH$_3$)$_2$), 2.31 (s superimposed, 6H, $C^{Ar}CH_3$), 1.65 (s, 3H, $C^{quart.}(CH_3)_2$), 1.63 (d, $^3J_{HH}$ = 6.5 Hz, 6H, NCH(CH_3)$_2$), 1.60 (s, 3H, BiCH_3), 1.54 (d, $^3J_{HH}$ = 6.5 Hz, 6H, NCH(CH_3)$_2$), 1.49 (s, 3H, $C^{quart.}(CH_3)_2$).

^{13}C{^{1}H} NMR (126 MHz) δ(ppm) = 157.39 (C^{Ar}), 145.41 (C^{Ar}), 141.34 (C^{Ar}), 138.12 (C^{Ar}), 122.99 (C^{Ar}H), 114.43 (C^{Ar}H), 52.09 (NCH(CH$_3$)$_2$), 46.67 (BiCH$_3$), 38.33 ($C^{quart.}(CH_3)_2$), 36.40 ($C^{quart.}(CH_3)_2$), 32.09 ($C^{quart.}(CH_3)_2$), 26.13 (NCH(CH$_3$)$_2$), 23.16 (NCH(CH$_3$)$_2$), 22.78 ($C^{Ar}CH_3$).

^{19}F{^{1}H} NMR (471 MHz) δ(ppm)= −78.62.

ATR-IR (solid): $\tilde{\nu}$ (cm^{-1}) = 2967 (w), 1395 (m), 1243 (s), 1030 (s), 632 (s).

UV/Vis: (toluene, 5 × 10^{-5} M, 25 °C): λ_{max} (nm) = 890, 447.

Elem. Anal. found (calcd) for ($C_{25}H_{33}BiF_3N_3O_3S$): C: 41.08 (41.61), H: 4.42 (4.61), N: 5.67 (5.82), S: 4.06 (4.44).

Synthesis of 3^{BArF}

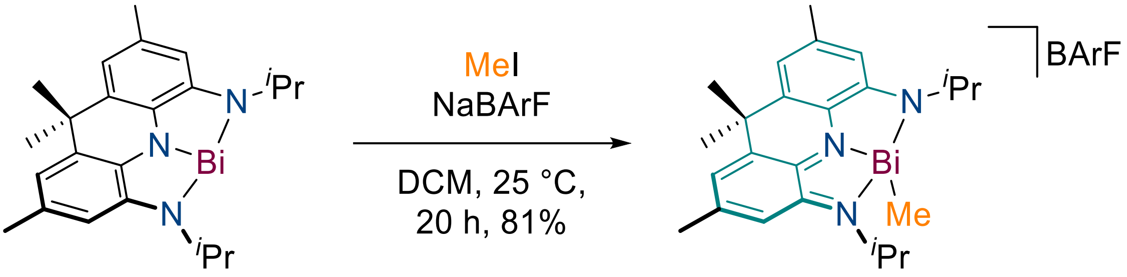

1^{Bi} 3^{BArF}

$\mathbf{1^{Bi}}$ (100 mg, 179 μmol, 1.00 eq.) and NaBArF (159 mg, 179 μmol, 1.00 eq.) are dissolved in DCM (15 mL) and iodomethane (MeI) (13 μL, 209 μmol, 1.17 eq.) is added while stirring. The dark blue reaction solution is stirred for 20 h at 25 °C, filtered and the residue is extracted with DCM (3 x 5 mL). All volatiles are removed *in vacuo*, followed by extraction with Et_2O (2 x 5 mL) and subsequent removal of the solvent *in vacuo*. The oily dark green solid is dissolved in DFB (3 mL), layered with hexane (15 mL) and crystallized at −30 °C. The supernatant is decanted and the dark green crystalline residue is washed with hexane (4 x 5 mL). Removing residual solvent *in vacuo* yields $\mathbf{3^{BArF}} \cdot (C_6H_4F_2)_{0.5}$ as dark green crystals (216.8 mg, 145 μmol, 81%).

See **Figure S33-Figure S39**, **Figure S40** and **Figure S41** for NMR, IR and UV/Vis spectra, respectively. For cyclic voltammogram see **Figure S129**.

NMR: (CD_2Cl_2, 25 °C) ^{1}H NMR (500 MHz) δ(ppm) = 7.74 – 7.70 (m, 8H, $C^{BArF}H$), 7.56 (s, 4H, $C^{BArF}H$), 6.79 (d, $^{4}J_{HH}$ = 1.1 Hz, 2H, $C^{Ar}H$), 6.62 (m, 2H, $C^{Ar}H$), 4.88 (hept, $^{3}J_{HH}$ = 6.5 Hz, 2H, $NCH(CH_3)_2$), 2.33 (s superimposed, 6H, $C^{Ar}CH_3$), 1.74 (s, 3H, $BiCH_3$), 1.66 (s, 3H, $C^{quart.}(CH_3)_2$), 1.57 (d, $^{3}J_{HH}$ = 6.5 Hz, 6H, $NCH(CH_3)_2$), 1.53 (s, 3H, $C^{quart.}(CH_3)_2$), 1.50 (d, $^{3}J_{HH}$ = 6.5 Hz, 6H, $NCH(CH_3)_2$).

$^{13}C\{^{1}H\}$ NMR (126 MHz) δ(ppm) = 162.16 ($^{1}J_{BC}$ = 49.9 Hz, BC^{BArF}), 157.10 (C^{Ar}), 147.06 (C^{Ar}), 141.23 (C^{Ar}), 137.55 (C^{Ar}), 135.21 (m, $C^{BArF}H$), 129.27 (qq, $^{2}J_{CF}$ = 31.6 Hz, $^{4}J_{CF}$ = 2.8 Hz, $C^{BArF}CF_3$), 125.01 (q, $^{1}J_{CF}$ = 272.4 Hz, $C^{BArF}CF_3$), 124.52 ($C^{Ar}H$), 117.88 (hept, $^{3}J_{CF}$ = 3.9 Hz $C^{BArF}(CF_3)_2$), 114.16 ($C^{Ar}H$), 52.37 ($NCH(CH_3)_2$), 43.53 ($BiCH_3$), 38.42 ($C^{quart.}(CH_3)_2$), 36.29 ($C^{quart.}(CH_3)_2$), 32.65 ($C^{quart.}(CH_3)_2$), 26.23 ($NCH(CH_3)_2$), 23.29 ($NCH(CH_3)_2$), 22.85 ($C^{Ar}CH_3$).

$^{19}F\{^{1}H\}$ NMR (471 MHz) δ(ppm) = −62.86.

^{11}B NMR (160 MHz) δ(ppm) = −6.65 (br.).

Elem. Anal. found (calcd) for ($C_{56}H_{45}BBiF_{24}N_3$): C: 47.10 (46.85), H: 2.94 (3.16), N: 2.81 (2.93).

ATR-IR (solid): $\tilde{\nu}$ (cm^{-1}) = 2970 (w), 1353 (s), 1269 (s), 1117 (s), 681 (m).

UV/Vis: (THF, 2 × 10^{-5} M, 25 °C): λ_{max} (nm) = 890, 447, 349.

Synthesis of 3^{Et_BArF}

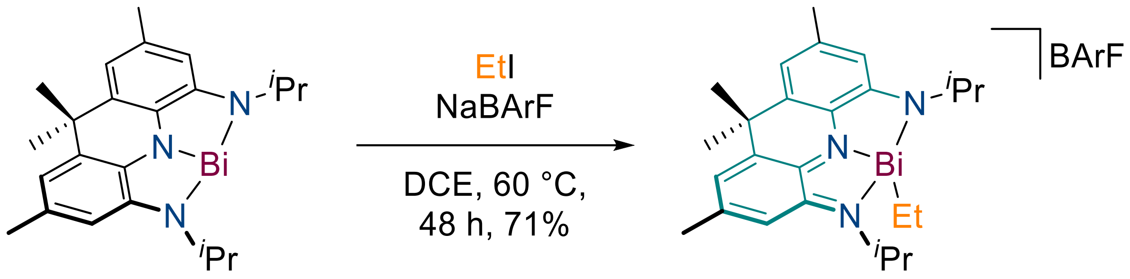

1^{Bi} 3^{Et_BArF}

1^{Bi} (65.3 mg, 117 µmol, 1.00 eq.) and NaBArF (103.8 mg, 117 µmol, 1.00 eq.) are dissolved in DCE (10 mL) and iodoethane (EtI) (10 µL, 124 µmol, 1.06 eq.) is added while stirring. The dark blue reaction solution is stirred for 48 h at 60 °C, volatiles are removed *in vacuo* and the residue is extracted with DCM (2 x 5 mL). After removing the solvent *in vacuo* the resulting violet oil is washed with hexane (3 x 15 mL) followed by extraction with Et_2O (2 x 5 mL) and subsequent removal of the solvent *in vacuo*. The violet oil is recrystallized out of DFB (3 mL), layered with hexane (15 mL) at −30 °C. The supernatant is decanted and the dark violet crystalline residue is washed with hexane (4 x 5 mL). Removing residual solvent *in vacuo* yields **3^{Et_BArF}** as dark violet crystals (120.9 mg, 83.4 µmol, 71%).

See **Figure S42-Figure S48** and

Figure S49 for NMR and IR spectra, respectively.

NMR: (CD_2Cl_2, 25 °C) ^1H NMR (600 MHz) δ(ppm) = 7.74 – 7.70 (m, 8H, $C^{BArF}H$), 7.56 (s, 4H, $C^{BArF}H$), 6.81 (d, $^4J_{HH}$ = 1.3 Hz, 2H, $C^{Ar}H$), 6.65 (t, $^4J_{HH}$ = 1.3 Hz, 2H, $C^{Ar}H$), 4.89 (hept, $^3J_{HH}$ = 6.4 Hz, 2H, $NCH(CH_3)_2$), 2.84 (t, $^3J_{HH}$ = 7.8 Hz, 3H, $BiCH_2CH_3$), 2.36 (q superimposed, $^3J_{HH}$ = 7.8 Hz, 2H), 2.34 (s superimposed, 6H, $C^{Ar}CH_3$), 1.69 (s, 3H, $C^{quart.}(CH_3)_2$), 1.59 (d, $^3J_{HH}$ = 6.5 Hz, 6H, $NCH(CH_3)_2$), 1.51 (d, $^3J_{HH}$ = 6.5 Hz, 6H, $NCH(CH_3)_2$), 1.45 (s, 3H, $C^{quart.}(CH_3)_2$).

$^{13}C\{^1H\}$ NMR (150 MHz) δ(ppm) = 162.18 ($^1J_{BC}$ = 49.9 Hz, BC^{BArF}), 157.34 (C^{Ar}), 147.04 (C^{Ar}), 141.41 (C^{Ar}), 137.24 (C^{Ar}), 135.23 (m, $C^{BArF}H$), 129.30 (qq, $^2J_{CF}$ = 31.6 Hz, $^4J_{CF}$ = 2.8 Hz, $C^{BArF}CF_3$), 125.03 (q, $^1J_{CF}$ = 272.5 Hz, $C^{BArF}CF_3$), 124.27 ($C^{Ar}H$), 117.90 (hept, $^3J_{CF}$ = 3.9 Hz $C^{BArF}(CF_3)_2$), 114.33 ($C^{Ar}H$), 57.51 ($BiCH_2CH_3$), 52.45 ($NCH(CH_3)_2$), 38.41 ($C^{quart.}(CH_3)_2$), 37.38 ($C^{quart.}(CH_3)_2$), 30.83 ($C^{quart.}(CH_3)_2$), 26.56 ($NCH(CH_3)_2$), 23.31 ($C^{quart.}(CH_3)_2$), 22.88 ($NCH(CH_3)_2$), 10.80 ($BiCH_2CH_3$).

$^{19}F\{^1H\}$ NMR (565 MHz) δ(ppm) = −62.88.

^{11}B NMR (160 MHz) δ(ppm) = −6.61 (br).

Elem. Anal. found (calcd) for ($C_{57}H_{47}BBiF_{24}N_3$ · DFB): C: 48.16 (48.39), H: 2.63 (3.29), N: 3.08 (2.69).

ATR-IR (solid): $\tilde{\nu}$ (cm^{-1}) = 2971 (w), 1611 (m), 1352 (s), 1270 (s), 1113 (s), 669 (s).

Generation of 4 *in situ*

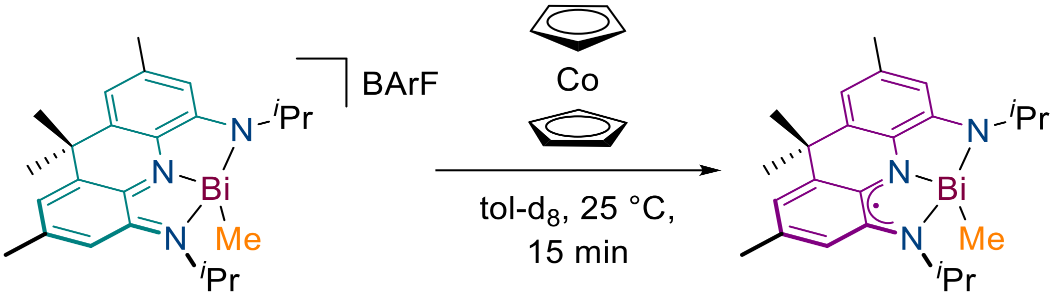

3BArF **4**

3BArF (11.7 mg, 8.15 µmol, 1.00 eq.) and cobaltocene (CoCp$_2$) (1.6 mg, 8.46 µmol, 1.00 eq.) are added to a J.-Young NMR tube, dissolved DCM-d$_2$ (0.6 mL) and shaken for 5 minutes and monitored by NMR spectroscopy (see **Figure S50-Figure S51**). The ^{1}H NMR spectra show gradual formation of **1**Bi over several days at 25 °C

For follow up reactivity: **3**BArF (33.4 mg, 23.2 µmol, 1.00 eq.) is added to a scintillation vial along with a stirring bar and dissolved in toluene-d$_8$ (0.8 mL). CoCp$_2$ (4.4 mg, 23.2 µmol, 1.00 eq.) is added and stirred for 15 min at 25 °C followed by filtration into a J-Young NMR tube after stirring for 15 min. This protocol was also utilized to prepare the EPR sample.

For spectro-electrochemistry see **Figure S131**.

UV/Vis (THF, 25 °C, SEC): 431 nm.

Synthesis of 5

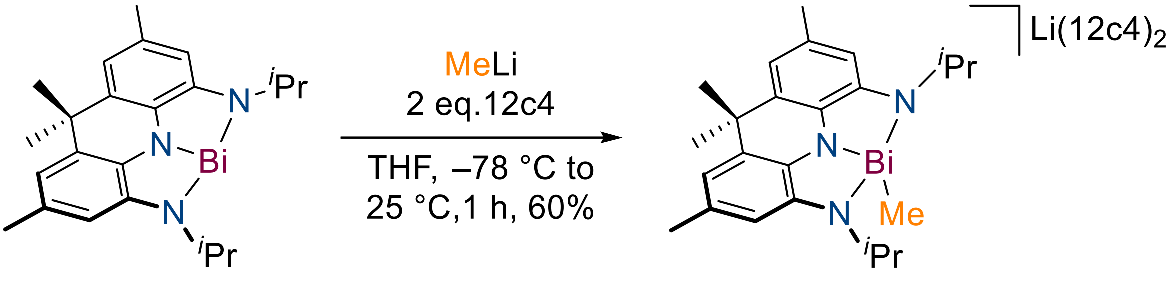

$\mathbf{1^{Bi}}$ **5**

$\mathbf{1^{Bi}}$ (72.0 mg, 129 µmol, 1.00 eq.) and 1,4,7,10-tetraoxacyclododecane (12c4) (41.8 µL, 258 µmol, 2.00 eq.) are dissolved in THF (15 mL) inside a J.-Young Schlenk flask and cooled to −78 °C. Methyllithium (MeLi) (82.0 µL, 1.6 M in Et_2O, 1.02 eq.) is added dropwise and the reaction solution is stirred for 15 min followed by warming to room temperature and stirring for an additional 1 h at 25 °C. After removing the solvent *in vacuo*, the reaction vessel is transferred into a glove box and the red residue is extracted with THF (2 mL) into a scintillation vial and layered with hexane (3 mL). Crystallization for 3 d at −30 °C leads to the formation of dark red crystals. The supernatant is decanted and the red crystalline residue is washed with hexane (4 x 3 mL). Removing residual solvent *in vacuo* yields **5** as bright red crystals (72.3 mg, 77.6 µmol, 60%). *Co-crystallized THF can be removed by drying in vacuo leading to the formation of a dark red powder.* ***5*** *is highly reactive and immediately decomposes when exposed to trace amounts of oxygen or moisture.*

See **Figure S52-Figure S57** and **Figure S58** for NMR and IR spectra, respectively. For spectro-electrochemistry see **Figure S132** and for the cyclic voltammogram see **Figure S133**.

NMR: (THF-d_8, 25 °C) ^{1}H NMR (300 MHz) δ(ppm) = 5.92 (s, 2H, $C^{Ar}H$), 5.61 (s, 2H, $C^{Ar}H$), 4.09 (hept, $^3J_{HH}$ = 6.4 Hz, 2H, NC*H*($CH_3)_2$), 3.46 (s, 32H, 12c4), 2.13 (s, 6H, $C^{Ar}CH_3$), 1.55 (s, 3H, $C^{quart.}(CH_3)_2$), 1.27 (s superimposed, $^3J_{HH}$ = 6.4 Hz, 12H, NCH($CH_3)_2$), 1.17 (br. s, 3H, $C^{quart.}(CH_3)_2$), 0.92 (s, 3H, BiCH_3).

^{13}C{^{1}H} NMR (75 MHz) δ(ppm) = 148.54 (C^{Ar}), 136.00 (C^{Ar}), 127.12 (C^{Ar}), 126.08 (C^{Ar}), 107.11 (C^{Ar}H) 105.65 (C^{Ar}H), 70.12 (12c4), 68.39 (THF), 49.83 (N*C*H($CH_3)_2$), 45.51 (BiCH_3), 36.61 ($C^{quart.}(CH_3)_2$), 34.69 ($C^{quart.}(CH_3)_2$), 28.79 ($C^{quart.}(CH_3)_2$), 26.55 (THF), 25.87 (NCH($CH_3)_2$), 22.67 ($C^{Ar}CH_3$).

^{7}Li{^{1}H} NMR (194 MHz) δ(ppm) = −0.97.

ATR-IR (solid): $\tilde{\nu}$ (cm^{-1}) = 2954 (m), 2907 (m), 2858 (m), 1131 (s), 1092 (s), 1022 (s).

UV/Vis (THF, 25 °C, SEC): 391 nm.

Elem. Anal. found (calcd) for ($C_{40}H_{65}BiLiN_3O_8$): C: 51.78 (51.56), H: 7.34 (7.03), N: 4.29 (4.51).

Elem. Anal. found (calcd) for ($C_{40}H_{65}BiLiN_3O_8 \cdot$ THF): C: 52.28 (52.64), H: 7.33 (7.33), N: 4.21 (4.19).

Synthesis of 6

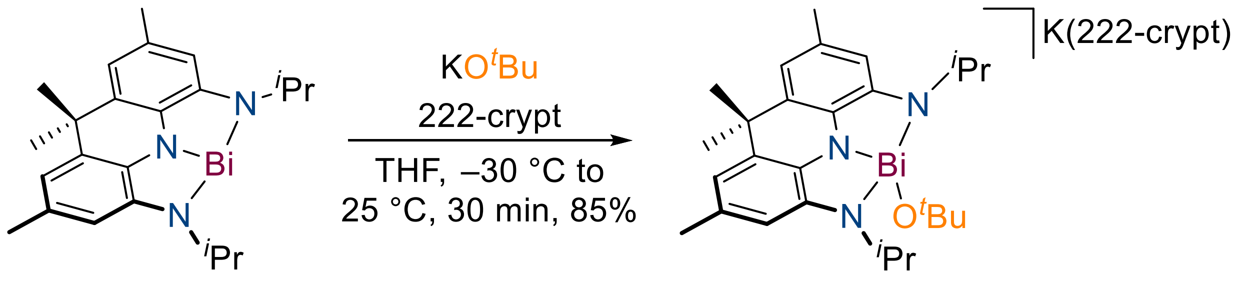

1^{Bi} **6**

1^{Bi} (93.4 mg, 168 µmol, 1.00 eq.) and 4,7,13,16,21,24-hexaoxa-1,10-diazabicyclo[8.8.8]hexacosane (222-crypt) (63.3 mg, 168 µmol, 1.00 eq.) are dissolved in THF (4 mL) inside a scintillation vial and cooled to −30 °C. Potassium tert-butoxide (18.8 mg, 168 µmol, 1.00 eq.) is added in small portions while stirring inside the glovebox. The reaction solution is stirred for 30 min while slowly warming up to 25 °C. The red reaction solution is filtered into a new scintillation vial and layered with hexane (15 mL). Crystallization for 7 d at −30 °C leads to the formation of dark red crystals. The supernatant is decanted and the red crystalline residue washed with hexane (3 x 5 mL). Removing residual solvent *in vacuo* yields **6** as bright red crystals (149 mg, 142 µmol, 85%).

See **Figure S59**-**Figure S64**, **Figure S65** and **Figure S66** for NMR, IR and UV/Vis spectra, respectively.

NMR: (THF-d_8, 25 °C) ^{1}H NMR (400 MHz) δ(ppm) = 6.03 (s, 2H, C^{Ar}*H*), 5.80 (s, 2H, C^{Ar}*H*), 4.45 (hept, $^3J_{HH}$ = 6.3 Hz, 2H, NC*H*(CH_3)$_2$), 3.33 (s, 12H, 222-crypt), 3.32 – 3.20 (m, 12H, 222-crypt), 2.35 – 2.30 (m, 12H, 222-crypt), 2.22 (s, 6H, C^{Ar}C*H*$_3$), 1.59 (s, 6H, C$^{quart.}$(C*H*$_3$)$_2$), 1.45 (s, 12H, NCH(C*H*$_3$)$_2$), 0.61 (s, 9H, BiOC(C*H*$_3$)$_3$).

^{13}C{^{1}H} NMR (101 MHz) δ(ppm) = 149.81 (*C*^{Ar}), 138.39 (*C*^{Ar}), 130.19 (*C*^{Ar}), 125.94 (*C*^{Ar}), 109.24 (*C*^{Ar}H), 106.81(*C*^{Ar}H), 71.28 (222-crypt), 68.48 (BiO*C*(CH_3)$_3$), 54.82 (222-crypt), 51.00 (222-crypt), 36.87 (*C*$^{quart.}$(CH_3)$_2$), 35.02 (BiOC(*C*H$_3$)$_3$), 22.69 (C^{Ar}*C*H$_3$).

ATR-IR (solid): $\tilde{\nu}$ (cm^{-1}) = 2952 (m), 2880 (m), 2812 (m), 1577 (s), 1100 (s), 930 (s).

Elem. Anal. found (calcd) for ($C_{45}H_{75}BiKN_5O_7$): C: 50.86 (51.66), H: 6.92 (7.23), N: 6.39 (6.69).

UV/Vis (THF, 25 °C): 368 nm.

Me^{+}, $Me^{•}$, and Me^{-} Transfer

Transfer of Me^{+} from 3^{BArF} to $P(^tBu_3)_3$

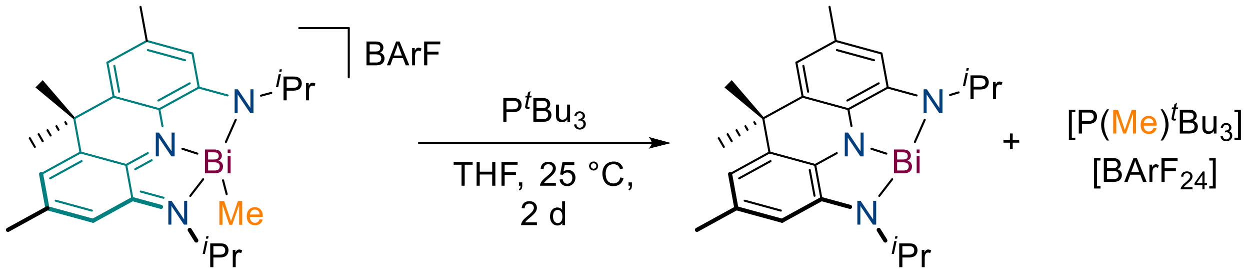

3^{BArF} 1^{Bi}

3^{BArF} (7.4 mg, 4.96 µmol, 1.00 eq.) and P^tBu_3 (1.0 mg, 4.96 µmol, 1.00 eq.) are dissolved in THF and added to a J.-Young NMR tube. The reaction mixture is stirred at 25 °C and monitored *via* NMR spectroscopy. The ^{31}P NMR spectra show the gradual formation of $P(Me)^tBu_3$. After two days no further reactivity is observed. The solvent is removed and the blue solid is re-dissolved in DCM-d_2 and hexamethylbenzene (0.8 mg, 4.96 µmol, 1.00 eq.) as an internal standard is added. The ^1H NMR spectrum reveals the quantitative transfer of the methyl group to P^tBu_3 (see **Figure S67-Figure S69.**).

Transfer of $Me^{•}$ from 4 to TEMPO

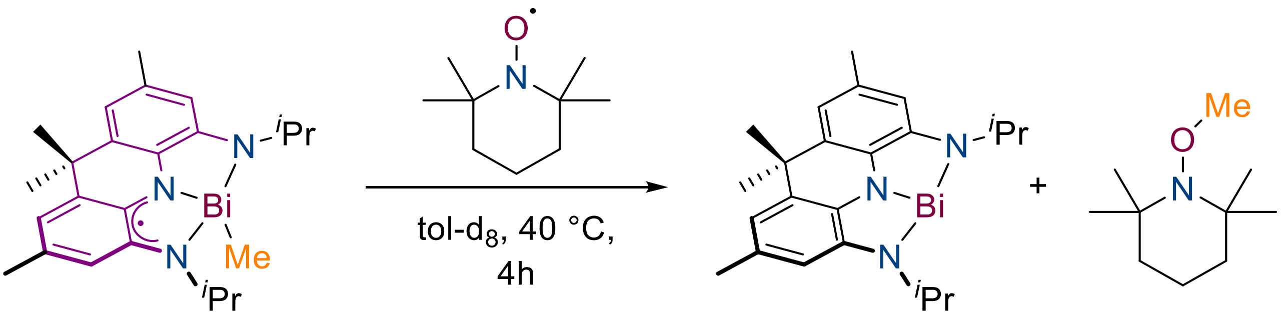

4 1^{Bi}

A solution of freshly prepared **4** (33.4 mg, 23.3 µmol, 1.00 eq.) in tol-d_8 (0.7 mL) is added to a J.-Young NMR tube containing TEMPO (3.9 mg, 25.0 µmol, 1.07 eq.) and hexamethylbenzene (3.7 mg, 23.4 µmol, 1.01 eq.) as an internal standard. The reaction mixture is heated to 40 °C and monitored by NMR spectroscopy (see **Figure S71-Figure S72**). After 4 h, $\mathbf{1^{Bi}}$ (65%) is regenerated accompanied by the formation of TEMPOMe (42%).

Transfer of Me⁻ from 5 to $B(C_6F_5)_3$

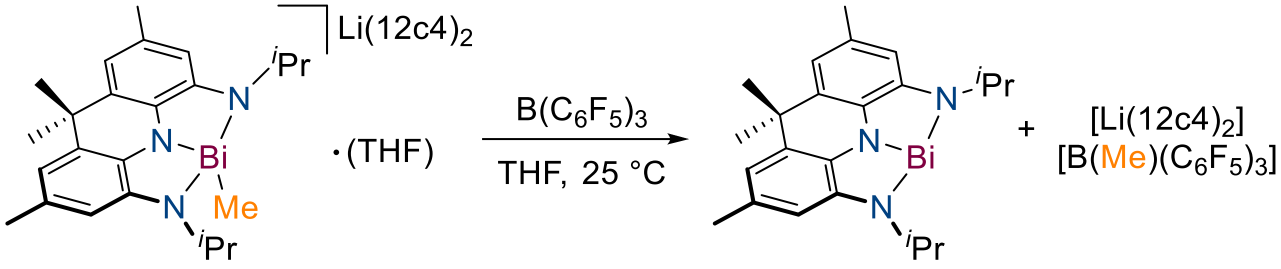

5 **1^Bi**

Tris(pentafluorophenyl)borane (BCF) (16.8 mg, 32.8 μmol, 1.00 eq.) is dissolved in THF-d_8 (0.7 mL) and added to a scintillation vial containing freshly prepared **5**·(THF) (32.9 mg, 32.8 μmol, 1.00 eq.). An immediate color change from red to blue is observed. The reaction solution is added to a J.-Young NMR tube containing hexamethylbenzene (HMB) (5.3 mg, 32.8 μmol, 1.00 eq.). The reaction mixture is stirred at 25 °C for 4 h and monitored by NMR spectroscopy revealing the formation of $\mathbf{1^{Bi}}$ (46%) accompanied by $[Li(12c4)_2]$ $[B(Me)(C_6F_5)_3]$ (49%) (see **Figure S75-Figure S78**).

Additional Reactions

Reaction of $\mathbf{1^{Bi}}$ with Water

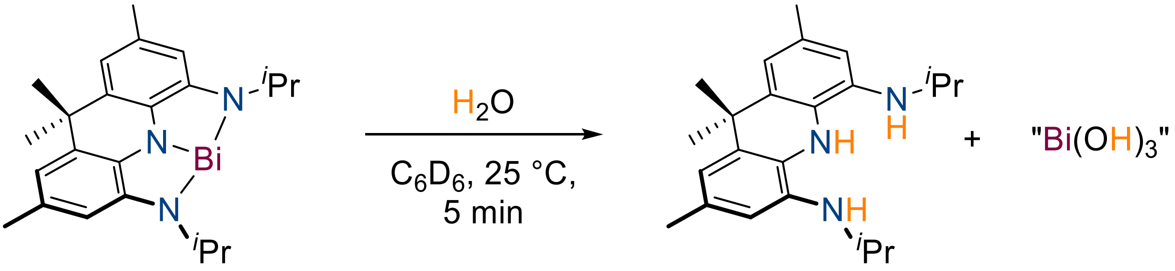

$\mathbf{1^{Bi}}$ **$\mathbf{H_3NNN}$**

$\mathbf{1^{Bi}}$ (22.4 mg, 40.2 μmol, 1.00 eq.) is added to a J.-Young NMR tube and dissolved in C_6D_6. Distilled and degassed water (3.6 μL, 199.4 μmol, 4.96 eq.) is added to the tube. The reaction mixture is stirred at 25 °C for 5 min. An immediate decolorization of the reaction solution as well as the formation of a white precipitant is visible. The ^1H NMR spectrum reveals the quantitative formation of the protioligand **$\mathbf{H_3NNN}$** (see **Figure S79-Figure S80**).

Reaction of 1^{Bi} with Diphenyl diselenide

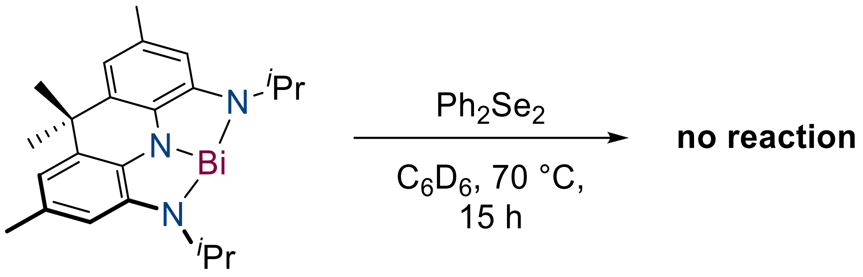

1^{Bi}

1^{Bi} (17.5 mg, 31.4 µmol, 1.00 eq.) and diphenyl diselenide (Ph_2Se_2) (9.8 mg, 31.4 µmol, 1.00 eq.) are added to a J.-Young NMR tube and dissolved in C_6D_6. The reaction mixture is stirred at 25 °C for 5 h and monitored *via* NMR spectroscopy. No conversion of the substrate could be observed in the ^{1}H NMR even after heating to 70 °C for 15 h (see **Figure S81-Figure S82**).

Reaction of 3^{BArF} with KHMDS

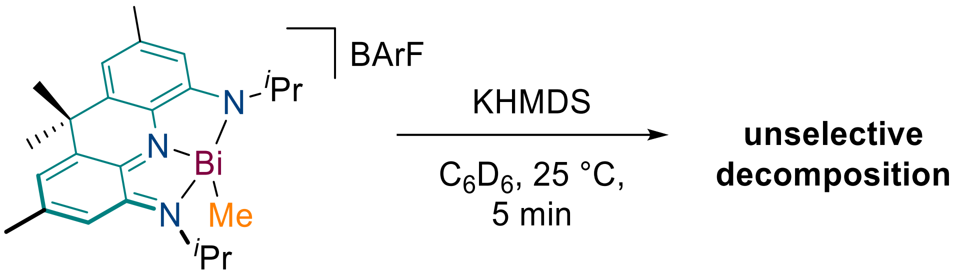

3^{BArF}

3^{BArF} (10.5 mg, 7.3 µmol, 1.00 eq.) and potassium bis(trimethylsilyl)amide (KHMDS) (2.0 mg, 10 µmol, 1.37 eq.) are added to a J.-Young NMR tube and dissolved in C_6D_6. An immediate color change to yellow/orange is observed. Investigation *via* NMR spectroscopy reveals the formation of HMDS (see **Figure S83**).

Due to the poor solubility of **3^{BArF}** in benzene the reaction was reattempted in a different solvent. For this KHMDS (3.3 mg, 16.6 µmol, 1.00 eq.) is added to a scintillation vial inside the glovebox, dissolved in Et_2O (2mL) and cooled to –30 °C for 15 min. While stirring **3^{BArF}** (24 mg, 16.7 µmol, 1.00 eq.) is added at 25 °C. A rapid color change from green to purple and finally to orange was observed over the course of 5 min. The solvent is removed *in vacuo* and the green oily substance is re-dissolved in THF-d_8. The ^{1}H NMR spectrum reveals only signals for the BArF anion as well as residual HMDS (**Figure S84**).

NMR, IR and UV/Vis Spectroscopy

Determination of Lewis Acidities *via* the Gutmann-Beckett Method

The Lewis acidity of **1**^{Bi} and **1**^{P} was assessed *via* addition of one equivalent of Et_3PO in a benzene solution of the respective complexes including a capillary of PPh_3 as an internal standard.[11] The acceptor number (AN) for **1**^{Bi} and **2** was assessed *via* addition of one equivalent of Me_3PS in a DCM solution of the respective complexes including a capillary of PPh_3 as an internal standard.[12] The acceptor number was derived *via* application of the following formula:

$$AN(Et_3PO) = 2.21 \cdot (\delta(^{31}P\ NMR)_{Sample}/ppm - 41.0)$$

Acceptor numbers: **1**^{Bi} = 10.8; **1**^{P} = 11.8

$$AN(Me_3PS) = 6.41 \cdot (\delta(^{31}P\ NMR)_{Sample}/ppm - 29.2)$$

Acceptor numbers: **1**^{Bi} = 6.8; **2** = 8.1

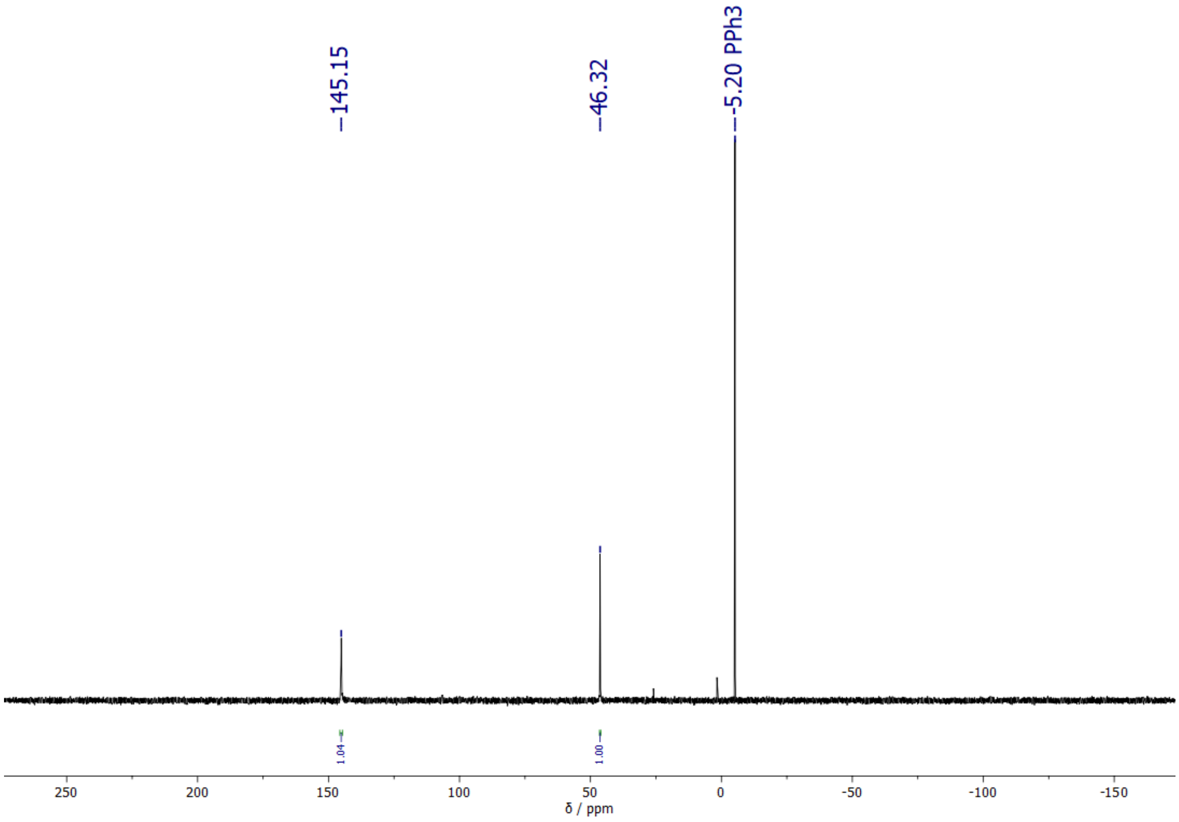

Figure S1. $^{31}P\{^{1}H\}$ NMR spectrum of **1**^{P} in the presence of one equivalent of Et_3PO, benzene, 25 °C.

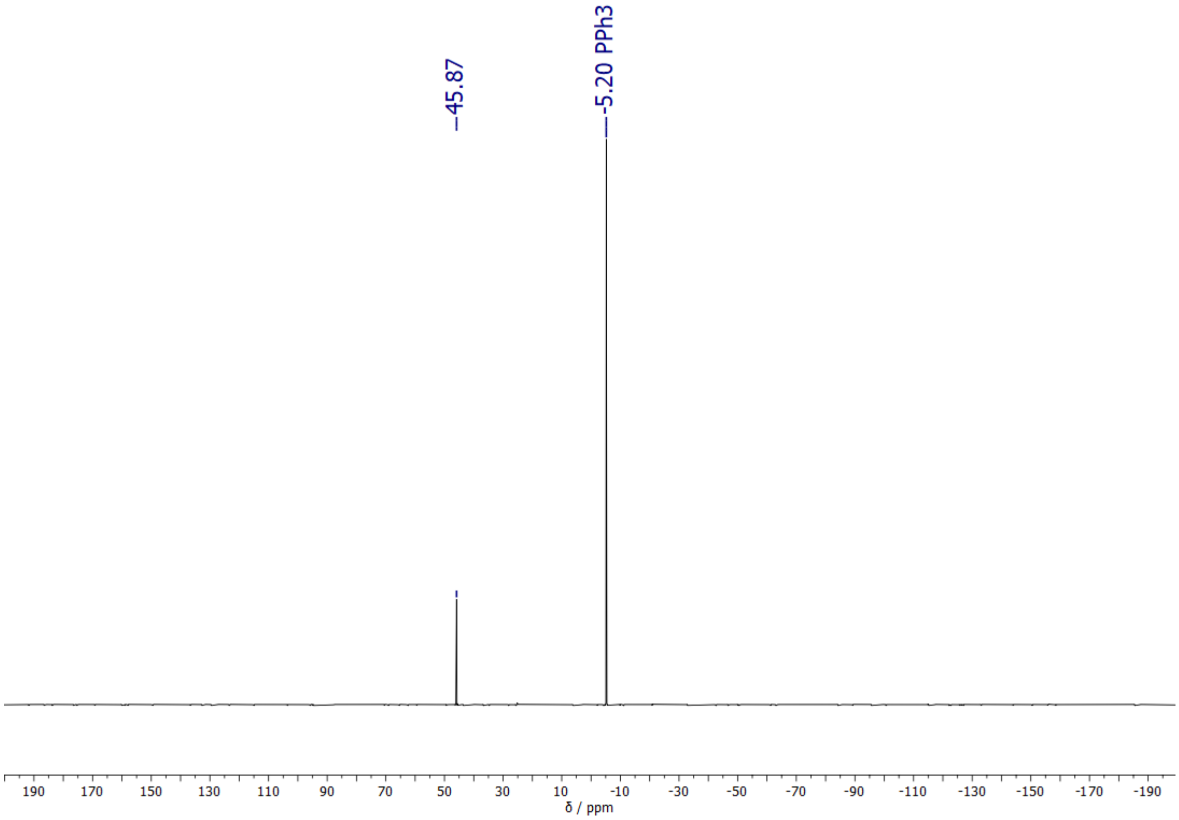

Figure S2. $^{31}P\{^{1}H\}$ NMR spectrum of $\mathbf{1}^{Bi}$ in the presence of one equivalent of Et_3PO, benzene, 25 °C.

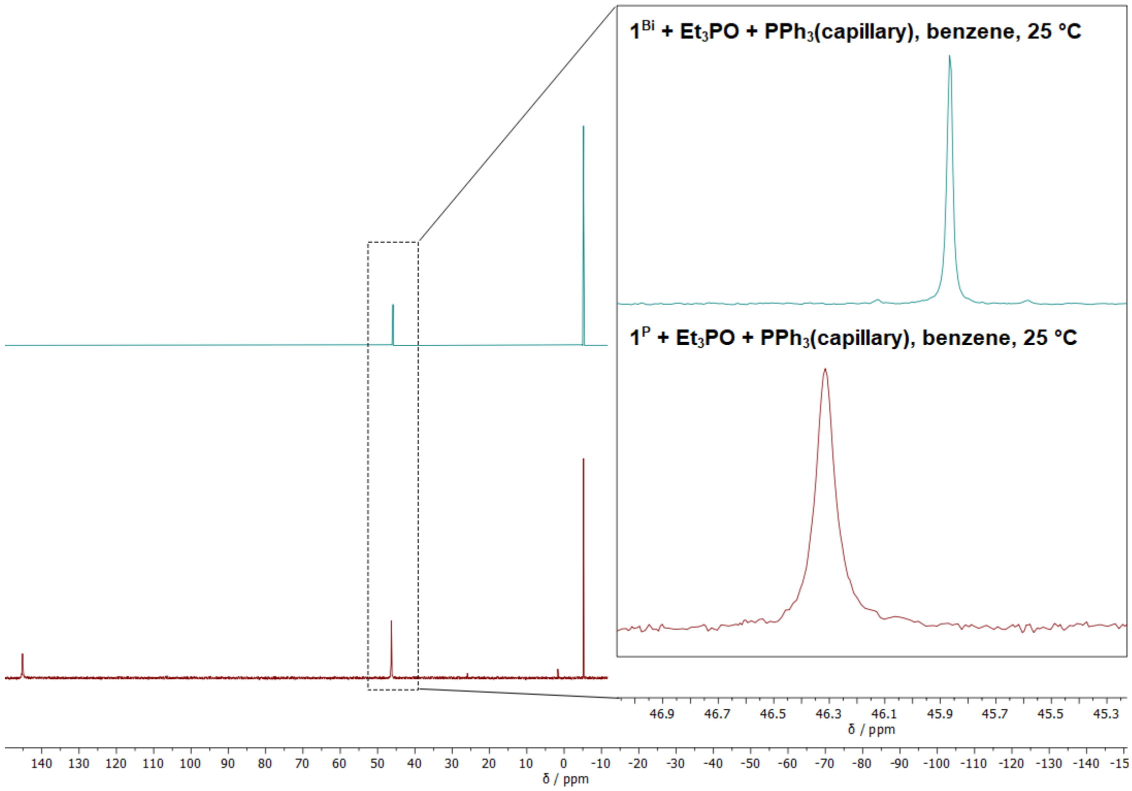

Figure S3. $^{31}P\{^{1}H\}$ NMR spectrum of $\mathbf{1}^{Bi}$ and $\mathbf{1}^{P}$ in the presence of one equivalent of Me_3PS, DCM, 25 °C. inset: magnification of the Et_3PO signal.

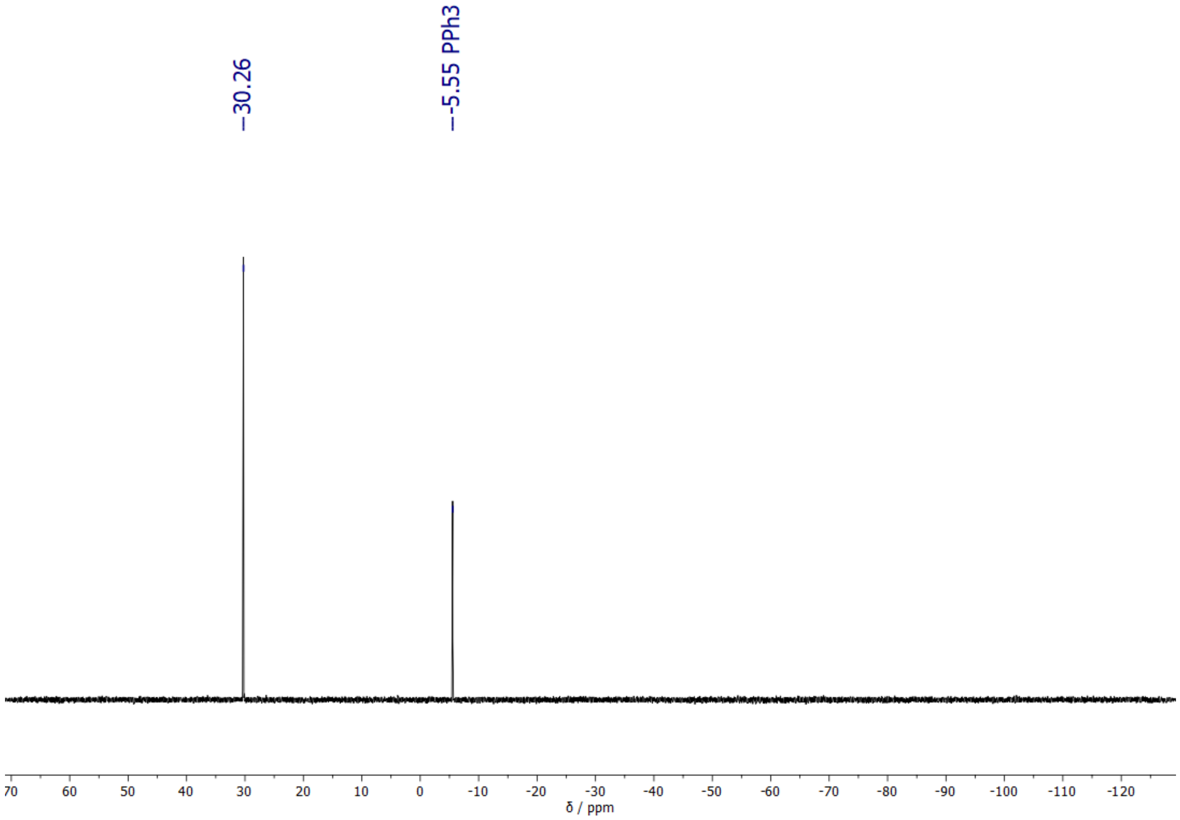

Figure S4. $^{31}P\{^{1}H\}$ NMR spectrum of **1^{Bi}** in the presence of one equivalent of Me_3PS, DCM, 25 °C.

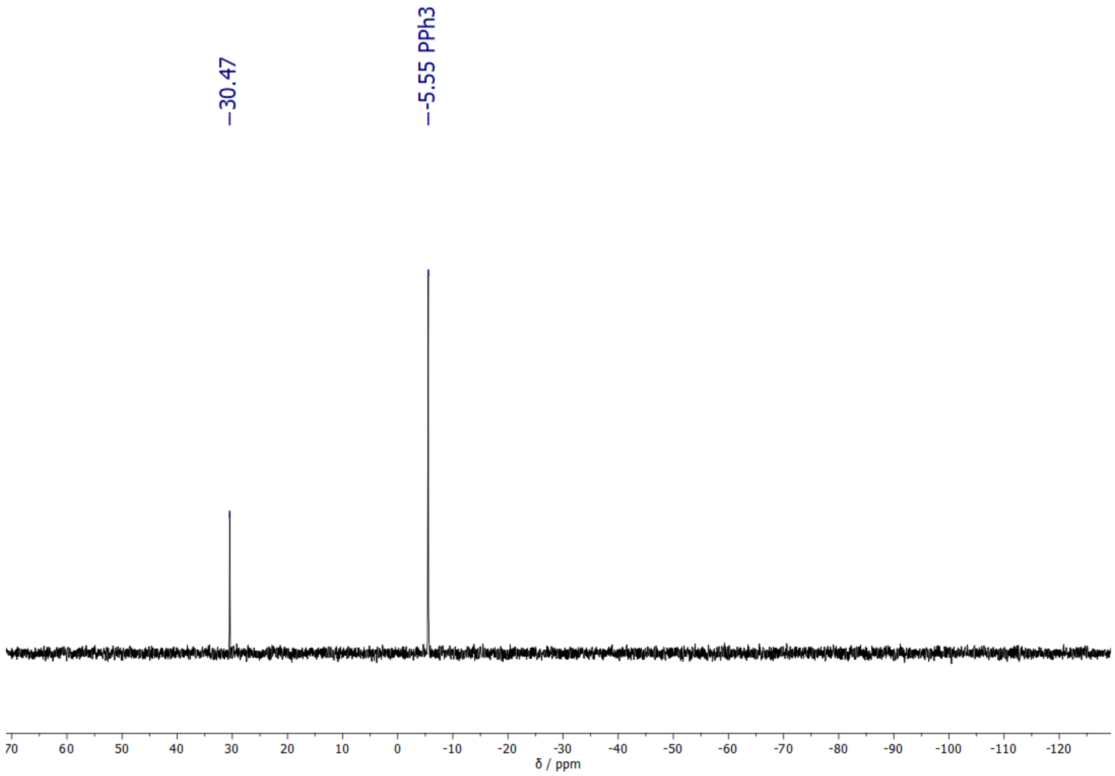

Figure S5. $^{31}P\{^{1}H\}$ NMR spectrum of **2** in the presence of one equivalent of Me_3PS, DCM, 25 °C.

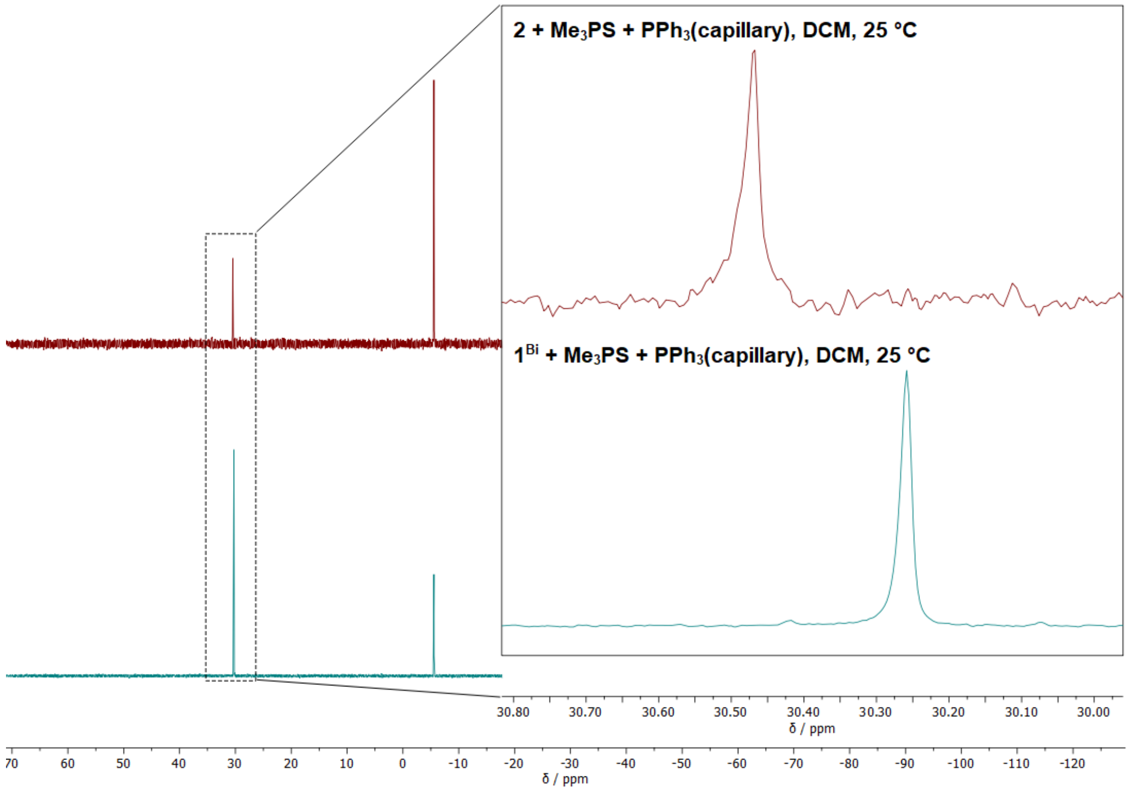

Figure S6. $^{31}P\{^{1}H\}$ NMR spectrum of $\mathbf{1}^{Bi}$ and **2** in the presence of one equivalent of Me_3PS, DCM, 25 °C. inset: magnification of the Me_3PS signal.

Spectroscopy of $\mathbf{1}^{P}$

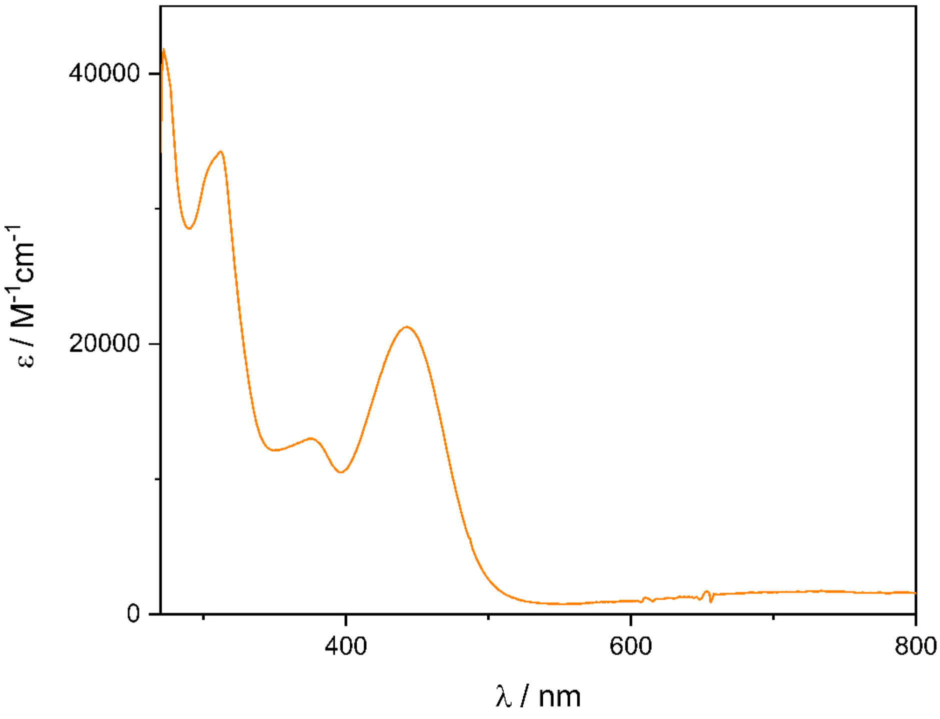

Figure S7. UV/Vis spectrum of $\mathbf{1}^{P}$, hexane, 25 °C.

Spectroscopy of 1^{Bi}

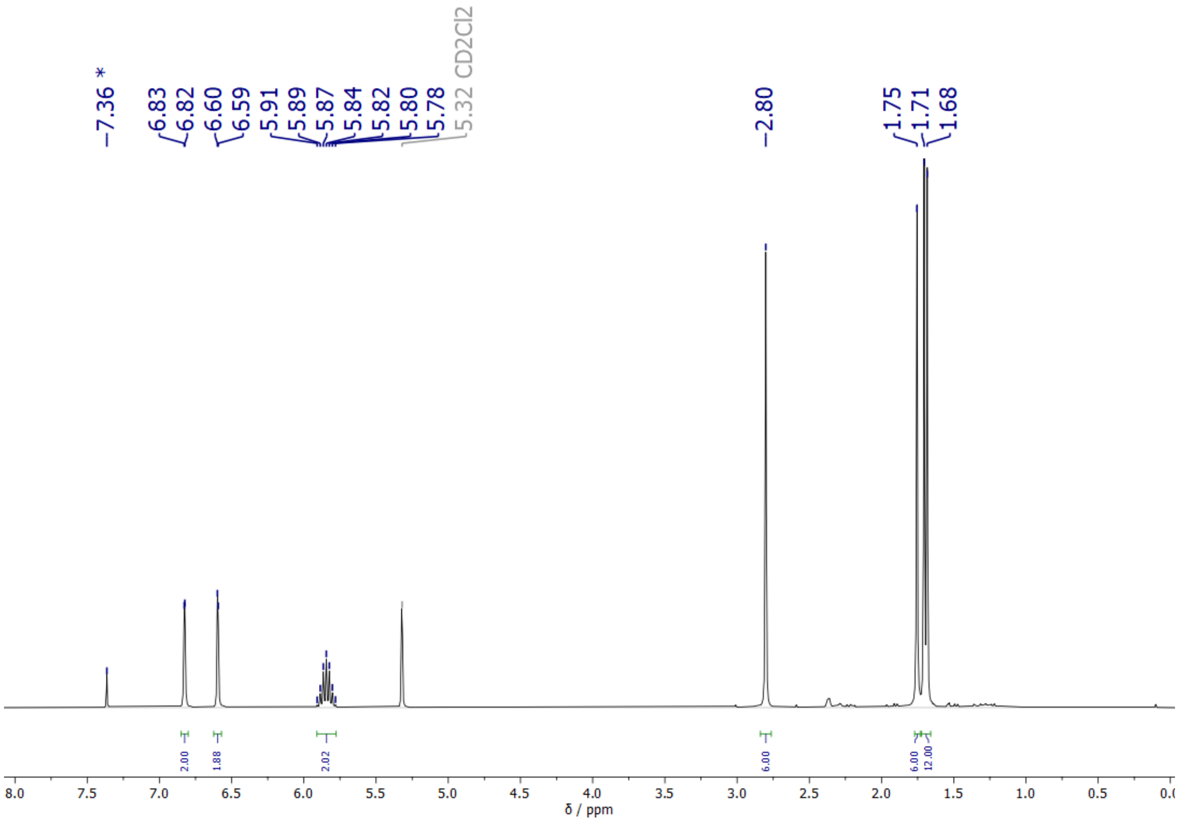

Figure S8. ^{1}H NMR spectrum of 1^{Bi}, CD_2Cl_2, 25 °C; benzene marked with *.

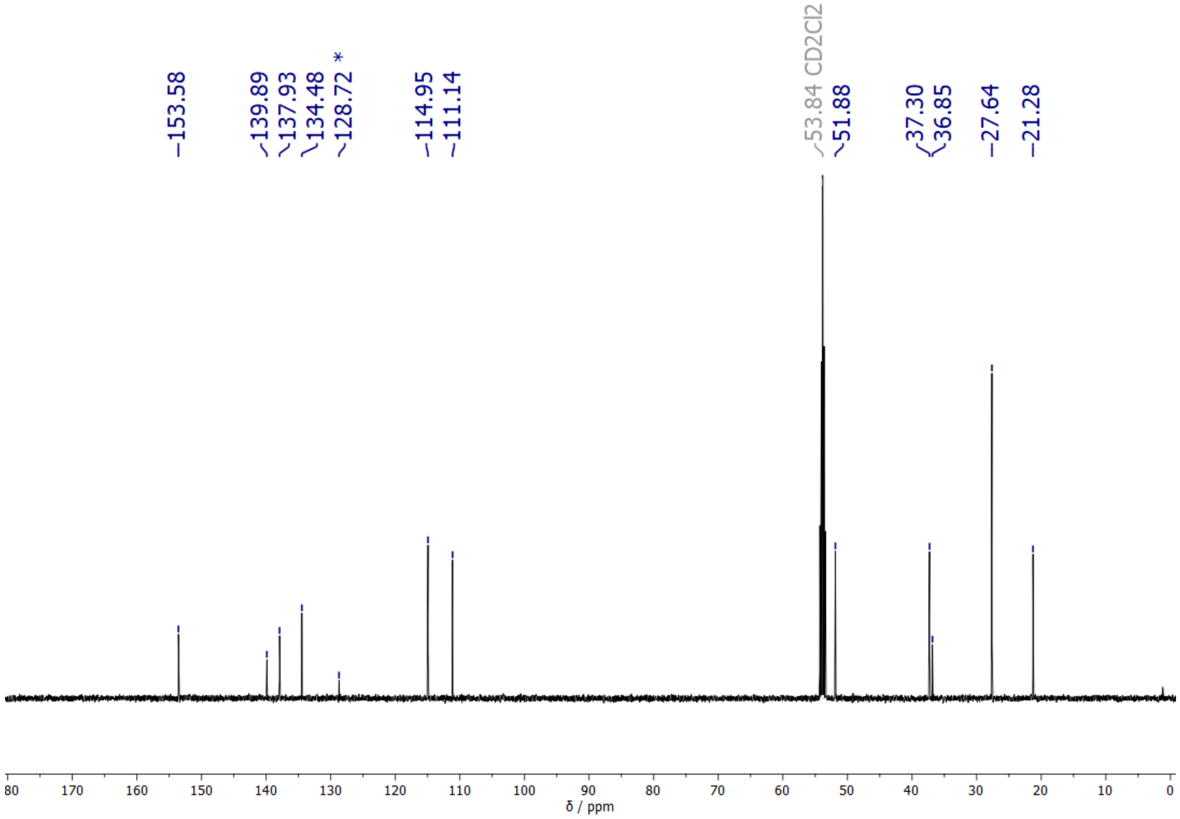

Figure S9. $^{13}C\{^{1}H\}$ NMR spectrum of 1^{Bi}, CD_2Cl_2, 25 °C; benzene marked with *.

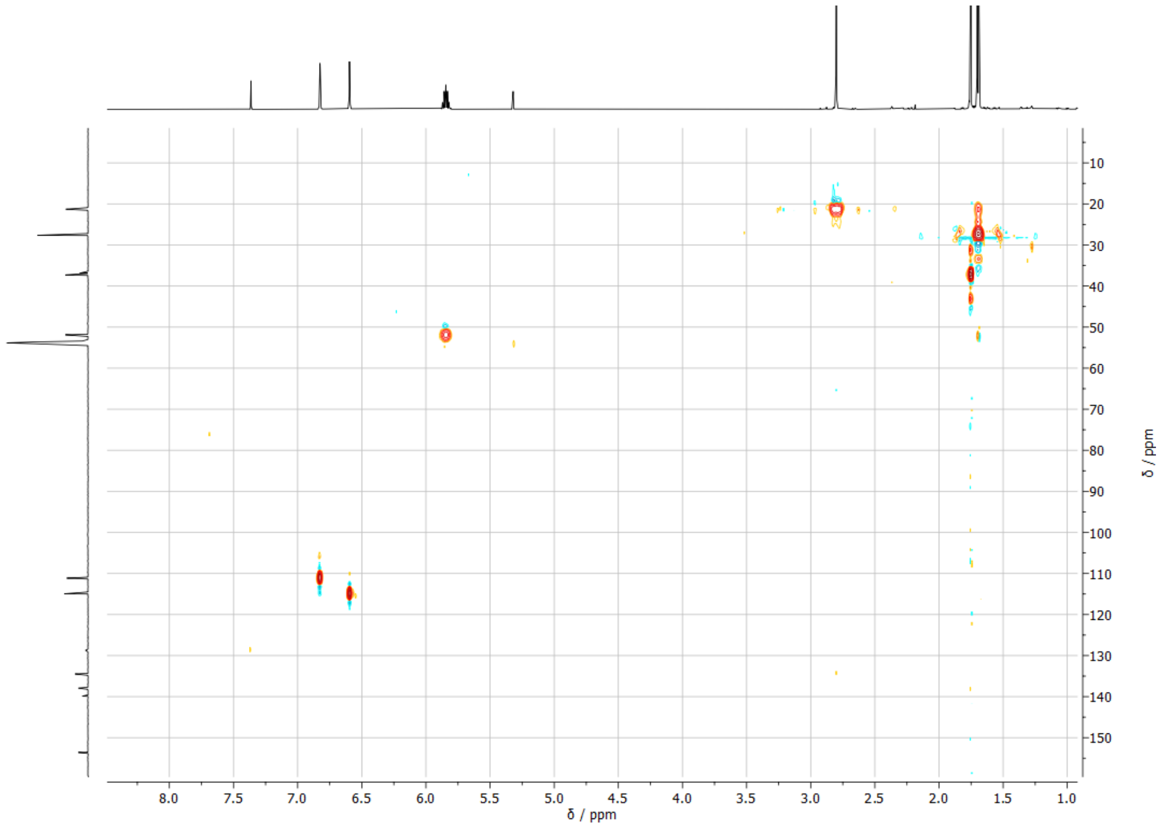

Figure S10. HSQC NMR spectrum of **1**[Bi], CD_2Cl_2, 25 °C.

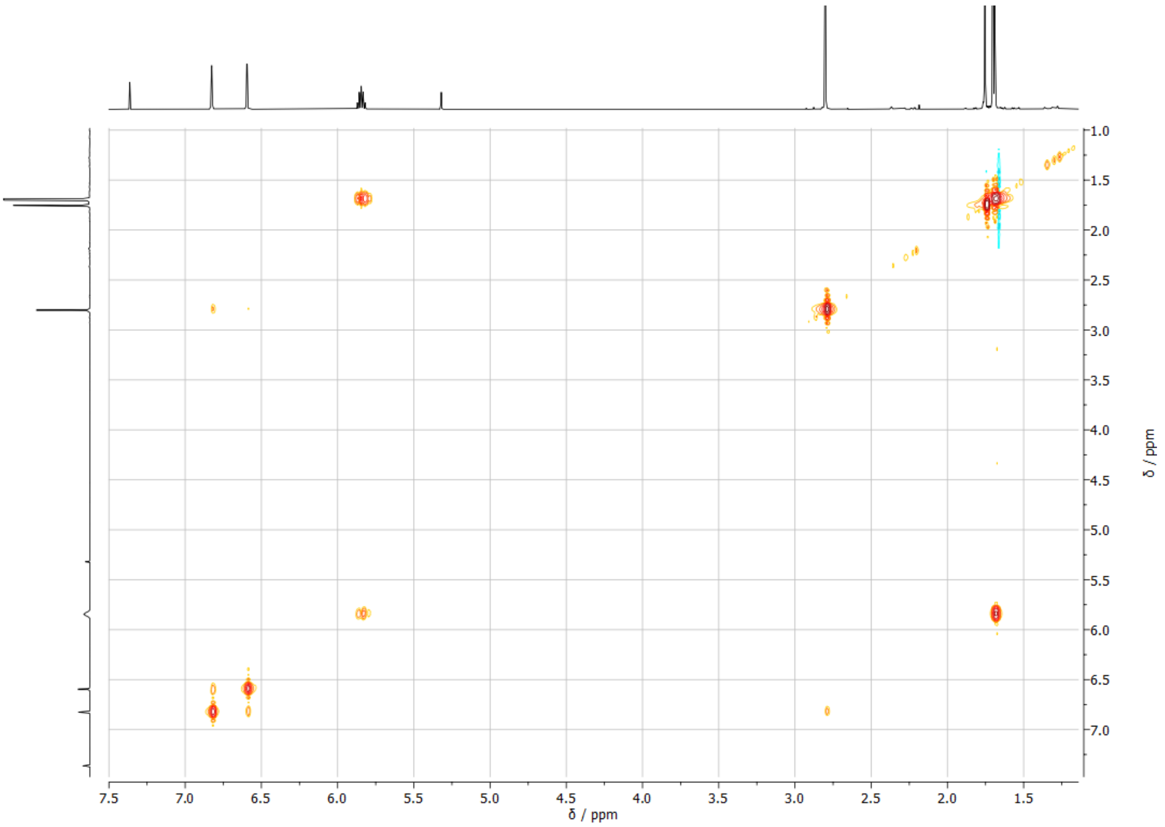

Figure S11. COSY NMR spectrum of **1**[Bi], CD_2Cl_2, 25 °C.

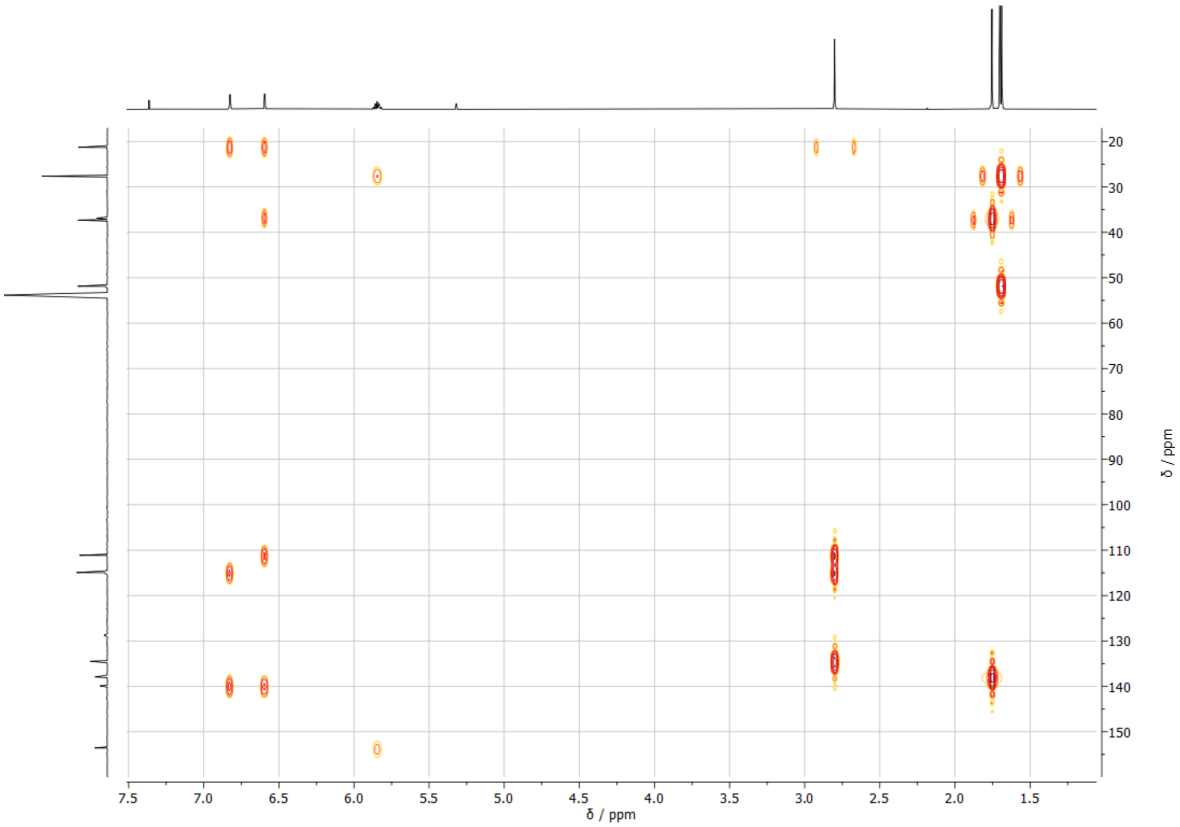

Figure S12. HMBC NMR spectrum of **1**^{Bi}, CD_2Cl_2, 25 °C.

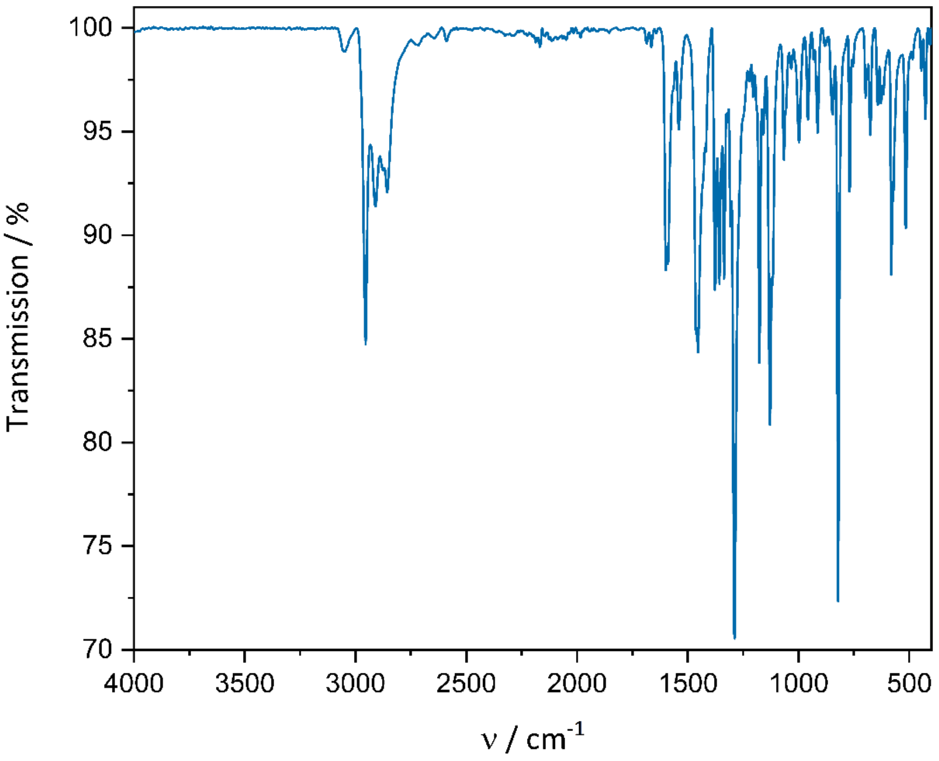

Figure S13. ATR-IR spectrum of **1**^{Bi}, solid, 25 °C.

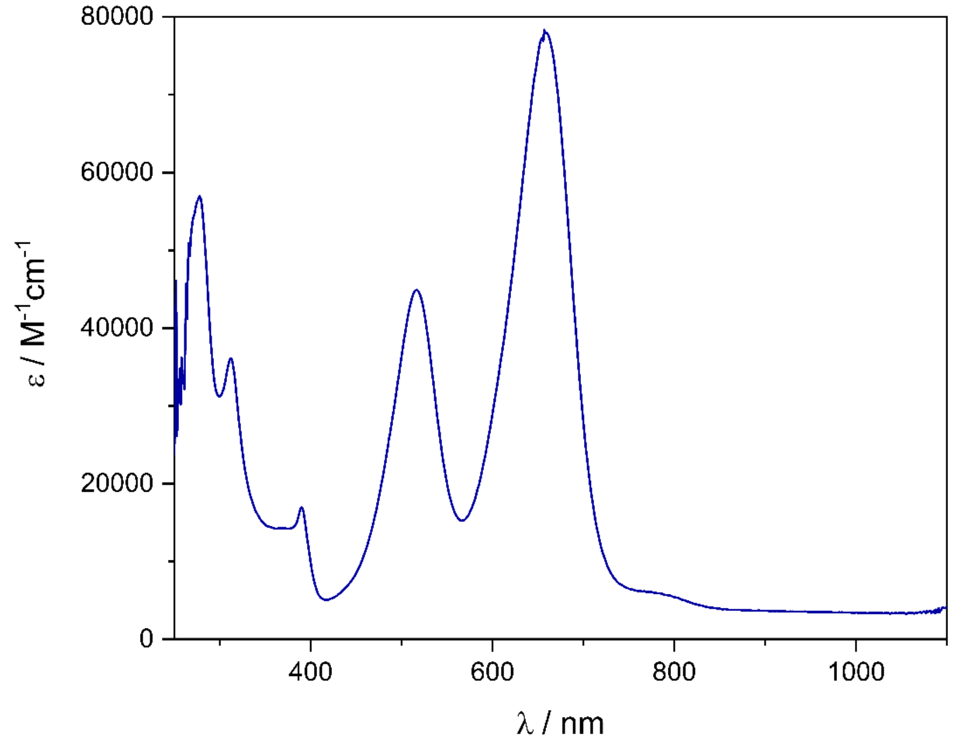

Figure S14. UV/Vis spectrum of **1**^{Bi}, hexane, 25 °C.

Spectroscopy of 2

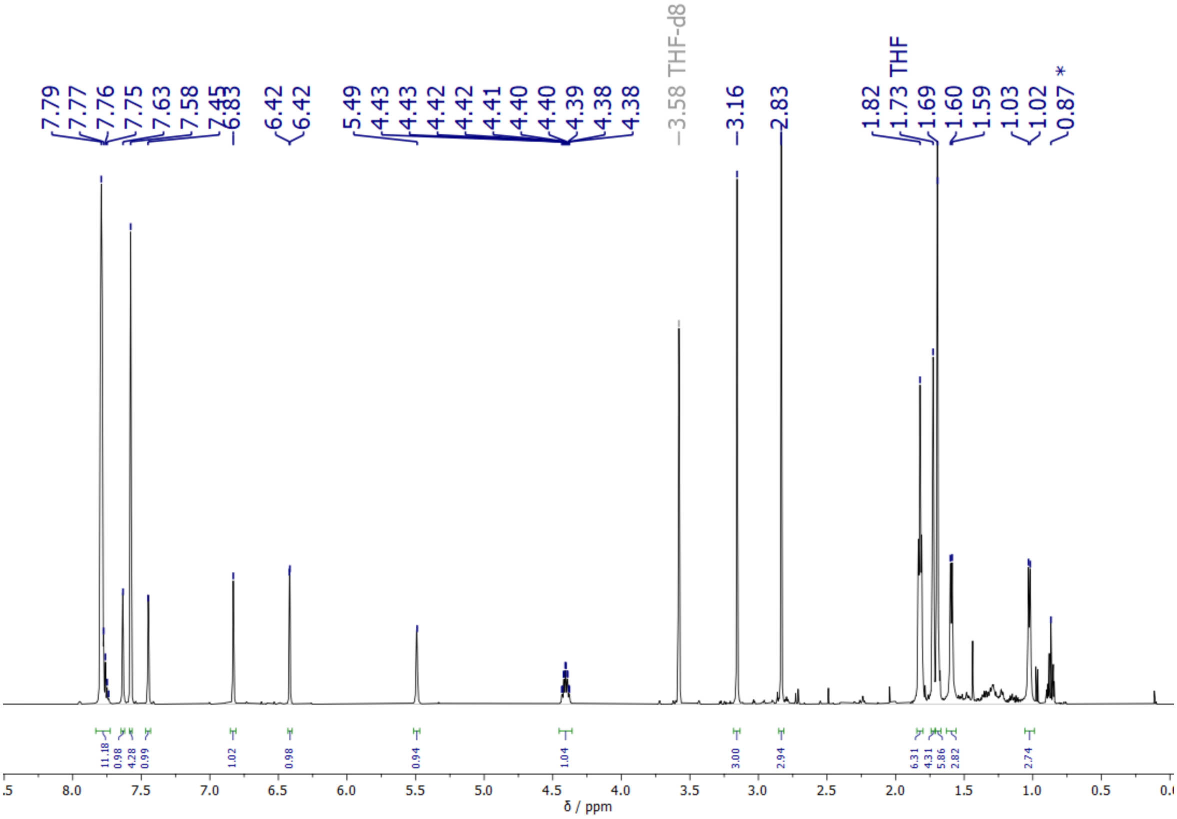

Figure S15. ^1H NMR spectrum of **2**, THF-d_8, 25 °C; H grease marked with *.

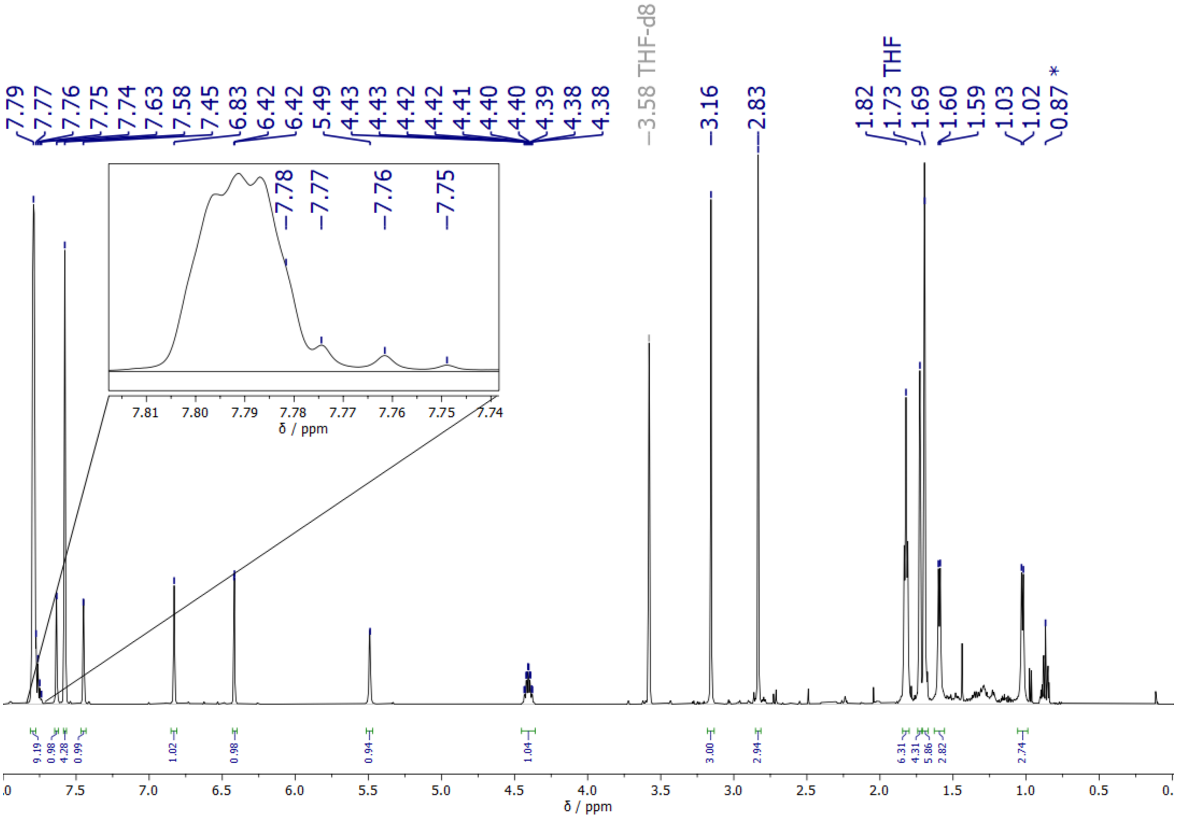

Figure S16. ^1H NMR spectrum of **2**, THF-d_8, 25 °C; H grease marked with *.

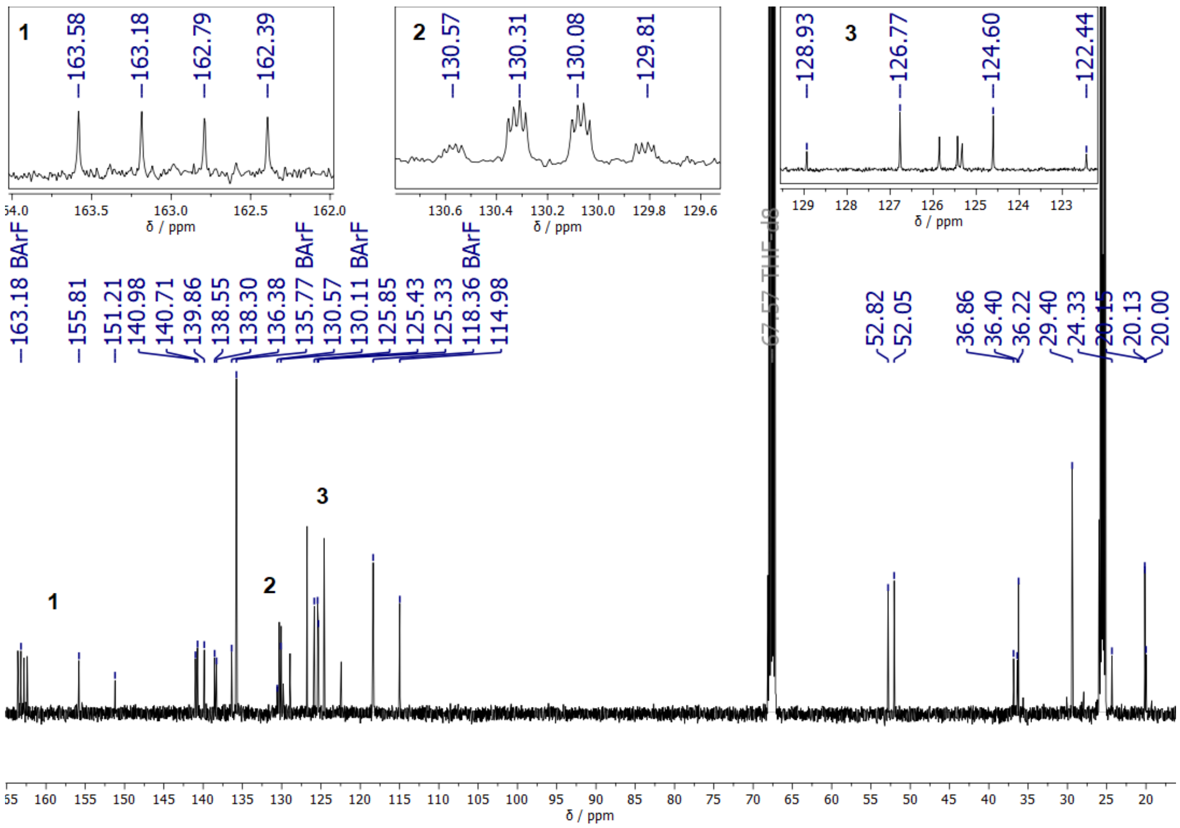

Figure S17. $^{13}C\{^{1}H\}$ NMR spectrum of **2**, THF-d_8, 25 °C; inset: signals of the BArF anion.

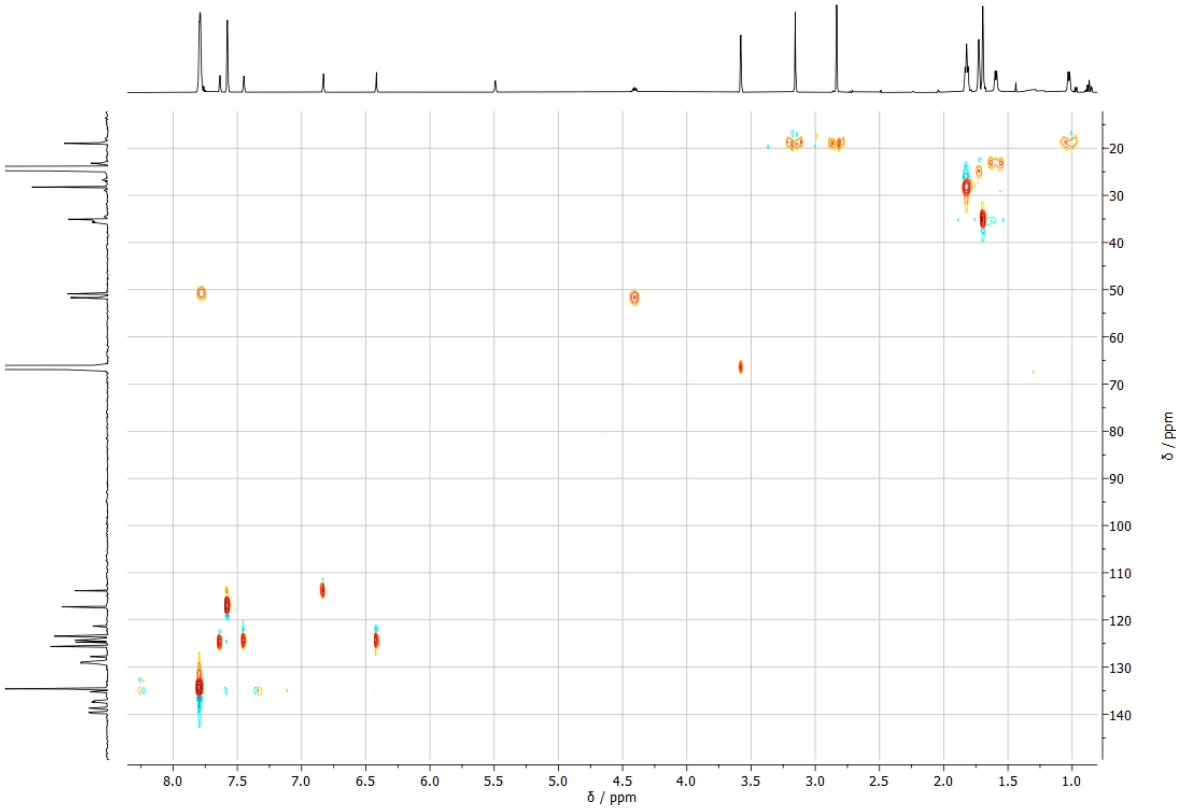

Figure S18. HSQC NMR spectrum of **2**, THF-d_8, 25 °C.

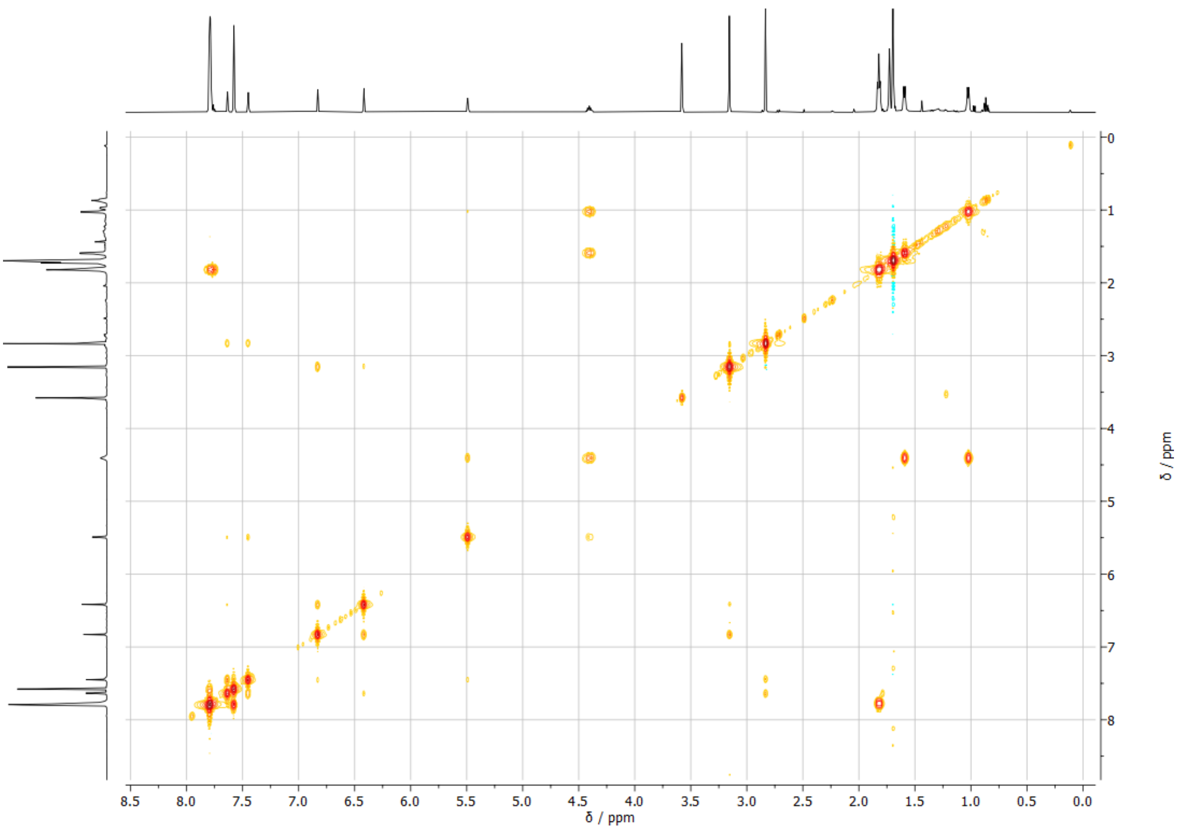

Figure S19. COSY NMR spectrum of **2**, THF-d_8, 25 °C.

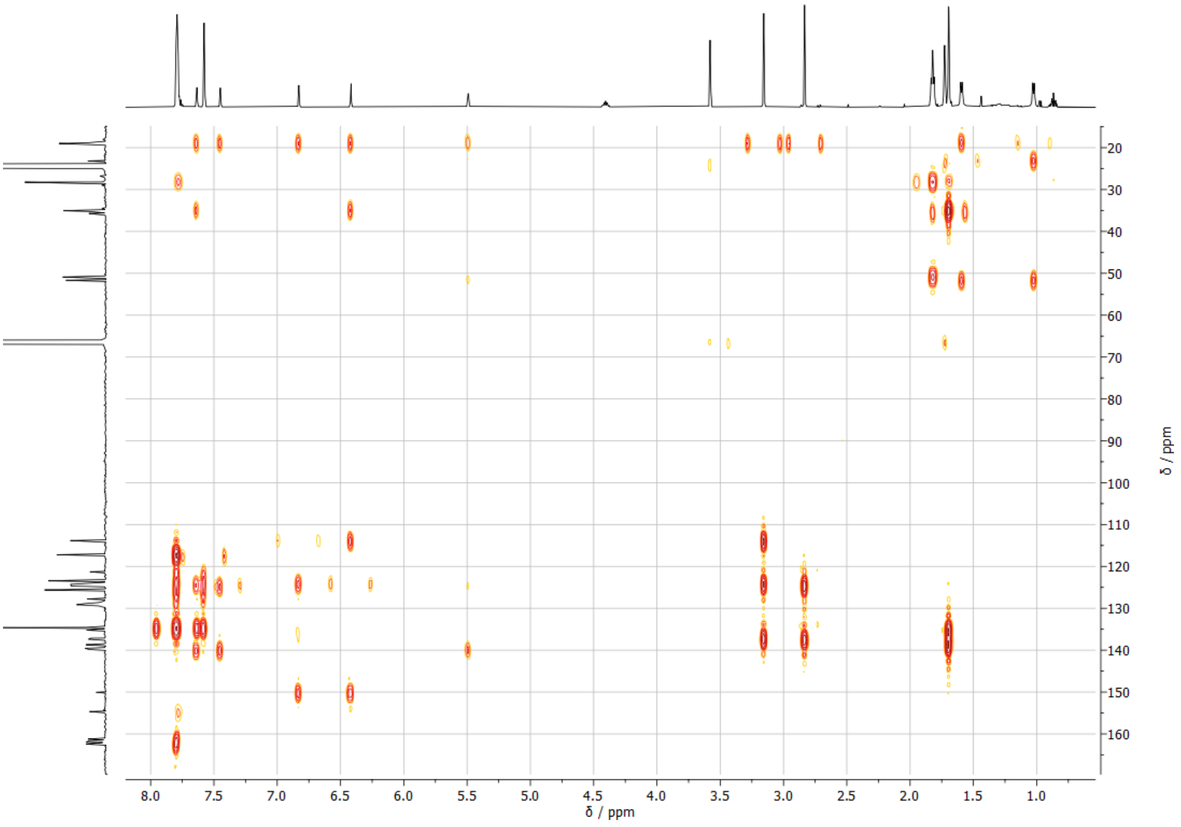

Figure S20. HMBC NMR spectrum of **2**, THF-d_8, 25 °C.

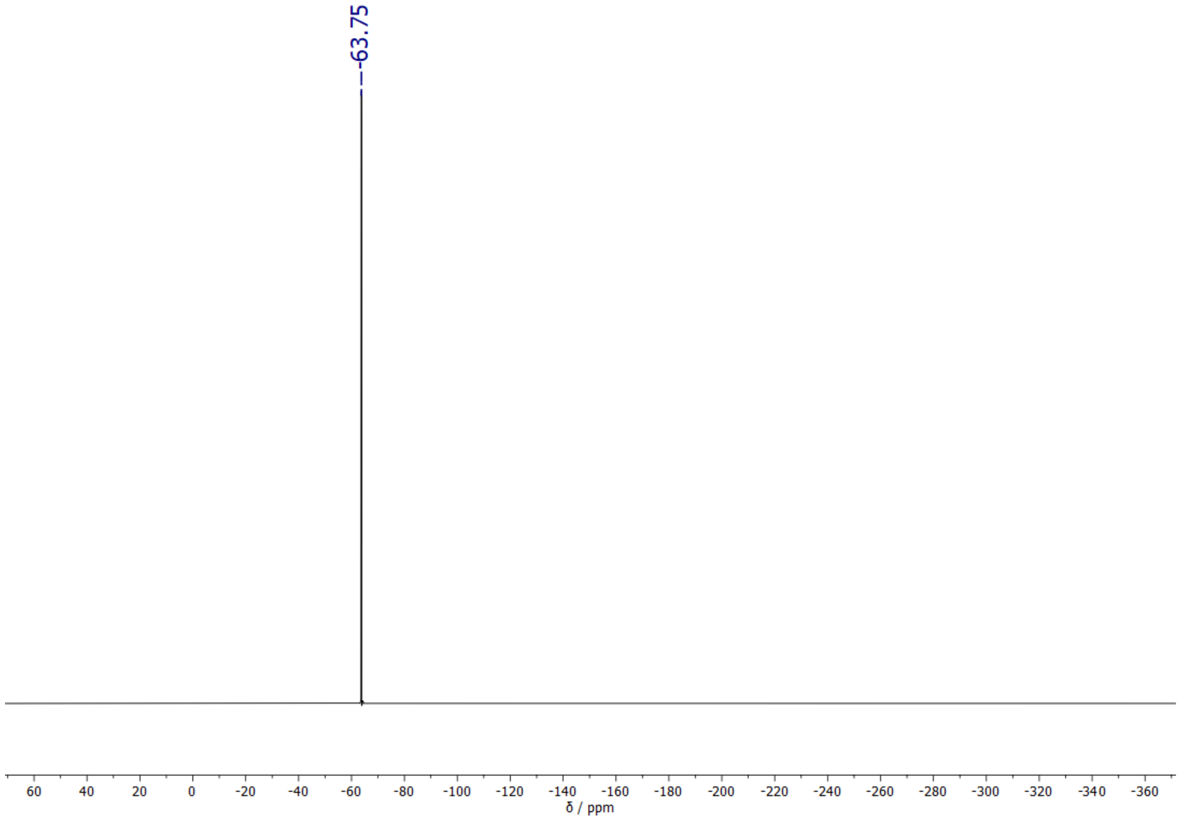

Figure S21. $^{19}F\{^{1}H\}$ NMR spectrum of **2**, THF-d_8, 25 °C.

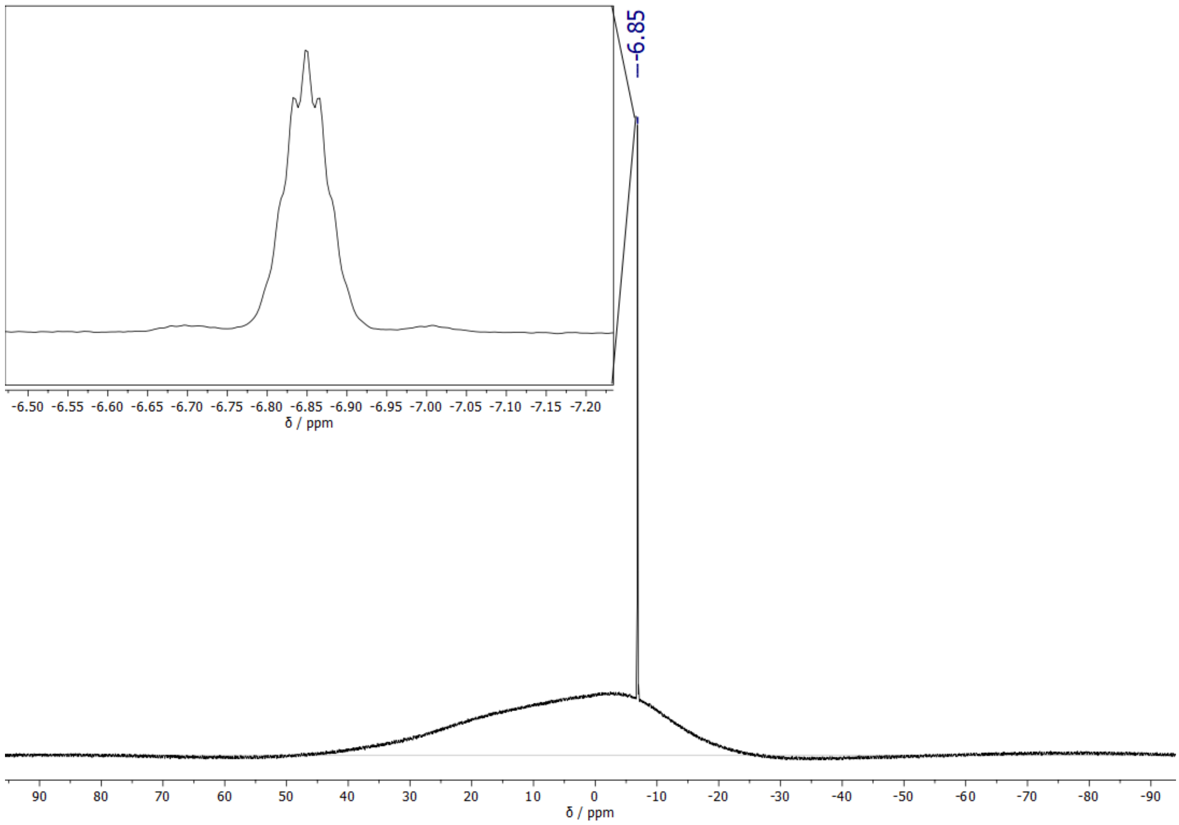

Figure S22. ^{11}B NMR spectrum of **2**, THF-d_8, 25 °C.

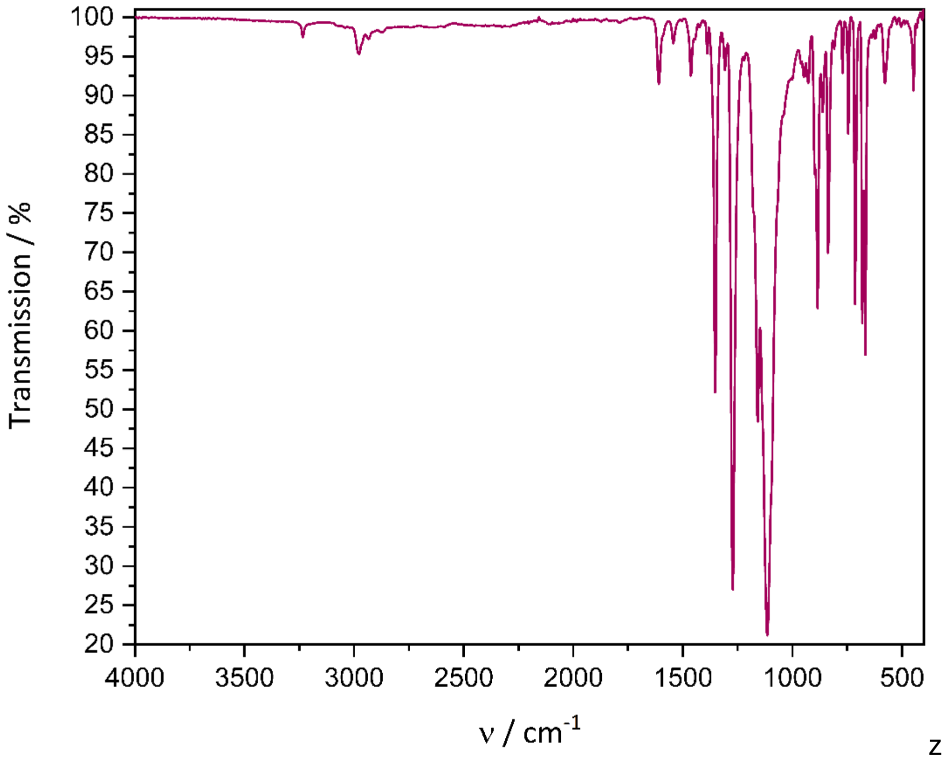

Figure S23. ATR-IR spectrum of **2**, solid, 25 °C.

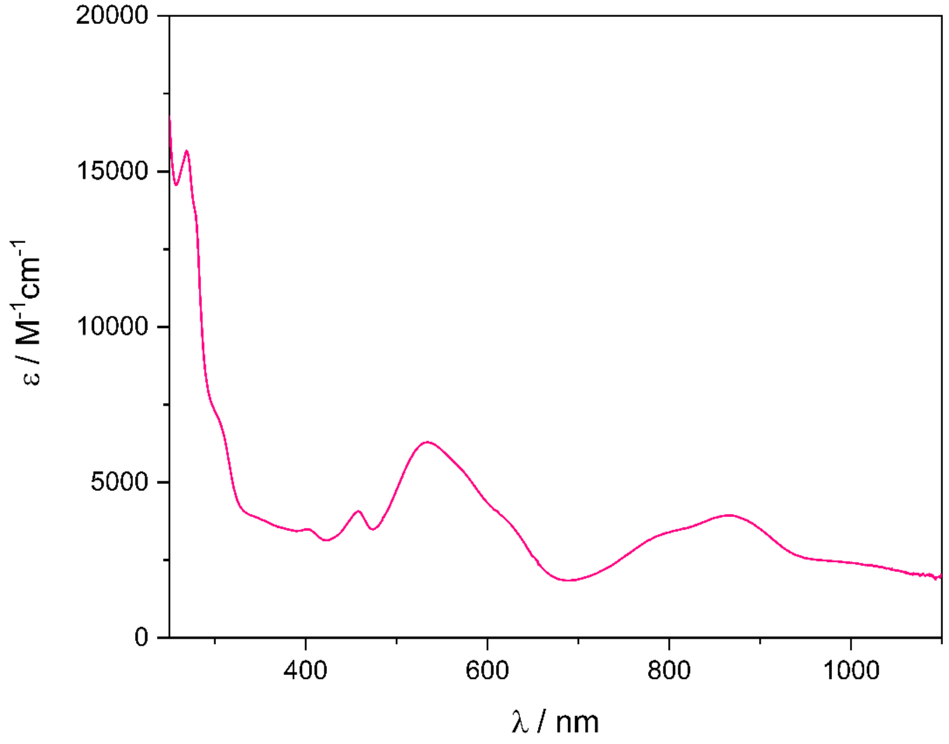

Figure S24. UV/Vis spectrum of **2**, DCM, 25 °C.

Spectroscopy of 3^{OTf}

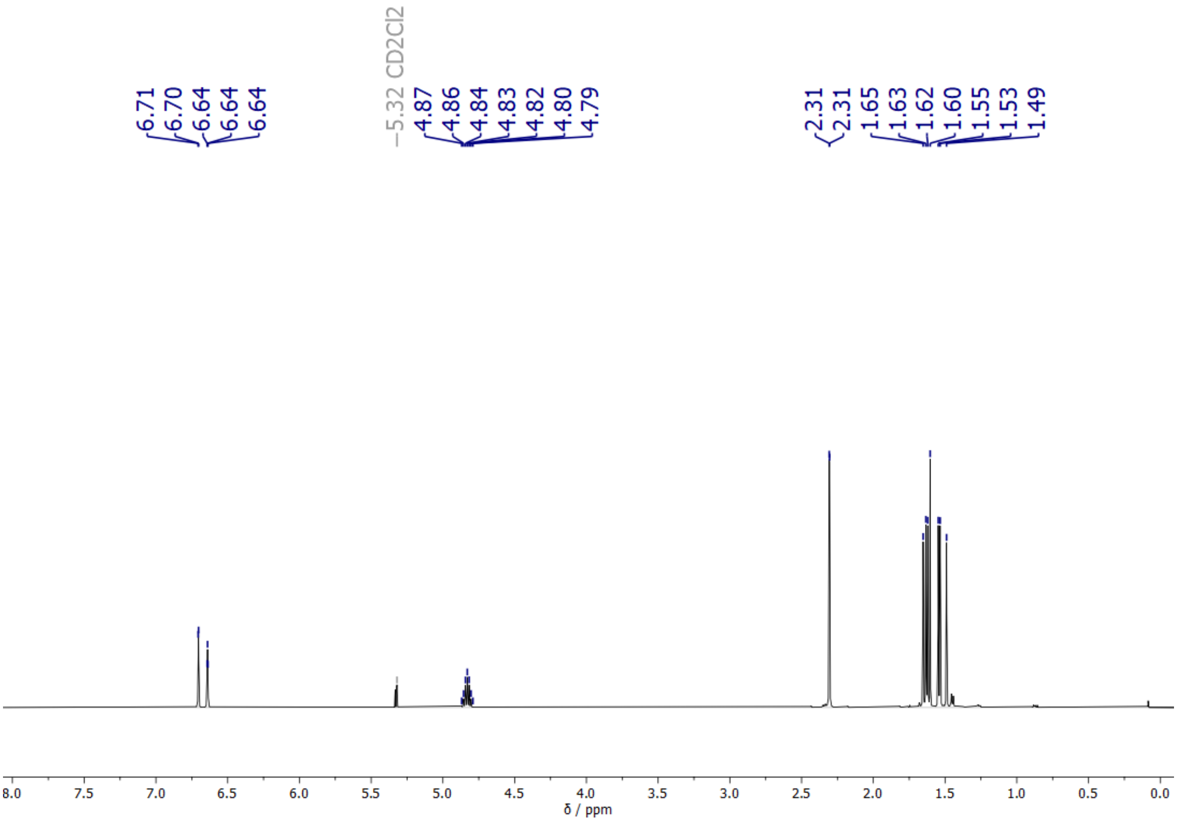

Figure S25. ^{1}H NMR spectrum of $\mathbf{3}^{\mathbf{OTf}}$, CD_2Cl_2, 25 °C.

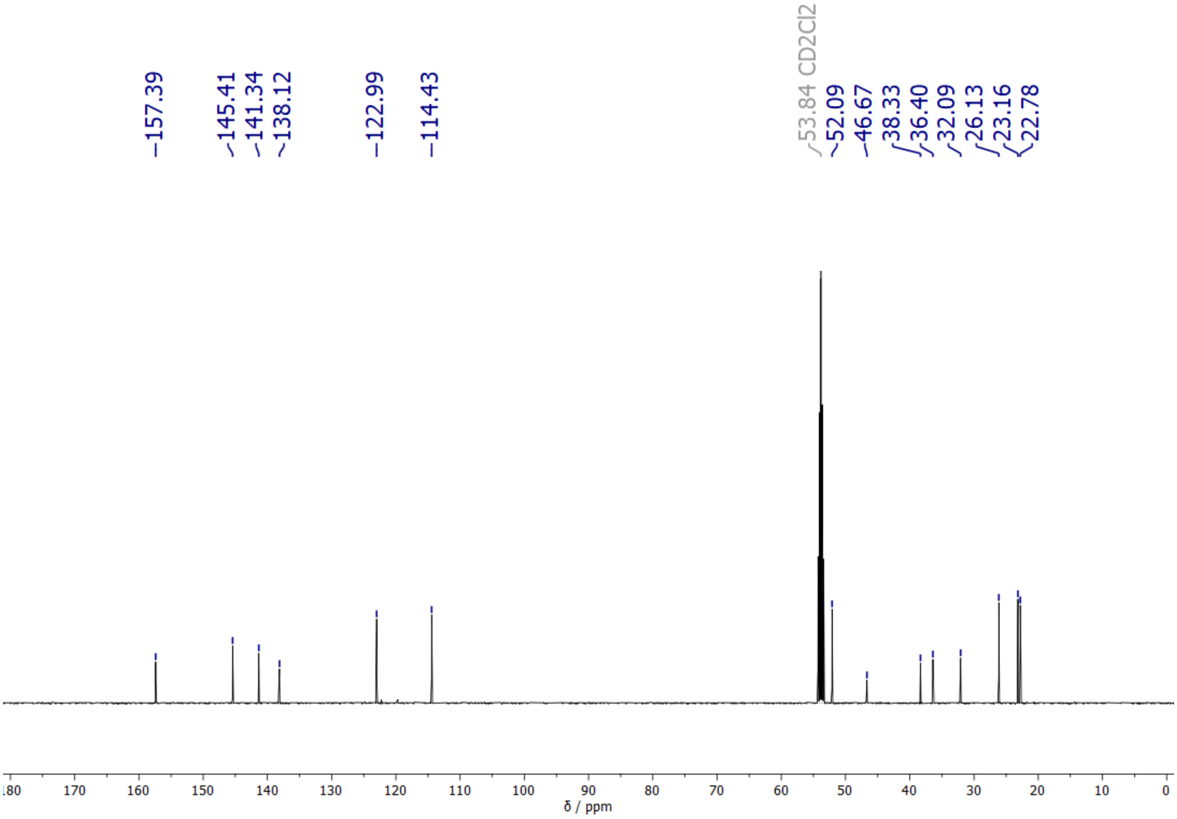

Figure S26. $^{13}C\{^{1}H\}$ NMR spectrum of $\mathbf{3}^{\mathbf{OTf}}$, CD_2Cl_2, 25 °C.

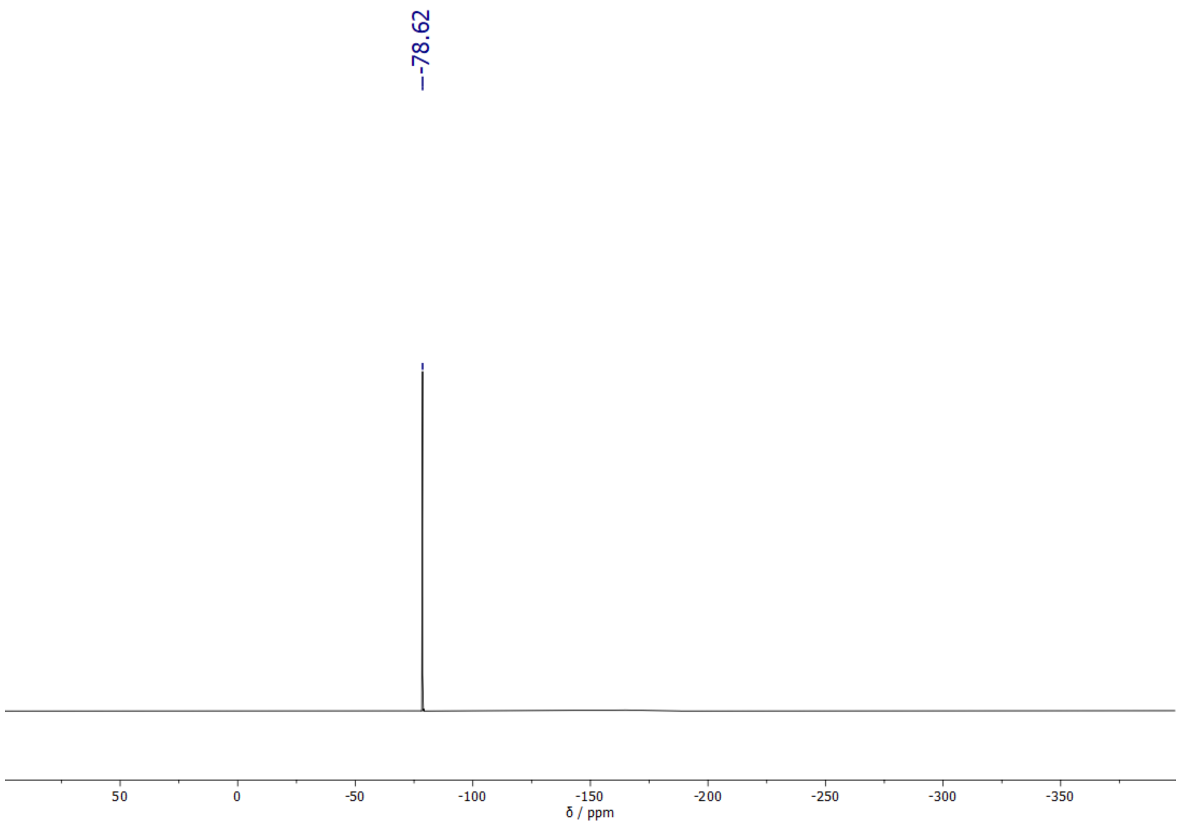

Figure S27. $^{19}F\{^1H\}$ NMR spectrum of **3**^{OTf}, CD_2Cl_2, 25 °C.

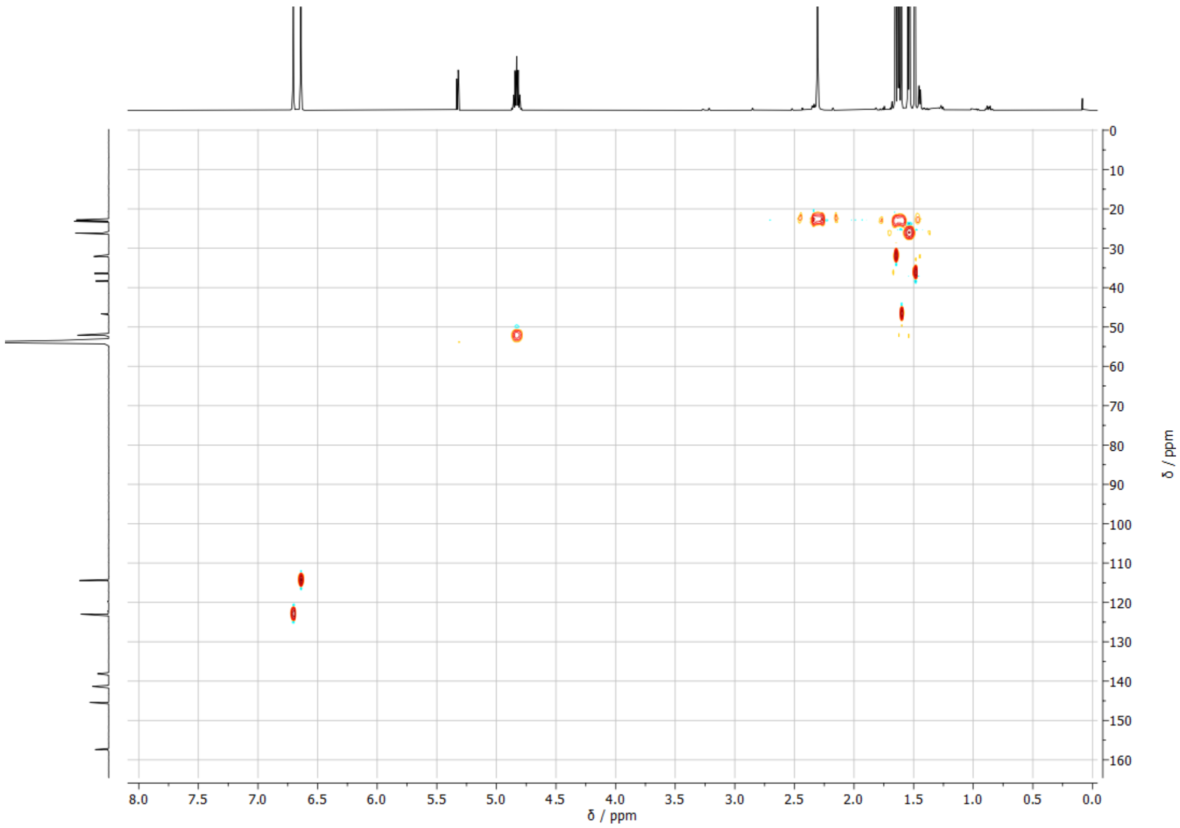

Figure S28. HSQC NMR spectrum of **3**^{OTf}, CD_2Cl_2, 25 °C.

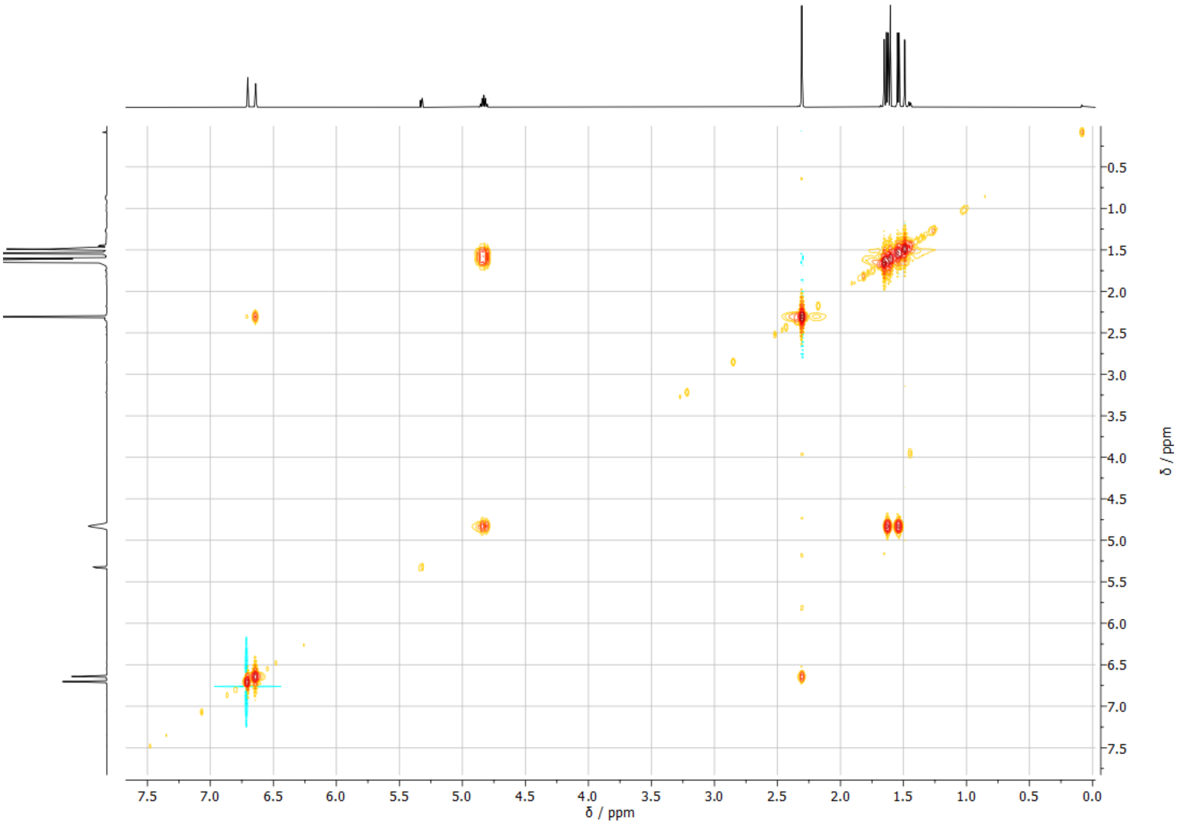

Figure S29. COSY NMR spectrum of **3**^{OTf}, CD_2Cl_2, 25 °C.

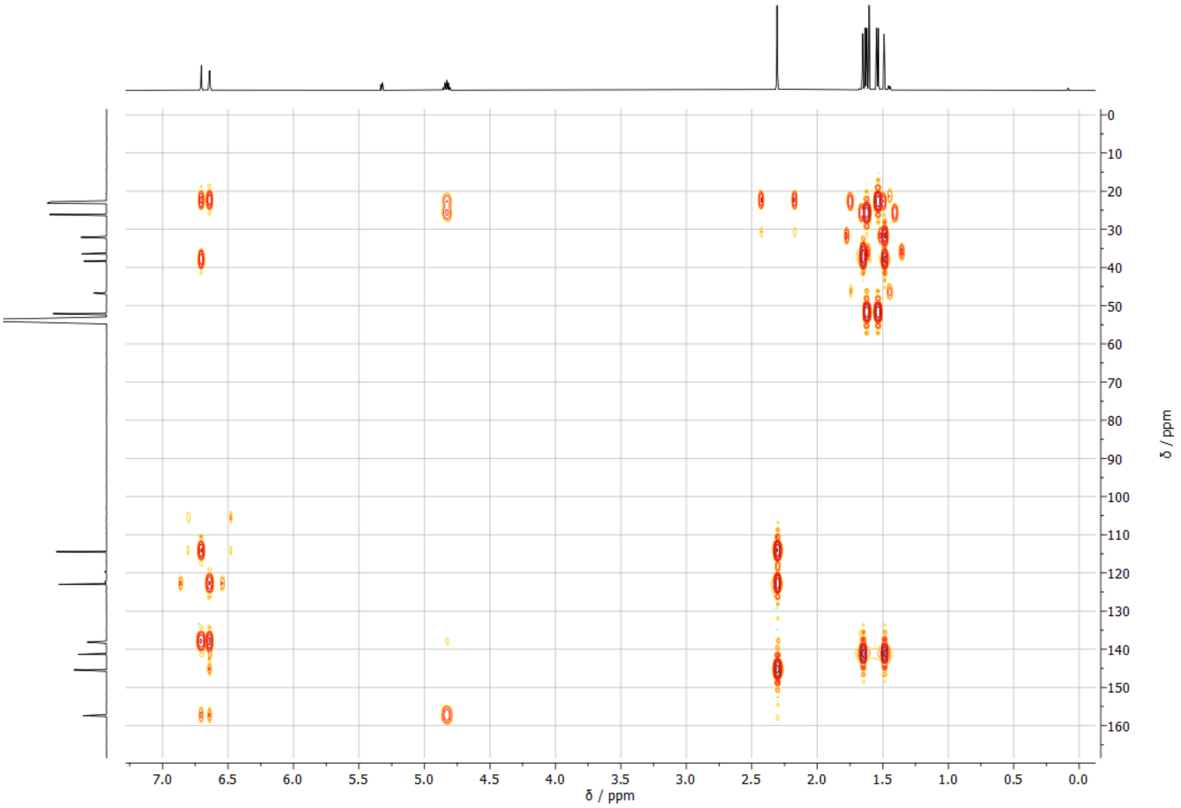

Figure S30. HMBC NMR spectrum of **3**^{OTf}, CD_2Cl_2, 25 °C.

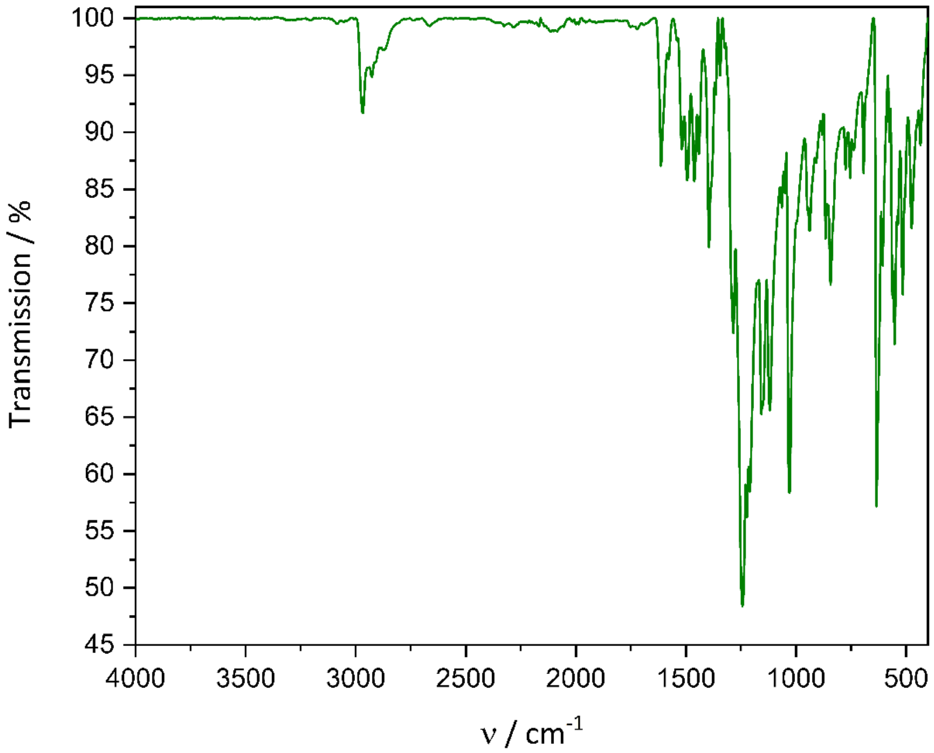

Figure S31. ATR-IR spectrum of **3**^{OTf}, solid, 25 °C.

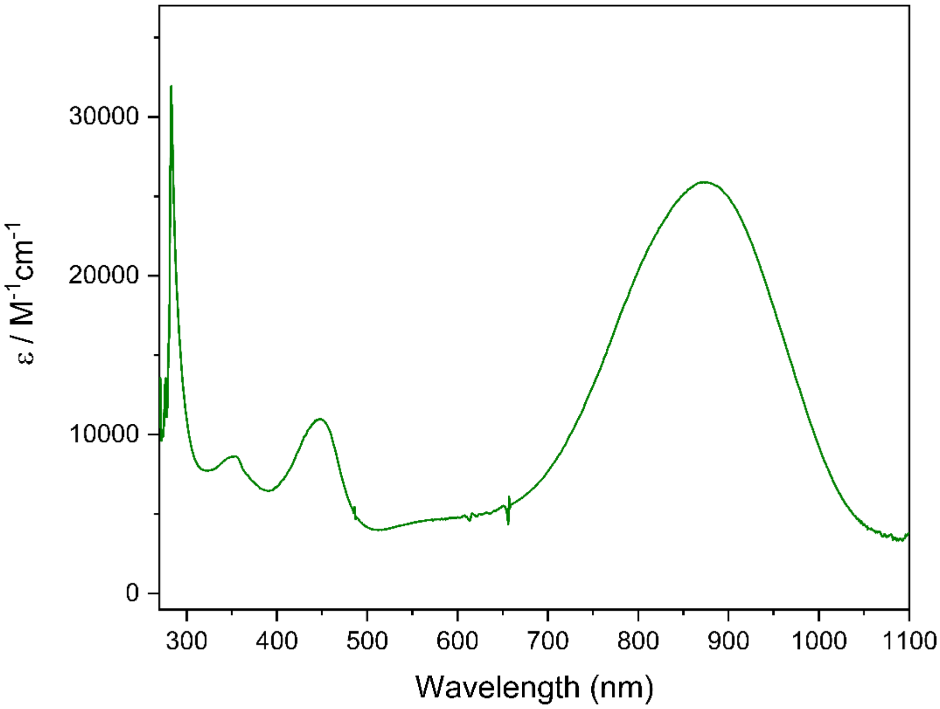

Figure S32. UV/Vis spectrum of **3**^{OTf}, toluene, 25 °C.

Spectroscopy of 3^{BArF}

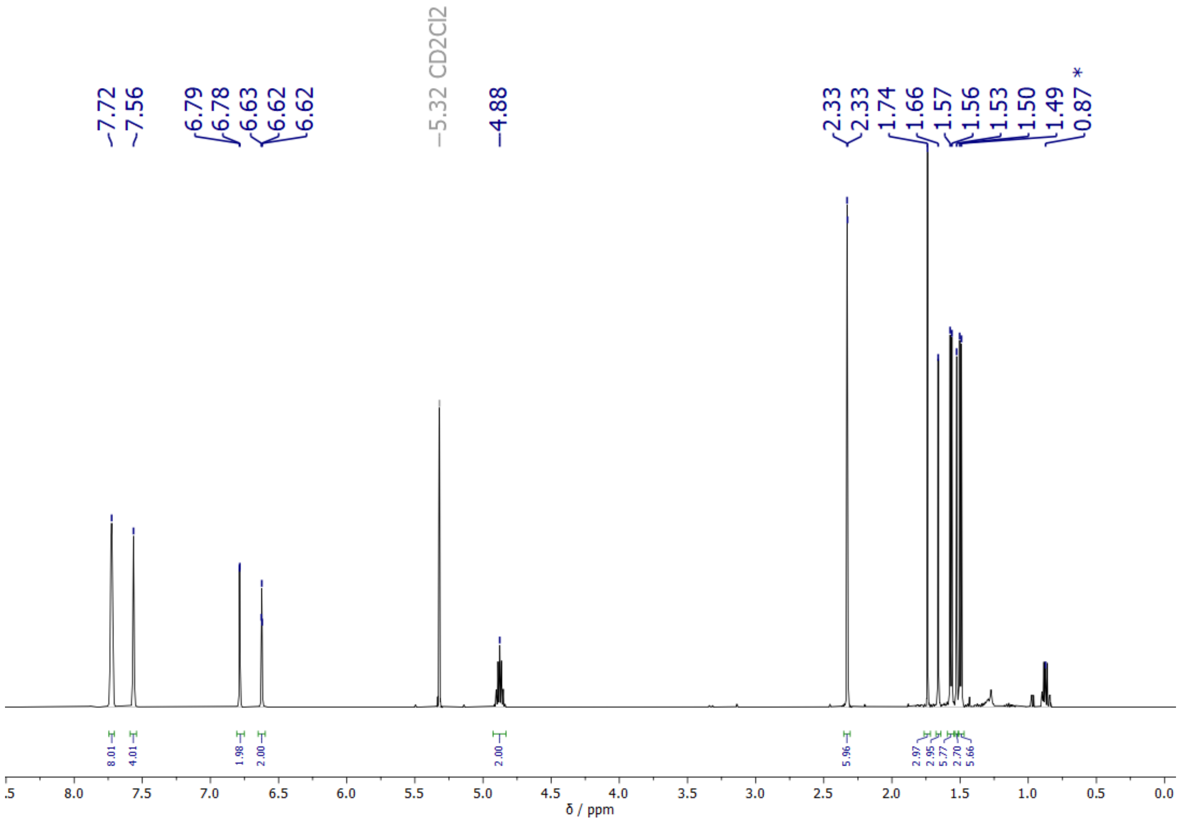

Figure S33. ^{1}H NMR spectrum of **3^{BArF}**, CD_2Cl_2, 25 °C; H grease marked with *.

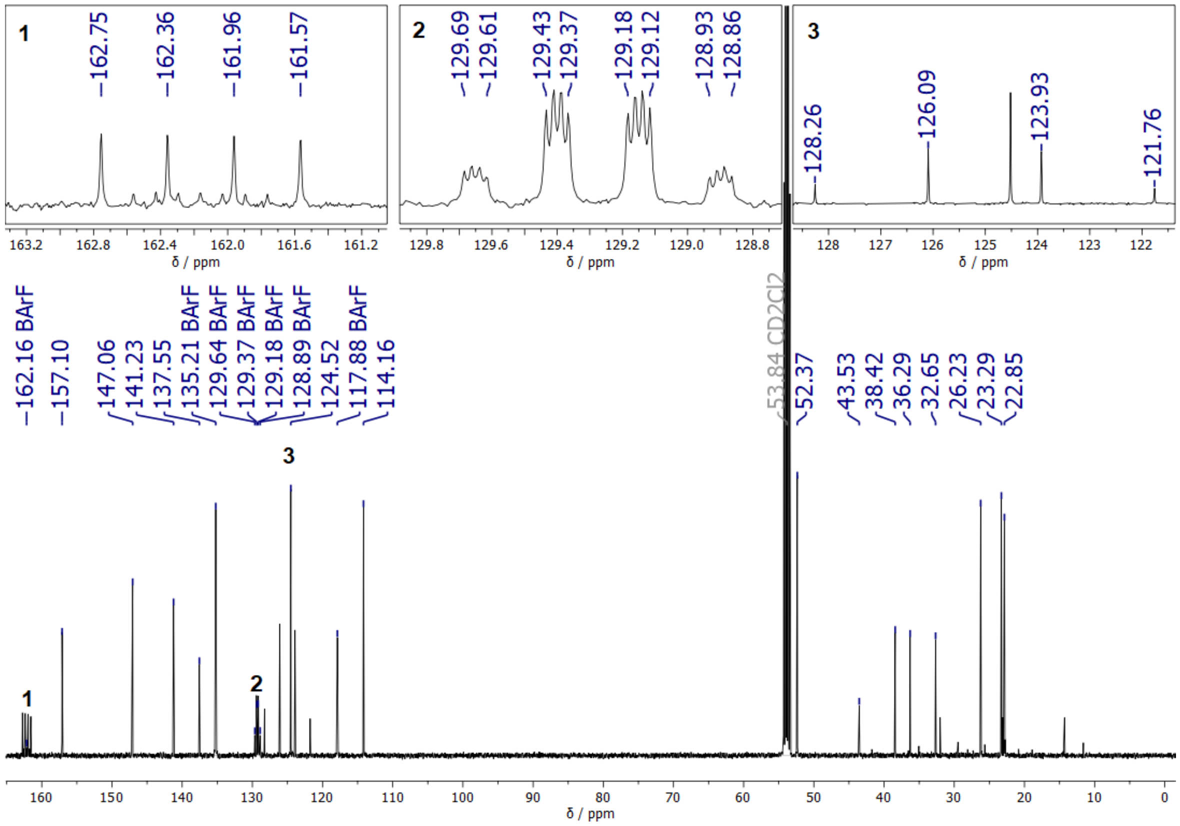

Figure S34. ^{13}C{^{1}H} NMR spectrum of **3^{BArF}**, CD_2Cl_2, 25 °C; inset: signals of the BArF anion.

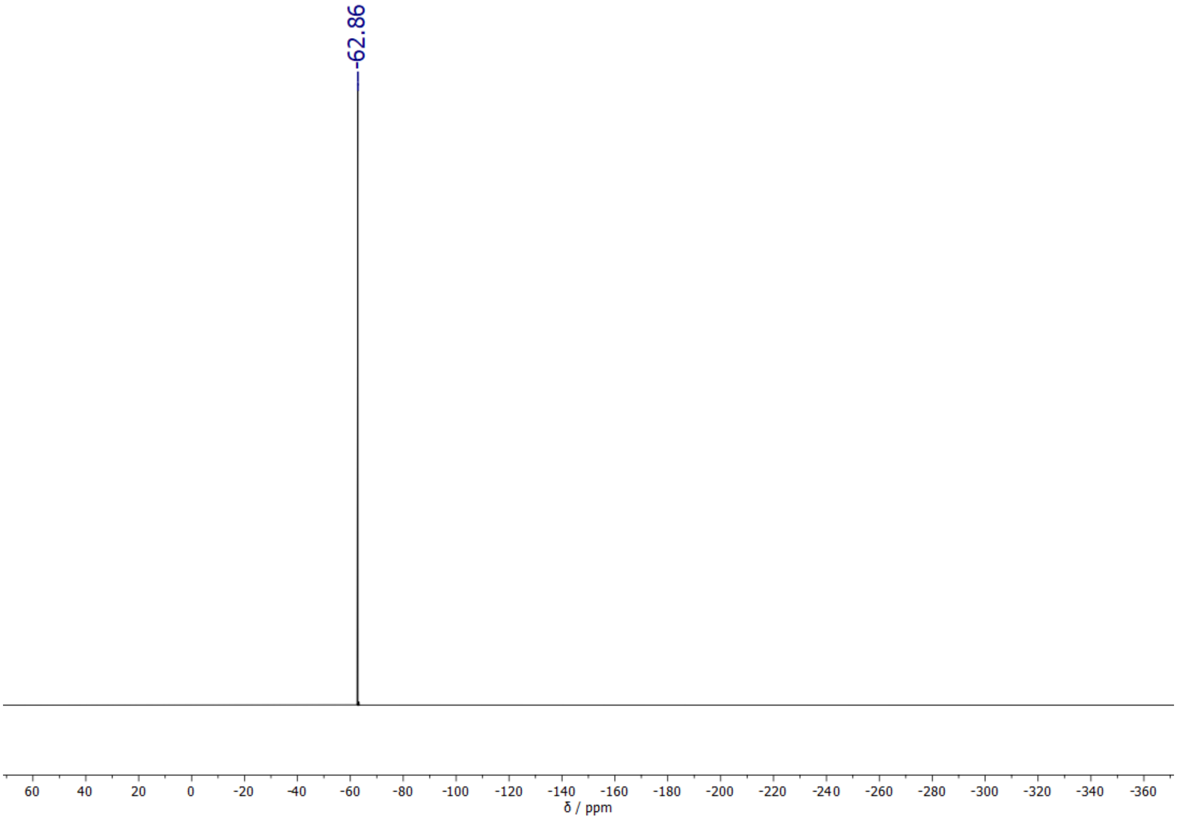

Figure S35. $^{19}F\{^{1}H\}$ NMR spectrum of $\mathbf{3}^{\mathbf{BArF}}$, CD_2Cl_2, 25 °C.

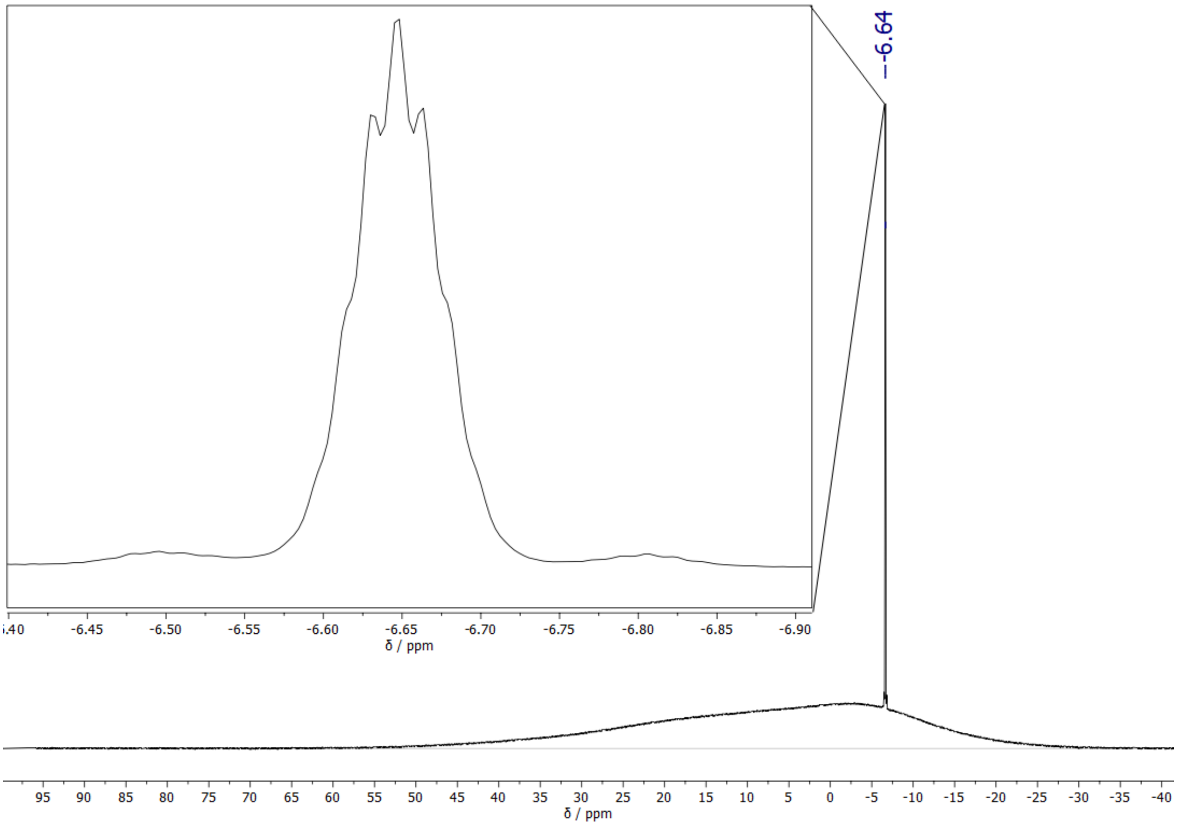

Figure S36. ^{11}B NMR spectrum of $\mathbf{3}^{\mathbf{BArF}}$, CD_2Cl_2, 25 °C.

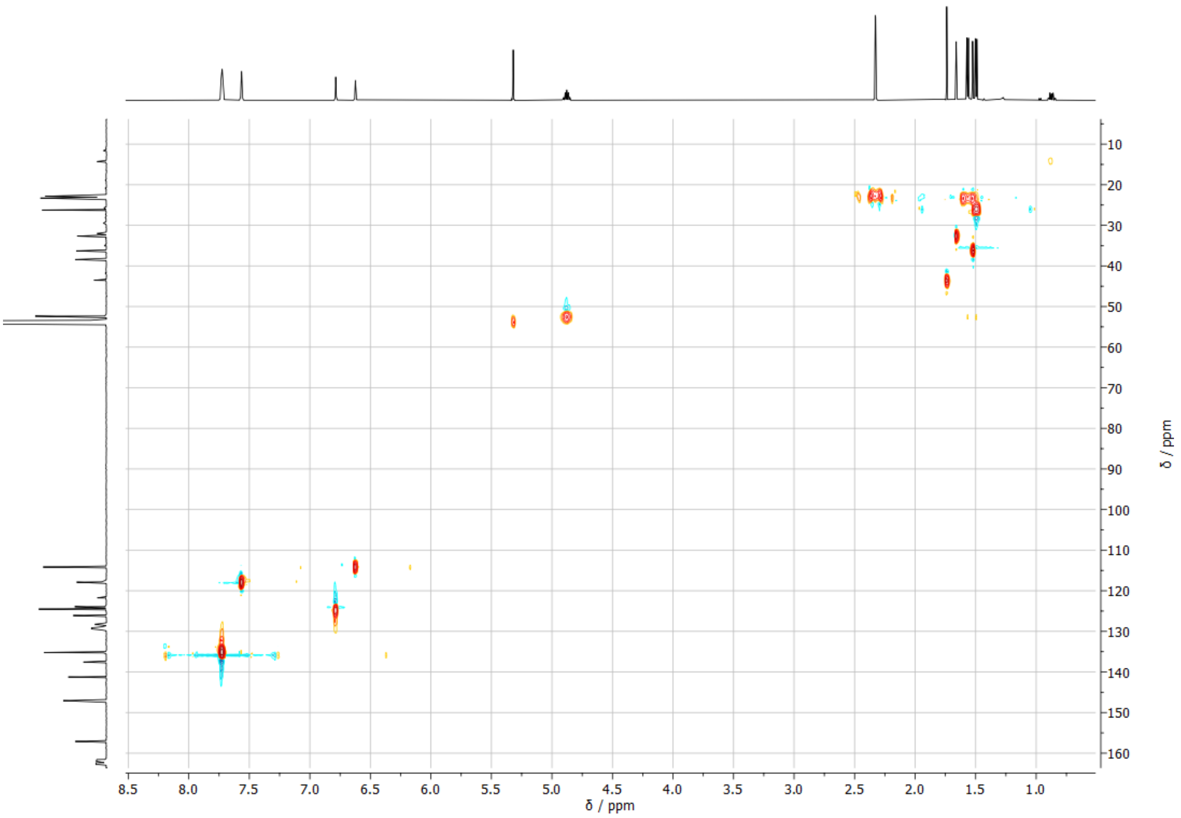

Figure S37. HSQC NMR spectrum of $\mathbf{3}^{\mathbf{BArF}}$, CD_2Cl_2, 25 °C.

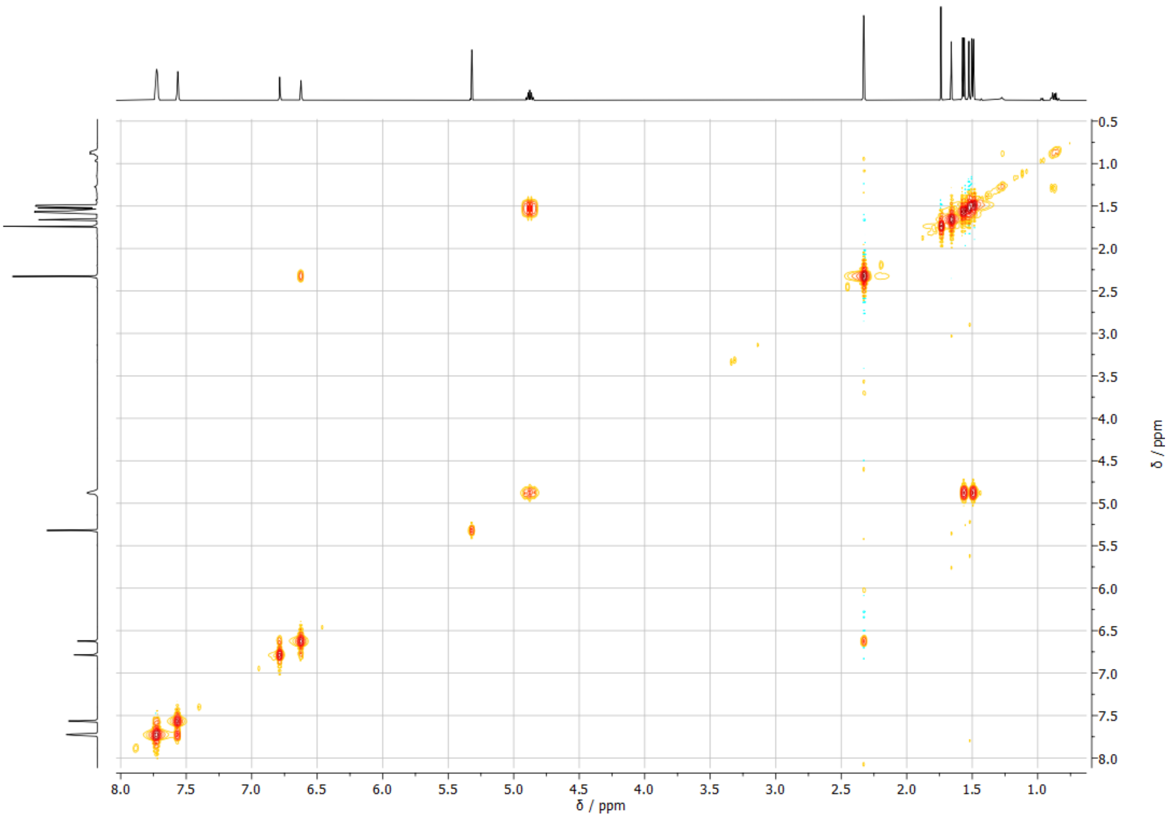

Figure S38. COSY NMR spectrum of $\mathbf{3}^{\mathbf{BArF}}$, CD_2Cl_2, 25 °C.

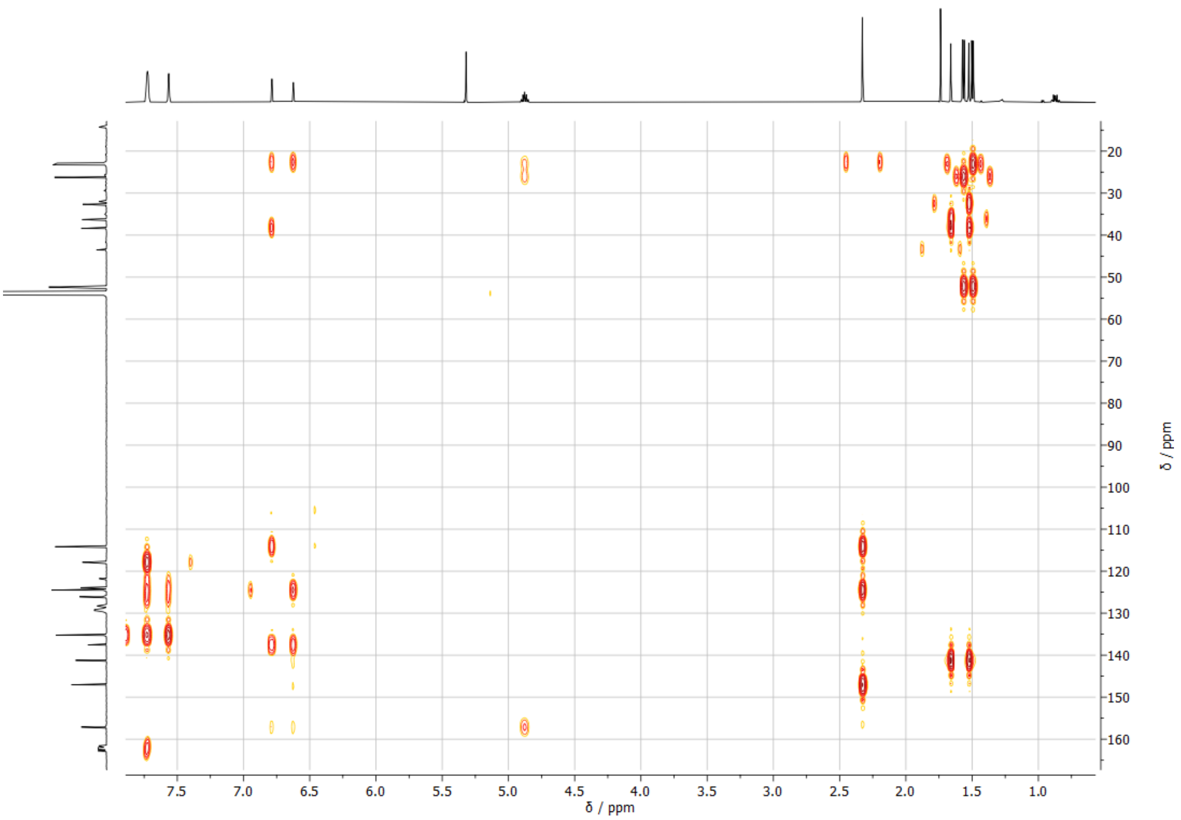

Figure S39. HMBC NMR spectrum of **3**^{BArF}, CD_2Cl_2, 25 °C.

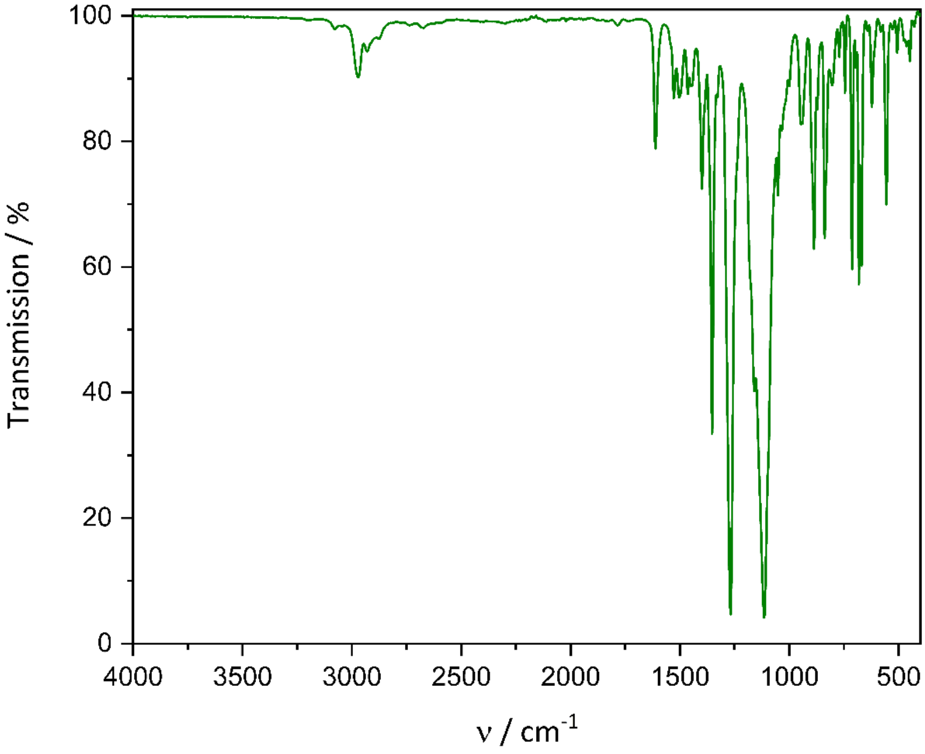

Figure S40. ATR-IR spectrum of $\mathbf{3}^{\mathbf{BArF}}$, solid, 25 °C.

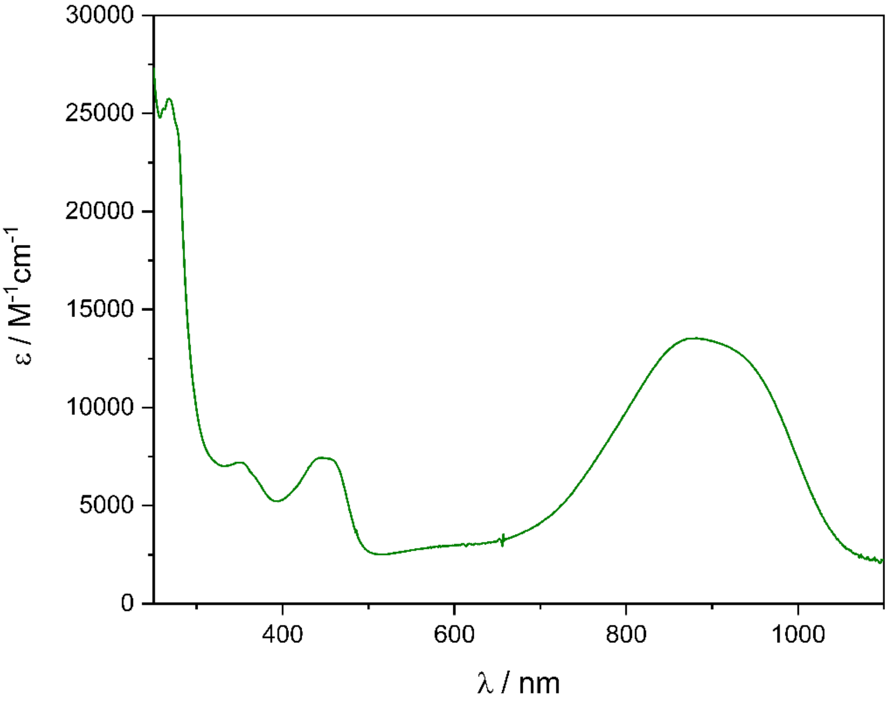

Figure S41. UV/Vis spectrum of $\mathbf{3}^{\mathbf{BArF}}$, THF, 25 °C.

Spectroscopy of 3^{Et_BArF}

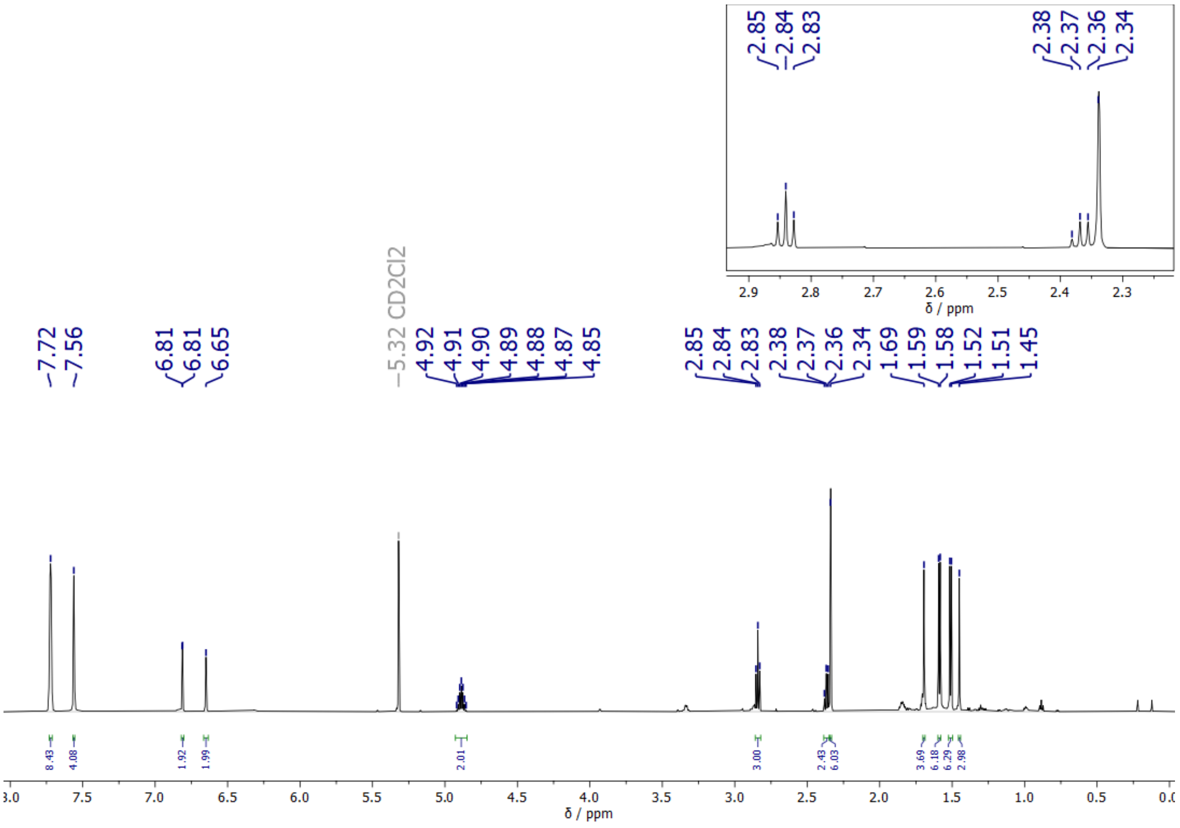

Figure S42. ^{1}H NMR spectrum of $\mathbf{3^{Et\_BArF}}$, CD_2Cl_2, 25 °C; inset: signals of the Bi*Et* moiety.

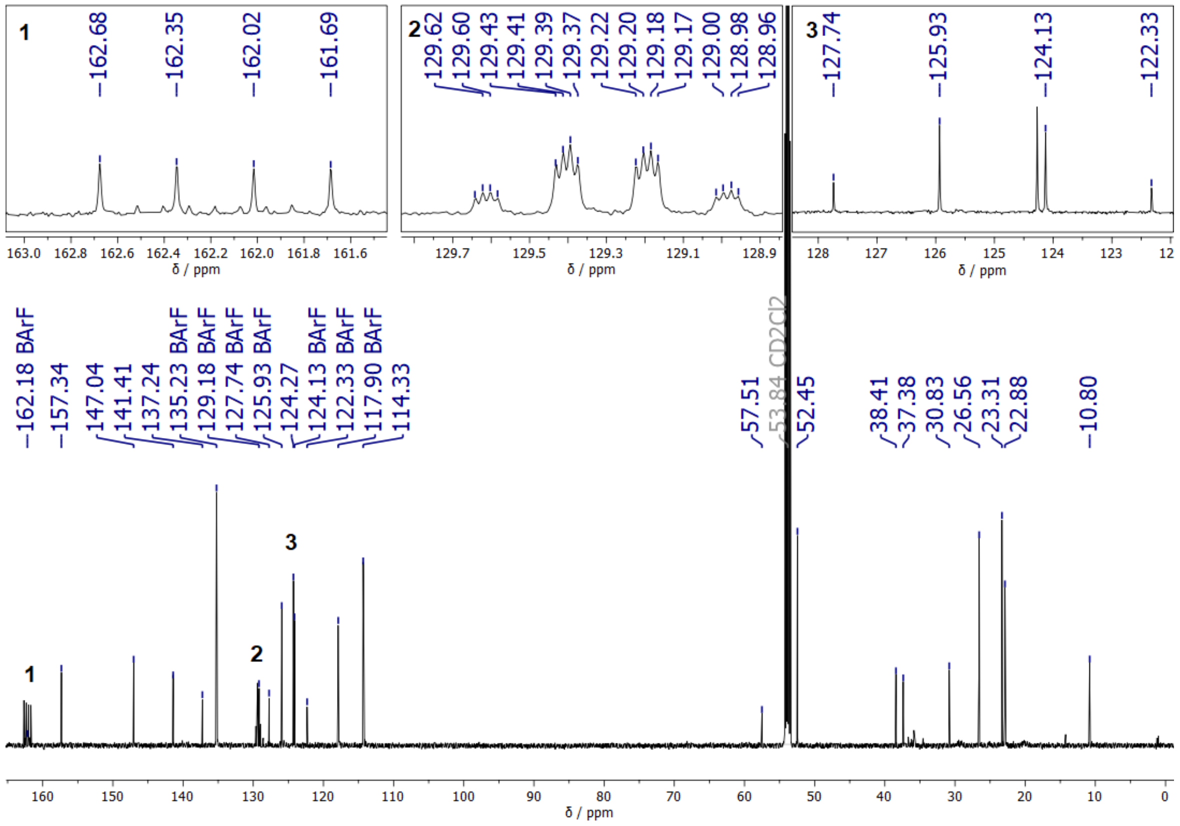

Figure S43. $^{13}C\{^{1}H\}$ NMR spectrum of $\mathbf{3^{Et\_BArF}}$, CD_2Cl_2, 25 °C; inset: signals of the BArF anion.

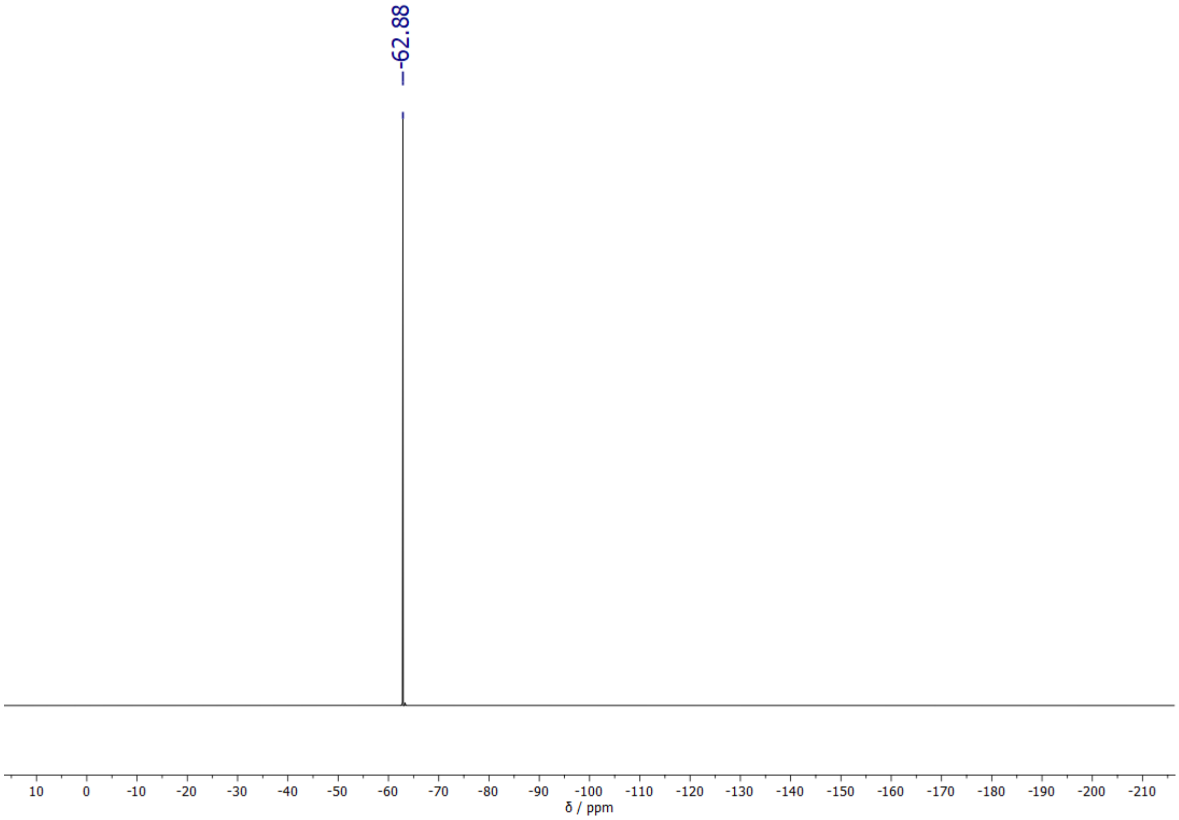

Figure S44. $^{19}F\{^{1}H\}$ NMR spectrum of $\mathbf{3}^{\mathbf{Et\_BArF}}$, CD_2Cl_2, 25 °C.

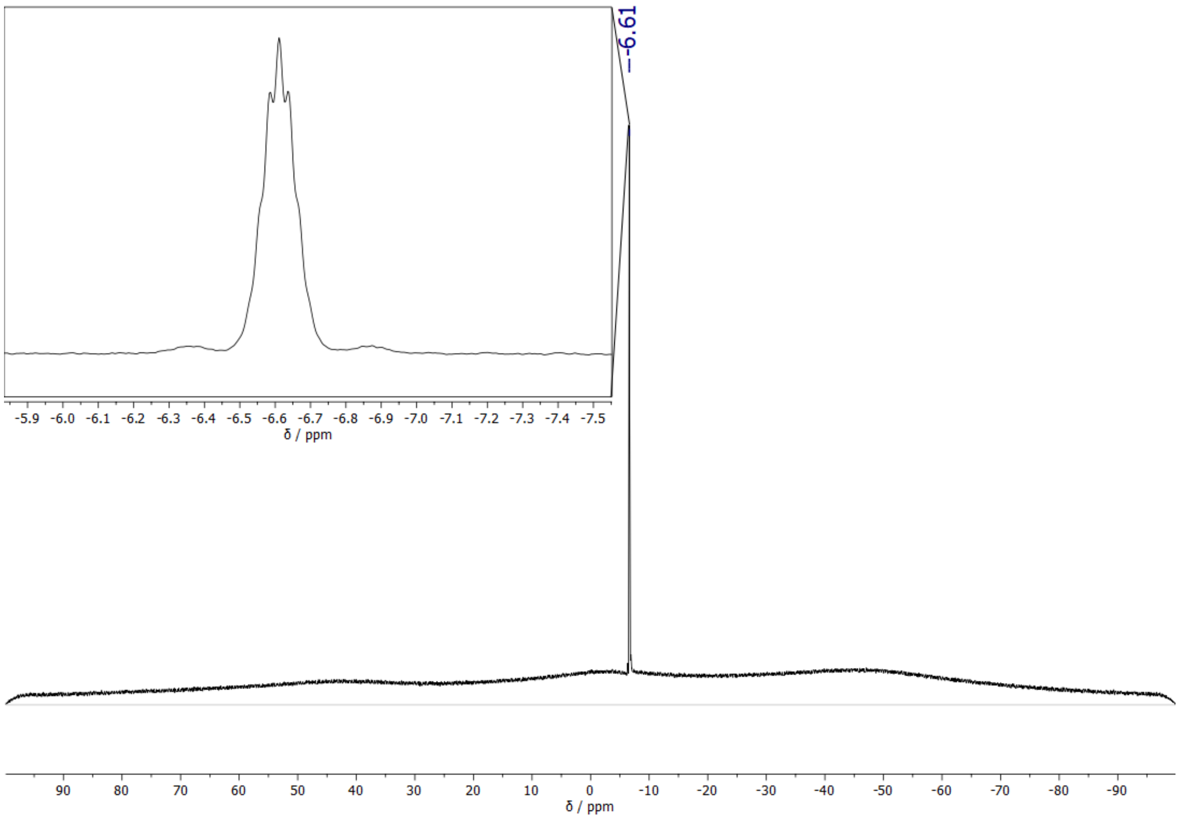

Figure S45. ^{11}B NMR spectrum of $\mathbf{3}^{\mathbf{Et\_BArF}}$, CD_2Cl_2, 25 °C.

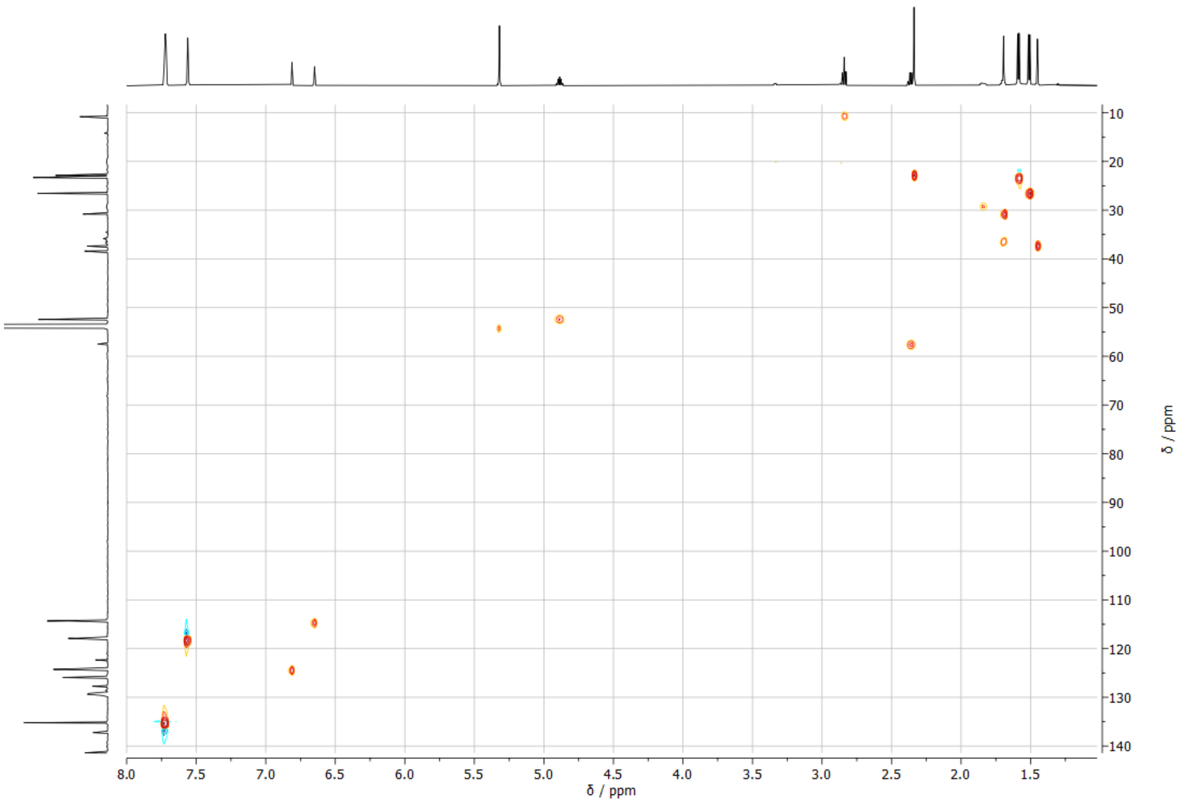

Figure S46. HSQC NMR spectrum of 3^{Et_BArF}, CD_2Cl_2, 25 °C.

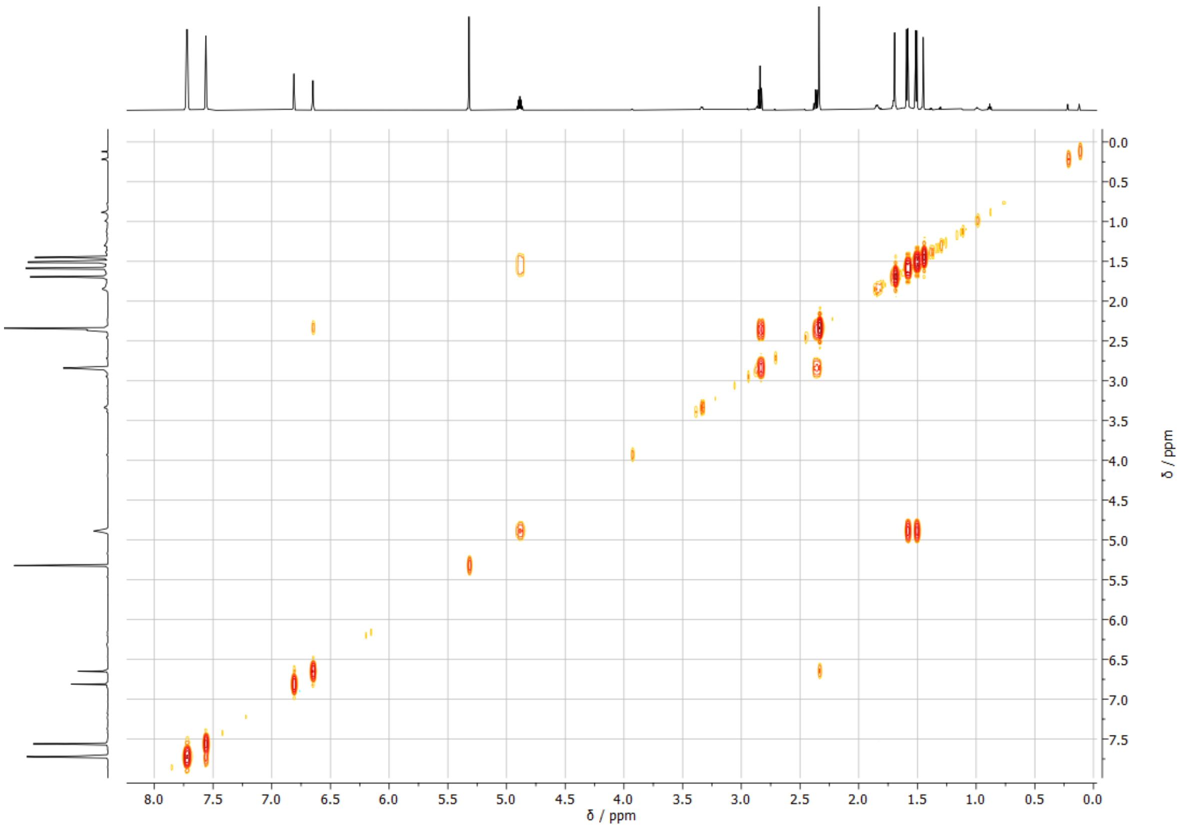

Figure S47. COSY NMR spectrum of 3^{Et_BArF}, CD_2Cl_2, 25 °C.

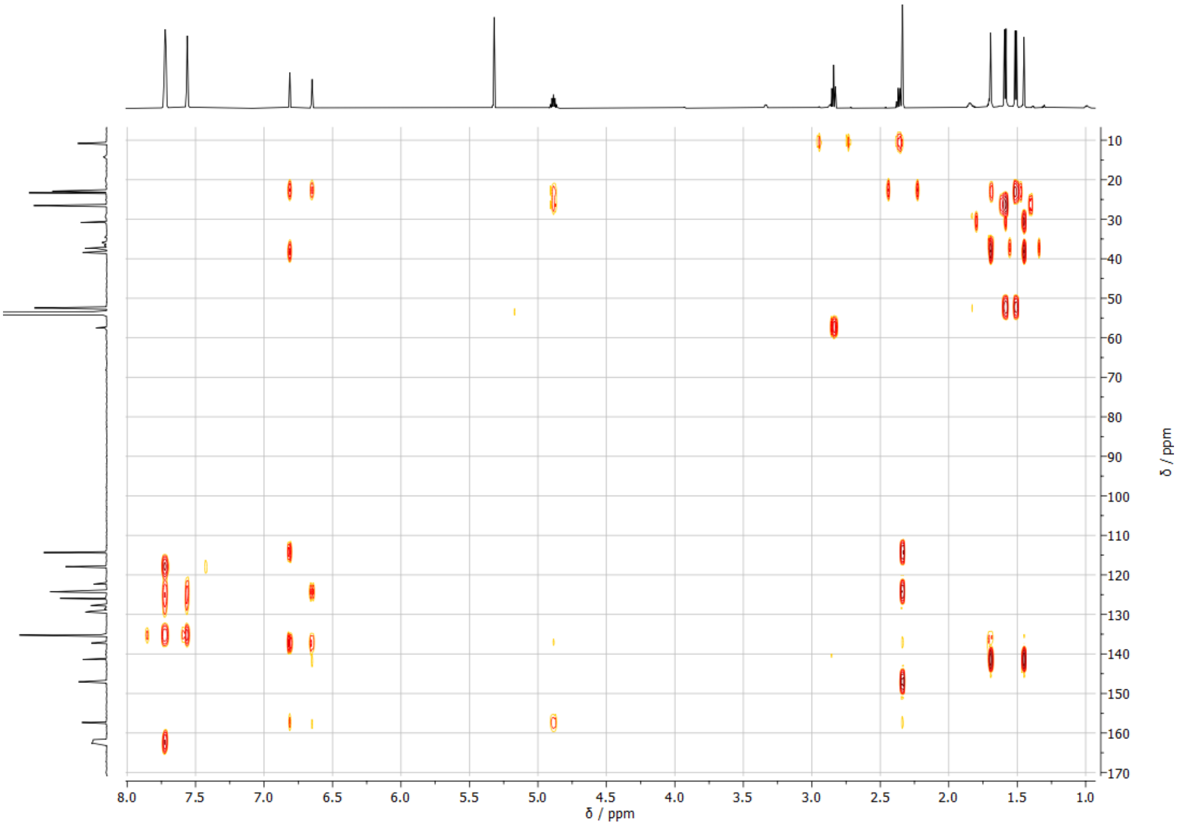

Figure S48. HMBC NMR spectrum of **3**$^{Et\_BArF}$, CD_2Cl_2, 25 °C.

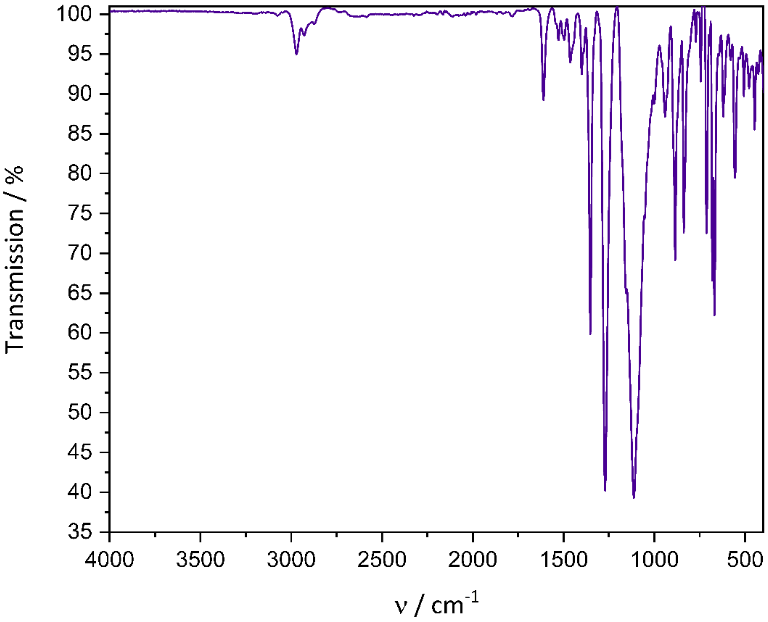

Figure S49. ATR-IR spectrum of **3**$^{Et\_BArF}$, solid, 25 °C.

NMR Spectroscopy of the One-Electron Reduction of 3^{BArF} by $CoCp_2$

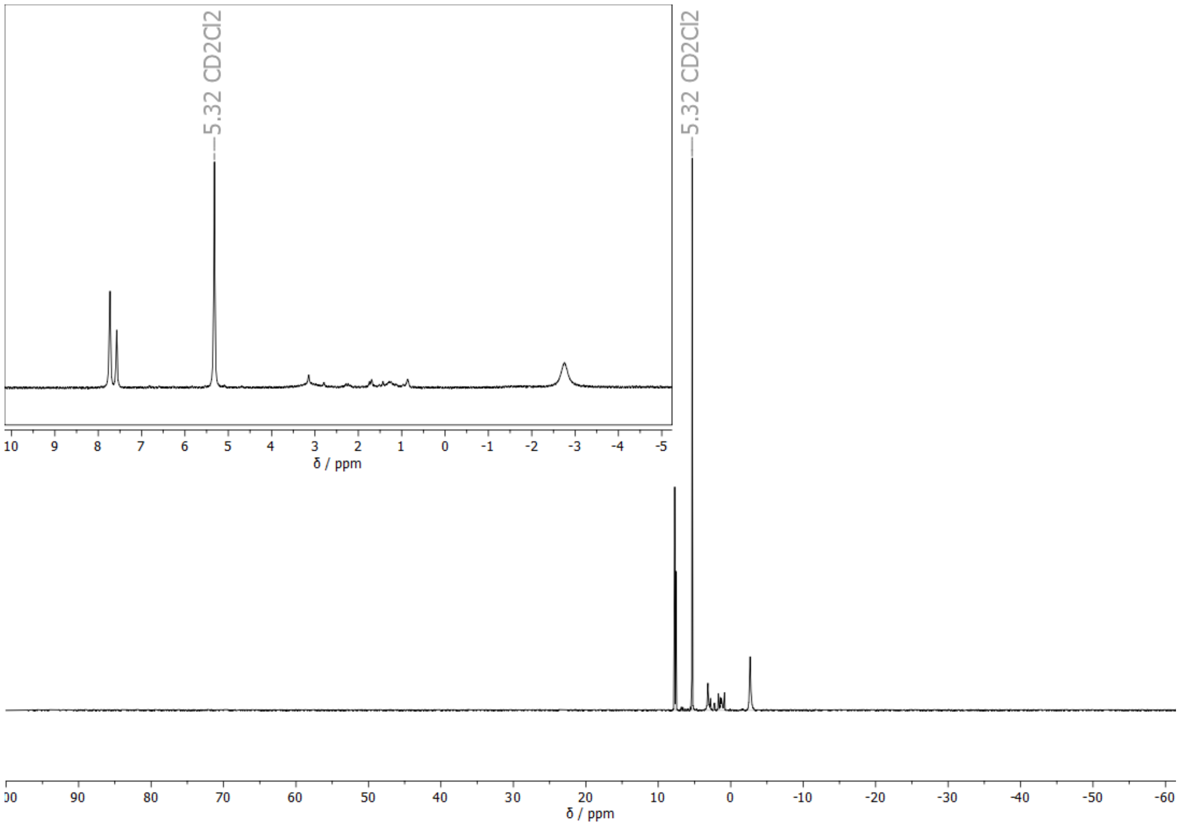

Figure S50. ^{1}H NMR spectrum of freshly prepared **4**, CD_2Cl_2, 25 °C.

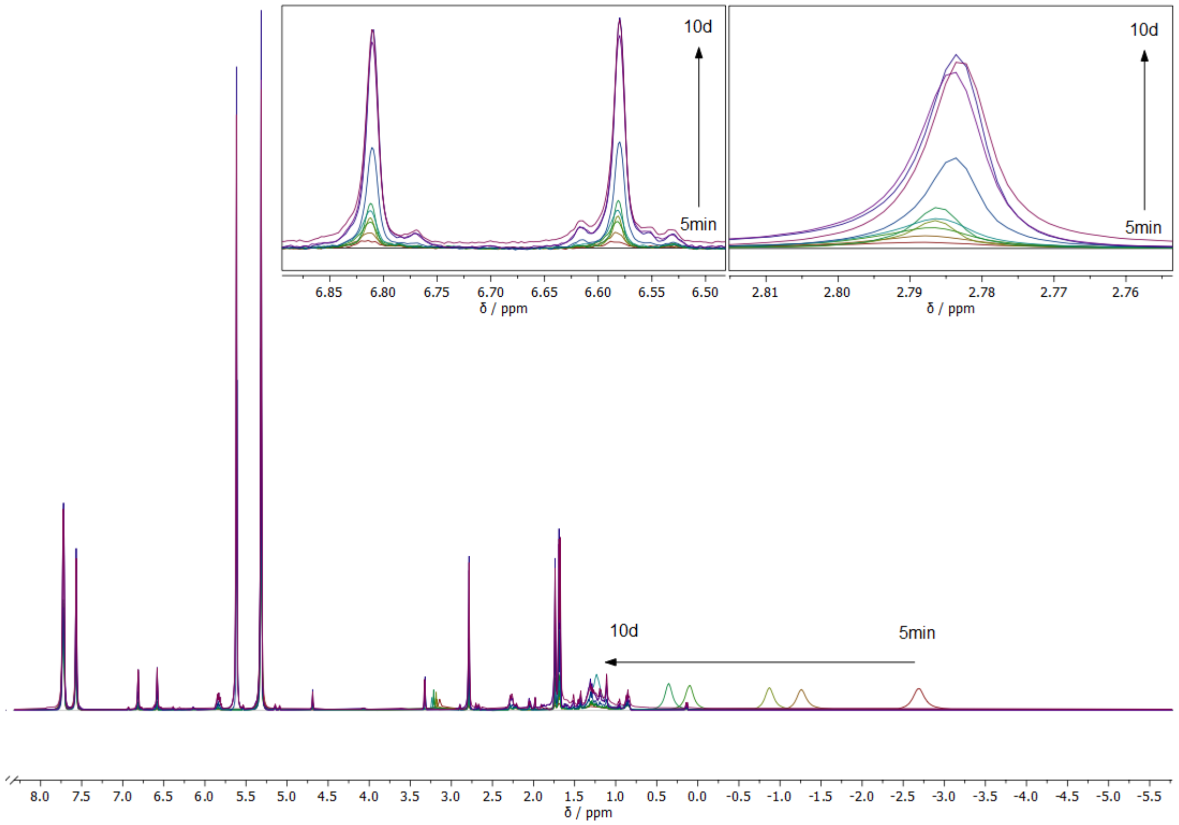

Figure S51. ^{1}H NMR spectrum of **4** over time, CD_2Cl_2, 25 °C. t = 10min, 45min, 1.15h, 2h, 3h, 3.5h, 3d, 6d, 7d, 10d (red to violet).

NMR and IR Spectroscopy of 5

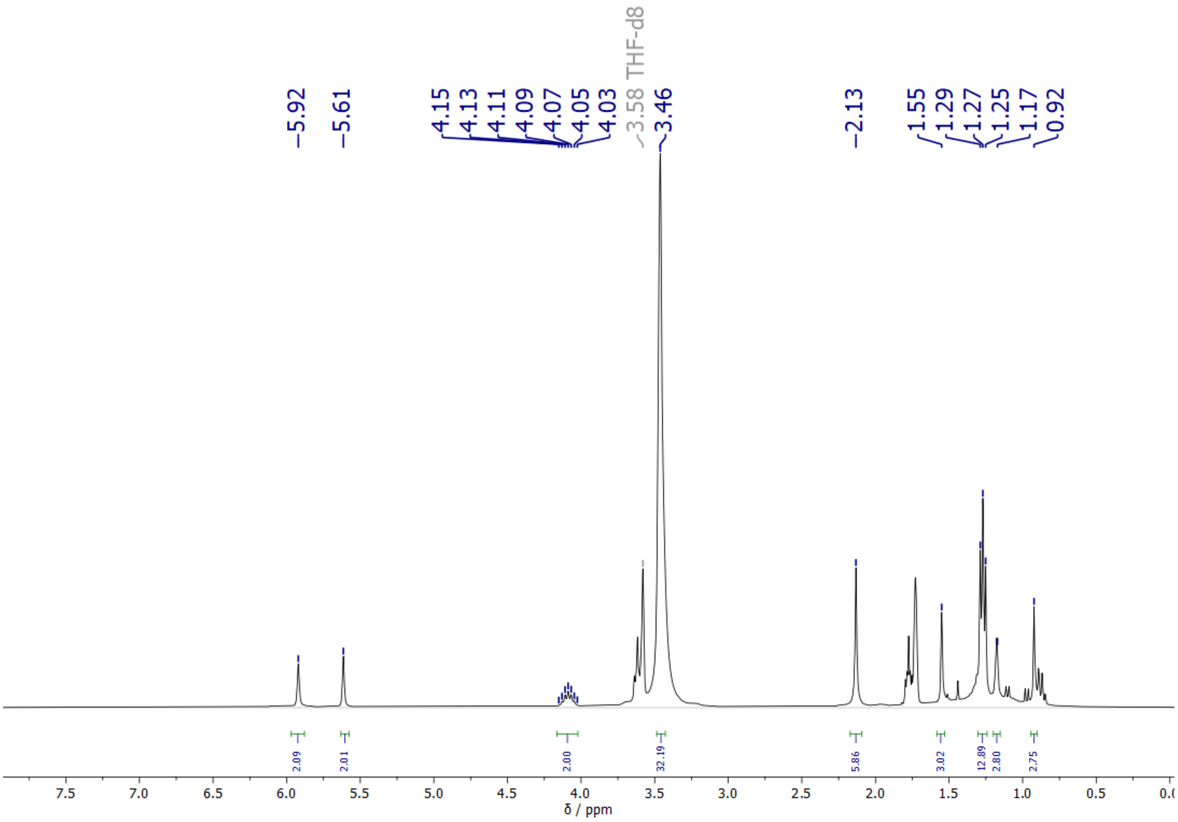

Figure S52. ^{1}H NMR spectrum of **5**, THF-d_8, 25 °C; minor unidentified impurities are observed in the aliphatic region due to rapid decomposition.

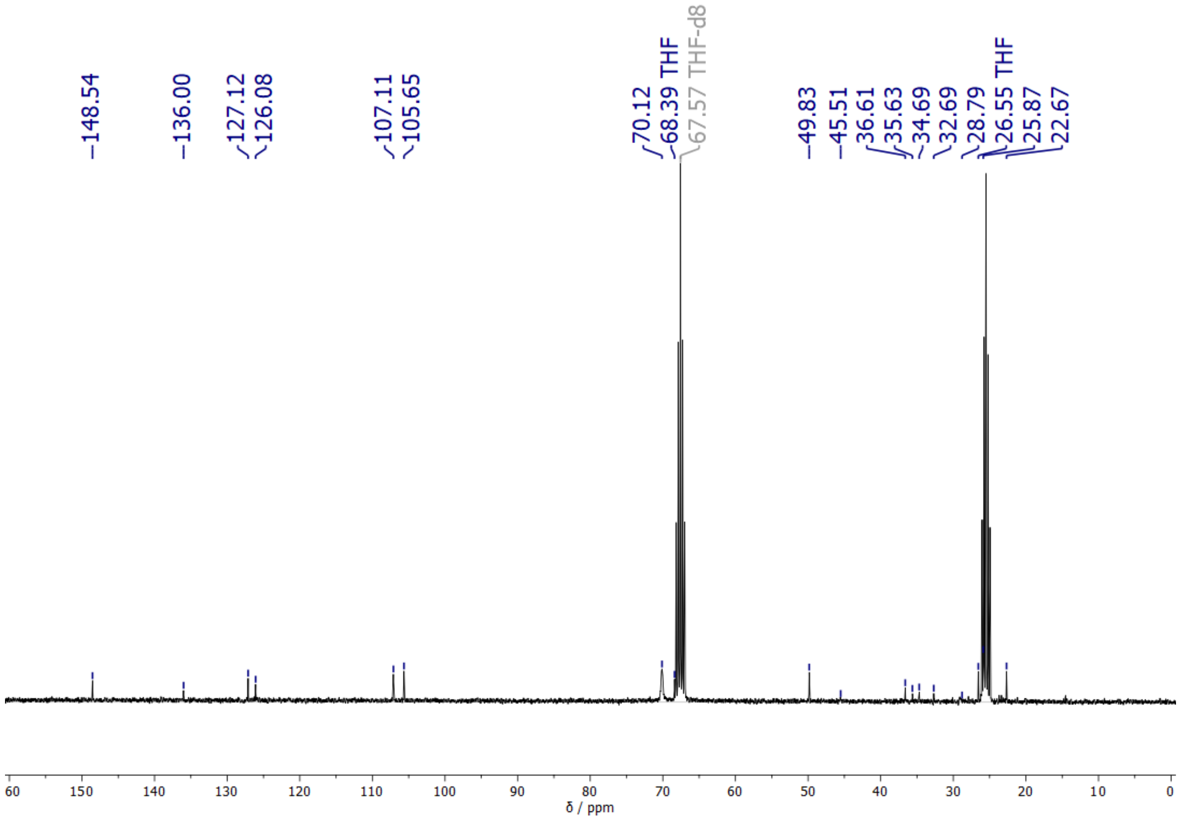

Figure S53. $^{13}C\{^{1}H\}$ NMR spectrum of **5**, THF-d_8, 25 °C; Unidentified impurities stemming from decomposition are observed at δ_C = 35.63, 32.69 ppm.

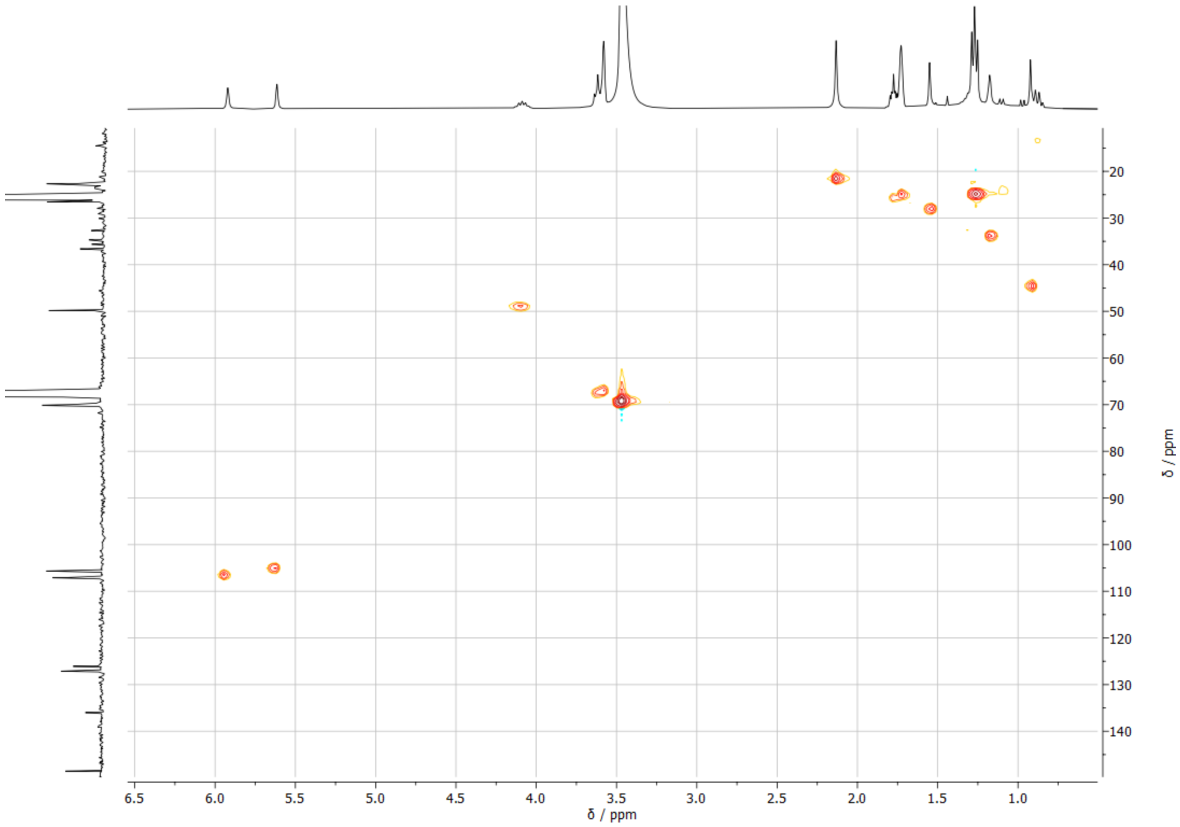

Figure S54. HSQC NMR spectrum of **5**, THF-d_8, 25 °C.

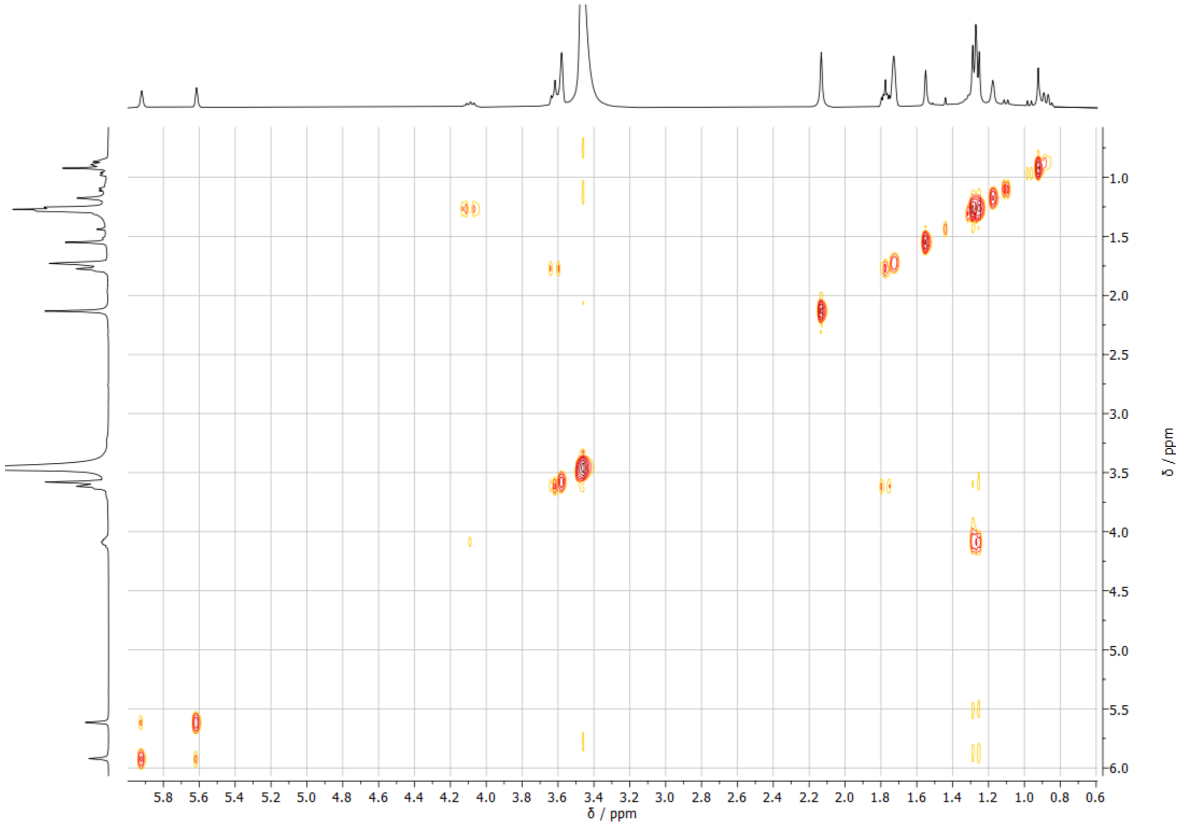

Figure S55. COSY NMR spectrum of **5**, THF-d_8, 25 °C.

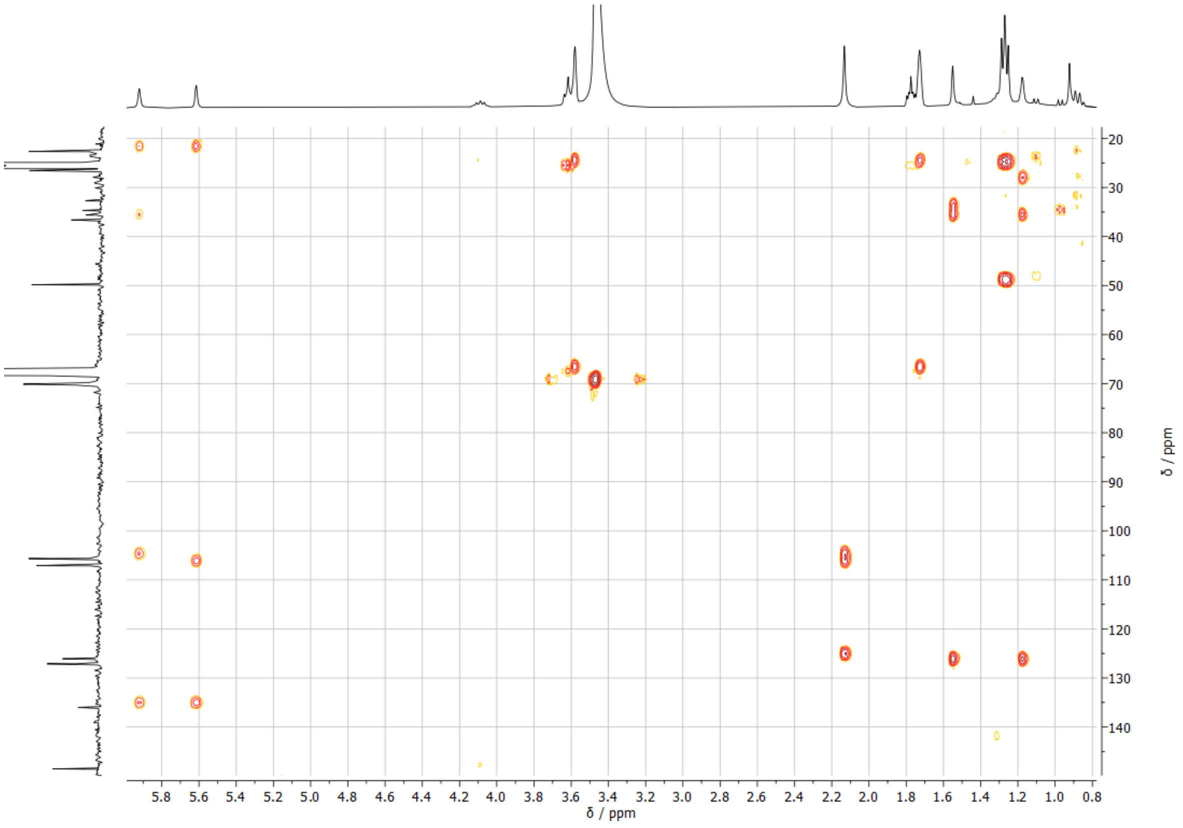

Figure S56. HMBC NMR spectrum of **5**, THF-d_8, 25 °C.

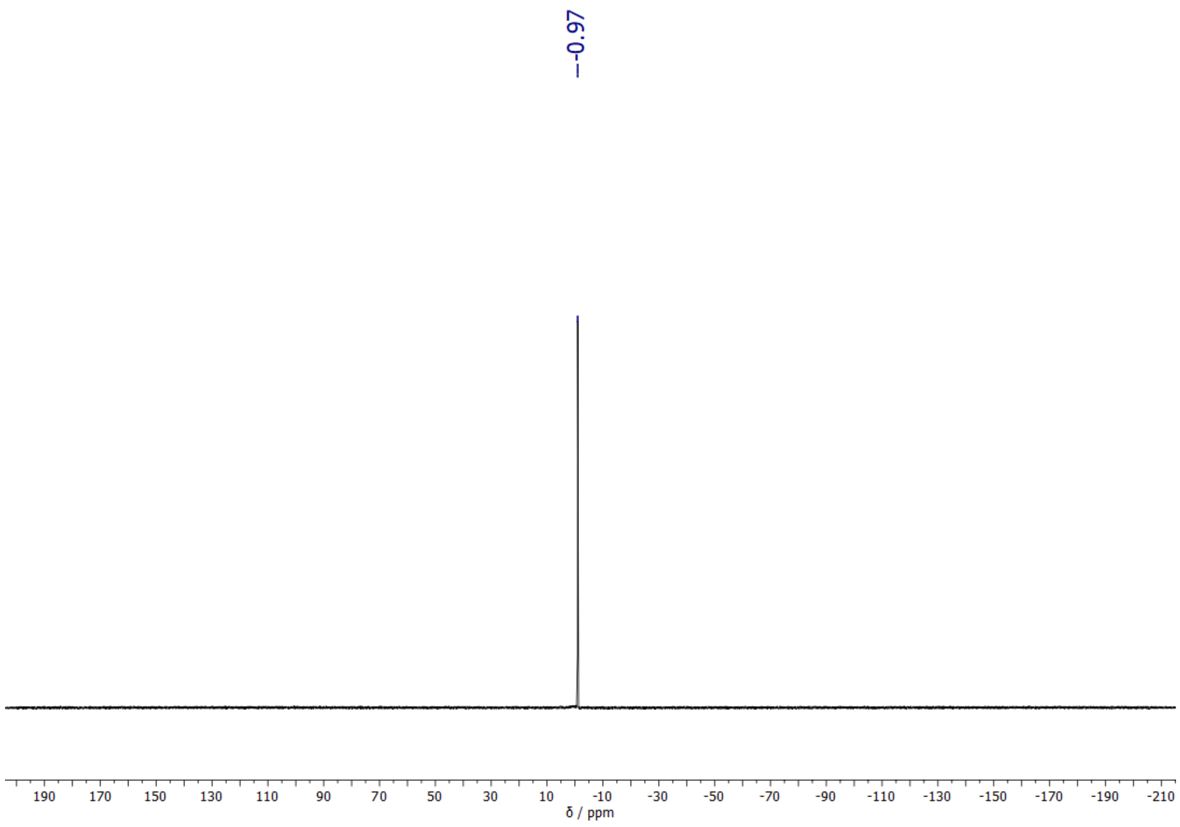

Figure S57. $^{7}Li\{^{1}H\}$ NMR spectrum of **5**, THF-d_8, 25 °C.

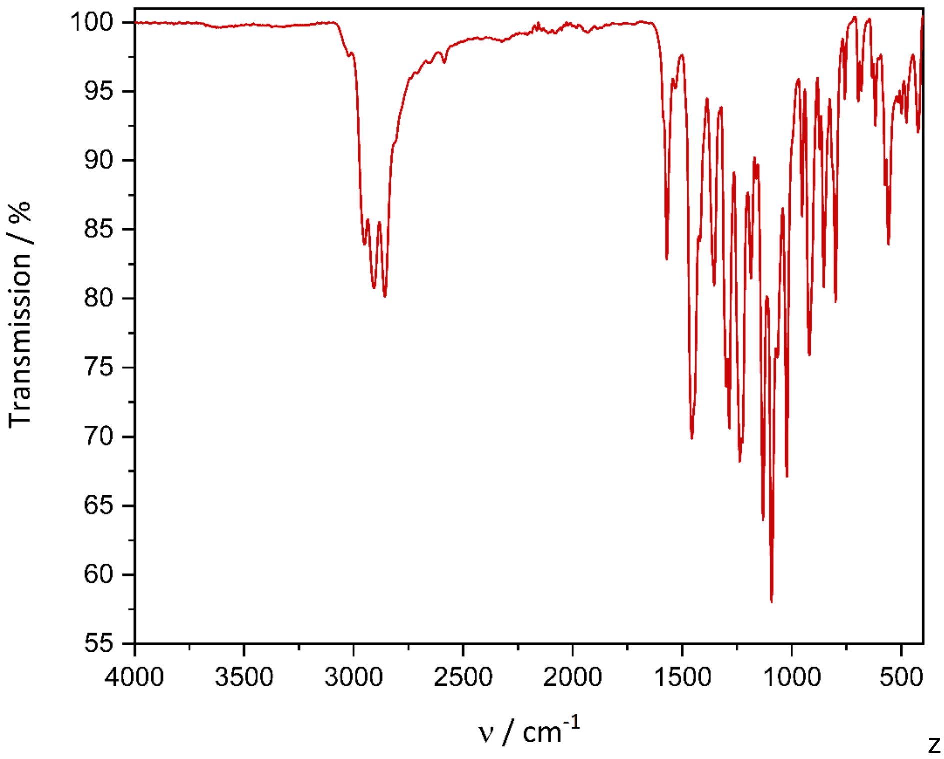

z

Figure S58. ATR-IR spectrum of **5**, solid, 25 °C.

NMR and IR Spectroscopy of 6

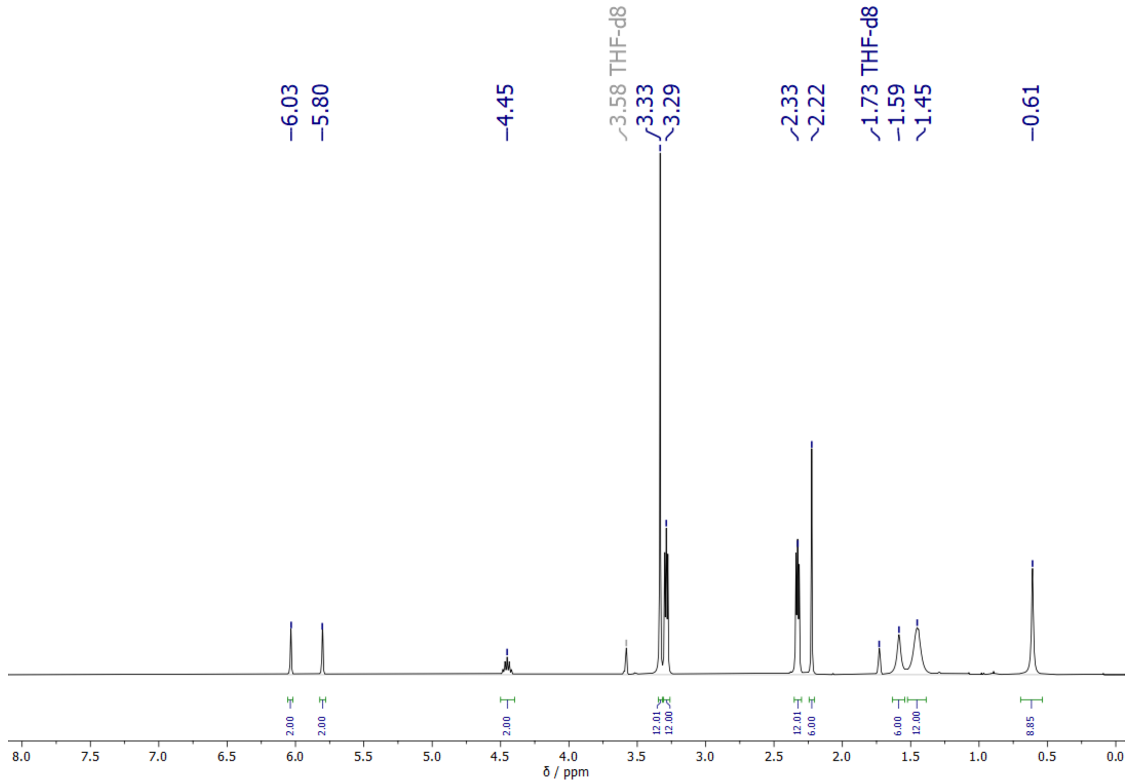

Figure S59. ^{1}H NMR spectrum of **6**, THF-d_8, 25 °C; signal broadening is observed in the aliphatic region.

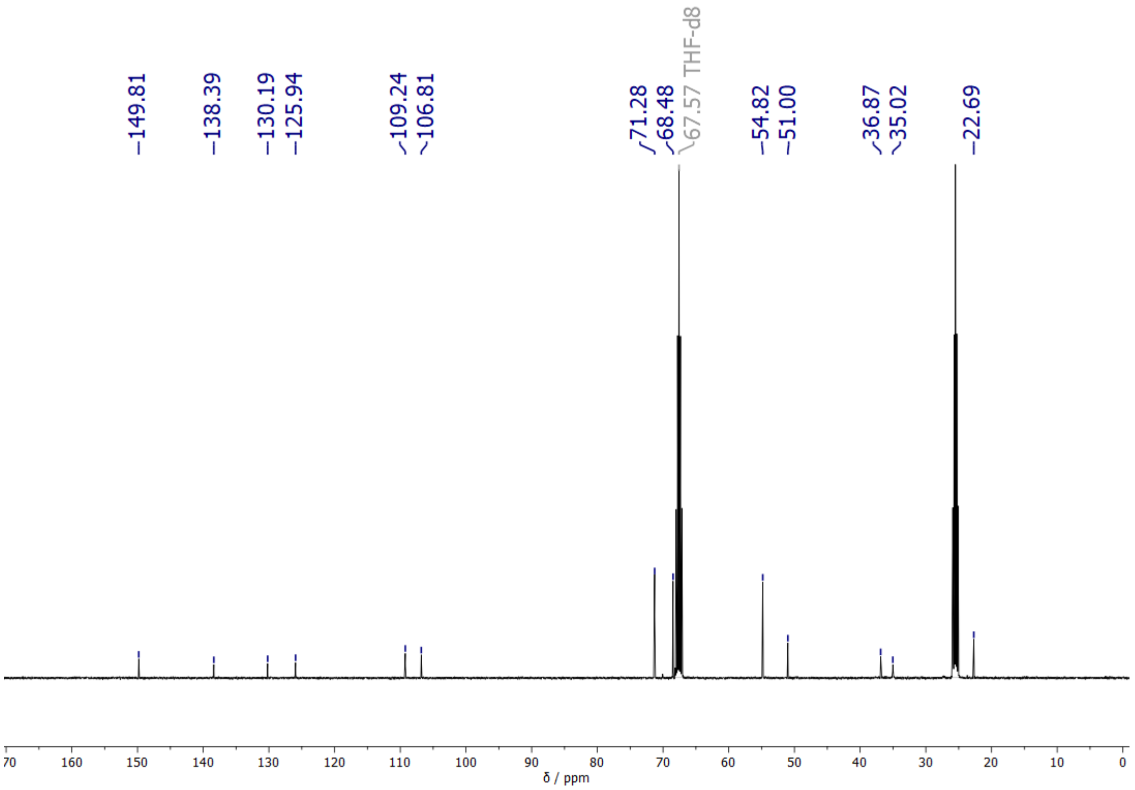

Figure S60. $^{13}C\{^1H\}$ NMR spectrum of **6**, THF-d_8, 25 °C.

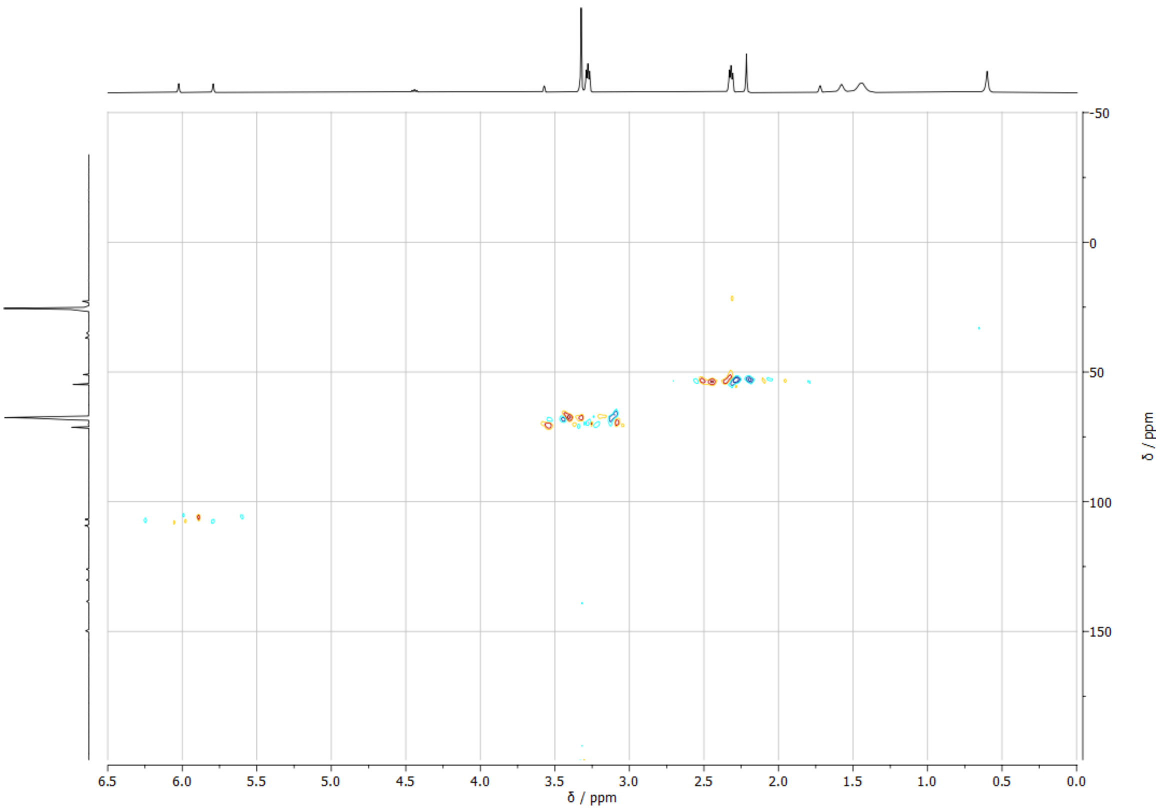

Figure S61. HSQC NMR spectrum of **6**, THF-d_8, 25 °C.

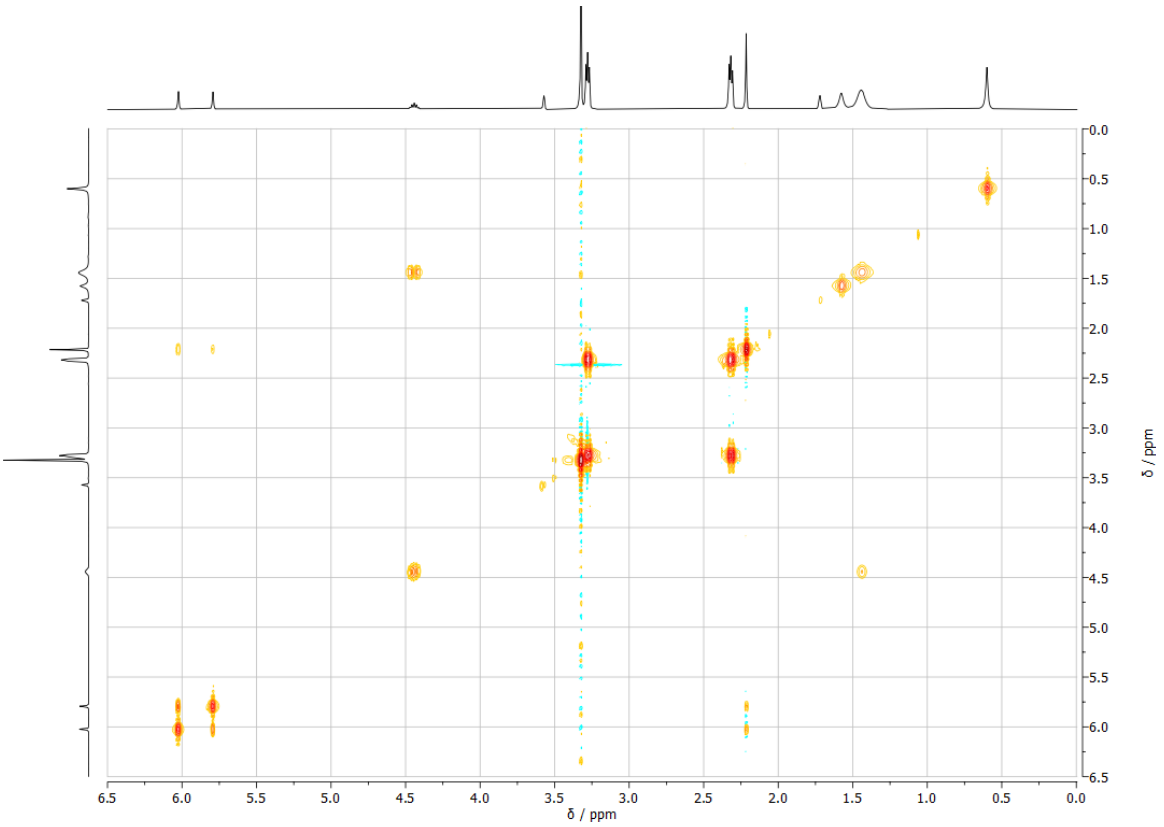

Figure S62. COSY NMR spectrum of **6**, THF-d_8, 25 °C.

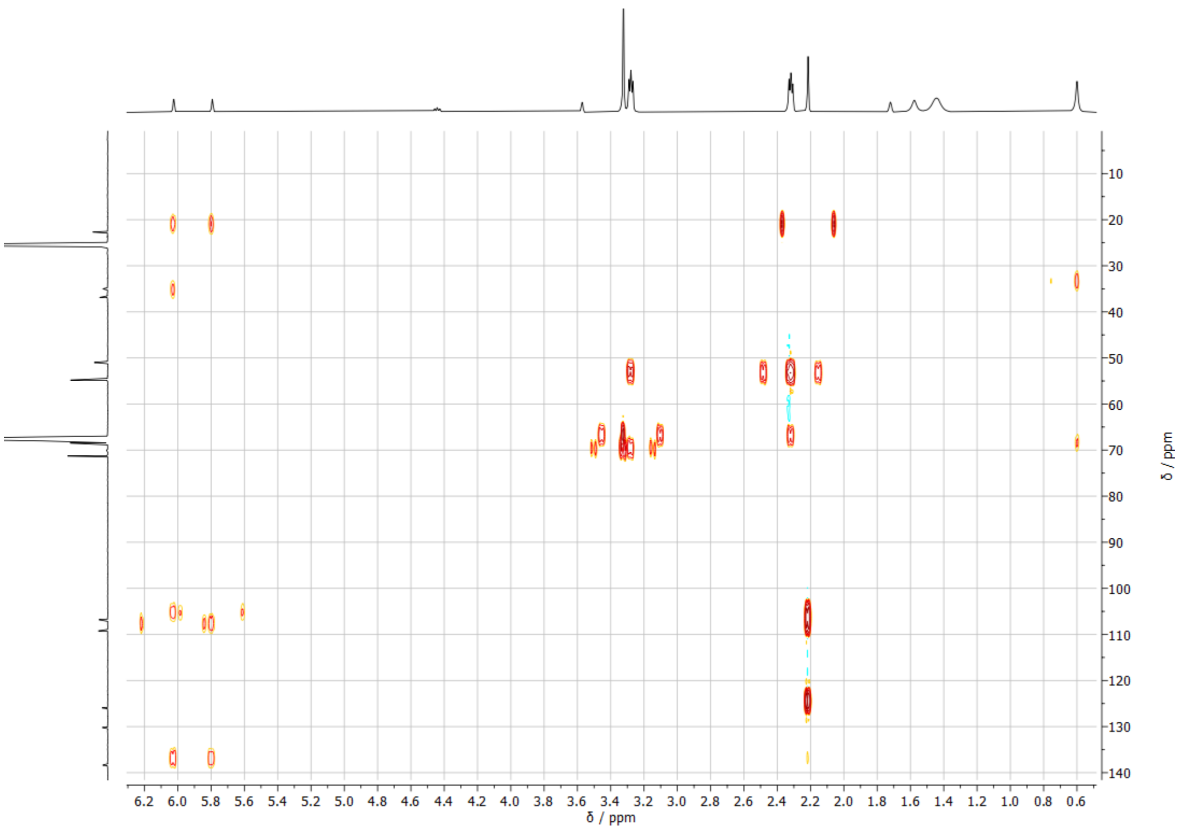

Figure S63. HMBC NMR spectrum of **6**, THF-d₈, 25 °C.

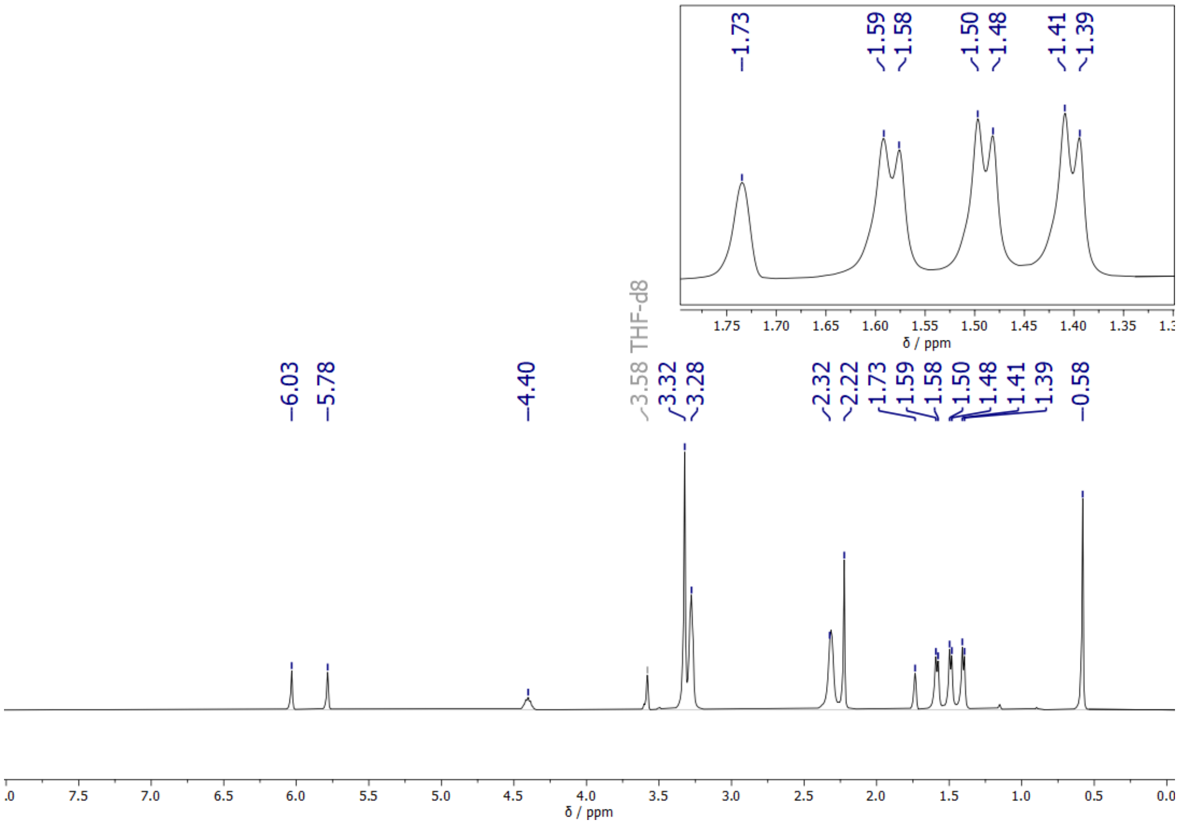

Figure S64. ^{1}H NMR spectrum of **6**, THF-d₈, –27 °C; inset: aliphatic region seeing the splitting of the broad signals at lower temperatures.

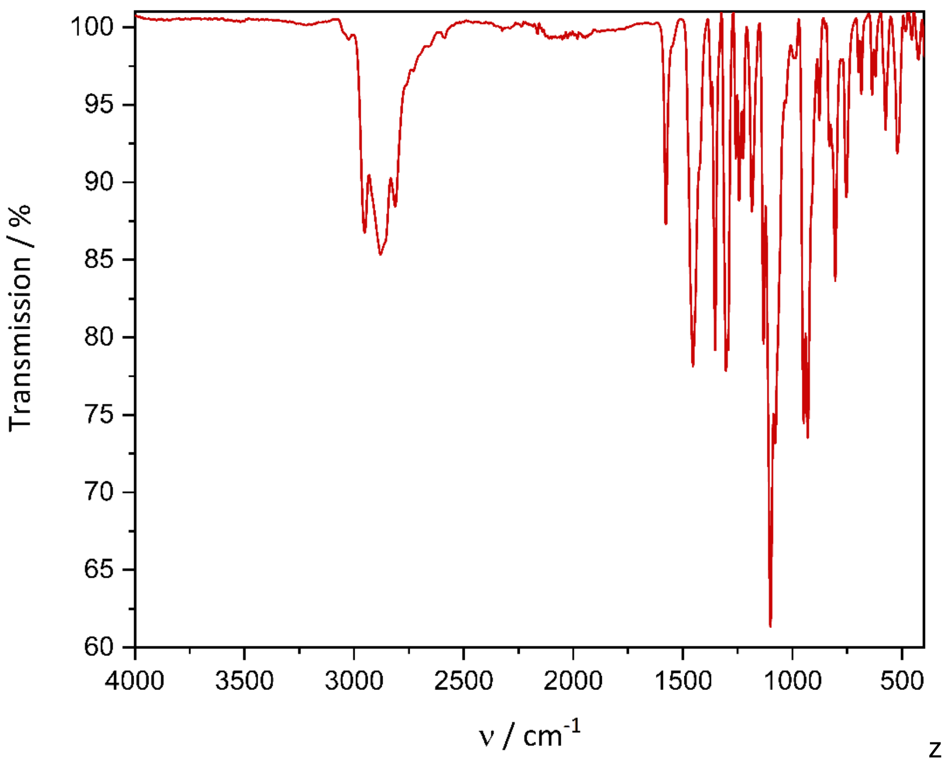

Figure S65. ATR-IR spectrum of **6**, solid, 25 °C.

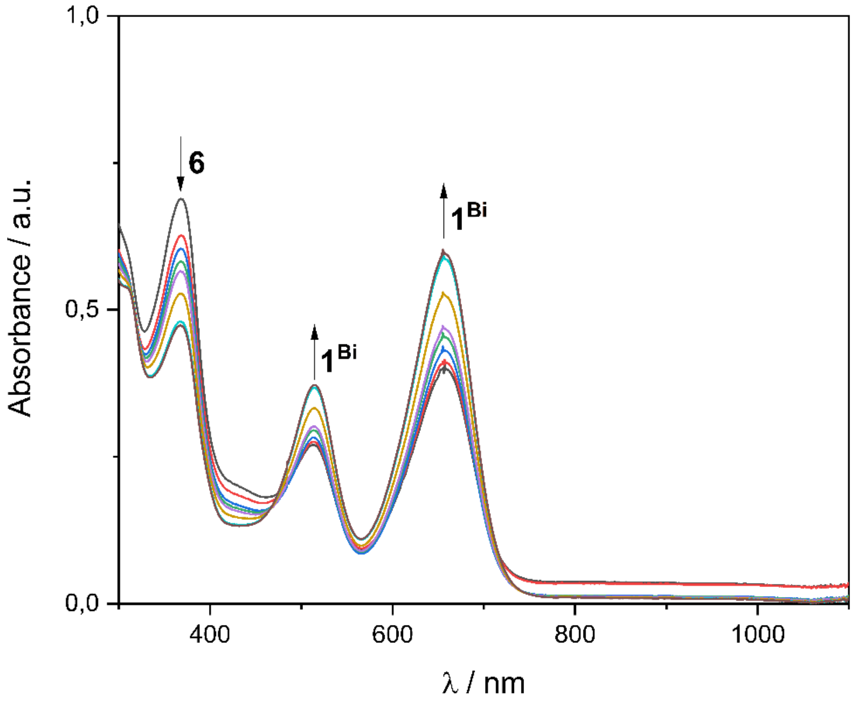

Figure S66. UV/Vis spectrum of **6**, THF, 25 °C. Spectral changes measured over the course of 1 h. The formation of $\mathbf{1}^{Bi}$ is indicated by increasing absorption bands at 657 nm and 519 nm, while the decay of **6** is evident from the decreasing band at 368 nm. The rapid degradation of 6 is attributed to its high sensitivity to moisture.

Spectroscopic Data for the Me^+, $Me^•$, and $Me^−$ Transfer Reactivity

Spectroscopy of Me^+ Transfer from 3^{BArF} to $P(^tBu_3)_3$

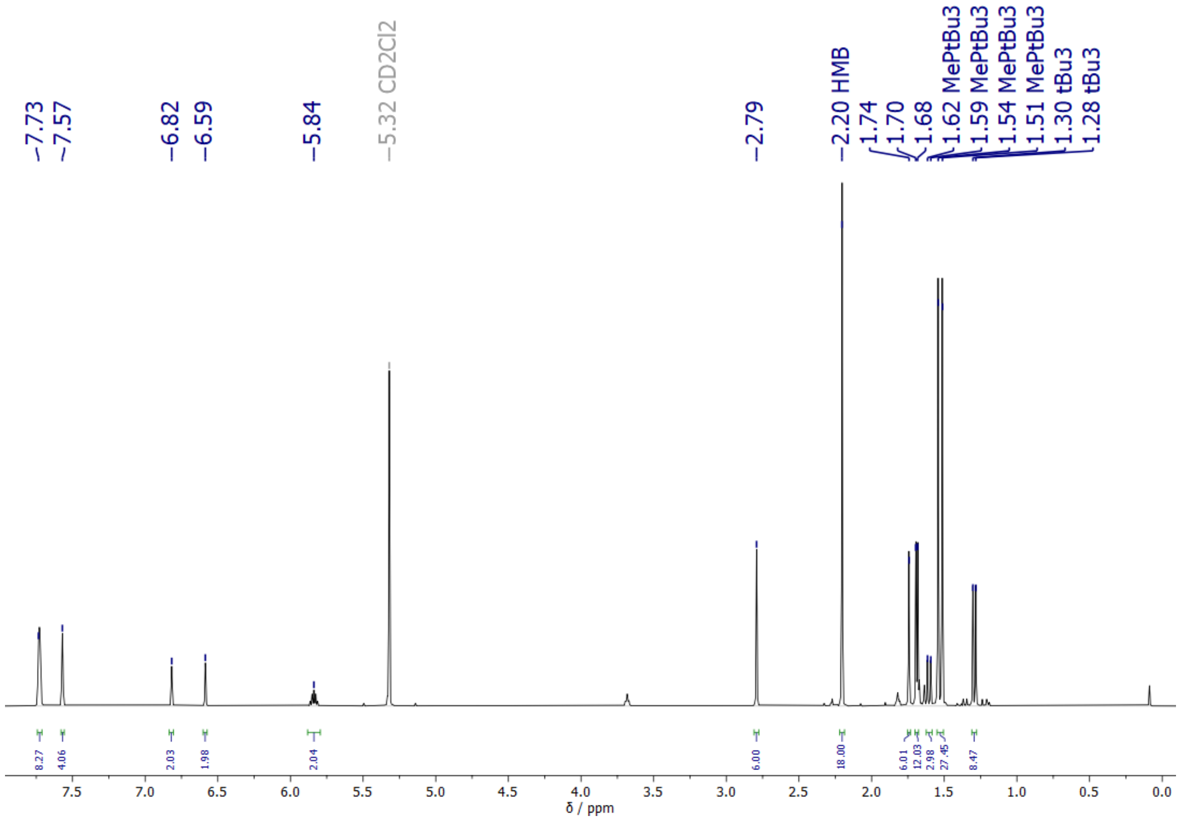

Figure S67. ^{1}H NMR spectrum of the reaction solution of $\mathbf{3^{BArF}}$ and $P(^tBu)_3$, CD_2Cl_2 25 °C, hexamethylbenzene (HMB) as internal standard.

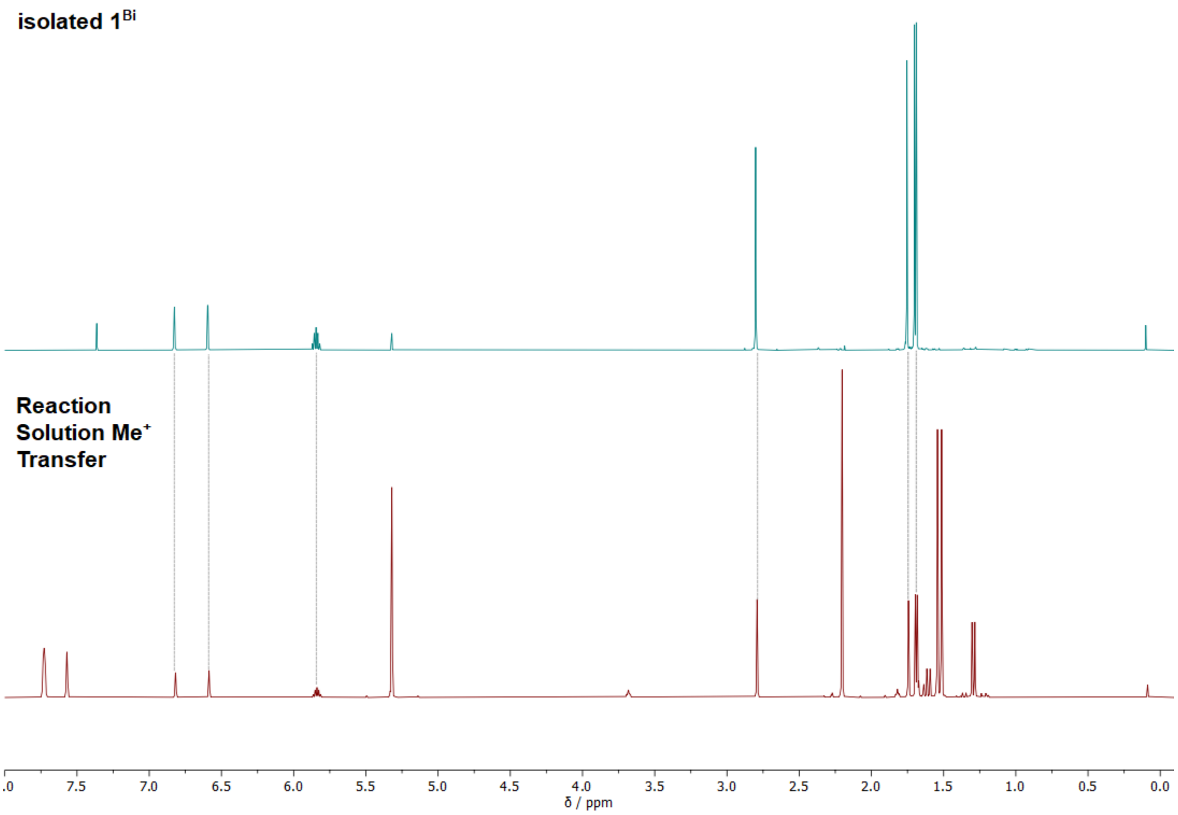

Figure S68. ^{1}H NMR spectra of isolated **1^{Bi}** and the reaction solution of **3^{BArF}** and P(^{t}Bu)$_3$, CD_2Cl_2, 25 °C.

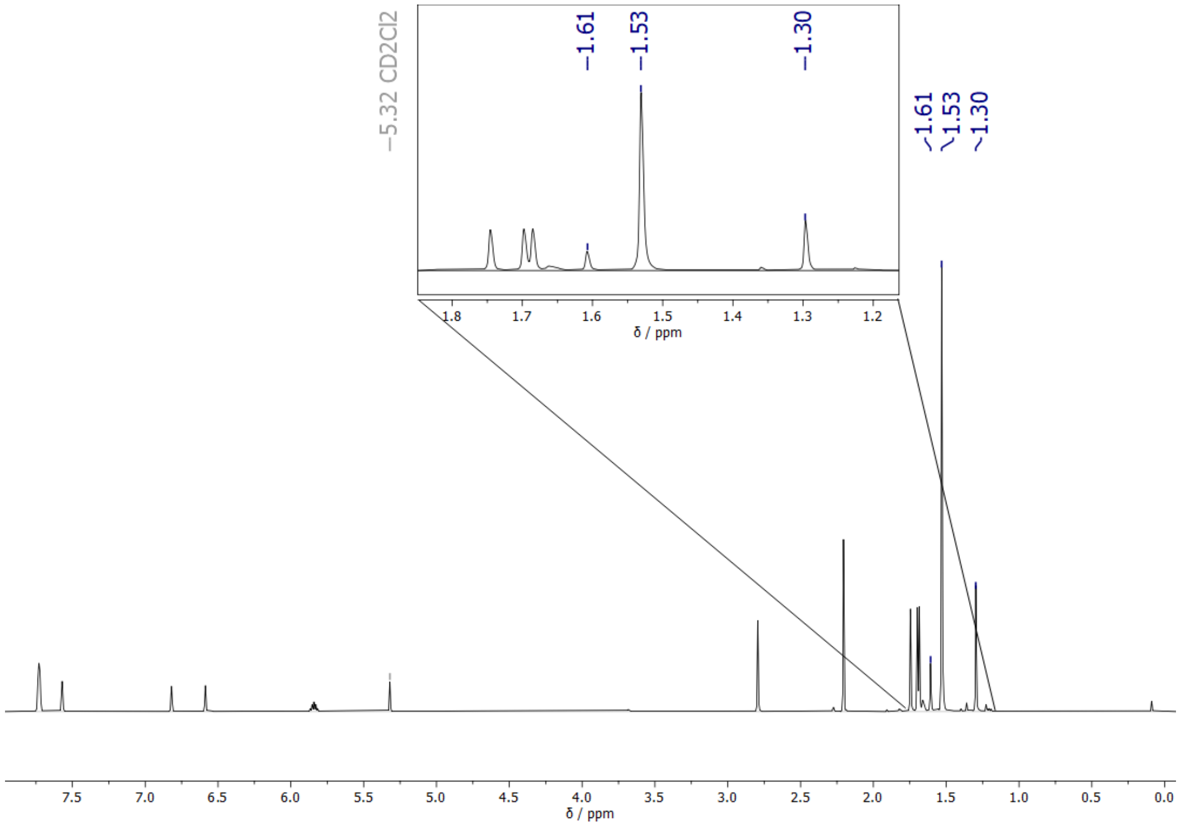

Figure S69. $^{1}H\{^{31}P\}$ NMR spectrum the reaction solution of $\mathbf{3}^{\mathbf{BArF}}$ and $P(^{t}Bu_3)_3$, CD_2Cl_2 25 °C, hexamethylbenzene (HMB) as internal standard; inset: indicative signals sensitive to phosphorus decoupling δ = 1.6 (*Me*P^{t}Bu$_3$), 1.53 (MeP^{t}*Bu$_3$*) and 1.3 (P^{t}Bu$_3$) ppm.

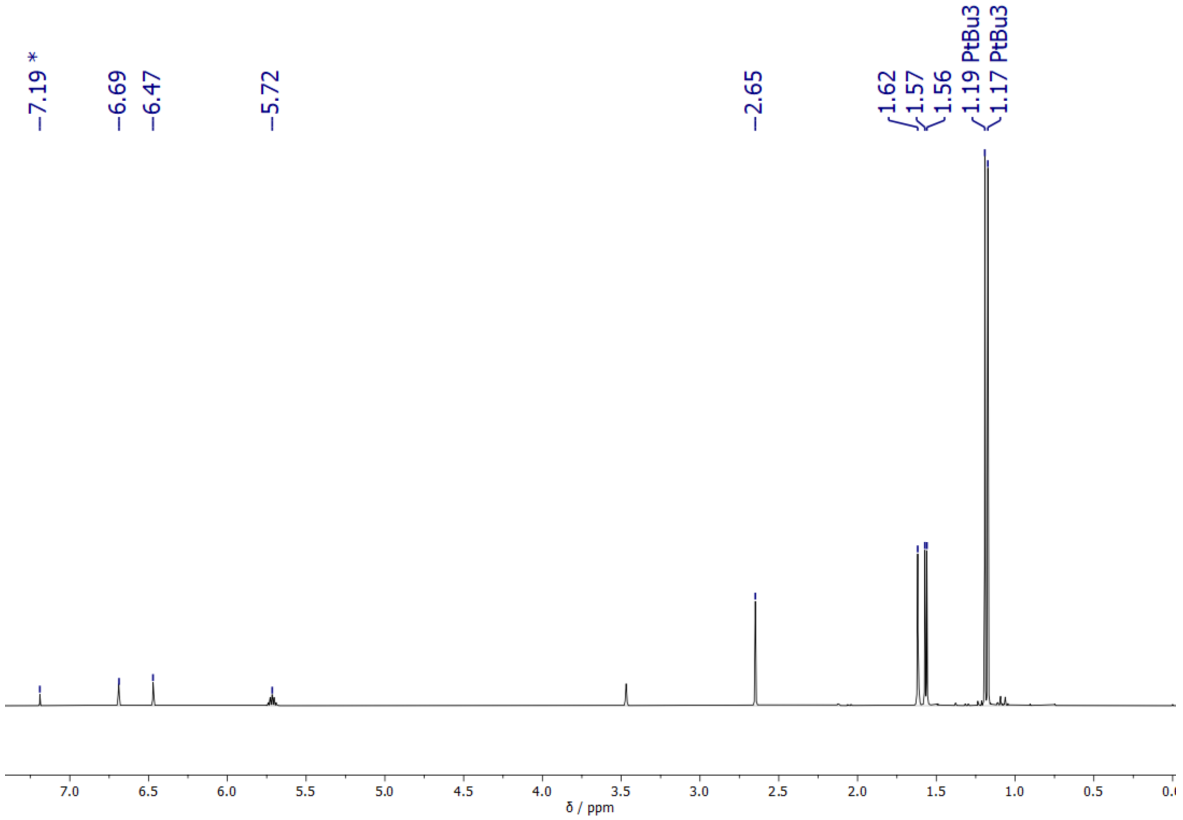

Figure S70. $^1H\{^{31}P\}$ NMR spectrum of **1^{Bi}** in the presence of $P(^tBu)_3$ after 2d at 25 °C, THF-d_8, 25 °C; benzene marked with *.

Spectroscopy of Me• Transfer from 4 to TEMPO

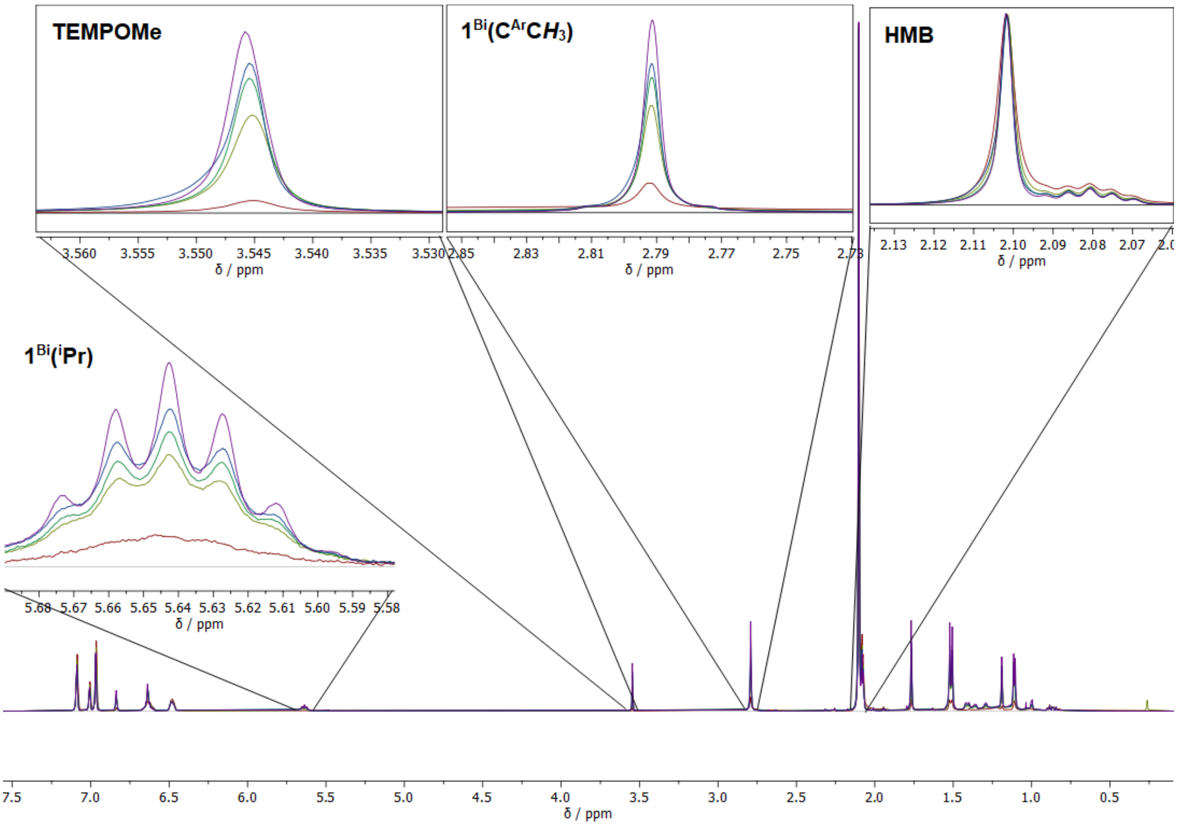

Figure S71. ^{1}H NMR spectrum of the reaction solution of **4** and TEMPO over time (t = 10 min, 1 h, 2 h, 3 h, 24 h (red to violet)), tol-d_8, 25 °C, hexamethylbenzene (HMB) as internal standard; inset of separated signals used for integration to determine spectroscopic yield.

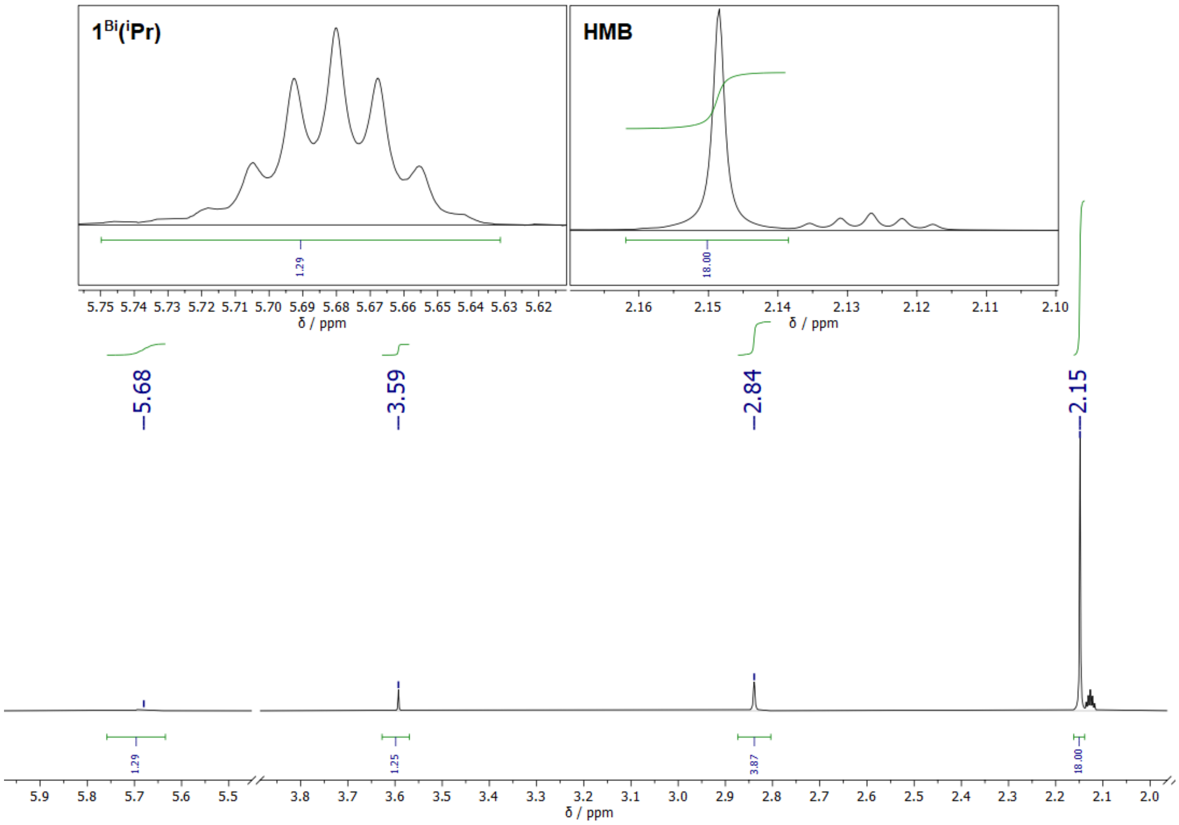

Figure S72. q^{1}H NMR spectrum of the reaction solution of **4** and TEMPO after 24 h, tol-d$_8$, 25 °C, hexamethylbenzene (HMB) as internal standard; inset magnification to clarify integration.

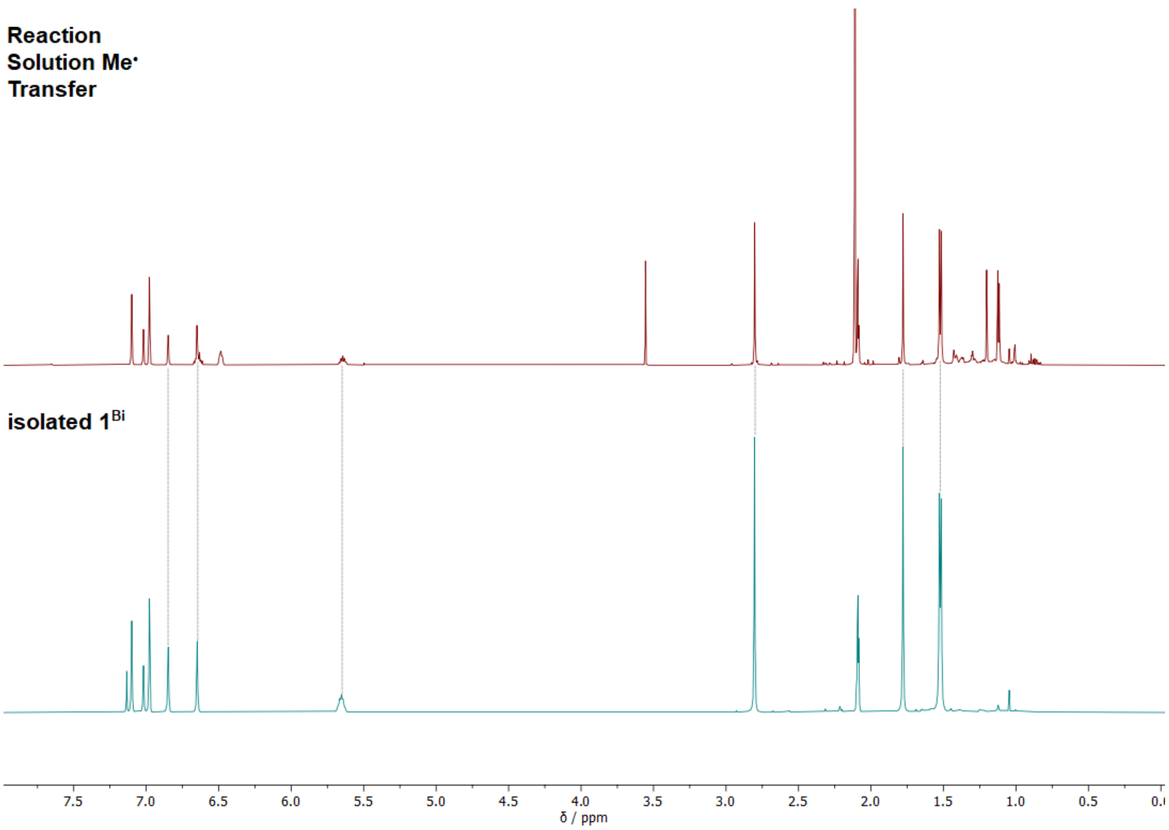

Figure S73. ^{1}H NMR spectra of the reaction solution of **4** and TEMPO and isolated **1^{Bi}**, tol-d_8 25 °C.

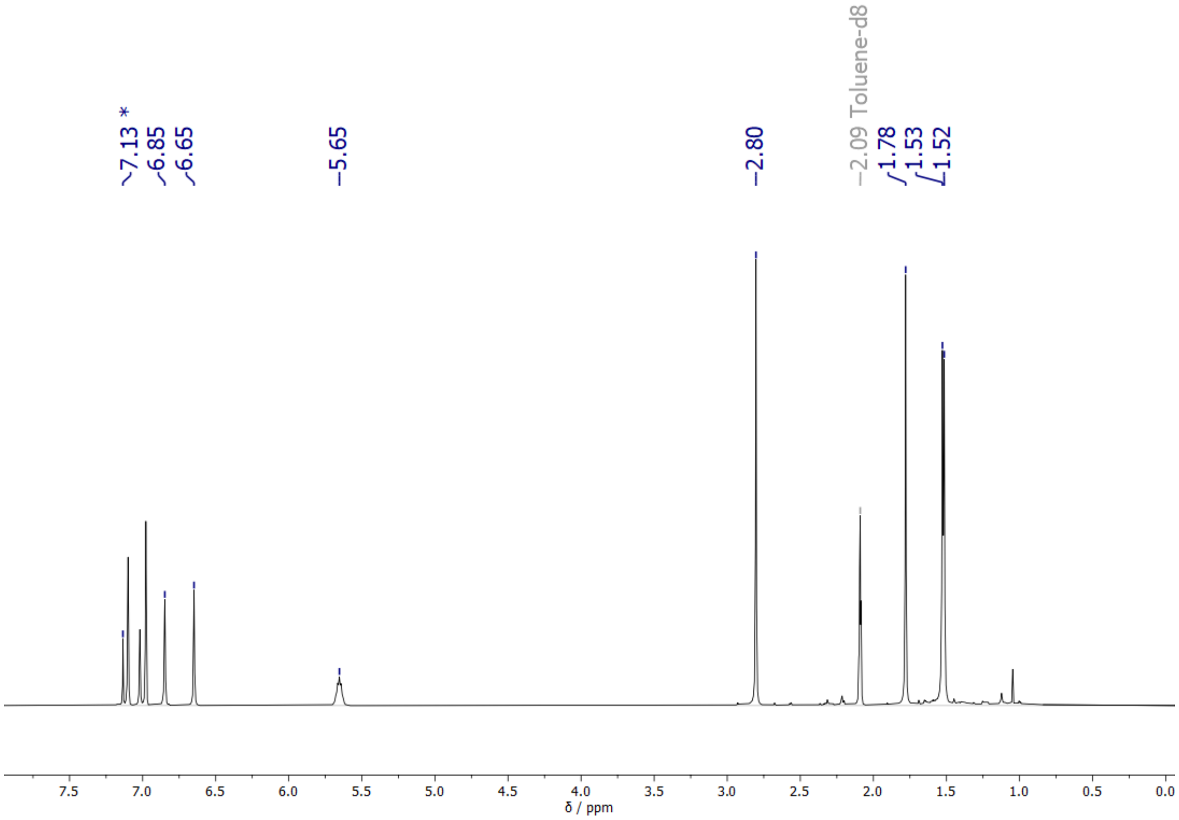

Figure S74. ^{1}H NMR spectrum of **1^{Bi}** in the presence of TEMPO after 2d at 40 °C, tol-d_8, 25 °C; benzene marked with *.

Spectroscopy of Me⁻ Transfer from 5 to $B(C_6F_5)_3$

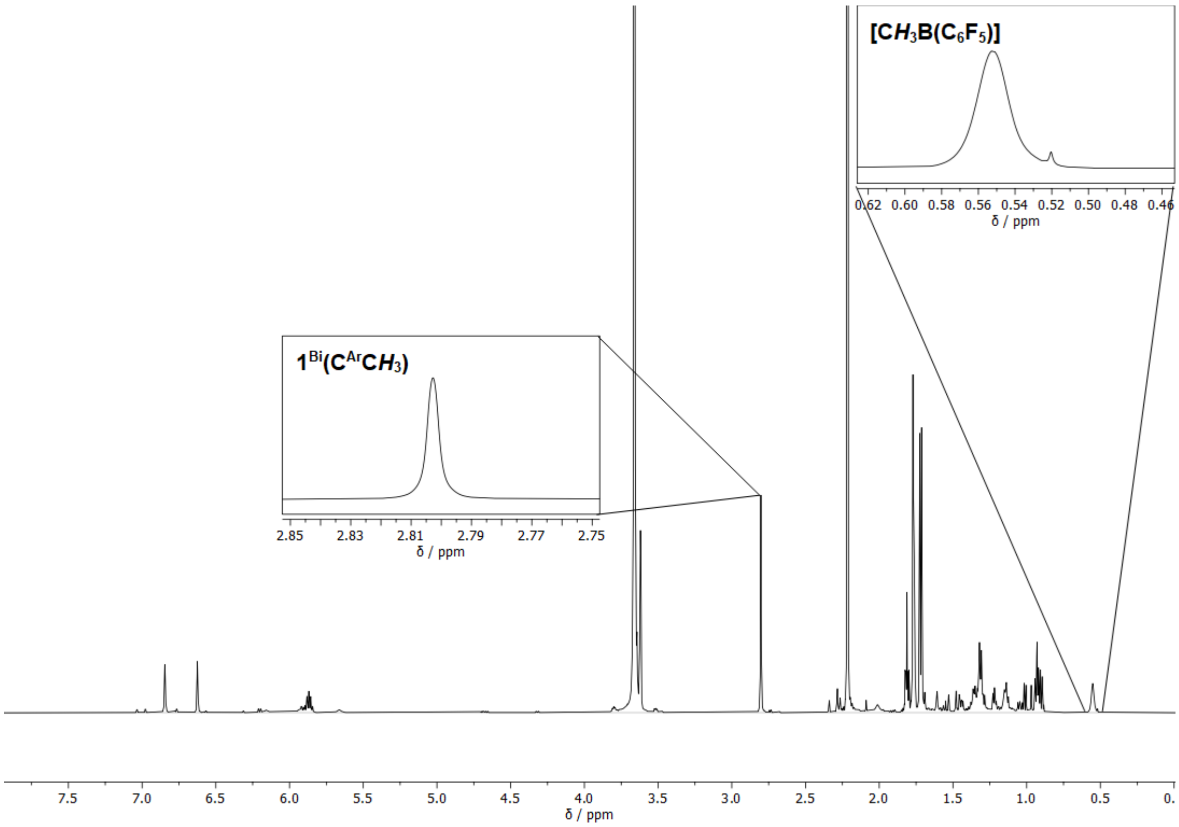

Figure S75. ^{1}H NMR spectrum of the reaction solution of **5** and $B(C_6F_5)_3$, THF-d_8, 25 °C; inset: signals used for yield determination.

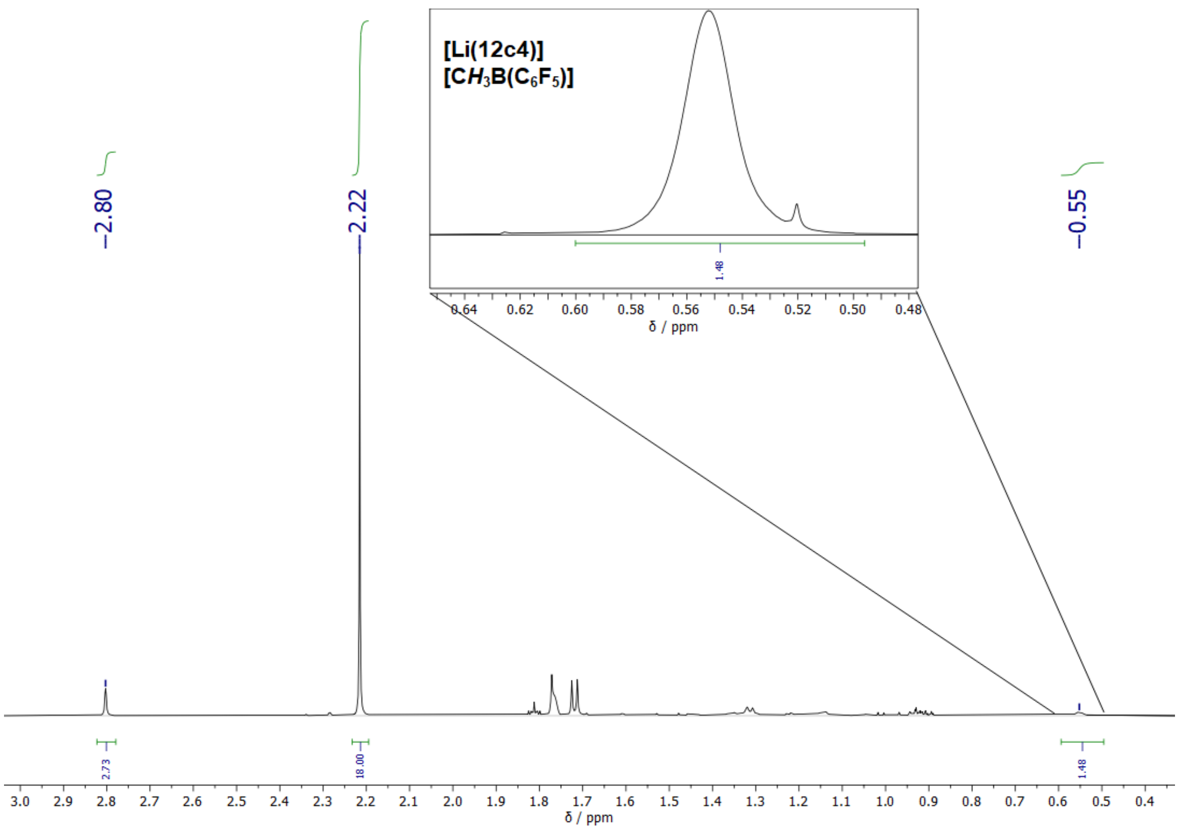

Figure S76. ^{1}H NMR spectrum of the reaction solution of **5** and $B(C_6F_5)_3$, THF-d_8, 25 °C, HMB as internal standard; inset: signal associated with [$\mathit{Me}B(C_6F_5)_3$]$^-$.

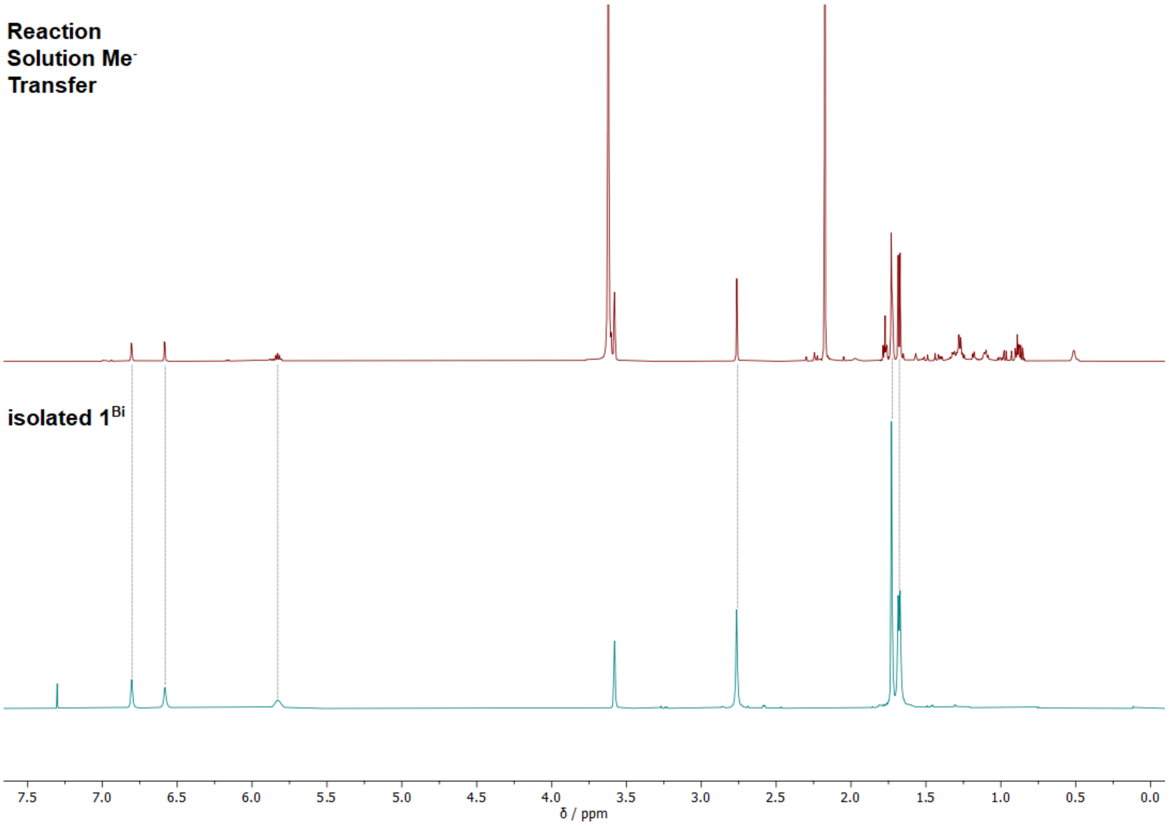

Figure S77. ^{1}H NMR spectra of the reaction solution of **5** and $B(C_6F_5)_3$ and isolated $\mathbf{1^{Bi}}$, THF-d_8, 25 °C.

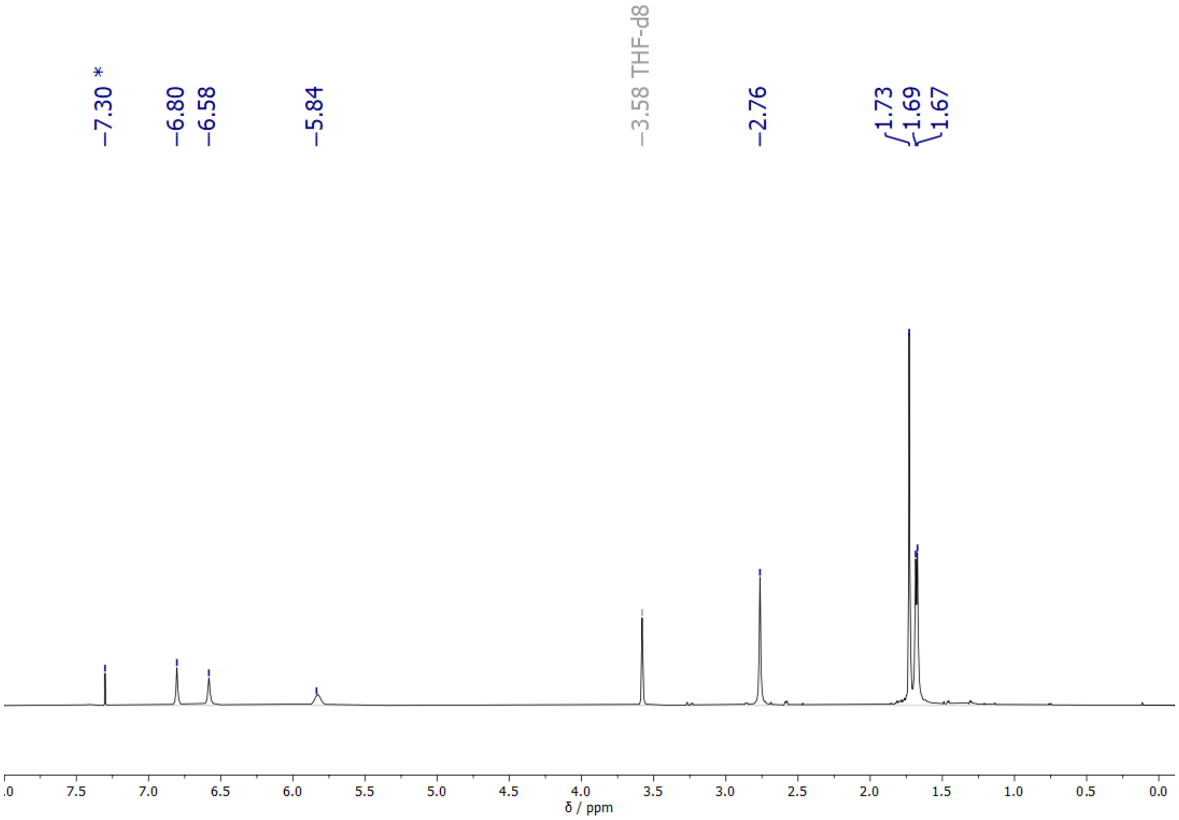

Figure S78. ^{1}H NMR spectrum of **1**^{Bi} and $B(C_6F_5)_3$ after 2 d at 25 °C, THF-d_8, 25 °C. benzene marked with *.

Spectroscopic Data for Additional Reactions

Spectroscopy of 1[Bi] with Water

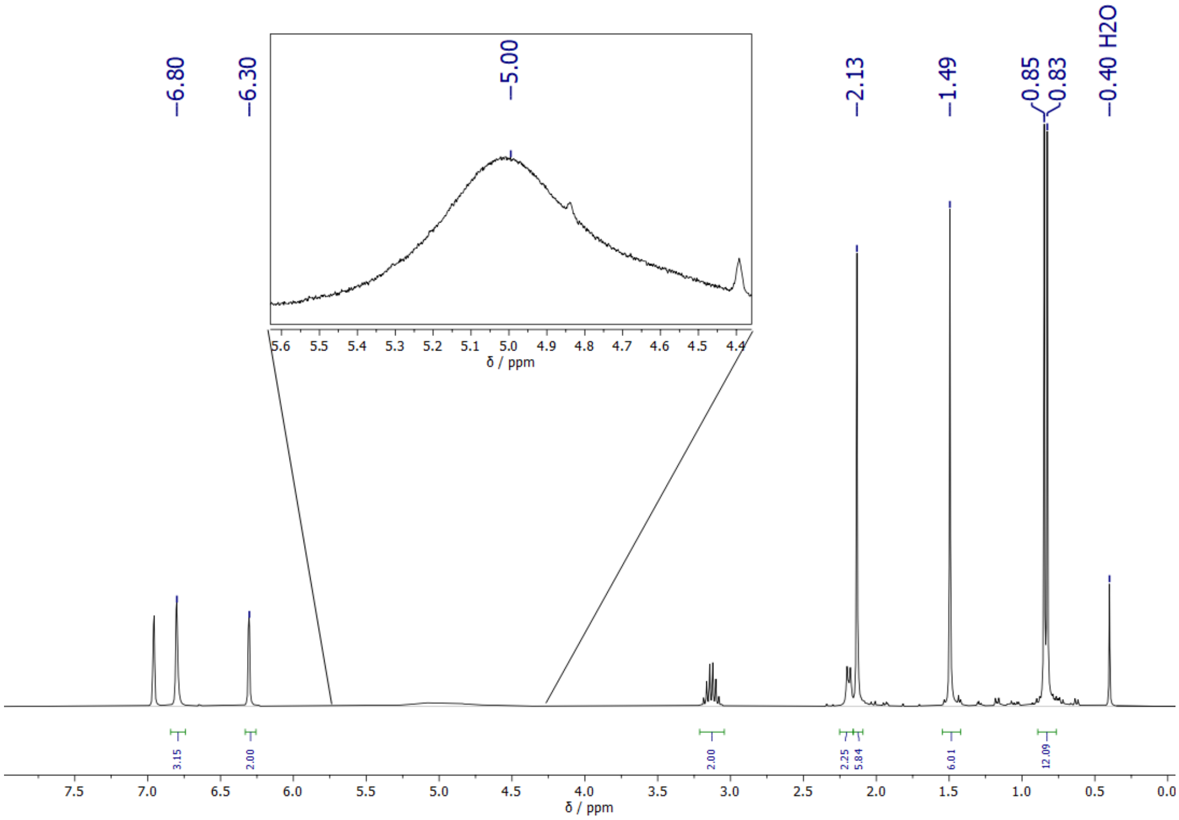

Figure S79. ^{1}H NMR spectrum of the reaction solution of **1**^{Bi} and water, C_6D_6, 25 °C; inset: magnification of the $Bi(OH)_3$ signal.

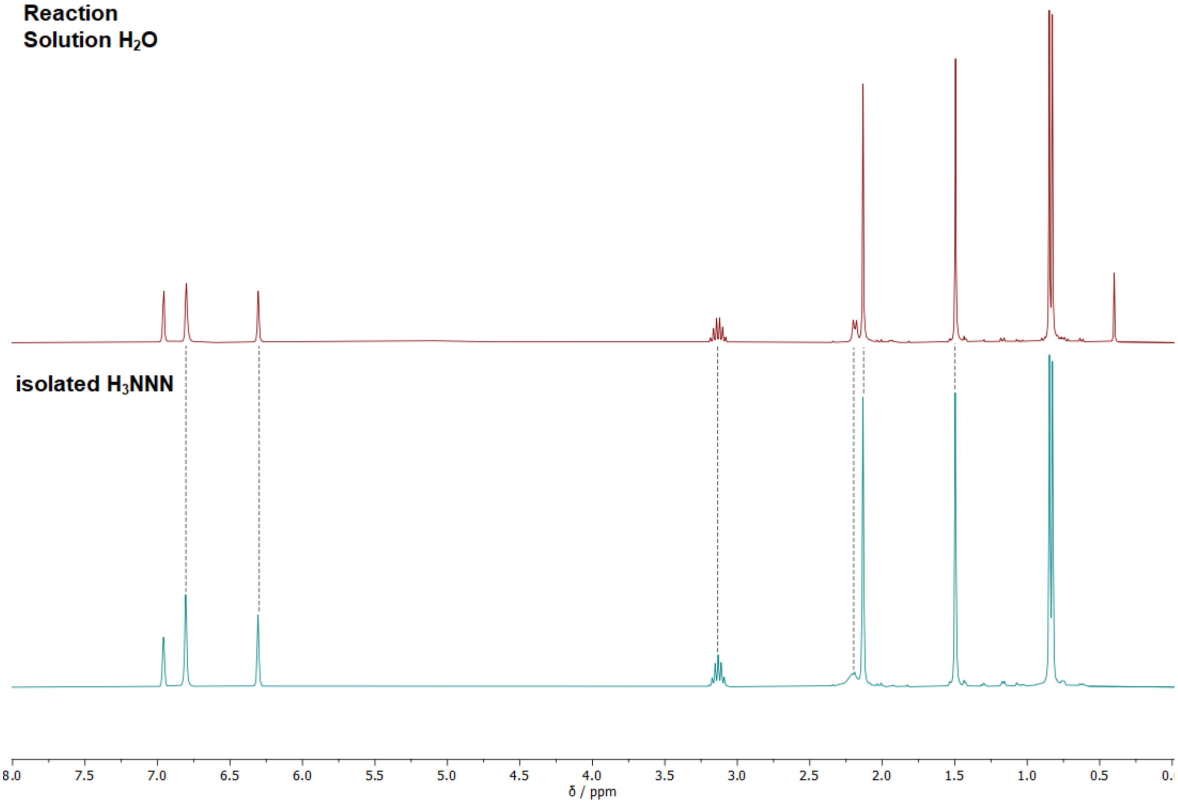

Figure S80. ^{1}H NMR spectrum of $\mathbf{1^{Bi}}$ and water and isolated $\mathbf{H_3NNN}$, C_6D_6, 25 °C.

Spectroscopy of 1^Bi with Diphenyl Diselenide

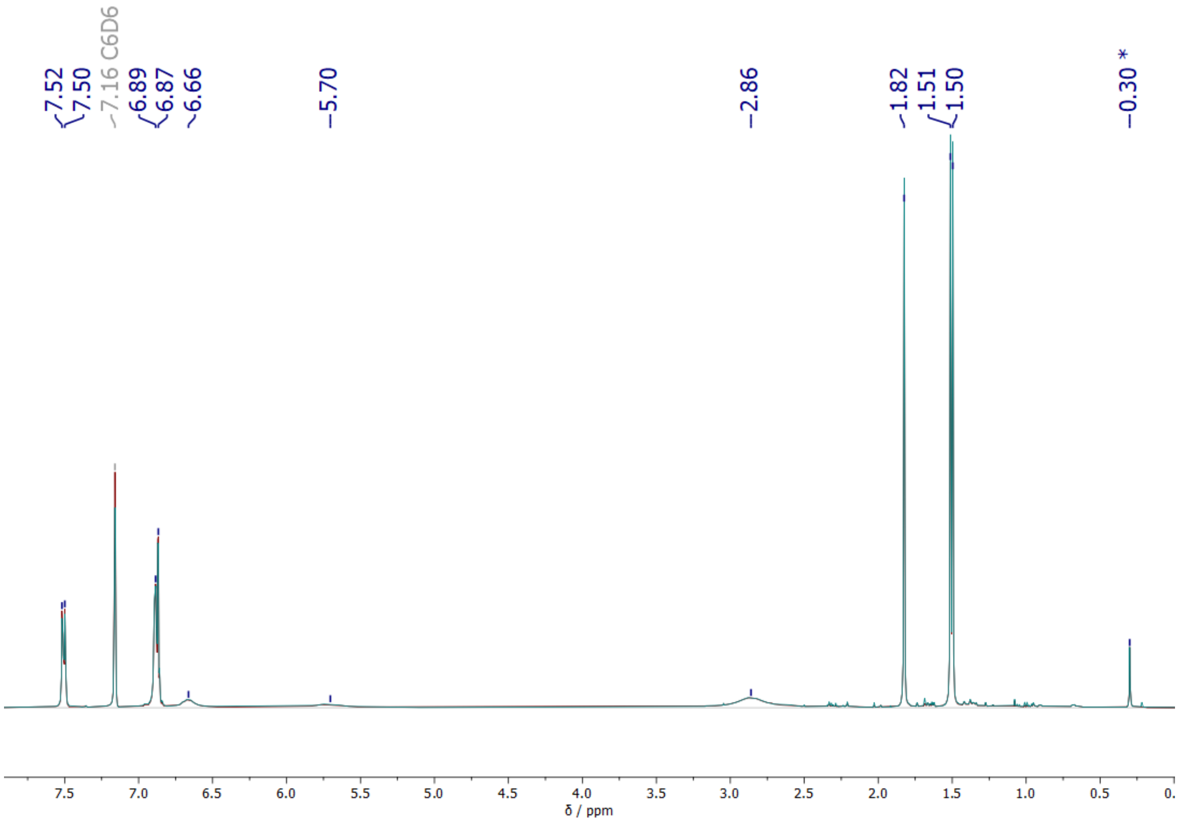

Figure S81. ^{1}H NMR spectrum of the reaction solution of **1^{Bi}** and Ph_2Se_2, C_6D_6, 25 °C; *red*: after stirring for 5 h at 25 °C, *green*: after heating 15 h at 70 °C. Silicone grease marked with *.

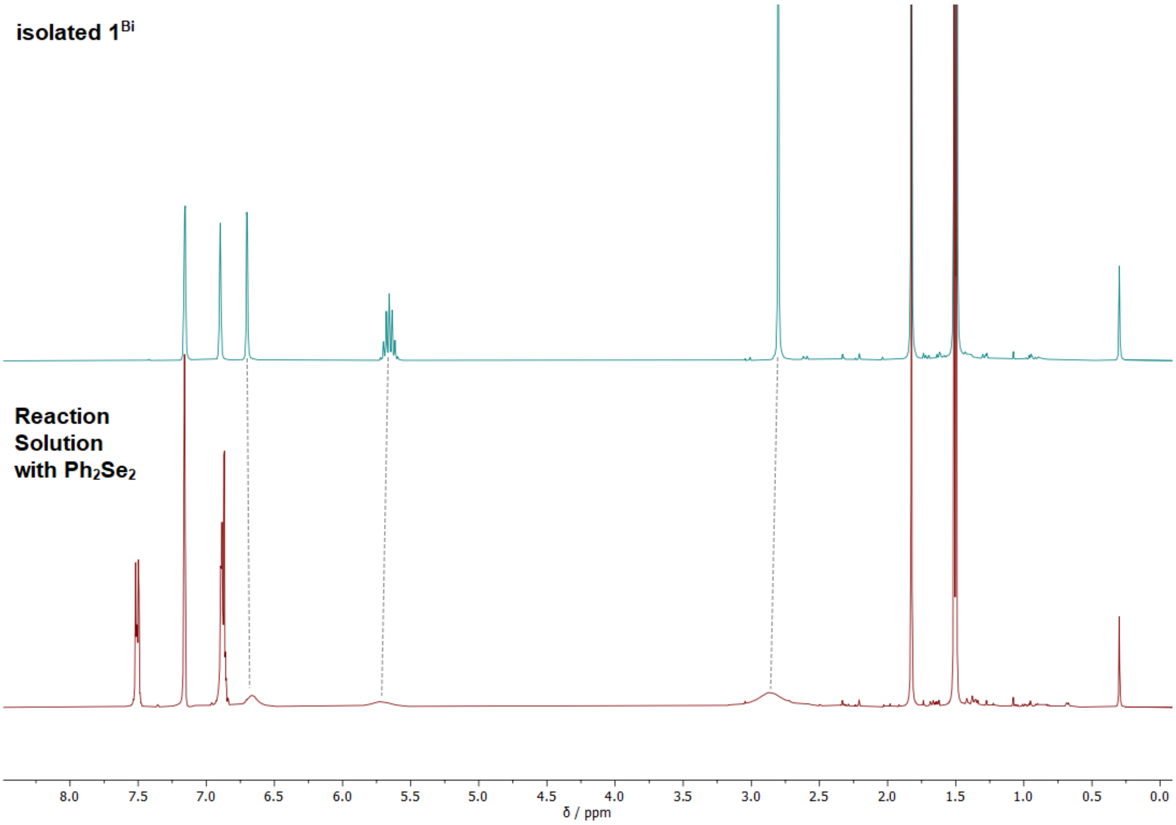

Figure S82. ^{1}H NMR spectrum of isolated $\mathbf{1}^{Bi}$ and the reaction solution containing Ph_2Se_2, C_6D_6, 25 °C; line broadening of the signals associated with $\mathbf{1}^{Bi}$ is visible after addition of Ph_2Se_2.

Spectroscopy of 3^{BArF} with KHMDS

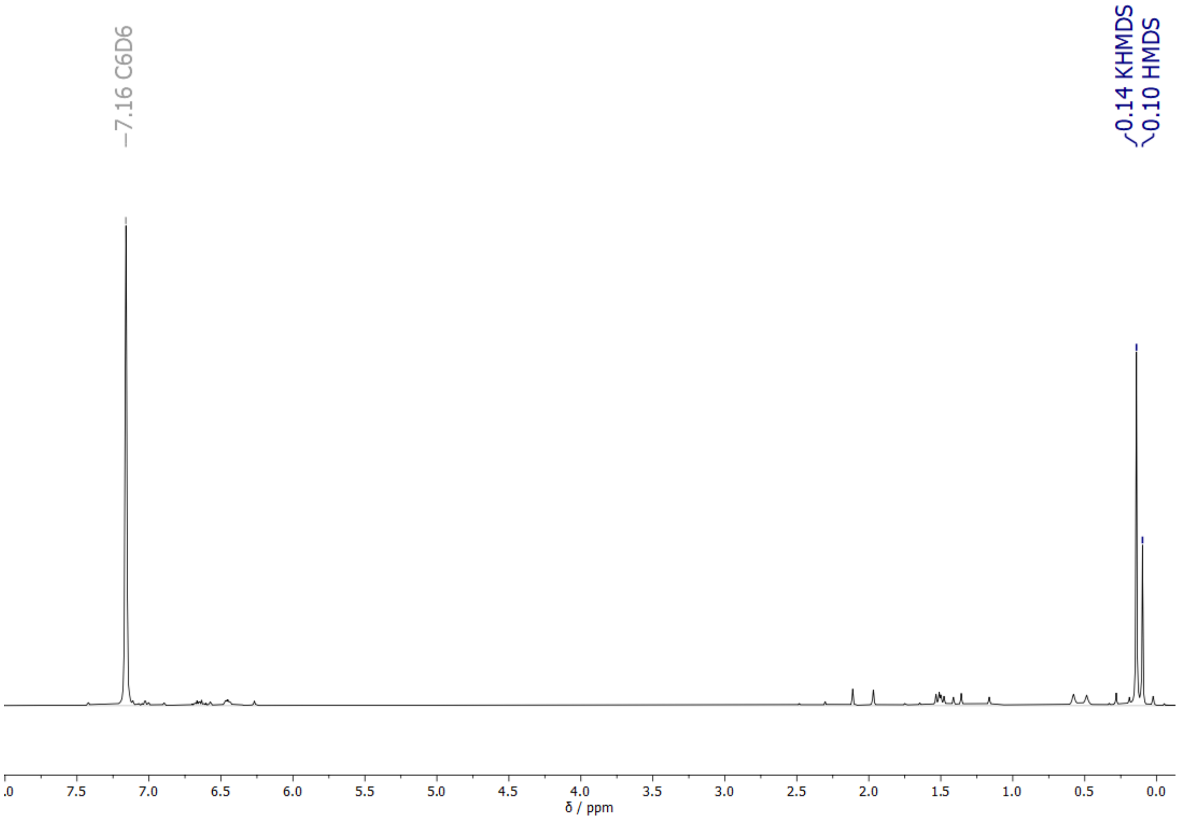

Figure S83. ^{1}H NMR spectrum of the reaction solution of $\mathbf{3^{BArF}}$ and KHMDS, C_6D_6, 25 °C.

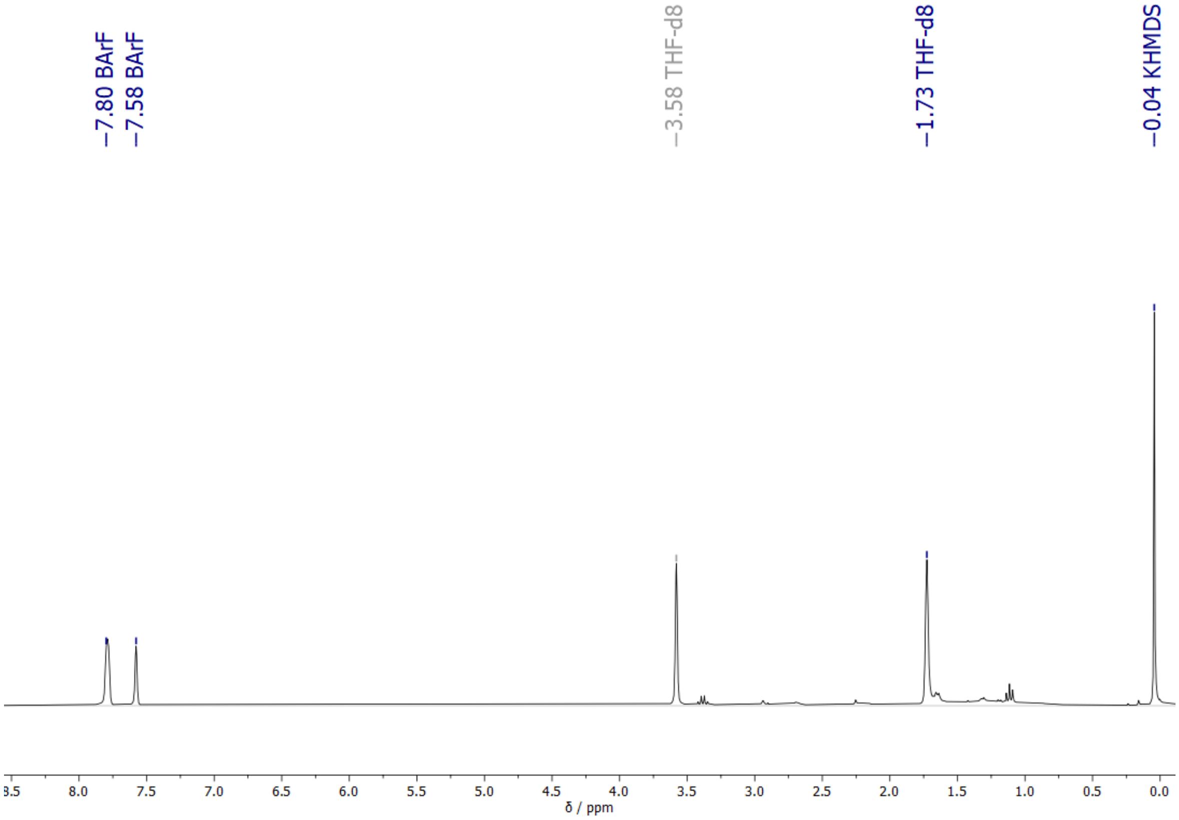

Figure S84. ^{1}H NMR spectrum of the reaction solution of **3**$^{\mathbf{BArF}}$ and KHMDS synthesized in Et_2O and re-dissolved, THF-d_8, 25 °C.

Single Crystal X-ray Crystallography

Crystals were mounted on a MiTeGen micromount with perfluoroether oil. Data were collected from a shock-cooled single crystal on a Bruker D8 VENTURE dual wavelength Mo/Cu three-circle diffractometer with a microfocus sealed X-ray tube using a mirror optics as monochromator and a Bruker PHOTON III detector. The diffractometer used Mo K_α radiation (λ = 0.71073 Å). All data were integrated with SAINT V8.40B and a multi-scan absorption correction using SADABS 2016/2 was applied. The structure was solved by direct methods with SHELXT 2018/2 and refined by full-matrix least-squares methods against F^2 using SHELXL-2019/2.[13,14] All hydrogen atoms were refined isotropic on calculated positions using a riding model with their U_{iso} values constrained to 1.5 times the U_{eq} of their pivot atoms for terminal sp^3 carbon atoms and 1.2 times for all other carbon atoms. Crystallographic data for the structures reported in this paper have been deposited with the Cambridge Crystallographic Data Centre.[15] CCDC 2476840, 2476845, 2476838, 2495710 and 2497817 contain the supplementary crystallographic data for this paper. These data can be obtained free of charge from The Cambridge Crystallographic Data Centre *via* www.ccdc.cam.ac.uk/structures.

1^{Bi}

CCDC number	2476840
Empirical formula	$C_{23}H_{30}BiN_3$
Formula weight	557.48
Temperature [K]	100(2)
Crystal system	orthorhombic
Space group (number)	$Cmca$ (64)
a [Å]	23.2608(8)
b [Å]	17.6704(6)
c [Å]	10.0773(4)
α [°]	90
β [°]	90
γ [°]	90
Volume [Å^3]	4142.0(3)
Z	8
ρ_{calc} [gcm^{-3}]	1.788
μ [mm^{-1}]	8.526
F(000)	2176
Crystal size [mm^3]	0.020×0.040×0.130
Crystal color	clear violet
Crystal shape	block
Radiation	MoK_α (λ=0.71073 Å)
2θ range [°]	4.61 to 61.12 (0.70 Å)
Index ranges	−33 ≤ h ≤ 33<br>−24 ≤ k ≤ 25<br>−14 ≤ l ≤ 14
Reflections collected	57524
Independent reflections	3251<br>R_{int} = 0.0539<br>R_{sigma} = 0.0187
Completeness to θ = 25.242°	99.9 %
Data / Restraints / Parameters	3251 / 7 / 135
Absorption correction T_{min}/T_{max} (method)	0.5190 / 0.7461 (multi-scan)
Goodness-of-fit on F^2	1.146
Final *R* indexes [I≥2σ(I)]	R_1 = 0.0189<br>wR_2 = 0.0426
Final *R* indexes [all data]	R_1 = 0.0250<br>wR_2 = 0.0453
Largest peak/hole [eÅ^{-3}]	0.99/−0.94

Atom–Atom	Length [Å]
Bi1–N2	2.168(3)
Bi1–N1	2.300(2)
Bi1–N1$^{\#1}$	2.300(2)
N1–C2	1.352(3)
N1–C11	1.465(3)
N2–C1$^{\#1}$	1.372(2)
N2–C1	1.372(2)
C1–C6	1.411(3)
C1–C2	1.431(3)
C5–C6	1.388(3)
C5–C4	1.408(3)
C6–C7	1.525(3)
C7–C10	1.540(4)
C7–C9	1.548(5)
C8–C4	1.504(3)
C11–C12	1.527(3)
C11–C13	1.530(4)
C2–C3	1.420(3)
C3–C4	1.382(3)

Atom–Atom–Atom	Angle [°]
N2–Bi1–N1	72.29(5)
N2–Bi1–N1$^{\#1}$	72.29(5)
N1–Bi1–N1$^{\#1}$	143.37(10)
C2–N1–C11	119.4(2)
C2–N1–Bi1	115.68(15)
C11–N1–Bi1	124.90(15)
C1$^{\#1}$–N2–C1	121.3(3)
C1$^{\#1}$–N2–Bi1	119.32(13)
C1–N2–Bi1	119.32(13)
N2–C1–C6	121.8(2)
N2–C1–C2	116.4(2)
C6–C1–C2	121.8(2)
C6–C5–C4	121.8(2)
C5–C6–C1	118.2(2)
C5–C6–C7	121.8(2)
C1–C6–C7	119.7(2)
C6$^{\#1}$–C7–C6	111.6(3)
C6$^{\#1}$–C7–C10	111.23(17)
C6–C7–C10	111.23(17)
C6$^{\#1}$–C7–C9	106.99(18)
C6–C7–C9	106.99(18)
C10–C7–C9	108.6(3)
N1–C11–C12	110.5(2)
N1–C11–C13	111.6(2)
C12–C11–C13	110.1(2)
N1–C2–C3	126.7(2)
N1–C2–C1	116.1(2)
C3–C2–C1	117.2(2)
C4–C3–C2	121.4(2)
C3–C4–C5	119.7(2)
C3–C4–C8	121.5(2)
C5–C4–C8	118.8(2)

Bonds and angles to hydrogen atoms were omitted.
Symmetry transformations used to generate equivalent atoms:
#1: 1-X, +Y, +Z;

2

CCDC number	2476845
Empirical formula	$C_{55}H_{43}BBiF_{24}N_3$
Formula weight	1421.741
Temperature [K]	104.00
Crystal system	monoclinic
Space group (number)	$P2_1/n$ (14)
a [Å]	18.2575(12)
b [Å]	14.9341(12)
c [Å]	20.9639(16)
α [°]	90
β [°]	98.913(3)
γ [°]	90
Volume [Å^3]	5647.0(7)
Z	4
ρ_{calc} [gcm^{-3}]	1.672
μ [mm^{-1}]	3.241
F(000)	2781.456
Crystal size [mm^3]	0.07×0.08×0.35
Crystal color	purple
Crystal shape	block
Radiation	Mo K_α (λ=0.71073 Å)
2θ range [°]	4.22 to 50.76 (0.83 Å)
Index ranges	−22 ≤ h ≤ 21<br>−18 ≤ k ≤ 18<br>−25 ≤ l ≤ 25
Reflections collected	122577
Independent reflections	10349<br>R_{int} = 0.0384<br>R_{sigma} = 0.0199
Completeness to θ = 25.2417°	99.9 %
Data / Restraints / Parameters	10349 / 0 / 765
Absorption correction T_{min}/T_{max} (method)	0.4940 / 0.7452 (multi-scan)
Goodness-of-fit on F^2	1.0495
Final *R* indexes [I≥2σ(I)]	R_1 = 0.0302<br>wR_2 = 0.0662
Final *R* indexes [all data]	R_1 = 0.0323<br>wR_2 = 0.0671
Largest peak/hole [eÅ^{-3}]	2.75/−2.21

Atom–Atom	Length [Å]
F1–C30	1.332(5)
F2–C30	1.336(4)
F3–C30	1.334(4)
F4–C31	1.325(5)
F5–C31	1.321(5)
F6–C31	1.331(5)
F7–C38	1.347(4)
F8–C38	1.333(4)
F9–C38	1.333(4)
F10–C39	1.321(4)
F11–C39	1.348(4)
F12–C39	1.341(4)
F13–C46	1.345(4)
F14–C46	1.351(4)
F15–C46	1.340(4)
F16–C47	1.341(4)
F17–C47	1.349(4)
F18–C47	1.342(4)
F19–C54	1.339(4)
F20–C54	1.341(4)
F21–C54	1.332(4)
F22–C55	1.326(4)
F23–C55	1.324(4)
F24–C55	1.336(4)
Bi1–N1	2.227(3)
Bi1–N2	2.176(3)
Bi1–N3	2.556(3)
N1–C2	1.360(4)
N1–C18	1.477(4)
N2–C1	1.365(4)
N2–C13	1.404(4)
N3–C12	1.439(4)
N3–C21	1.483(4)
C1–C2	1.429(5)
C1–C6	1.418(5)
C2–C3	1.416(5)
C3–C4	1.368(5)
C4–C5	1.415(5)
C4–C14	1.507(5)
C5–C6	1.375(5)
C6–C7	1.519(5)
C7–C8	1.517(5)
C7–C15	1.545(5)
C7–C16	1.551(5)
C8–C9	1.394(5)
C8–C13	1.403(5)
C9–C10	1.391(5)
C10–C11	1.384(5)
C10–C17	1.512(5)
C11–C12	1.391(5)
C12–C13	1.408(5)
C18–C19	1.524(5)
C18–C20	1.509(5)
C21–C22	1.509(5)
C21–C23	1.527(6)
C24–C25	1.402(4)
C24–C29	1.400(5)
C24–B1	1.644(5)
C25–C26	1.390(5)
C26–C27	1.388(5)
C26–C30	1.496(5)
C27–C28	1.388(5)
C28–C29	1.390(5)
C28–C31	1.499(5)
C32–C33	1.402(4)
C32–C37	1.405(4)
C32–B1	1.635(5)
C33–C34	1.385(5)
C34–C35	1.389(5)
C34–C38	1.491(5)
C35–C36	1.385(5)
C36–C37	1.391(5)
C36–C39	1.494(5)
C40–C41	1.400(4)
C40–C45	1.405(4)
C40–B1	1.646(5)
C41–C42	1.392(5)
C42–C43	1.390(4)
C42–C46	1.495(4)
C43–C44	1.388(4)
C44–C45	1.391(4)
C44–C47	1.500(4)
C48–C49	1.400(4)
C48–C53	1.405(4)
C48–B1	1.644(5)
C49–C50	1.394(4)
C50–C51	1.388(5)
C50–C54	1.501(5)
C51–C52	1.387(5)
C52–C53	1.393(5)
C52–C55	1.501(5)

**Atom–Atom–Atom**	**Angle [°]**
N2–Bi1–N1	73.89(10)
N3–Bi1–N1	144.28(10)
N3–Bi1–N2	70.91(10)
C2–N1–Bi1	116.0(2)
C18–N1–Bi1	123.4(2)
C18–N1–C2	120.1(3)
C1–N2–Bi1	117.0(2)
C13–N2–Bi1	123.0(2)
C13–N2–C1	120.0(3)
C12–N3–Bi1	109.0(2)
C21–N3–Bi1	115.1(2)
C21–N3–C12	115.9(3)
C2–C1–N2	117.0(3)
C6–C1–N2	122.8(3)
C6–C1–C2	120.2(3)
C1–C2–N1	116.0(3)
C3–C2–N1	124.8(3)
C3–C2–C1	119.2(3)
C4–C3–C2	120.5(3)
C5–C4–C3	119.4(3)
C14–C4–C3	121.6(3)
C14–C4–C5	119.0(3)
C6–C5–C4	122.9(3)
C5–C6–C1	117.9(3)
C7–C6–C1	121.2(3)
C7–C6–C5	120.9(3)
C8–C7–C6	111.4(3)
C15–C7–C6	109.9(3)
C15–C7–C8	109.8(3)
C16–C7–C6	107.2(3)
C16–C7–C8	108.6(3)
C16–C7–C15	109.8(3)
C9–C8–C7	120.0(3)
C13–C8–C7	122.1(3)
C13–C8–C9	117.8(3)
C10–C9–C8	123.0(3)
C11–C10–C9	118.3(3)
C17–C10–C9	121.4(3)
C17–C10–C11	120.3(3)
C12–C11–C10	120.8(3)
C11–C12–N3	121.4(3)
C13–C12–N3	118.4(3)
C13–C12–C11	120.2(3)
C8–C13–N2	121.6(3)
C12–C13–N2	118.5(3)
C12–C13–C8	119.9(3)
C19–C18–N1	109.4(3)
C20–C18–N1	110.2(3)
C20–C18–C19	111.3(3)
C22–C21–N3	108.8(3)
C23–C21–N3	108.8(3)
C23–C21–C22	113.0(3)
C29–C24–C25	115.3(3)
B1–C24–C25	123.9(3)
B1–C24–C29	120.6(3)
C26–C25–C24	122.2(3)
C27–C26–C25	121.2(3)
C30–C26–C25	121.0(3)
C30–C26–C27	117.8(3)
C28–C27–C26	117.6(3)
C29–C28–C27	120.8(3)
C31–C28–C27	120.0(3)
C31–C28–C29	119.2(3)
C28–C29–C24	122.7(3)
F2–C30–F1	105.8(3)
F3–C30–F1	105.6(3)
F3–C30–F2	106.1(3)
C26–C30–F1	112.9(3)
C26–C30–F2	113.4(3)
C26–C30–F3	112.4(3)
F5–C31–F4	107.2(3)
F6–C31–F4	105.3(4)
F6–C31–F5	105.1(4)
C28–C31–F4	113.7(3)
C28–C31–F5	112.3(3)
C28–C31–F6	112.6(3)
C37–C32–C33	115.4(3)
B1–C32–C33	123.6(3)
B1–C32–C37	121.0(3)
C34–C33–C32	122.5(3)
C35–C34–C33	121.1(3)
C38–C34–C33	119.8(3)
C38–C34–C35	119.1(3)
C36–C35–C34	117.8(3)
C37–C36–C35	121.0(3)
C39–C36–C35	120.6(3)
C39–C36–C37	118.4(3)
C36–C37–C32	122.3(3)
F8–C38–F7	106.2(3)
F9–C38–F7	105.9(3)
F9–C38–F8	106.4(3)

C34–C38–F7	111.7(3)
C34–C38–F8	112.8(3)
C34–C38–F9	113.3(3)
F11–C39–F10	105.6(3)
F12–C39–F10	107.8(3)
F12–C39–F11	104.8(3)
C36–C39–F10	112.7(3)
C36–C39–F11	112.1(3)
C36–C39–F12	113.3(3)
C45–C40–C41	115.5(3)
B1–C40–C41	122.2(3)
B1–C40–C45	122.3(3)
C42–C41–C40	122.4(3)
C43–C42–C41	121.1(3)
C46–C42–C41	118.6(3)
C46–C42–C43	120.1(3)
C44–C43–C42	117.6(3)
C45–C44–C43	121.1(3)
C47–C44–C43	120.0(3)
C47–C44–C45	118.8(3)
C44–C45–C40	122.3(3)
F14–C46–F13	105.6(3)
F15–C46–F13	106.8(3)
F15–C46–F14	106.5(3)
C42–C46–F13	112.1(3)
C42–C46–F14	112.6(3)
C42–C46–F15	112.8(3)
F17–C47–F16	106.0(3)
F18–C47–F16	106.6(3)
F18–C47–F17	106.2(3)
C44–C47–F16	112.4(3)
C44–C47–F17	112.1(3)
C44–C47–F18	113.0(3)
C53–C48–C49	115.7(3)
B1–C48–C49	120.4(3)
B1–C48–C53	123.7(3)
C50–C49–C48	122.4(3)
C51–C50–C49	121.0(3)
C54–C50–C49	117.9(3)
C54–C50–C51	121.1(3)
C52–C51–C50	117.5(3)
C53–C52–C51	121.6(3)
C55–C52–C51	119.7(3)
C55–C52–C53	118.7(3)
C52–C53–C48	121.8(3)
F20–C54–F19	105.2(3)
F21–C54–F19	107.2(3)
F21–C54–F20	106.3(3)
C50–C54–F19	112.4(3)
C50–C54–F20	112.0(3)
C50–C54–F21	113.2(3)
F23–C55–F22	107.1(3)
F24–C55–F22	105.5(3)
F24–C55–F23	105.8(3)
C52–C55–F22	113.4(3)
C52–C55–F23	112.6(3)
C52–C55–F24	112.0(3)
C32–B1–C24	104.6(3)
C40–B1–C24	110.6(3)
C40–B1–C32	112.0(3)
C48–B1–C24	114.7(3)
C48–B1–C32	110.1(3)
C48–B1–C40	105.0(3)

Bonds and angles to hydrogen atoms were omitted.

3^{OTf}

CCDC number	2476838
Empirical formula	$C_{25}H_{33}BiF_3N_3O_3S$
Formula weight	721.58
Temperature [K]	100(2)
Crystal system	orthorhombic
Space group (number)	$Pmn2_1$ (31)
a [Å]	15.549(6)
b [Å]	11.758(3)
c [Å]	7.322(3)
α [°]	90
β [°]	90
γ [°]	90
Volume [Å^3]	1338.7(8)
Z	2
ρ_{calc} [gcm^{-3}]	1.790
μ [mm^{-1}]	6.716
F(000)	708
Crystal size [mm^3]	0.040×0.060×0.200
Crystal color	green
Crystal shape	block
Radiation	MoK_α (λ=0.71073 Å)
2θ range [°]	4.34 to 61.28 (0.70 Å)
Index ranges	−22 ≤ h ≤ 22<br>−15 ≤ k ≤ 16<br>−10 ≤ l ≤ 10
Reflections collected	36460
Independent reflections	4223<br>R_{int} = 0.0385<br>R_{sigma} = 0.0326
Completeness to θ = 25.242°	99.9 %
Data / Restraints / Parameters	4223 / 4 / 185
Absorption correction T_{min}/T_{max} (method)	0.5286 / 0.7461 (multi-scan)
Goodness-of-fit on F^2	1.085
Final *R* indexes [I≥2σ(I)]	R_1 = 0.0227<br>wR_2 = 0.0531
Final *R* indexes [all data]	R_1 = 0.0251<br>wR_2 = 0.0543
Largest peak/hole [eÅ^{-3}]	1.56/−1.02
Flack X parameter	0.006(8)

Refined as a 2-component inversion twin.

**Atom–Atom**	**Length [Å]**
Bi1–C14	2.188(9)
Bi1–N2	2.298(5)
Bi1–N1	2.384(4)
Bi1–N1$^{\#1}$	2.384(4)
Bi1–O1	2.707(6)
S1–O2$^{\#1}$	1.436(4)
S1–O2	1.436(4)
S1–O1	1.440(6)
S1–C15	1.806(9)
F1–C15	1.358(11)
N1–C2	1.320(6)
N1–C11	1.474(6)
C1–N2	1.346(5)
C1–C6	1.426(6)
C1–C2	1.457(6)
F2–C15	1.355(6)
C2–C3	1.442(6)
C3–C4	1.355(6)
C4–C5	1.431(6)
C4–C8	1.499(6)
C5–C6	1.369(6)
C6–C7	1.525(6)
C7–C9	1.518(10)
C7–C10	1.559(10)
C11–C12	1.526(8)
C11–C13	1.528(7)

**Atom–Atom–Atom**	**Angle [°]**
C14–Bi1–N2	96.7(3)
C14–Bi1–N1	88.98(13)
N2–Bi1–N1	70.16(9)
C14–Bi1–N1$^{\#1}$	88.98(13)
N2–Bi1–N1$^{\#1}$	70.16(9)
N1–Bi1–N1$^{\#1}$	139.73(18)
C14–Bi1–O1	80.7(3)
N2–Bi1–O1	177.4(2)
N1–Bi1–O1	109.66(9)
N1$^{\#1}$–Bi1–O1	109.66(9)
O2$^{\#1}$–S1–O2	115.9(3)
O2$^{\#1}$–S1–O1	115.4(2)
O2–S1–O1	115.4(2)
O2$^{\#1}$–S1–C15	102.6(3)
O2–S1–C15	102.7(3)
O1–S1–C15	101.6(4)
S1–O1–Bi1	175.9(4)
C2–N1–C11	119.8(4)
C2–N1–Bi1	115.0(3)
C11–N1–Bi1	124.2(3)
N2–C1–C6	121.4(4)
N2–C1–C2	117.0(4)
C6–C1–C2	121.6(4)
C1–N2–C1$^{\#1}$	123.0(6)
C1–N2–Bi1	118.0(3)
C1$^{\#1}$–N2–Bi1	118.0(3)
N1–C2–C3	125.5(4)
N1–C2–C1	118.1(4)
C3–C2–C1	116.4(4)
C4–C3–C2	121.1(4)
C3–C4–C5	120.9(4)
C3–C4–C8	120.5(4)
C5–C4–C8	118.5(4)
C6–C5–C4	121.7(4)
C5–C6–C1	118.2(4)
C5–C6–C7	122.6(4)
C1–C6–C7	118.9(4)
C9–C7–C6$^{\#1}$	112.5(3)
C9–C7–C6	112.5(3)
C6$^{\#1}$–C7–C6	111.1(5)
C9–C7–C10	108.8(6)
C6$^{\#1}$–C7–C10	105.8(4)
C6–C7–C10	105.8(4)
N1–C11–C12	108.7(4)
N1–C11–C13	110.4(4)
C12–C11–C13	112.0(5)
F2–C15–F2$^{\#1}$	105.6(7)
F2–C15–F1	106.9(5)
F2$^{\#1}$–C15–F1	106.9(5)
F2–C15–S1	112.1(4)
F2$^{\#1}$–C15–S1	112.1(4)
F1–C15–S1	113.0(6)

Bonds and angles to hydrogen atoms were omitted.
Symmetry transformations used to generate equivalent atoms:
#1: 1-X, +Y, +Z;

3[BArF]

CCDC number	2476839
Empirical formula	$C_{56}H_{45}BBiF_{24}N_3$
Formula weight	1435.74
Temperature [K]	100(2)
Crystal system	triclinic
Space group (number)	$P\overline{1}$ (2)
a [Å]	12.6269(9)
b [Å]	14.8204(9)
c [Å]	17.6874(13)
α [°]	111.489(3)
β [°]	92.316(3)
γ [°]	95.322(3)
Volume [Å^3]	3056.7(4)
Z	2
ρ_{calc} [gcm^{-3}]	1.560
μ [mm^{-1}]	2.995
F(000)	1412
Crystal size [mm^3]	0.040×0.120×0.170
Crystal color	green
Crystal shape	block
Radiation	MoK_α (λ=0.71073 Å)
2θ range [°]	3.93 to 52.85 (0.80 Å)
Index ranges	−15 ≤ h ≤ 15<br>−18 ≤ k ≤ 18<br>−22 ≤ l ≤ 22
Reflections collected	102949
Independent reflections	12582<br>R_{int} = 0.0410<br>R_{sigma} = 0.0217
Completeness to θ = 25.242°	100.0 %
Data / Restraints / Parameters	12582 / 107 / 760
Absorption correction T_{min}/T_{max} (method)	0.4957 / 0.7461 (multi-scan)
Goodness-of-fit on F^2	1.019
Final *R* indexes [I≥2σ(I)]	R_1 = 0.0375<br>wR_2 = 0.0948
Final *R* indexes [all data]	R_1 = 0.0397<br>wR_2 = 0.0963
Largest peak/hole [eÅ^{-3}]	2.26/−1.36

**Atom–Atom**	**Length [Å]**
Bi1–C24	2.228(4)
Bi1–N2	2.254(3)
Bi1–N3	2.341(3)
Bi1–N1	2.369(3)
N1–C2	1.324(5)
N1–C18	1.475(5)
C1–N2	1.347(5)
C1–C6	1.432(5)
C1–C2	1.442(5)
B1–C33	1.630(5)
B1–C25	1.640(5)
B1–C41	1.645(5)
B1–C49	1.645(5)
F1–C31	1.371(8)
F2–C31	1.339(8)
F3–C31	1.323(8)
F1A–C31	1.388(10)
F2A–C31	1.318(9)
F3A–C31	1.328(10)
N2–C13	1.352(5)
C2–C3	1.442(5)
N3–C12	1.322(5)
N3–C21	1.477(5)
C3–C4	1.361(6)
C4–C5	1.433(6)
C4–C14	1.500(6)
F4–C32	1.356(8)
F5–C32	1.329(7)
F6–C32	1.329(8)
F4A–C32	1.329(9)
F5A–C32	1.320(8)
F6A–C32	1.362(9)
C5–C6	1.360(5)
C6–C7	1.523(5)
C7–C8	1.526(5)
C7–C16	1.528(5)
C7–C15	1.549(5)
F7–C40	1.471(7)
F8–C40	1.269(6)
F9–C40	1.296(6)
F7A–C40	1.333(10)
F8A–C40	1.642(10)
F9A–C40	1.290(11)
C8–C9	1.363(5)
C8–C13	1.429(5)

C9–C10	1.424(6)
F10–C39	1.332(5)
C10–C11	1.357(6)
C10–C17	1.505(6)
F11–C39	1.336(5)
C11–C12	1.437(6)
F12–C39	1.337(5)
C12–C13	1.445(5)
F13–C47	1.354(7)
F14–C47	1.328(7)
F15–C47	1.307(7)
F13A–C47	1.346(12)
F14A–C47	1.261(12)
F15A–C47	1.432(12)
F16–C48	1.447(7)
F17–C48	1.303(6)
F18–C48	1.288(8)
F16A–C48	1.412(9)
F17A–C48	1.265(9)
F18A–C48	1.392(11)
C18–C20	1.520(7)
C18–C19	1.522(7)
F19–C55	1.329(6)
F20–C55	1.324(6)
F21–C55	1.340(7)
C21–C23	1.514(7)
C21–C22	1.520(7)
F22–C56	1.439(8)
F23–C56	1.320(8)
F24–C56	1.303(6)
F22A–C56	1.324(15)
F23A–C56	1.191(10)
F24A–C56	1.476(10)
C25–C26	1.403(5)
C25–C30	1.407(5)
C26–C27	1.392(5)
C27–C28	1.382(6)
C27–C31	1.498(5)
C28–C29	1.385(6)
C29–C30	1.393(5)
C29–C32	1.497(5)
C33–C34	1.399(5)
C33–C38	1.401(5)
C34–C35	1.389(5)
C35–C36	1.379(5)
C35–C39	1.497(5)

C36–C37	1.388(6)
C37–C38	1.388(6)
C37–C40	1.476(6)
C41–C46	1.392(5)
C41–C42	1.407(5)
C42–C43	1.393(5)
C43–C44	1.385(6)
C43–C47	1.493(5)
C44–C45	1.380(6)
C45–C46	1.398(5)
C45–C48	1.491(6)
C49–C50	1.398(5)
C49–C54	1.403(5)
C50–C51	1.396(5)
C51–C52	1.388(7)
C51–C55	1.496(6)
C52–C53	1.379(7)
C53–C54	1.392(5)
C53–C56	1.501(7)

**Atom–Atom–Atom**	**Angle [°]**
C24–Bi1–N2	90.46(14)
C24–Bi1–N3	87.64(14)
N2–Bi1–N3	71.06(11)
C24–Bi1–N1	91.55(14)
N2–Bi1–N1	70.37(11)
N3–Bi1–N1	141.41(11)
C2–N1–C18	121.6(3)
C2–N1–Bi1	114.5(2)
C18–N1–Bi1	120.6(3)
N2–C1–C6	121.4(3)
N2–C1–C2	116.9(3)
C6–C1–C2	121.7(3)
C33–B1–C25	112.2(3)
C33–B1–C41	111.0(3)
C25–B1–C41	105.0(3)
C33–B1–C49	104.1(3)
C25–B1–C49	111.8(3)
C41–B1–C49	112.9(3)
C1–N2–C13	122.8(3)
C1–N2–Bi1	118.7(2)
C13–N2–Bi1	118.3(2)
N1–C2–C3	126.2(4)
N1–C2–C1	117.0(3)
C3–C2–C1	116.8(3)
C12–N3–C21	121.4(3)
C12–N3–Bi1	115.9(2)
C21–N3–Bi1	121.5(3)
C4–C3–C2	120.4(4)
C3–C4–C5	120.8(4)
C3–C4–C14	120.8(4)
C5–C4–C14	118.4(4)
C6–C5–C4	122.0(4)
C5–C6–C1	117.6(3)
C5–C6–C7	124.5(3)
C1–C6–C7	117.6(3)
C6–C7–C8	110.8(3)
C6–C7–C16	112.5(3)
C8–C7–C16	112.4(3)
C6–C7–C15	106.1(3)
C8–C7–C15	105.6(3)
C16–C7–C15	108.9(3)
C9–C8–C13	118.1(4)
C9–C8–C7	123.3(3)
C13–C8–C7	118.2(3)
C8–C9–C10	121.8(4)
C11–C10–C9	120.7(4)
C11–C10–C17	121.8(4)
C9–C10–C17	117.5(4)
C10–C11–C12	121.4(4)
N3–C12–C11	126.0(4)
N3–C12–C13	117.7(3)
C11–C12–C13	116.3(4)
N2–C13–C8	121.3(3)
N2–C13–C12	116.8(3)
C8–C13–C12	121.7(3)
N1–C18–C20	108.5(3)
N1–C18–C19	111.4(4)
C20–C18–C19	111.4(4)
N3–C21–C23	110.3(4)
N3–C21–C22	110.9(4)
C23–C21–C22	111.1(4)
C26–C25–C30	115.5(3)
C26–C25–B1	123.1(3)
C30–C25–B1	121.1(3)
C27–C26–C25	122.5(3)
C28–C27–C26	120.9(4)
C28–C27–C31	119.9(4)
C26–C27–C31	119.2(4)
C27–C28–C29	118.0(4)
C28–C29–C30	121.3(4)
C28–C29–C32	119.7(4)
C30–C29–C32	119.0(3)

C29–C30–C25	121.8(3)	F8–C40–C37	115.5(4)
F2A–C31–F3A	109.1(6)	F9A–C40–C37	121.3(8)
F3–C31–F2	106.1(5)	F9–C40–C37	115.8(4)
F3–C31–F1	107.7(5)	F7A–C40–C37	121.9(7)
F2–C31–F1	104.6(5)	F7–C40–C37	107.7(4)
F2A–C31–F1A	104.0(5)	F9A–C40–F8A	93.7(8)
F3A–C31–F1A	106.7(5)	F7A–C40–F8A	86.0(7)
F2A–C31–C27	113.6(4)	C37–C40–F8A	100.6(5)
F3–C31–C27	113.9(4)	C46–C41–C42	115.4(3)
F3A–C31–C27	113.8(5)	C46–C41–B1	123.7(3)
F2–C31–C27	113.7(4)	C42–C41–B1	120.7(3)
F1–C31–C27	110.2(4)	C43–C42–C41	122.4(3)
F1A–C31–C27	108.9(4)	C44–C43–C42	120.8(3)
F6–C32–F5	108.4(5)	C44–C43–C47	119.5(4)
F5A–C32–F4A	113.6(6)	C42–C43–C47	119.6(4)
F6–C32–F4	102.0(5)	C45–C44–C43	117.9(3)
F5–C32–F4	108.4(5)	C44–C45–C46	121.1(4)
F5A–C32–F6A	105.7(6)	C44–C45–C48	119.9(4)
F4A–C32–F6A	97.5(6)	C46–C45–C48	118.9(4)
F5A–C32–C29	115.1(5)	C41–C46–C45	122.3(3)
F6–C32–C29	114.7(4)	F15–C47–F14	107.7(5)
F5–C32–C29	113.1(4)	F14A–C47–F13A	115.9(8)
F4A–C32–C29	110.9(5)	F15–C47–F13	104.6(5)
F4–C32–C29	109.6(4)	F14–C47–F13	105.7(5)
F6A–C32–C29	112.7(5)	F14A–C47–F15A	105.2(8)
C34–C33–C38	115.4(3)	F13A–C47–F15A	93.6(7)
C34–C33–B1	122.5(3)	F14A–C47–C43	118.8(6)
C38–C33–B1	121.7(3)	F15–C47–C43	114.0(4)
C35–C34–C33	122.3(3)	F14–C47–C43	112.9(4)
C36–C35–C34	121.2(3)	F13A–C47–C43	110.6(5)
C36–C35–C39	120.0(3)	F13–C47–C43	111.2(4)
C34–C35–C39	118.8(3)	F15A–C47–C43	109.3(5)
C35–C36–C37	117.7(4)	F18–C48–F17	115.1(5)
C36–C37–C38	121.1(4)	F17A–C48–F18A	107.9(8)
C36–C37–C40	120.4(4)	F17A–C48–F16A	105.1(6)
C38–C37–C40	118.5(4)	F18A–C48–F16A	98.6(7)
C37–C38–C33	122.3(4)	F18–C48–F16	100.7(5)
F10–C39–F12	105.5(4)	F17–C48–F16	100.3(4)
F10–C39–F11	106.1(3)	F17A–C48–C45	121.9(6)
F12–C39–F11	106.4(4)	F18–C48–C45	116.2(5)
F10–C39–C35	112.8(3)	F17–C48–C45	115.4(4)
F12–C39–C35	112.1(3)	F18A–C48–C45	111.1(8)
F11–C39–C35	113.3(3)	F16A–C48–C45	109.6(5)
F8–C40–F9	113.0(5)	F16–C48–C45	105.9(4)
F9A–C40–F7A	115.6(9)	C50–C49–C54	115.5(3)
F8–C40–F7	100.9(4)	C50–C49–B1	123.4(3)
F9–C40–F7	101.4(4)	C54–C49–B1	120.8(3)

C51–C50–C49	122.0(4)
C52–C51–C50	121.0(4)
C52–C51–C55	119.6(4)
C50–C51–C55	119.3(4)
C53–C52–C51	118.0(4)
C52–C53–C54	120.8(4)
C52–C53–C56	121.7(4)
C54–C53–C56	117.5(4)
C53–C54–C49	122.5(4)
F20–C55–F19	107.5(4)
F20–C55–F21	105.2(4)
F19–C55–F21	105.6(5)
F20–C55–C51	113.3(4)
F19–C55–C51	111.7(4)
F21–C55–C51	113.0(4)
F24–C56–F23	110.7(5)
F23A–C56–F22A	116.6(9)
F24–C56–F22	103.1(4)
F23–C56–F22	101.6(6)
F23A–C56–F24A	108.1(6)
F22A–C56–F24A	99.4(8)
F23A–C56–C53	111.6(6)
F24–C56–C53	117.8(5)
F23–C56–C53	110.6(5)
F22A–C56–C53	116.6(9)
F22–C56–C53	111.7(4)
F24A–C56–C53	102.4(5)

Bonds and angles to hydrogen atoms were omitted.

3[Et_BArF]

CCDC number	2495710
Empirical formula	$C_{60}H_{49}BBiF_{25}N_3$
Formula weight	1506.94
Temperature [K]	100(2)
Crystal system	triclinic
Space group (number)	$P\overline{1}$ (2)
a [Å]	12.9857(4)
b [Å]	14.0789(5)
c [Å]	17.7347(6)
α [°]	91.2040(10)
β [°]	101.2130(10)
γ [°]	99.5640(10)
Volume [Å^3]	3131.47(18)
Z	2
ρ_{calc} [gcm^{-3}]	1.598
μ [mm^{-1}]	2.930
F(000)	1486
Crystal size [mm^3]	0.030×0.070×0.160
Crystal color	green
Crystal shape	needle
Radiation	MoK_α (λ=0.71073 Å)
2θ range [°]	3.86 to 56.67 (0.75 Å)
Index ranges	−17 ≤ h ≤ 17<br>−18 ≤ k ≤ 18<br>−23 ≤ l ≤ 23
Reflections collected	131297
Independent reflections	15617<br>R_{int} = 0.0535<br>R_{sigma} = 0.0276
Completeness to θ = 25.242°	99.9 %
Data / Restraints / Parameters	15617 / 9 / 829
Absorption correction T_{min}/T_{max} (method)	0.5975 / 0.7457 (multi-scan)
Goodness-of-fit on F^2	1.082
Final *R* indexes [I≥2σ(I)]	R_1 = 0.0353<br>wR_2 = 0.0967
Final *R* indexes [all data]	R_1 = 0.0379<br>wR_2 = 0.0982
Largest peak/hole [eÅ^{-3}]	2.56/−1.08

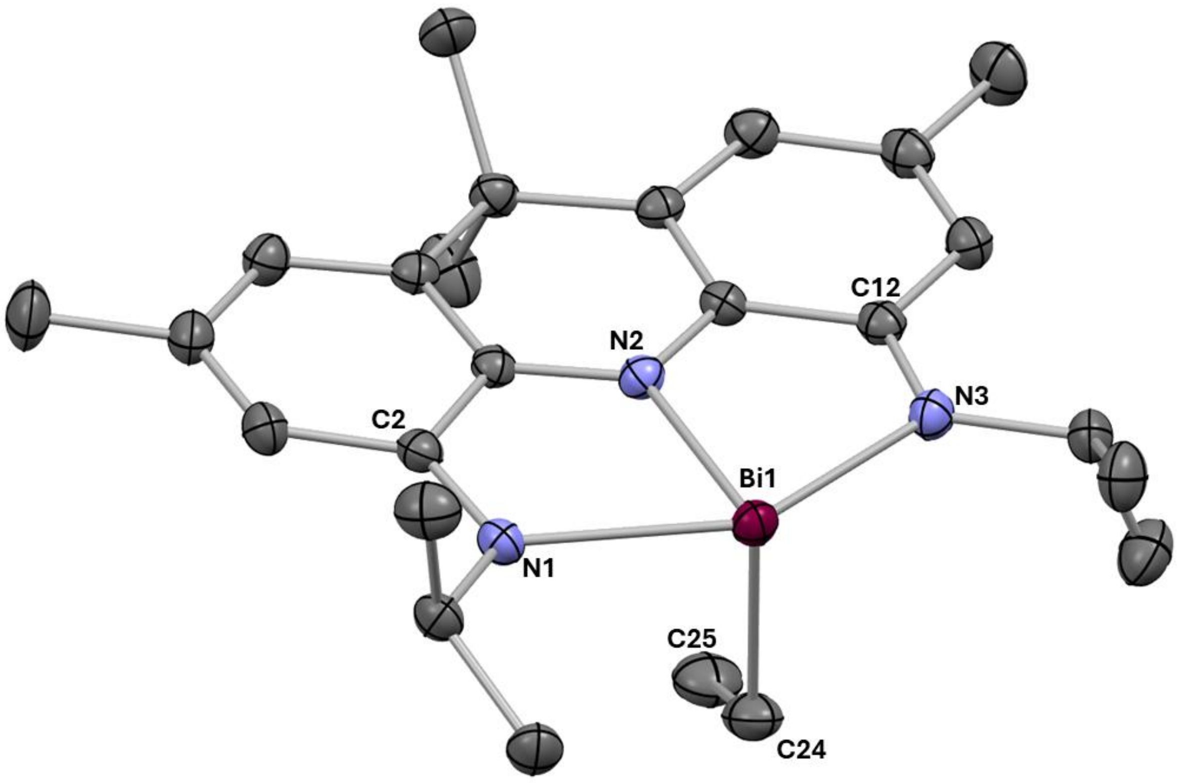

Figure S85. Molecular structure of $\mathbf{3}^{Et\_BArF}$ derived by scXRD, ellipsoids at 50% probability, anions and hydrogen atoms omitted for clarity.

Atom–Atom	Length [Å]
Bi1–N2	2.254(3)
Bi1–C24	2.269(4)
Bi1–N1	2.341(3)
Bi1–N3	2.349(3)
F1–C32	1.343(5)
N1–C2	1.325(4)
N1–C18	1.480(4)
C1–N2	1.355(4)
C1–C6	1.422(4)
C1–C2	1.446(4)
B1–C42	1.637(5)
B1–C26	1.637(5)
B1–C50	1.637(5)
B1–C34	1.644(5)
F2–C32	1.347(4)
N2–C13	1.341(4)
C2–C3	1.435(4)
F3–C32	1.334(4)
N3–C12	1.324(4)
N3–C21	1.473(4)
C3–C4	1.360(5)
F4–C33	1.343(4)
C4–C5	1.424(5)
C4–C14	1.504(5)
F5–C33	1.336(4)
C5–C6	1.373(4)
F6–C33	1.341(5)
C6–C7	1.528(4)
C7–C8	1.523(4)
C7–C15	1.537(5)
C7–C16	1.546(5)
C8–C9	1.373(4)
C8–C13	1.421(4)
F7–C40	1.333(14)
F8–C40	1.445(13)
F9–C40	1.287(10)
F7A–C40	1.520(13)
F8A–C40	1.391(14)
F9A–C40	1.246(12)
F7B–C40	1.390(11)
F8B–C40	1.166(9)
F9B–C40	1.451(10)
C9–C10	1.426(5)
F10–C41	1.331(4)
C10–C11	1.354(5)
C10–C17	1.506(5)
F11–C41	1.345(4)
C11–C12	1.427(4)
F12–C41	1.344(5)
C12–C13	1.455(4)
F13–C48	1.348(4)
F14–C48	1.324(4)
F15–C48	1.357(4)
F16–C49	1.339(4)
F17–C49	1.343(4)
F18–C49	1.340(4)
C18–C20	1.524(5)
C18–C19	1.529(5)
F19–C56	1.370(7)
F20–C56	1.312(5)
F21–C56	1.311(6)
C21–C22	1.520(5)
C21–C23	1.521(5)
C57–F23	1.269(6)
C57–F22	1.285(5)
C57–F24	1.345(6)
C57–C54	1.501(5)
C24–C25	1.512(6)
C26–C31	1.397(5)
C26–C27	1.404(4)
C27–C28	1.402(5)
C28–C29	1.391(5)
C28–C32	1.498(5)
C29–C30	1.379(5)
C30–C31	1.397(5)
C30–C33	1.496(5)
C34–C39	1.403(4)
C34–C35	1.403(4)
C35–C36	1.390(5)
C36–C37	1.387(5)
C36–C40	1.501(5)
C37–C38	1.389(5)
C38–C39	1.389(4)
C38–C41	1.497(5)
C42–C43	1.399(4)
C42–C47	1.401(4)
C43–C44	1.391(4)
C44–C45	1.389(5)
C44–C48	1.501(5)
C45–C46	1.389(5)
C46–C47	1.396(4)

C46–C49	1.504(4)
C50–C51	1.397(5)
C50–C55	1.405(4)
C51–C52	1.394(5)
C52–C53	1.389(5)
C52–C56	1.491(6)
C53–C54	1.390(5)
C54–C55	1.392(5)
C58–C59	1.257(14)
C58–C63	1.347(13)
C58–F25	1.358(12)
C59–C60	1.408(13)
C60–C61	1.436(13)
C61–C62	1.297(14)
C62–C63	1.400(13)
C63–F26	1.358(13)

**Atom–Atom–Atom**	**Angle [°]**
N2–Bi1–C24	92.13(12)
N2–Bi1–N1	71.05(10)
C24–Bi1–N1	90.84(12)
N2–Bi1–N3	70.86(10)
C24–Bi1–N3	87.73(12)
N1–Bi1–N3	141.80(10)
C2–N1–C18	120.7(3)
C2–N1–Bi1	115.9(2)
C18–N1–Bi1	121.0(2)
N2–C1–C6	121.4(3)
N2–C1–C2	117.0(3)
C6–C1–C2	121.5(3)
C42–B1–C26	104.4(2)
C42–B1–C50	113.9(3)
C26–B1–C50	112.7(3)
C42–B1–C34	111.7(2)
C26–B1–C34	111.7(3)
C50–B1–C34	102.6(2)
C13–N2–C1	123.1(3)
C13–N2–Bi1	118.7(2)
C1–N2–Bi1	118.2(2)
N1–C2–C3	125.8(3)
N1–C2–C1	117.2(3)
C3–C2–C1	116.9(3)
C12–N3–C21	120.5(3)
C12–N3–Bi1	116.1(2)
C21–N3–Bi1	123.2(2)
C4–C3–C2	121.0(3)
C3–C4–C5	120.5(3)
C3–C4–C14	121.2(3)
C5–C4–C14	118.3(3)
C6–C5–C4	122.0(3)
C5–C6–C1	117.9(3)
C5–C6–C7	121.8(3)
C1–C6–C7	120.2(3)
C8–C7–C6	111.9(3)
C8–C7–C15	107.6(3)
C6–C7–C15	108.2(3)
C8–C7–C16	110.2(3)
C6–C7–C16	109.4(3)
C15–C7–C16	109.4(3)
C9–C8–C13	118.0(3)
C9–C8–C7	121.4(3)
C13–C8–C7	120.4(3)
C8–C9–C10	122.1(3)
C11–C10–C9	120.5(3)
C11–C10–C17	121.3(3)
C9–C10–C17	118.2(3)
C10–C11–C12	121.0(3)
N3–C12–C11	125.6(3)
N3–C12–C13	117.0(3)
C11–C12–C13	117.4(3)
N2–C13–C8	121.8(3)
N2–C13–C12	117.2(3)
C8–C13–C12	121.0(3)
N1–C18–C20	110.2(3)
N1–C18–C19	109.0(3)
C20–C18–C19	111.4(3)
N3–C21–C22	109.8(3)
N3–C21–C23	109.8(3)
C22–C21–C23	111.8(3)
F23–C57–F22	112.4(5)
F23–C57–F24	101.3(5)
F22–C57–F24	102.9(5)
F23–C57–C54	114.5(4)
F22–C57–C54	113.9(3)
F24–C57–C54	110.4(4)
C25–C24–Bi1	114.7(2)
C31–C26–C27	116.0(3)
C31–C26–B1	121.1(3)
C27–C26–B1	122.5(3)
C28–C27–C26	121.6(3)
C29–C28–C27	120.9(3)
C29–C28–C32	121.1(3)
C27–C28–C32	118.1(3)

C30–C29–C28	118.2(3)
C29–C30–C31	120.8(3)
C29–C30–C33	121.1(3)
C31–C30–C33	118.1(3)
C26–C31–C30	122.4(3)
F3–C32–F1	106.0(3)
F3–C32–F2	106.8(3)
F1–C32–F2	106.2(3)
F3–C32–C28	112.6(3)
F1–C32–C28	112.3(3)
F2–C32–C28	112.4(3)
F5–C33–F6	107.0(3)
F5–C33–F4	106.5(3)
F6–C33–F4	105.4(3)
F5–C33–C30	113.1(3)
F6–C33–C30	112.6(3)
F4–C33–C30	111.8(3)
C39–C34–C35	116.0(3)
C39–C34–B1	121.9(3)
C35–C34–B1	121.8(3)
C36–C35–C34	121.8(3)
C37–C36–C35	121.4(3)
C37–C36–C40	119.2(3)
C35–C36–C40	119.4(3)
C36–C37–C38	117.6(3)
C39–C38–C37	121.2(3)
C39–C38–C41	119.7(3)
C37–C38–C41	119.0(3)
C38–C39–C34	122.0(3)
F9–C40–F7	107.9(7)
F8B–C40–F7B	112.4(7)
F9A–C40–F8A	120.4(8)
F9–C40–F8	108.8(8)
F7–C40–F8	102.7(8)
F8B–C40–F9B	111.7(6)
F7B–C40–F9B	99.3(6)
F8B–C40–C36	116.2(5)
F9A–C40–C36	119.5(7)
F9–C40–C36	115.8(5)
F7–C40–C36	113.1(7)
F7B–C40–C36	108.9(5)
F8A–C40–C36	116.3(6)
F8–C40–C36	107.5(5)
F9B–C40–C36	106.8(5)
F9A–C40–F7A	96.1(8)
F8A–C40–F7A	86.1(8)
C36–C40–F7A	107.3(5)
F10–C41–F12	106.1(3)
F10–C41–F11	107.3(3)
F12–C41–F11	105.6(3)
F10–C41–C38	113.3(3)
F12–C41–C38	111.8(3)
F11–C41–C38	112.2(3)
C43–C42–C47	115.8(3)
C43–C42–B1	121.0(3)
C47–C42–B1	123.1(3)
C44–C43–C42	122.5(3)
C45–C44–C43	121.0(3)
C45–C44–C48	120.9(3)
C43–C44–C48	118.0(3)
C44–C45–C46	117.4(3)
C45–C46–C47	121.5(3)
C45–C46–C49	119.7(3)
C47–C46–C49	118.8(3)
C46–C47–C42	121.7(3)
F14–C48–F13	107.6(3)
F14–C48–F15	106.1(3)
F13–C48–F15	104.7(3)
F14–C48–C44	114.0(3)
F13–C48–C44	111.8(3)
F15–C48–C44	112.1(3)
F16–C49–F18	106.9(3)
F16–C49–F17	106.6(3)
F18–C49–F17	105.8(3)
F16–C49–C46	112.5(3)
F18–C49–C46	112.5(3)
F17–C49–C46	112.1(3)
C51–C50–C55	115.9(3)
C51–C50–B1	122.3(3)
C55–C50–B1	121.0(3)
C52–C51–C50	122.4(3)
C53–C52–C51	120.7(3)
C53–C52–C56	120.4(3)
C51–C52–C56	118.8(3)
C52–C53–C54	117.9(3)
C53–C54–C55	121.1(3)
C53–C54–C57	120.0(3)
C55–C54–C57	118.8(4)
C54–C55–C50	121.8(3)
F21–C56–F20	111.5(4)
F21–C56–F19	101.5(4)
F20–C56–F19	102.5(4)
F21–C56–C52	113.7(4)
F20–C56–C52	114.8(4)

F19–C56–C52	111.4(4)	C62–C61–C60	116.0(9)
C59–C58–C63	119.8(9)	C61–C62–C63	123.2(9)
C59–C58–F25	121.6(10)	C58–C63–F26	117.7(9)
C63–C58–F25	118.5(10)	C58–C63–C62	119.5(9)
C58–C59–C60	122.9(9)	F26–C63–C62	122.7(9)
C59–C60–C61	118.0(9)		

Bonds and angles to hydrogen atoms were omitted.

5

As mentioned above, the anionic methyl complex **5** is highly sensitive to trace amounts of oxygen and moisture, leading to immediate decomposition. Numerous attempts were made to obtain single crystals suitable for the acquisition of a publishable dataset; however, the dark red crystals obtained from THF/hexane decomposed immediately upon removal from the glovebox. We managed to obtain a dataset that allowed us to confirm the proposed structure, with the methyl group directly bound to the bismuth center. The presence of the $[Li(12c4)_2]^+$ cation, as well as co-crystallized THF, could also be confirmed. We refrain from discussing structural parameters further, as the overall quality of the dataset is insufficient. Nevertheless, the observed atom connectivity is fully consistent with the results obtained from NMR spectroscopy and elemental analysis.

Temperature [K]	101.00
Crystal system	triclinic
Space group (number)	$P\overline{1}$ (2)
a [Å]	20.607(3)
b [Å]	21.215(3)
c [Å]	23.196(4)
α [°]	90.365(5)
β [°]	105.481(5)
γ [°]	110.943(5)
Volume [Å^3]	9069(2)
Z	12
ρ_{calc} [gcm^{-3}]	1.387
μ [mm^{-1}]	3.931
F(000)	3841.827
Crystal size [mm^3]	0.1×0.05×0.03
Crystal color	Dark red
Crystal shape	
Radiation	Mo K_α (λ=0.71073 Å)
2θ range [°]	4.00 to 50.88 (0.83 Å)
Index ranges	−24 ≤ h ≤ 24<br>−25 ≤ k ≤ 25<br>−27 ≤ l ≤ 27
Reflections collected	225078
Independent reflections	33373<br>R_{int} = 0.0643<br>R_{sigma} = 0.0362
Completeness to θ = 25.2417°	100.0 %
Data / Restraints / Parameters	33373 / 410 / 1638
Absorption correction T_{min}/T_{max} (method)	0.6826 / 0.7452 (multi-scan)
Goodness-of-fit on F^2	1.0031
Final *R* indexes [$I \geq 2\sigma(I)$]	R_1 = 0.1261<br>wR_2 = 0.3027
Final *R* indexes [all data]	R_1 = 0.1492<br>wR_2 = 0.3208
Largest peak/hole [eÅ^{-3}]	20.36/−12.36

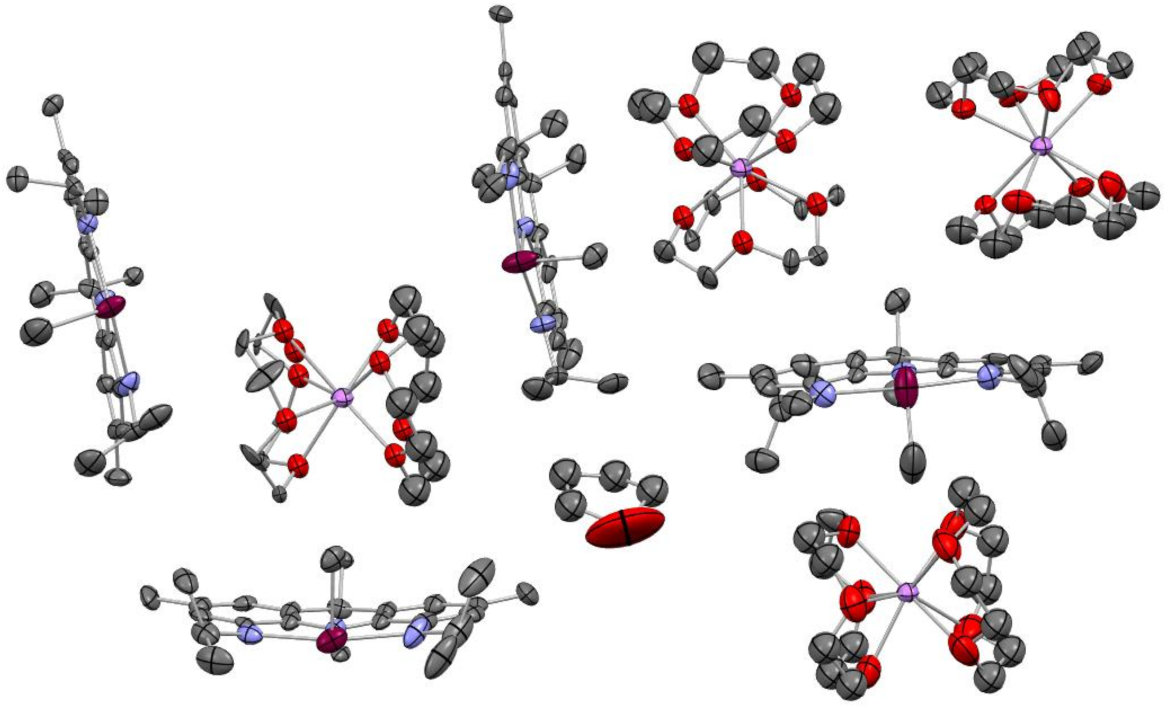

Figure S86. Asymmetric unit of **5**, ellipsoids at 50% probability, hydrogen atoms omitted for clarity.

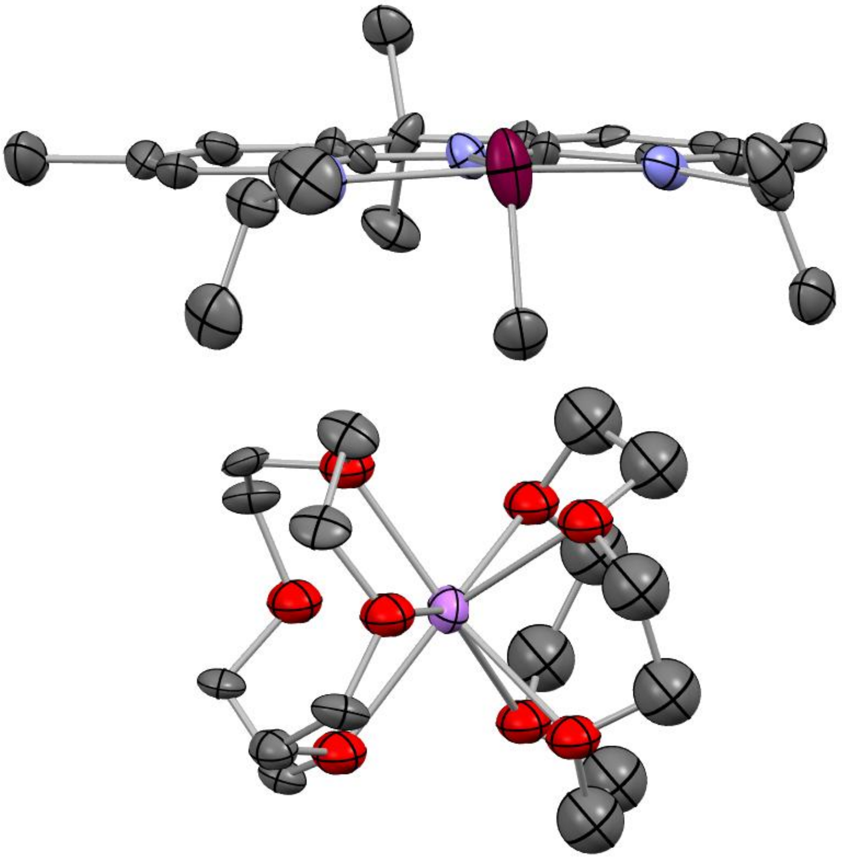

Figure S87. Confirmation of atom connectivity of **5**, ellipsoids at 50% probability, hydrogen atoms omitted for clarity.

6

CCDC number	2497817
Empirical formula	$C_{97}H_{165}Bi_2K_2N_{10}O_{14.50}$
Formula weight	2199.54
Temperature [K]	100(2)
Crystal system	triclinic
Space group (number)	$P\overline{1}$ (2)
a [Å]	13.0852(10)
b [Å]	20.1093(15)
c [Å]	20.8439(12)
α [°]	105.636(2)
β [°]	99.009(2)
γ [°]	90.725(3)
Volume [Å^3]	5207.9(6)
Z	2
ρ_{calc} [gcm^{-3}]	1.403
μ [mm^{-1}]	3.517
F(000)	2274
Crystal size [mm^3]	0.050×0.200×0.200
Crystal colour	red
Crystal shape	block
Radiation	MoK_α (λ=0.71073 Å)
2θ range [°]	3.89 to 50.79 (0.83 Å)
Index ranges	−15 ≤ h ≤ 15<br>−24 ≤ k ≤ 24<br>−25 ≤ l ≤ 24
Reflections collected	117794
Independent reflections	19137<br>R_{int} = 0.0565<br>R_{sigma} = 0.0355
Completeness to θ = 25.242°	99.9 %
Data / Restraints / Parameters	19137 / 224 / 1224
Absorption correction T_{min}/T_{max} (method)	0.4907 / 0.7452 (multi-scan)
Goodness-of-fit on F^2	1.062
Final *R* indexes [*I*≥2σ(*I*)]	R_1 = 0.0303<br>wR_2 = 0.0729
Final *R* indexes [all data]	R_1 = 0.0381<br>wR_2 = 0.0775
Largest peak/hole [eÅ^{-3}]	3.07/−1.27

Atom–Atom	Length [Å]	Atom–Atom	Length [Å]
N1–C2	1.370(4)	C7–C15	1.546(5)
N1–C18	1.455(5)	C7–C16	1.547(5)
N1–Bi1	2.319(3)	O8–C71	1.421(5)
C1–N2	1.394(4)	O8–C70	1.428(5)
C1–C6	1.397(5)	O8–K1	2.849(3)
C1–C2	1.429(5)	N8–C72	1.462(5)
N2–C13	1.393(5)	N8–C60	1.473(5)
N2–Bi1	2.177(3)	N8–C66	1.475(5)
C2–C3	1.408(5)	N8–K1	3.047(3)
O3–C57	1.429(5)	C8–C13	1.389(5)
O3–C56	1.433(5)	C8–C9	1.409(5)
O3–K1	2.822(3)	O9–C74	1.423(5)
N3–C12	1.376(5)	O9–C75	1.426(5)
N3–C21	1.463(5)	O9–K2	2.831(3)
N3–Bi1	2.294(3)	N9–C84	1.467(5)
C3–C4	1.400(5)	N9–C90	1.472(5)
O4–C59	1.422(5)	N9–C78	1.487(5)
O4–C58	1.430(5)	N9–K2	2.994(3)
O4–K1	2.791(3)	C9–C10	1.380(6)
N4–C29	1.365(6)	O10–C76	1.420(5)
N4–C45	1.461(5)	O10–C77	1.435(5)
N4–Bi2	2.312(4)	O10–K2	2.892(3)
C4–C5	1.381(5)	N10–C79	1.466(5)
C4–C14	1.513(5)	N10–C73	1.468(5)
O5–C63	1.418(6)	N10–C85	1.475(5)
O5–C62	1.428(6)	N10–K2	2.975(3)
O5–K1	2.778(3)	C10–C11	1.394(6)
N5–C28	1.397(5)	C10–C17	1.518(5)
N5–C40	1.402(5)	O11–C81	1.422(5)
N5–Bi2	2.166(3)	O11–C80	1.426(5)
C5–C6	1.407(5)	O11–K2	2.811(3)
O6–C65	1.412(6)	Bi1–O1	2.154(2)
O6–C64	1.434(5)	O1–C24	1.423(4)
O6–K1	2.818(3)	C11–C12	1.403(5)
N6–C39	1.367(5)	O12–C83	1.421(4)
N6–C48	1.464(5)	O12–C82	1.430(4)
N6–Bi2	2.326(3)	O12–K2	2.764(3)
C6–C7	1.527(5)	C12–C13	1.434(5)
O7–C68	1.420(5)	O13–C86	1.430(5)
O7–C69	1.424(5)	O13–C87	1.431(5)
O7–K1	2.796(3)	O13–K2	2.835(3)
N7–C61	1.461(6)	O14–C88	1.422(5)
N7–C55	1.472(5)	O14–C89	1.425(5)
N7–C67	1.475(6)	O14–K2	2.897(3)
N7–K1	3.032(4)	C18–C20	1.524(5)
C7–C8	1.534(5)	C18–C19	1.525(6)
		C21–C23	1.524(6)

C21–C22	1.525(6)
Bi2–O2	2.137(3)
O2–C51	1.411(5)
C24–C26	1.526(5)
C24–C27	1.526(5)
C24–C25	1.534(5)
C28–C33	1.388(6)
C28–C29	1.433(6)
C29–C30	1.414(6)
C30–C31	1.391(7)
C31–C32	1.378(7)
C31–C41	1.519(6)
C32–C33	1.406(6)
C33–C34	1.525(6)
C34–C35	1.520(6)
C34–C43	1.539(5)
C34–C42	1.550(5)
C35–C40	1.389(5)
C35–C36	1.409(5)
C36–C37	1.381(6)
C37–C38	1.389(6)
C37–C44	1.515(5)
C38–C39	1.410(5)
C39–C40	1.431(5)
C45–C47	1.513(7)
C45–C46	1.537(6)
C48–C49	1.517(6)
C48–C50	1.533(5)
C51–C54	1.530(6)
C51–C52	1.531(6)
C51–C53	1.533(6)
C55–C56	1.504(6)
C57–C58	1.490(6)
C59–C60	1.503(6)
C61–C62	1.509(8)
C63–C64	1.484(8)
C65–C66	1.521(7)
C67–C68	1.501(7)
C69–C70	1.477(6)
C71–C72	1.496(6)
C73–C74	1.506(6)
C75–C76	1.490(6)
C77–C78	1.488(6)
C79–C80	1.505(6)
C81–C82	1.493(5)
C83–C84	1.505(5)
C85–C86	1.505(6)
C87–C88	1.502(6)
C89–C90	1.492(6)
O15–C1A	1.405(12)
O15–C1D	1.436(14)
C1A–C1B	1.518(11)
C1B–C1C	1.524(12)
C1C–C1D	1.516(11)
C1E–C1F	1.583(13)
C1F–C1G	1.555(13)
C1G–C1H	1.567(13)
C1H–C1I	1.625(13)
C1J–C1K	1.585(13)
C1K–C1L	1.531(12)
C1L–C1M	1.503(12)
C1M–C1N	1.546(13)

**Atom–Atom–Atom**	**Angle [°]**
C2–N1–C18	118.2(3)
C2–N1–Bi1	116.0(2)
C18–N1–Bi1	124.6(2)
N2–C1–C6	122.4(3)
N2–C1–C2	116.6(3)
C6–C1–C2	121.0(3)
C13–N2–C1	118.8(3)
C13–N2–Bi1	118.8(2)
C1–N2–Bi1	119.3(2)
N1–C2–C3	126.8(3)
N1–C2–C1	115.3(3)
C3–C2–C1	117.9(3)
C57–O3–C56	111.9(3)
C57–O3–K1	114.9(2)
C56–O3–K1	114.1(2)
C12–N3–C21	118.2(3)
C12–N3–Bi1	115.5(2)
C21–N3–Bi1	125.1(2)
C4–C3–C2	121.2(3)
C59–O4–C58	112.2(3)
C59–O4–K1	120.5(2)
C58–O4–K1	110.6(2)
C29–N4–C45	118.0(4)
C29–N4–Bi2	115.5(3)
C45–N4–Bi2	126.0(3)
C5–C4–C3	119.4(3)
C5–C4–C14	120.5(3)
C3–C4–C14	120.1(3)
C63–O5–C62	112.5(4)

C63–O5–K1	111.9(2)
C62–O5–K1	122.4(3)
C28–N5–C40	118.5(3)
C28–N5–Bi2	119.3(2)
C40–N5–Bi2	119.2(2)
C4–C5–C6	121.7(3)
C65–O6–C64	112.2(4)
C65–O6–K1	115.1(2)
C64–O6–K1	114.3(3)
C39–N6–C48	117.8(3)
C39–N6–Bi2	114.7(2)
C48–N6–Bi2	125.2(2)
C1–C6–C5	118.6(3)
C1–C6–C7	121.9(3)
C5–C6–C7	119.4(3)
C68–O7–C69	110.5(3)
C68–O7–K1	116.4(3)
C69–O7–K1	115.5(2)
C61–N7–C55	111.1(4)
C61–N7–C67	111.1(4)
C55–N7–C67	109.8(4)
C61–N7–K1	106.6(3)
C55–N7–K1	111.2(2)
C67–N7–K1	106.9(2)
C6–C7–C8	111.1(3)
C6–C7–C15	108.7(3)
C8–C7–C15	109.1(3)
C6–C7–C16	109.0(3)
C8–C7–C16	109.6(3)
C15–C7–C16	109.3(3)
C71–O8–C70	112.0(3)
C71–O8–K1	119.1(2)
C70–O8–K1	114.6(2)
C72–N8–C60	110.7(3)
C72–N8–C66	108.9(3)
C60–N8–C66	110.2(3)
C72–N8–K1	109.4(2)
C60–N8–K1	105.8(2)
C66–N8–K1	111.9(3)
C13–C8–C9	119.0(3)
C13–C8–C7	121.6(3)
C9–C8–C7	119.5(3)
C74–O9–C75	111.7(3)
C74–O9–K2	119.3(2)
C75–O9–K2	117.5(2)
C84–N9–C90	110.6(3)
C84–N9–C78	110.1(3)
C90–N9–C78	108.7(3)
C84–N9–K2	105.7(2)
C90–N9–K2	111.8(2)
C78–N9–K2	109.9(2)
C10–C9–C8	121.3(4)
C76–O10–C77	111.1(3)
C76–O10–K2	111.9(2)
C77–O10–K2	115.4(2)
C79–N10–C73	109.3(3)
C79–N10–C85	110.7(3)
C73–N10–C85	109.6(3)
C79–N10–K2	110.0(2)
C73–N10–K2	109.4(2)
C85–N10–K2	107.9(2)
C9–C10–C11	119.5(4)
C9–C10–C17	120.3(4)
C11–C10–C17	120.2(4)
C81–O11–C80	110.5(3)
C81–O11–K2	113.9(2)
C80–O11–K2	115.2(2)
O1–Bi1–N2	110.43(10)
O1–Bi1–N3	96.23(10)
N2–Bi1–N3	72.49(11)
O1–Bi1–N1	94.87(10)
N2–Bi1–N1	71.81(10)
N3–Bi1–N1	144.28(11)
O5–K1–O4	122.01(9)
O5–K1–O7	104.31(9)
O4–K1–O7	126.73(8)
O5–K1–O6	61.19(10)
O4–K1–O6	85.22(8)
O7–K1–O6	144.20(9)
O5–K1–O3	84.43(9)
O4–K1–O3	61.21(8)
O7–K1–O3	102.70(9)
O6–K1–O3	107.68(9)
O5–K1–O8	129.81(9)
O4–K1–O8	101.22(8)
O7–K1–O8	59.79(8)
O6–K1–O8	102.54(9)
O3–K1–O8	142.89(8)
O5–K1–N7	59.94(10)
O4–K1–N7	120.02(9)
O7–K1–N7	61.27(9)
O6–K1–N7	120.57(10)
O3–K1–N7	59.52(9)
O8–K1–N7	120.46(10)

O5–K1–N8	119.55(10)	O11–K2–O14	109.31(8)
O4–K1–N8	60.51(9)	O9–K2–O14	141.57(8)
O7–K1–N8	118.78(9)	O13–K2–O14	59.38(8)
O6–K1–N8	58.97(9)	O10–K2–O14	100.84(8)
O3–K1–N8	120.94(9)	O12–K2–N10	120.78(8)
O8–K1–N8	59.49(9)	O11–K2–N10	60.97(8)
N7–K1–N8	179.37(10)	O9–K2–N10	60.27(9)
C24–O1–Bi1	129.3(2)	O13–K2–N10	60.93(9)
C10–C11–C12	121.5(4)	O10–K2–N10	118.36(9)
C83–O12–C82	111.6(3)	O14–K2–N10	118.99(9)
C83–O12–K2	119.2(2)	O12–K2–N9	61.56(8)
C82–O12–K2	114.9(2)	O11–K2–N9	121.21(8)
N3–C12–C11	126.4(3)	O9–K2–N9	119.14(9)
N3–C12–C13	115.9(3)	O13–K2–N9	117.41(9)
C11–C12–C13	117.7(3)	O10–K2–N9	60.60(8)
C86–O13–C87	111.0(3)	O14–K2–N9	59.81(8)
C86–O13–K2	119.0(2)	N10–K2–N9	177.62(9)
C87–O13–K2	114.6(2)	C51–O2–Bi2	132.9(3)
C8–C13–N2	123.1(3)	O1–C24–C26	110.3(3)
C8–C13–C12	120.9(3)	O1–C24–C27	111.2(3)
N2–C13–C12	116.0(3)	C26–C24–C27	109.3(3)
C88–O14–C89	109.5(3)	O1–C24–C25	106.6(3)
C88–O14–K2	115.4(2)	C26–C24–C25	109.7(3)
C89–O14–K2	113.5(2)	C27–C24–C25	109.8(3)
N1–C18–C20	110.1(3)	C33–C28–N5	123.0(3)
N1–C18–C19	112.6(3)	C33–C28–C29	121.1(4)
C20–C18–C19	109.7(3)	N5–C28–C29	115.8(4)
N3–C21–C23	111.5(3)	N4–C29–C30	126.8(4)
N3–C21–C22	110.5(3)	N4–C29–C28	115.9(4)
C23–C21–C22	109.7(4)	C30–C29–C28	117.2(4)
O2–Bi2–N5	109.59(11)	C31–C30–C29	121.8(4)
O2–Bi2–N4	97.51(11)	C32–C31–C30	119.0(4)
N5–Bi2–N4	72.13(12)	C32–C31–C41	121.2(5)
O2–Bi2–N6	94.62(11)	C30–C31–C41	119.8(4)
N5–Bi2–N6	72.36(11)	C31–C32–C33	122.1(4)
N4–Bi2–N6	144.49(12)	C28–C33–C32	118.7(4)
O12–K2–O11	60.97(7)	C28–C33–C34	121.2(4)
O12–K2–O9	124.50(8)	C32–C33–C34	120.1(4)
O11–K2–O9	102.62(8)	C35–C34–C33	111.6(3)
O12–K2–O13	130.80(8)	C35–C34–C43	108.4(3)
O11–K2–O13	91.61(8)	C33–C34–C43	109.2(3)
O9–K2–O13	99.67(8)	C35–C34–C42	109.6(3)
O12–K2–O10	102.54(8)	C33–C34–C42	109.2(3)
O11–K2–O10	145.01(8)	C43–C34–C42	108.8(3)
O9–K2–O10	59.34(8)	C40–C35–C36	118.1(4)
O13–K2–O10	119.50(8)	C40–C35–C34	121.7(3)
O12–K2–O14	90.23(8)	C36–C35–C34	120.1(4)

C37–C36–C35	122.0(4)	O7–C68–C67	109.3(4)
C36–C37–C38	119.2(4)	O7–C69–C70	110.0(3)
C36–C37–C44	120.7(4)	O8–C70–C69	108.9(3)
C38–C37–C44	120.1(4)	O8–C71–C72	109.4(4)
C37–C38–C39	121.9(4)	N8–C72–C71	115.1(4)
N6–C39–C38	126.3(4)	N10–C73–C74	114.1(3)
N6–C39–C40	116.6(3)	O9–C74–C73	109.0(3)
C38–C39–C40	117.0(4)	O9–C75–C76	108.9(3)
C35–C40–N5	122.4(3)	O10–C76–C75	110.1(3)
C35–C40–C39	121.8(3)	O10–C77–C78	110.1(3)
N5–C40–C39	115.8(3)	N9–C78–C77	114.6(3)
N4–C45–C47	110.3(4)	N10–C79–C80	113.3(3)
N4–C45–C46	112.7(4)	O11–C80–C79	109.4(3)
C47–C45–C46	110.0(4)	O11–C81–C82	109.8(3)
N6–C48–C49	110.7(3)	O12–C82–C81	108.9(3)
N6–C48–C50	111.4(3)	O12–C83–C84	109.7(3)
C49–C48–C50	109.6(3)	N9–C84–C83	113.9(3)
O2–C51–C54	112.7(3)	N10–C85–C86	114.2(3)
O2–C51–C52	108.4(3)	O13–C86–C85	110.2(3)
C54–C51–C52	108.9(4)	O13–C87–C88	109.3(3)
O2–C51–C53	107.1(4)	O14–C88–C87	109.7(3)
C54–C51–C53	109.3(4)	O14–C89–C90	109.8(3)
C52–C51–C53	110.4(3)	N9–C90–C89	113.1(3)
N7–C55–C56	112.6(3)	C1A–O15–C1D	108.8(9)
O3–C56–C55	108.4(3)	O15–C1A–C1B	108.7(9)
O3–C57–C58	108.9(3)	C1A–C1B–C1C	101.7(10)
O4–C58–C57	110.0(3)	C1D–C1C–C1B	102.1(9)
O4–C59–C60	109.8(3)	O15–C1D–C1C	107.1(9)
N8–C60–C59	113.8(3)	C1G–C1F–C1E	100.0(12)
N7–C61–C62	113.6(4)	C1F–C1G–C1H	107.9(12)
O5–C62–C61	109.3(4)	C1G–C1H–C1I	94.2(12)
O5–C63–C64	109.4(4)	C1L–C1K–C1J	103.1(13)
O6–C64–C63	110.4(4)	C1M–C1L–C1K	125.5(17)
O6–C65–C66	108.9(4)	C1L–C1M–C1N	118.4(17)
N8–C66–C65	111.5(4)		
N7–C67–C68	114.7(4)		

Bonds and angles to hydrogen atoms were omitted.

EPR Spectroscopy

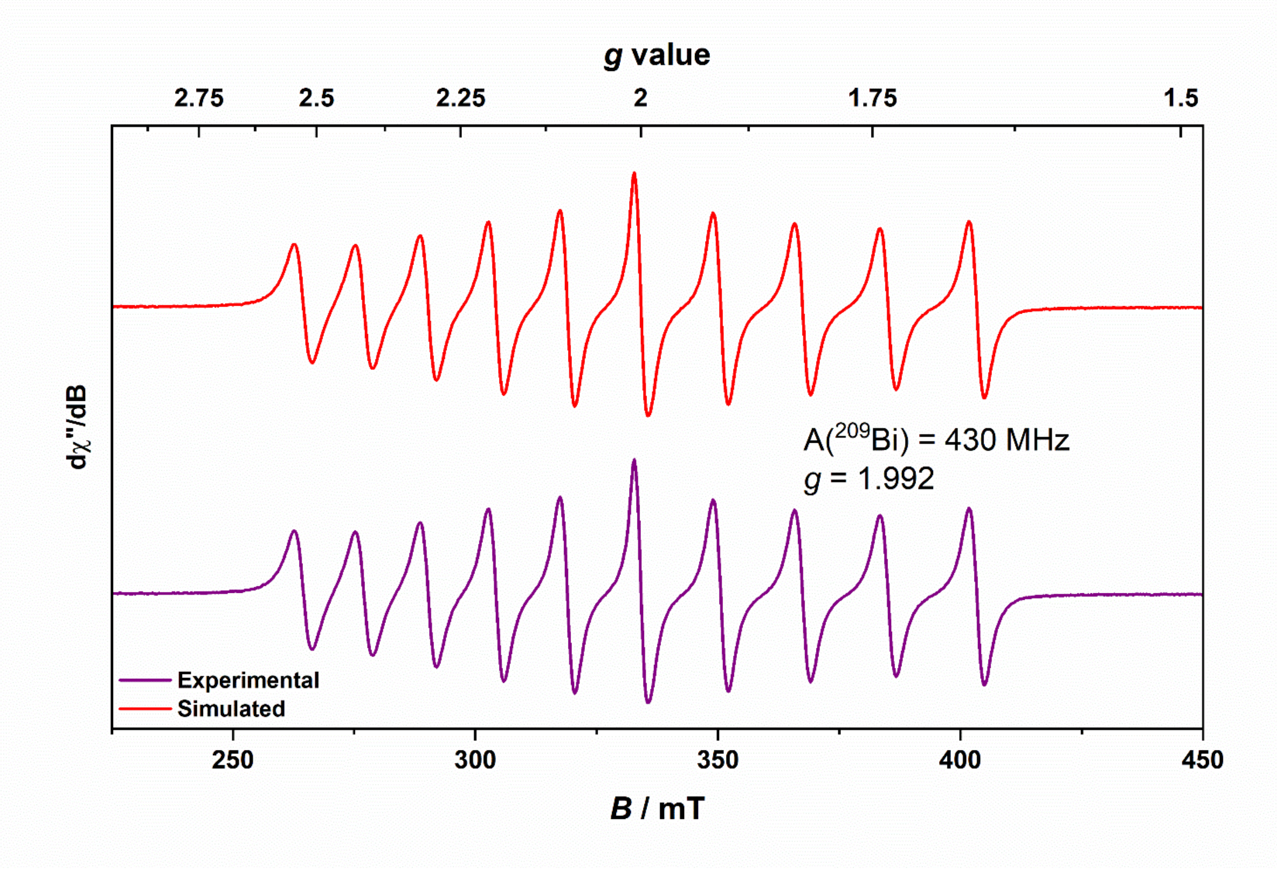

Figure S88. EPR spectrum of **4** in toluene, 25 °C.

Experimental details: perpendicular mode, 9.352159 GHz, 1 mW microwave power, 3 G modulation amplitude. Simulation parameters: g_x = 1.96, g_y = 2.02, g_z = 1.99 (g_{iso} = 1.99), A_x = 448 MHz, A_y = 467 MHz, A_z = 374 MHz (A_{iso} = 430 MHz).

X-ray Absorption Spectroscopy

Table S1. Characteristic XAS-energies for **2** and **3** and the reference $BiPh_3$.

Compound	L_1-edge / eV	L_1-whiteline/ eV	L_3-edge/ eV	L_3-whiteline/ eV
$\mathbf{1}^{Bi}$	16388.1	16393.8	13417.7	13439.0
$\mathbf{3}^{BArF}$	16388.1	16393.8	13418.6	13437.3
$BiPh_3$[16,17]	16388.5	16397.5	13412.0	13442.0

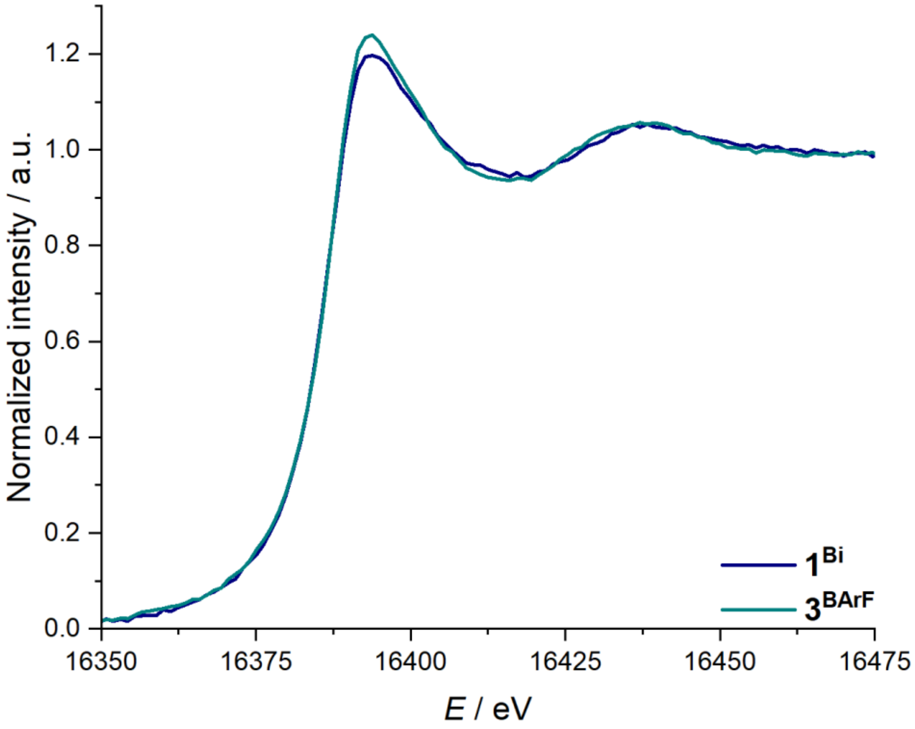

Figure S89. Bi L_1-edge XANES spectra of $\mathbf{1}^{Bi}$ and $\mathbf{3}^{BArF}$.

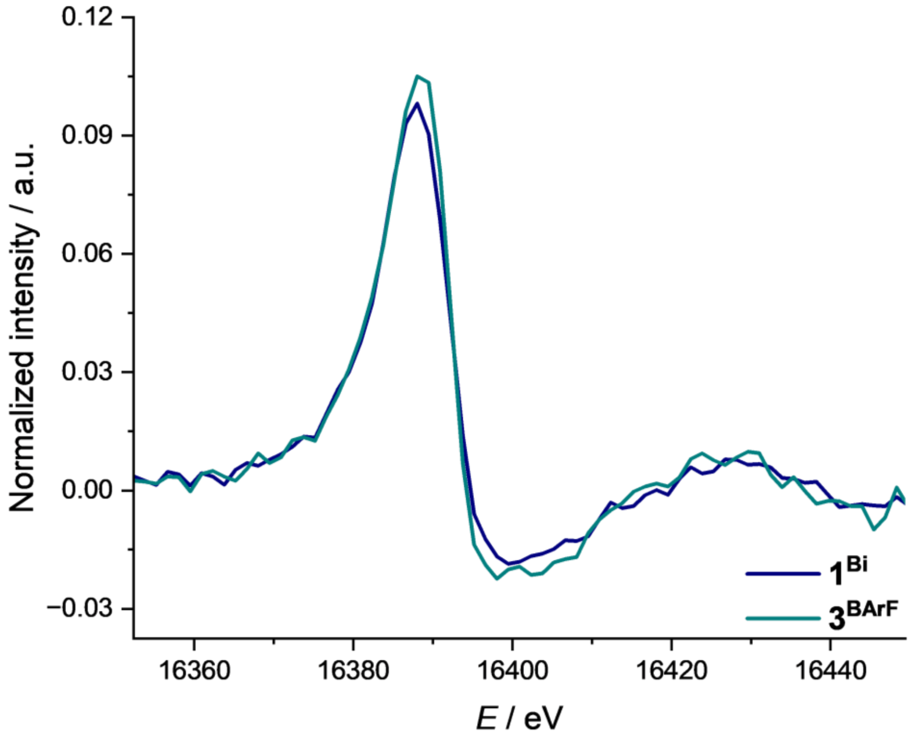

Figure S90. First derivative of the Bi L_1-edge XANES spectra of **1**^{Bi} and **3**^{BArF}.

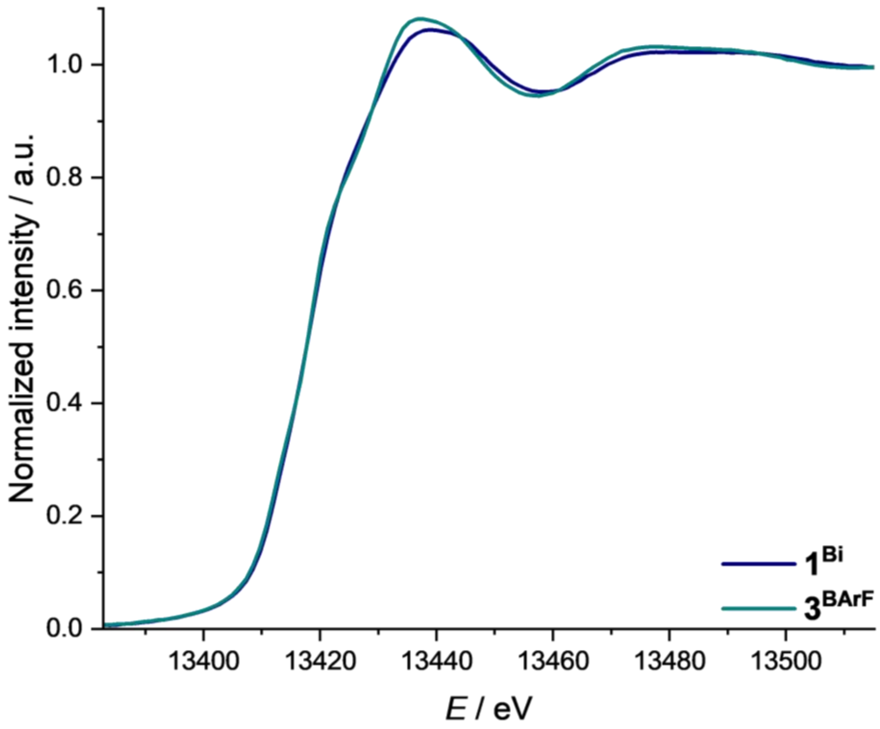

Figure S91. Bi L_3-edge XANES spectra of **1**^{Bi} and **3**^{BArF}.

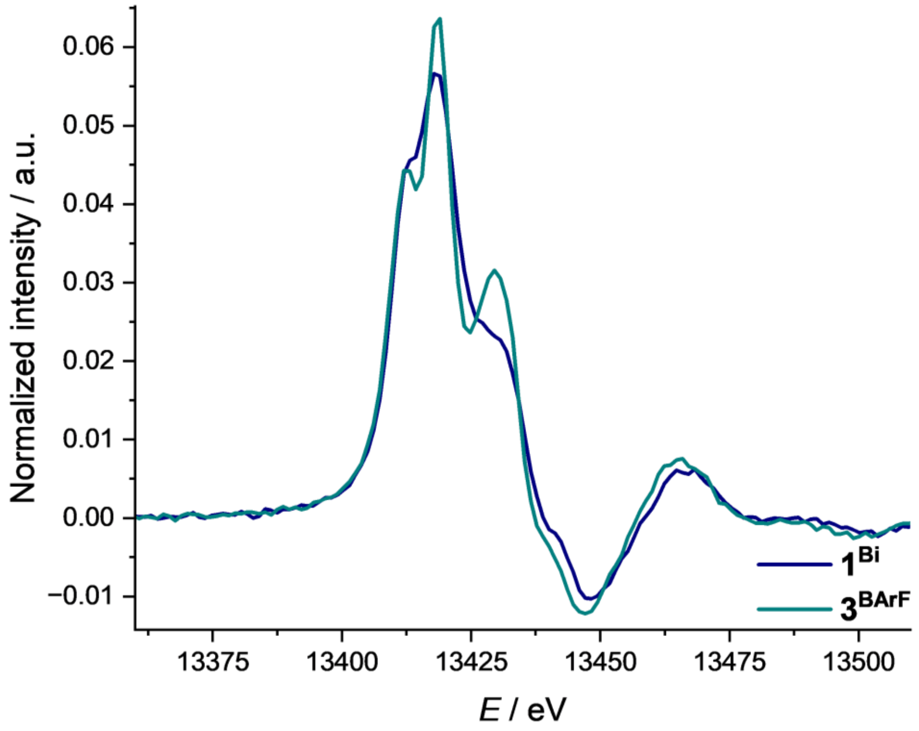

Figure S92. First derivative of the Bi L_3-edge XANES spectra of $\mathbf{1}^{\mathbf{Bi}}$ and $\mathbf{3}^{\mathbf{BArF}}$.

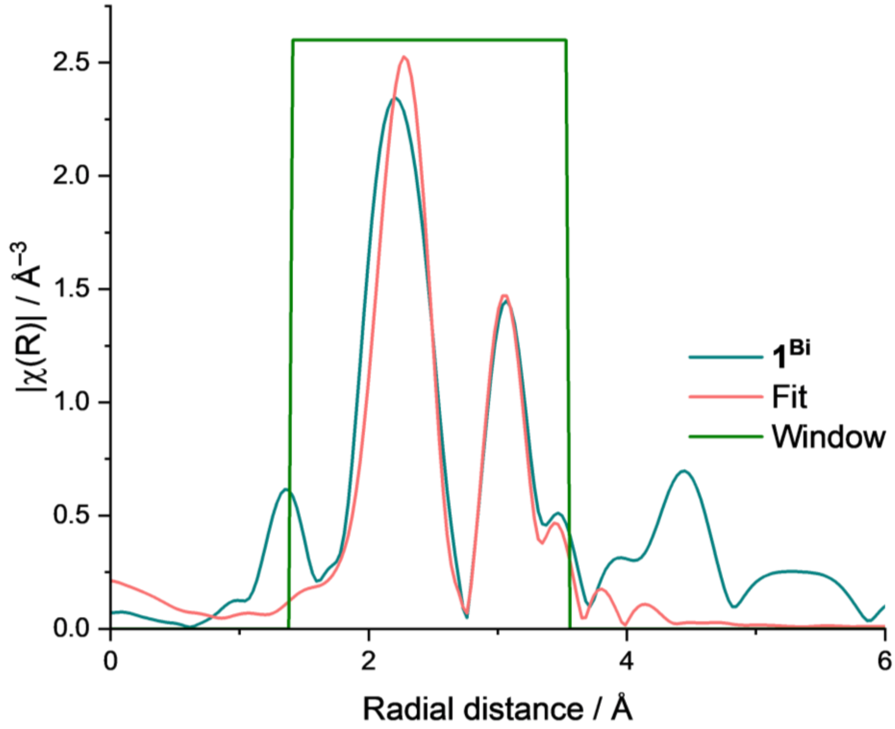

Figure S93. Bi L_3-edge EXAFS spectrum and fit of $\mathbf{1}^{\mathbf{Bi}}$.

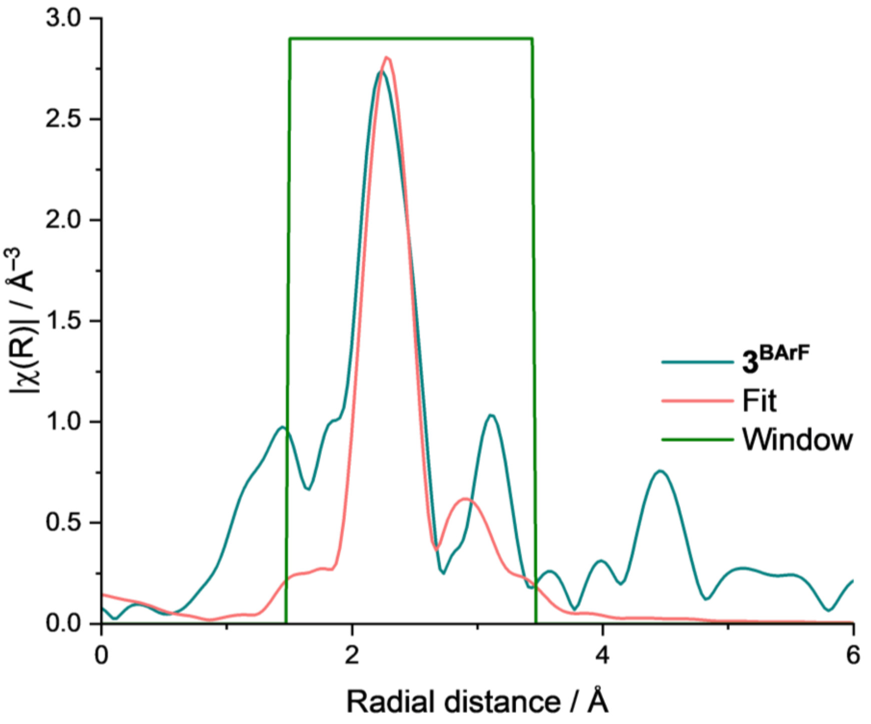

Figure S94. Bi L_3-edge EXAFS spectra and fit of $\mathbf{3^{BArF}}$.

		Scattering path	Type	N	σ^2 (Å^2)	R_{eff} (Å)	R_{fit} (Å)	delr (Å)
Fit of structure $\mathbf{1^{Bi}}$								
		Bi...N2.1	Single scattering	1	0.00527	2.1729	2.1651	-0.0077
**S_O^2**	1.10	Bi...N3.1	Single scattering	2	0.00527	2.2980	2.2902	-0.0077
**E_0**	3.05	Bi...C14.1	Single scattering	2	0.00423	3.0854	3.1242	0.0388
**R-factor**	0.018	Bi...C21.1	Single scattering	2	0.00423	3.1321	3.1709	0.0388
**Red. χ^2**	13.76	Bi...N2.1...C14.1	Obtuse triangle	4	0.00187	3.3130	3.4948	0.1818
		Bi...C18.1	Obtuse triangle	2	0.00432	3.3482	3.3871	0.0388
Fit of structure $\mathbf{3^{BArF}}$								
		Bi...C1.1	Single scattering	1	0.00671	2.2570	2.2403	-0.0166
**S_O^2**	0.99	Bi...N2.1	Single scattering	1	0.00671	2.2704	2.2537	-0.0166
**E_0**	-0.15	Bi...N1.1	Single scattering	2	0.00671	2.3383	2.3216	-0.0166
**R-factor**	0.019	Bi...C1.1	Single scattering	4	0.01996	3.1421	3.1037	-0.0383
**Red. χ^2**	46.25	Bi...C13.1	Single scattering	2	0.01966	3.3211	3.2827	-0.0383
		Bi...C11.1	Single scattering	2	0.01966	3.3693	3.3309	-0.0383

Results from Bi L_3-edge EXAFS fitted parameters, for each scattering path, including degeneracy (**N**), the half path length (**D**), which in case of single scattering paths it is called interatomic distance (**R_{eff}**), the mean square relative displacement (MSRD), also known as EXAFS Debye-Waller factor (**σ^2**), the amplitude reduction factor **S_O^2**, and **E_0**.

In both cases a Hanning-type window with dk = 4 was chosen to perform the Fourier transformation (FT). FT was performed over the k-space range between 2 to 12 Å^{-1} for samples **1** and $\mathbf{3^{BArF}}$. The R-range for the fit between calculated structures and samples was between 1.4 and 3.55 Å, and between 1.5 and 3.45 Å for samples $\mathbf{1^{Bi}}$ and $\mathbf{3^{BArF}}$, respectively.

Density Functional Theory Calculations

Conformer sampling was realized *via* the *Conformer–Rotamer Ensemble Sampling Tool* (CREST)[18] while geometry optimizations and electronic structure calculations were performed with the ORCA quantum chemistry software package, version 5.0.4[19,20]. Solvent effects were included on an implicit level *via* the Conductor-like Polarizable Continuum Model (CPCM) as implemented in ORCA 5.0.4.[21] All molecular structures were optimized on a Kohn-Sham density functional theory (DFT) level of theory *via* the TPSSh hybrid functional[22,23], Ahlrichs basis sets[24,25] specified below and the D4 dispersion correction.[26,27] The zeroth-order regular approximation (ZORA) scheme was applied for bismuth containing compounds to take into account scalar relativistic effects.[28] Computational UV/Vis spectra, frontier molecular orbitals and natural transition orbitals were obtain *via* the CAM-B3LYP range-separated hybrid functional[29] and Ahlrichs basis sets.[24,25] Energetic comparison of planar and bent $\mathbf{1}^{\mathbf{P}}$ conformers was based on high-level domain based local pair-natural orbital coupled cluster with singles, doubles and perturbative triples (DLPNO-CCSD(T)) calculations[30,31] employing Ahlrichs basis sets.[24,25]

$\mathbf{1}^{\mathbf{P}}$

Conformer Search and Comparison to Bent Structure

Conformers of the phosphine were sampled *via* CREST: GFN2-xTB and implicit solvation model for hexane (generalized Born (GB) model with surface area (SA), GBSA). Ten distinct conformers were found in an energy window of 6 kcal/mol. Six energetically most favorable structures were reoptimized *via* TPSSh/Def2-TZVP (ORCA 5.0.4) with a conductor-like polarizable continuum model (CPCM) for hexane and dispersion correction (D3). Relative to CREST results, mainly the reorientation of two ^{i}Pr-groups close to the P-atom lead to structural relaxation. Energy changes are observed to be significant since CREST-optimized structures have been selected for thermally accessible energies with max. energy 0.703 kcal/mol = 245 cm^{-1} = 353 K. The central phosphine moiety of $\mathbf{1}^{\mathbf{P}}$ is nearly planar with a dihedral angle CNPN = 179.9 °. The dihedral angle increases slightly compared to CREST results due to "repulsion" of rotated ^{i}Pr-groups.

The planar $\mathbf{1}^{\mathbf{P}}$-conformer was energetically compared to the bent $\mathbf{1}^{\mathbf{Pbent}}$-conformer discussed by Goicoechea, (*Chem. Eur. J.* **2024**, *30*, e202400624) for a gas phase scenario. In this publication, optimized structures were reported on a ZORA-ωB97X(D3)/Def2-TZVPP level of theory. A frequency calculation on the planar structure of this study, as characterized by a dihedral angle CNPN=161.5° (compared to CNPN=179.9° in this work), reveals an imaginary mode at 19 cm^{-1} on this level of theory. Energetic comparison of the planar structures relative to the bent conformer based on DLPNO-CCSD(T)/Def2-TZVPP level of theory gives $E(\mathbf{1}^{\mathbf{P}})$ = 0.81 kcal/mol and $E(\mathbf{1}^{\mathbf{P\_literature}})$ = 1.072 kcal/mol with a difference beyond chemical accuracy of 1 kcal/mol. However, optimization of $\mathbf{1}^{\mathbf{P}}$ *via* ZORA-ωB97X(D3)/Def2-TZVPP did not lead to the bent conformer in contrast to $\mathbf{1}^{\mathbf{P\_literature}}$, which in combination with the frequency calculation above suggest a potential transition state character of $\mathbf{1}^{\mathbf{P\_literature}}$.

1^P^

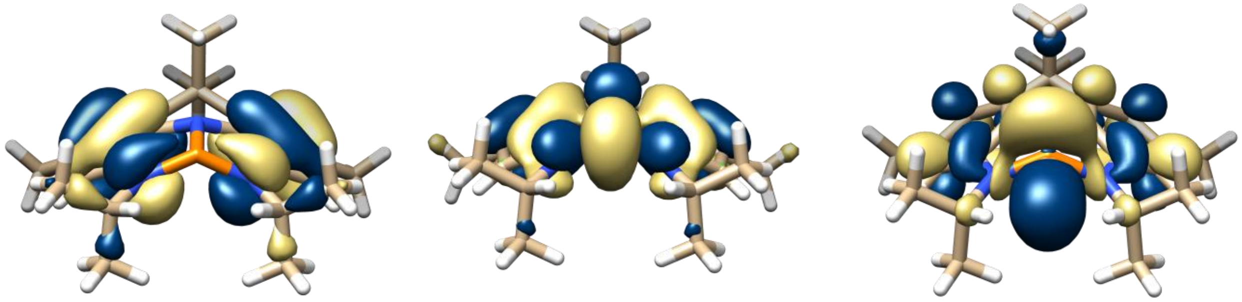

Figure S95. Frontier molecular orbitals of the bent conformation of **1**^{P} with HOMO–1 (left), HOMO (center) and LUMO (right) at iso-value 0.03 obtained with ORCA 5.0.4 at TDDFT/CAM-B3LYP/Def2-TZVP/CPCM(Hexane) level of theory. Color code: H in white, C in beige, N in blue, P in orange.

Table S2. Selected orbital energies of **1**^{Pbent}.

Orbital	Energy / Hartree	Energy / eV
**HOMO–1**	-0.252110	-6.8603
**HOMO**	-0.237754	-6.4696
**LUMO**	0.015405	0.4192

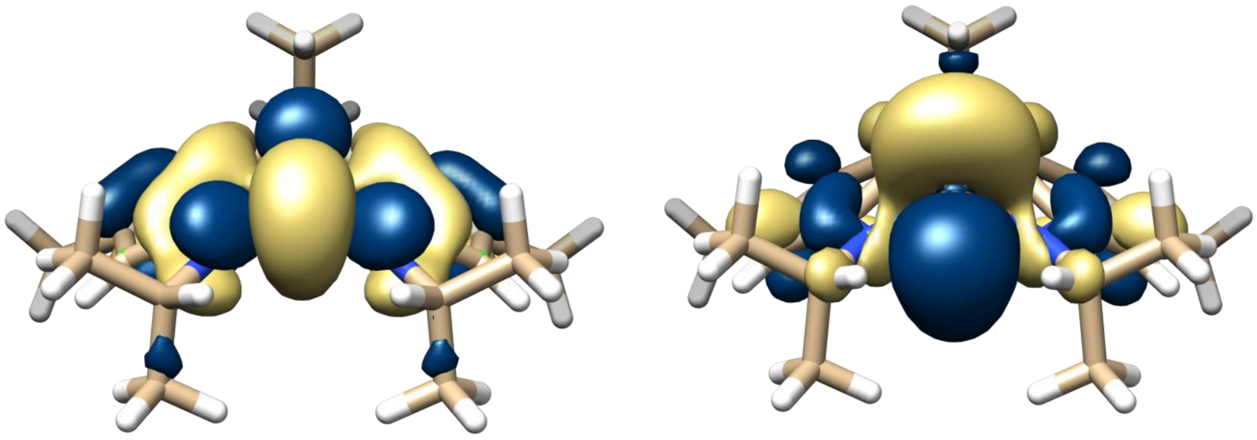

Figure S96. Dominant natural transition orbitals for S1 transition (381 nm) of the bent conformation of **1**^{P} with HOMO (left) and LUMO (right) at iso-value 0.03 obtained with ORCA 5.0.4 at TDDFT/CAM-B3LYP/Def2-TZVP/CPCM(Hexane) level of theory. Color code: H in white, C in beige, N in blue, P in orange.

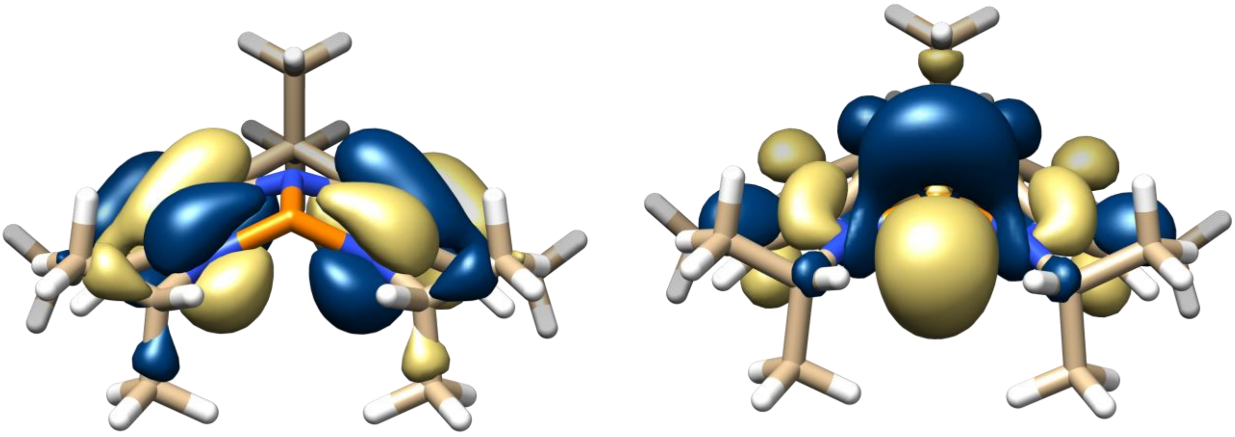

Figure S97. Dominant natural transition orbitals for S3 transition (316 nm) of the bent conformation of **1**^{P} with HOMO-1 (left) and LUMO (right) at iso-value 0.03 obtained with ORCA 5.0.4 at TDDFT/CAM-B3LYP/Def2-TZVP/CPCM(Hexane) level of theory. Color code: H in white, C in beige, N in blue, P in orange.

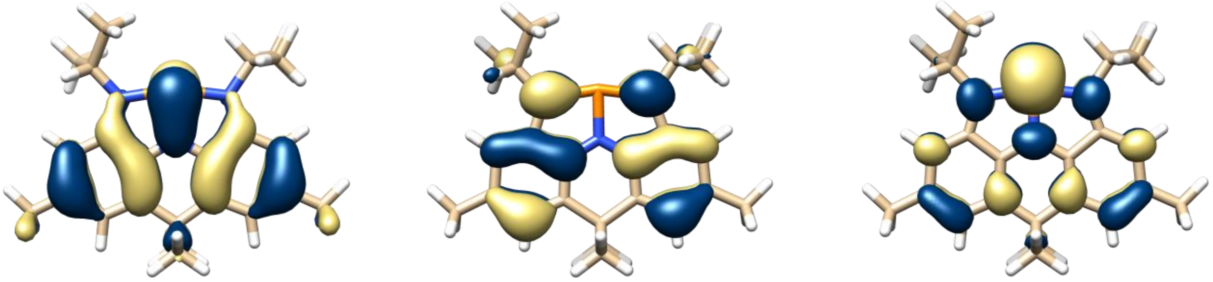

Figure S98. Frontier molecular orbitals of the T-shaped phosphine complex **1**^{P} with HOMO–1 (left, –0.17429 H), HOMO (center) and LUMO (right) at iso-value 0.03 obtained with ORCA 5.0.4 at TDDFT/CAM-B3LYP/Def2-TZVP/CPCM(Hexane) level of theory. Color code: H in white, C in beige, N in blue, P in orange.

Table S3. Selected orbital energies of **1**^{P}.

Orbital	Energy / Hartree	Energy / eV
**HOMO–1**	-0.256879	-6.9900
**HOMO**	-0.236576	-6.4376
**LUMO**	-0.028427	-0.7735

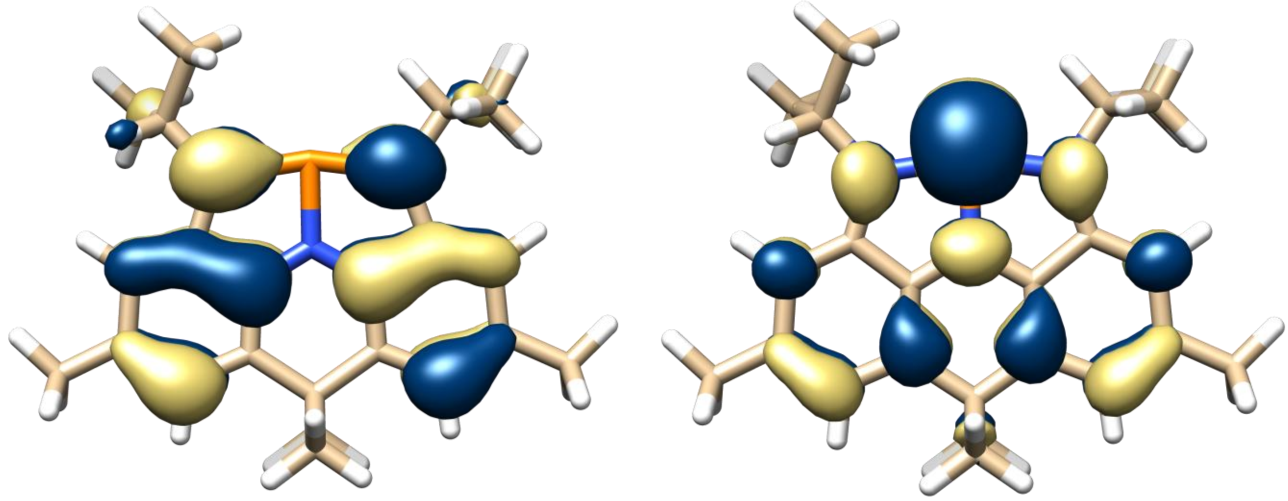

Figure S99. Dominant natural transition orbitals for S1 transition (381 nm) of the T-shaped phosphine complex **1**^{P} with HOMO (left) and LUMO (right) at iso-value 0.03 obtained with ORCA 5.0.4 at TDDFT/CAM-B3LYP/Def2-TZVP/CPCM(Hexane) level of theory. Color code: H in white, C in beige, N in blue, P in orange.

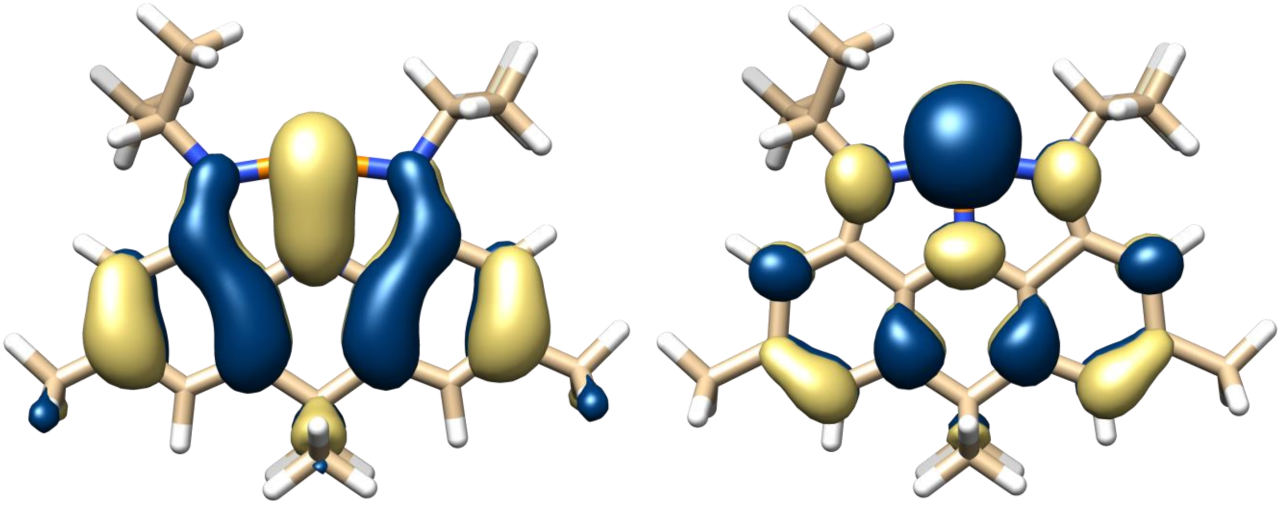

Figure S100. Dominant natural transition orbitals for S3 transition (316 nm) of the T-shaped phosphine complex **1**^{P} with HOMO–1 (left) and LUMO (right) at iso-value 0.03 obtained with ORCA 5.0.4 at TDDFT/CAM-B3LYP/Def2-TZVP/CPCM(Hexane) level of theory. Color code: H in white, C in beige, N in blue, P in orange.

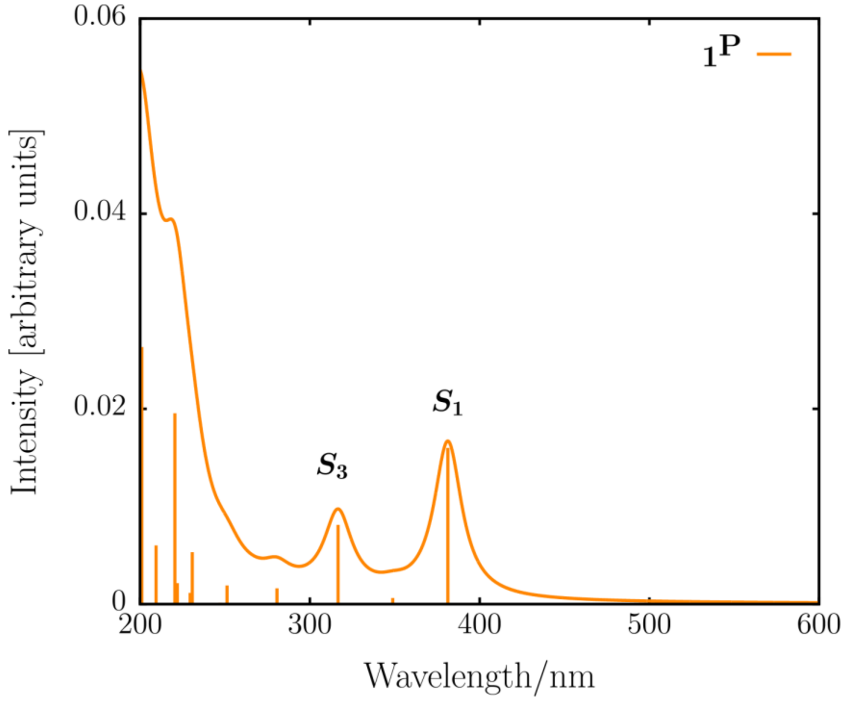

Figure S101. Computational UV/Vis spectrum of the T-shaped phosphine complex **1**^{P} obtained with ORCA 5.0.4 at TDDFT/CAM-B3LYP/Def2-TZVP/CPCM(hexane) level of theory.

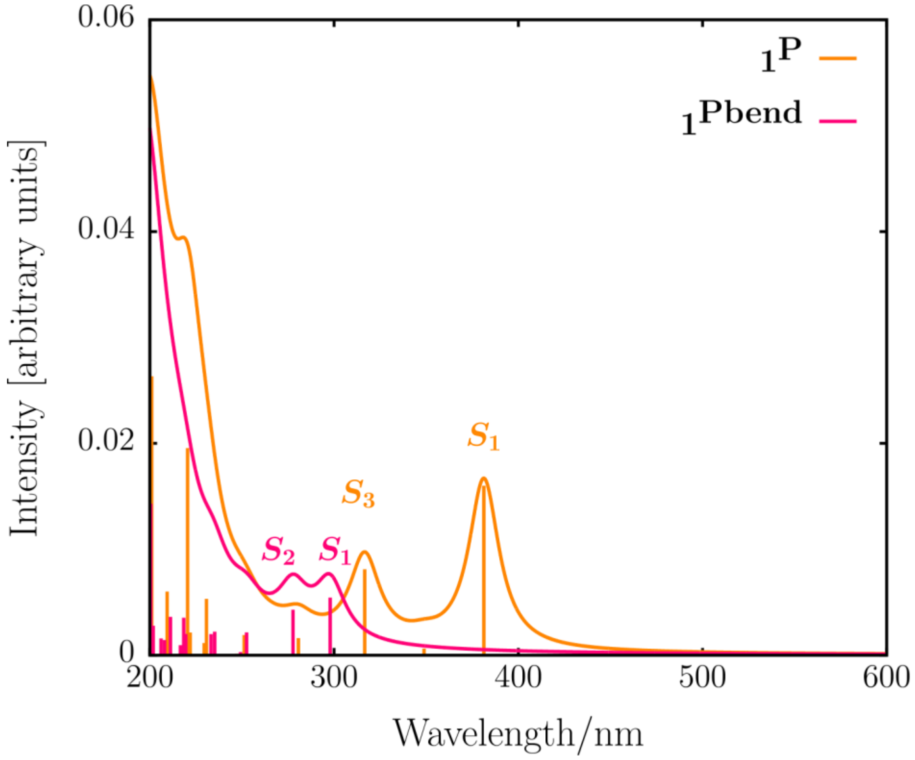

Figure S102. Computational UV/Vis spectrum of the T-shaped phosphine complex in planar (**1**^{P}) and bent (**1**^{Pbent}) conformations obtained with ORCA 5.0.4 at TDDFT/CAM-B3LYP/Def2-TZVP/CPCM(Hexane) level of theory.

1^{Bi}

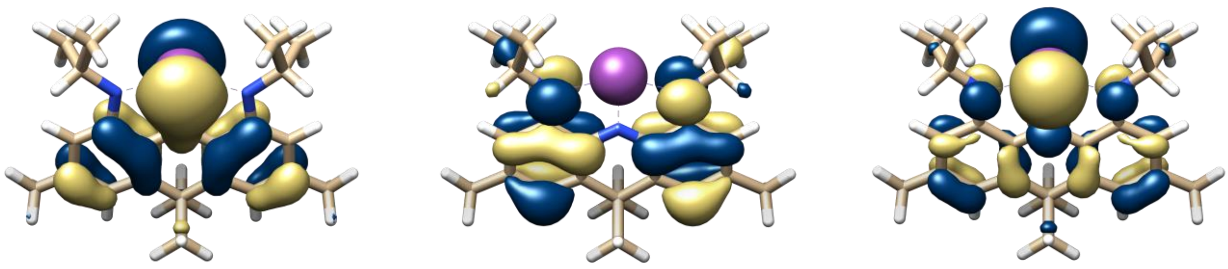

Figure S103. Frontier molecular orbitals of the T-shaped bismuth complex **1^{Bi}** with HOMO–1 (left), HOMO (center) and LUMO (right) at iso-value 0.03 obtained with ORCA 5.0.4 TDDFT/ZORA-CAM-B3LYP(D4)/Def2-TZVPP/CPCM(Hexane) level of theory. Color code: H in white, C in beige, N in blue, Bi in purple.

Table S4. Selected orbital energies of **1^{Bi}**.

Orbital	Energy / Hartree	Energy / eV
**HOMO–1**	-0.2279	-6.203
**HOMO**	-0.2218	-6.036
**LUMO**	-0.0541	-1.473

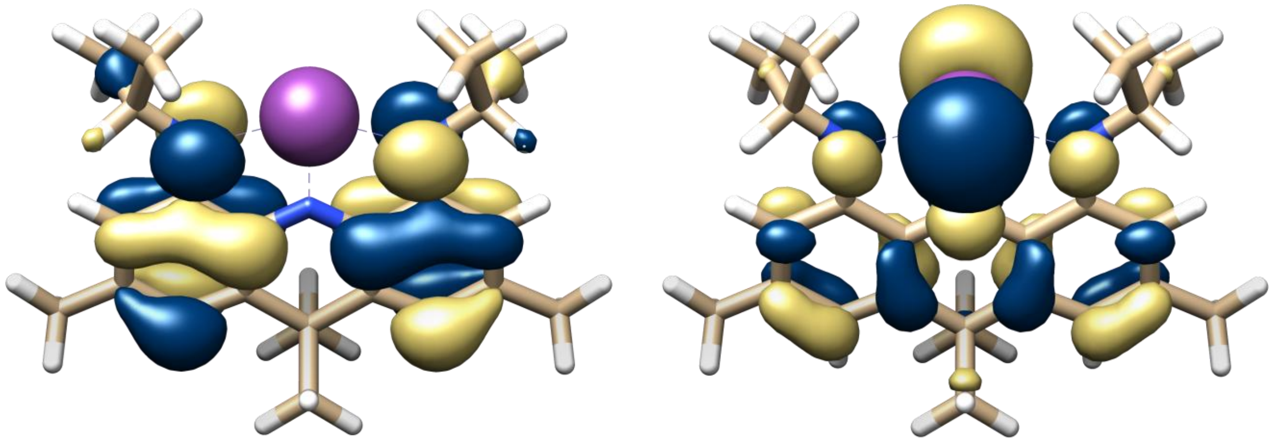

Figure S104. Dominant natural transition orbitals for S1 transition (501 nm) of the T-shaped bismuth complex **1^{Bi}** with HOMO (left) and LUMO (right) at iso-value 0.03 obtained with ORCA 5.0.4 at TDDFT/ZORA-CAM-B3LYP(D4)/Def2-TZVPP/CPCM(Hexane) level of theory. Color code: H in white, C in beige, N in blue, Bi in purple.

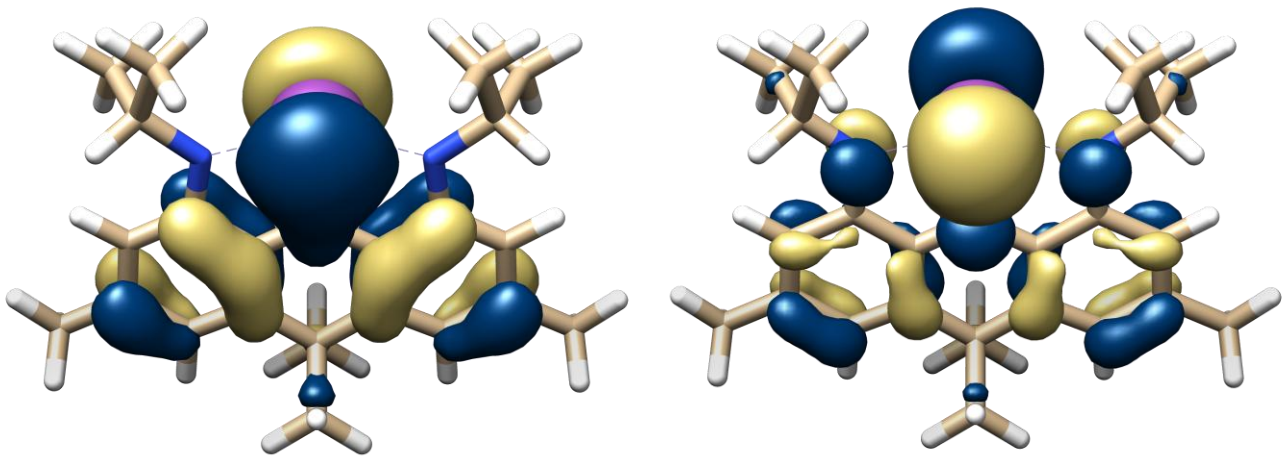

Figure S105. Dominant natural transition orbitals for S2 transition (441 nm) of the T-shaped bismuth complex **1**$^{\mathbf{Bi}}$ with HOMO–1 (left) and LUMO (right) at iso-value 0.03 obtained with ORCA 5.0.4 at TDDFT/ZORA-CAM-B3LYP(D4)/Def2-TZVPP/CPCM(Hexane) level of theory. Color code: H in white, C in beige, N in blue, Bi in purple.

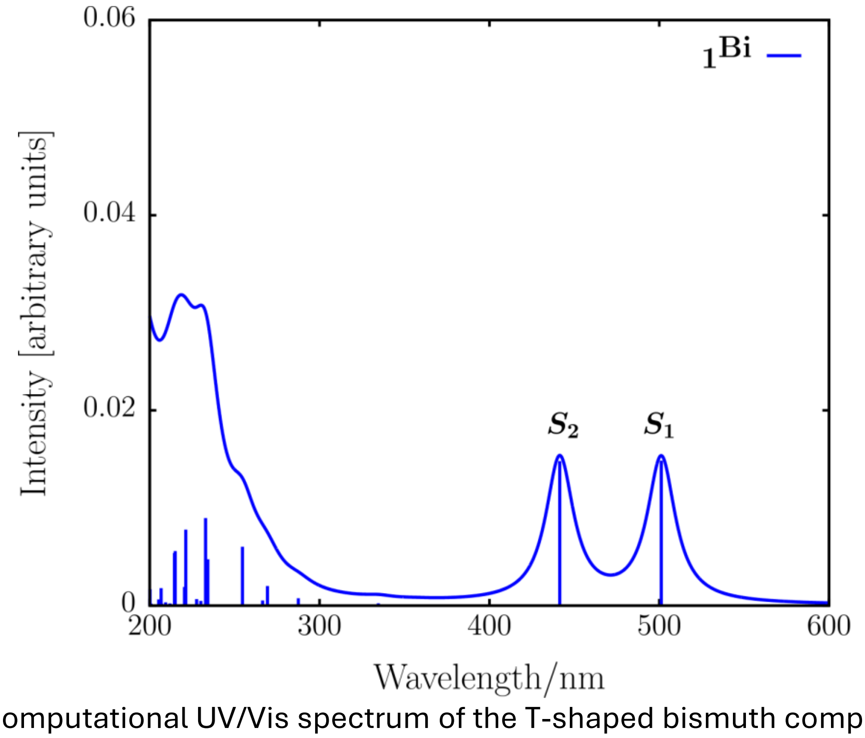

Figure S106. Computational UV/Vis spectrum of the T-shaped bismuth complex **1**$^{\mathbf{Bi}}$ obtained with ORCA 5.0.4 at TDDFT/ZORA-CAM-B3LYP/Def2-TZVPP/CPCM(Hexane) level of theory.

Comparison Between 1^{Bi} and 1^{Bi_TMS}

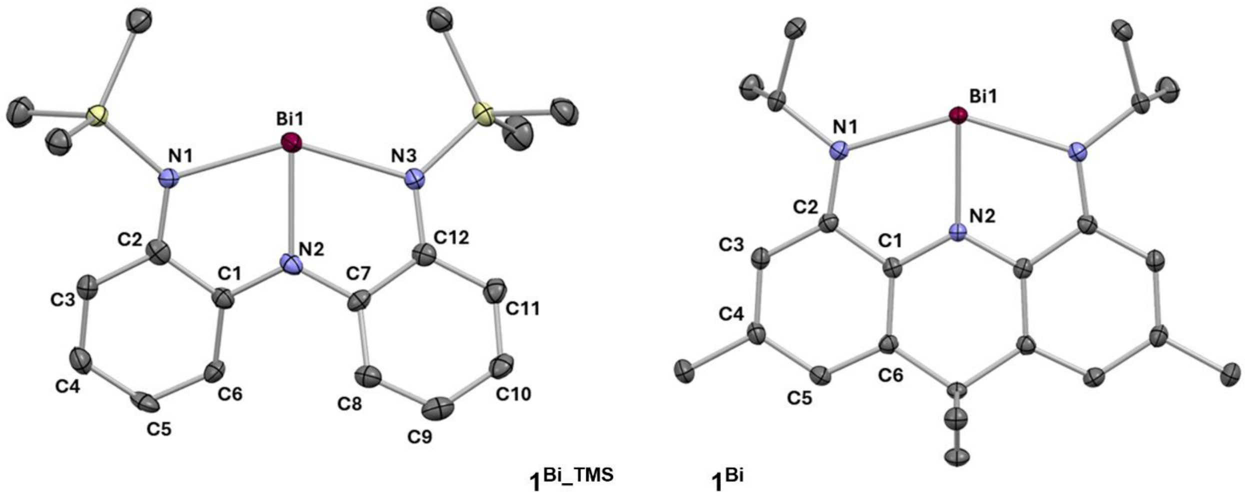

	1^{Bi_TMS}	1^{Bi}
Bi1–N1	2.303(4)	2.300(2)
Bi1–N2	2.181(4)	2.168(3)
N1–C2	1.363(7)	1.352(2)
N2–C1	1.383(6)	1.372(2)
C1–C2	1.428(6)	1.431(2)
C2–C3	1.421(7)	1.420(3)
C3–C4	1.373(7)	1.382(2)
C4–C5	1.391(6)	1.408(3)
C5–C6	1.372(7)	1.388(3)
C6–C1	1.407(7)	1.411(3)
Bi1–N3	2.282(3)	
N3–C12	1.362(7)	
N2–C7	1.388(6)	
C7–C8	1.408(8)	
C8–C9	1.368(6)	
C9–C10	1.395(8)	
C10–C11	1.364(8)	
C11–C12	1.421(6)	
C12–C7	1.426(7)	

Figure S107. Comparison of bond lengths of 1^{Bi} and 1^{Bi_TMS} derived by scXRD.

1^{Bi} and 1^{Bi_TMS} both represent planarized Bi(NNN) compounds. The main difference is that the latter features an untethered ligand which results in an overall non-planar structure due to a twist within the ligand backbone. Nevertheless, the Bi–N bond distances as well as the C–N and C–C bond lengths of both ligands reveal distinct similarities. While 1^{Bi} features equivalent Bi–N bonds to the flanking nitrogens, the scXRD derived molecular structure of 1^{Bi_TMS} is slightly asymmetric which might be a result of the twist angle within the ligand backbone. The longer of these two bonds is identical to the flanking Bi–N bonds of 1^{Bi} (2.300 Å), whereas the other bond is ~0.018 Å shorter. The Bi–N bond to the central nitrogen is slightly shorter in 1^{Bi}. Both ligands feature clear signs of partial ligand oxidation due to extensive delocalization of electron

density to the vacant Bi(6p) orbital as evidenced by comparably short N1–C2 and N3–C12 bonds. In addition, the C3–C4 and C5–C6 bonds feature significantly contracted bond lengths when compared to the C1–C2 and C4–C5 bonds. These effects appear slightly more pronounced in $\mathbf{1}^{\mathrm{Bi}}$

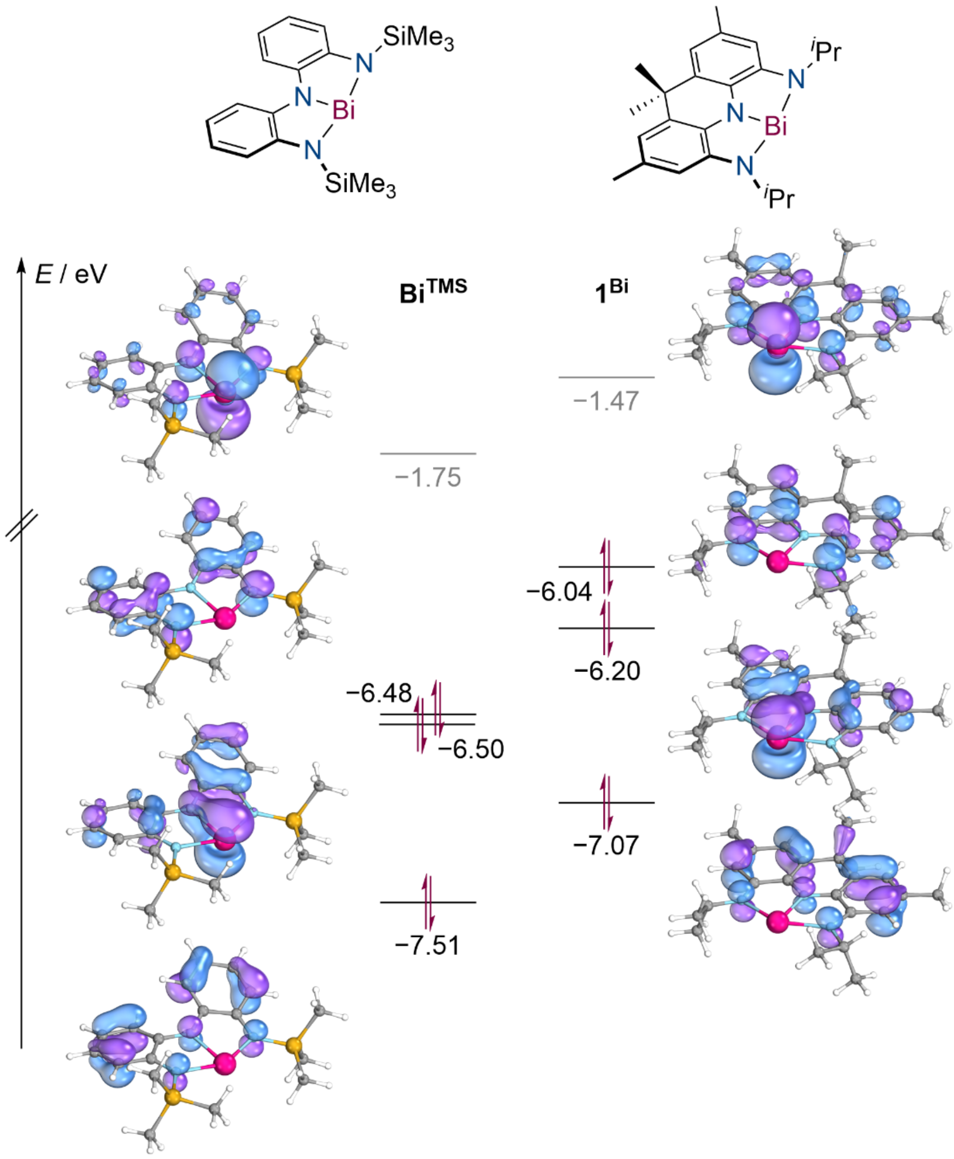

Figure S108. Frontier molecular orbitals of $\mathbf{1}^{\mathrm{Bi\_TMS}}$ and $\mathbf{1}^{\mathrm{Bi}}$ obtained with ORCA 5.0.4 at TDDFT/ZORA-CAM-B3LYP(D4)/Def2-TZVPP/CPCM(hexane) level of theory. Color code: H in white, C in grey, N in blue, Bi in purple, Si in orange.

Both complexes display pronounced similarities between their frontier molecular orbitals. The similar HOMO-LUMO gaps as well as their deep blue colors and performed TDDFT calculations show that the order and character of the frontier molecular orbitals in both compounds is basically the same.[32] The relative energy level and inter-orbital energetic difference however appears to be highly ligand dependent. Interestingly, the HOMO−1/LUMO energetic difference in both compounds is almost identical.

2

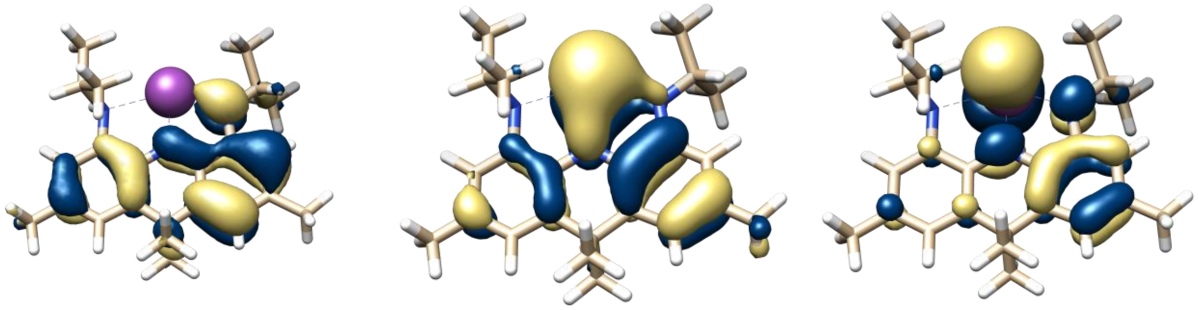

Figure S109. Frontier molecular orbitals of the protonated T-shaped bismuth(III) complex **2** with HOMO–1 (left), HOMO (center) and LUMO (right) at iso-value 0.03 obtained with ORCA 5.0.4 at TDDFT/ZORA-CAM-B3LYP(D4)/Def2-TZVPP/CPCM(DCM) level of theory. Color code: H in white, C in beige, N in blue, Bi in purple.

Table S5. Selected orbital energies of **2**.

Orbital	Energy / Hartree	Energy / eV
**HOMO–1**	-0.279841	-7.610
**HOMO**	-0.259644	-7.064
**LUMO**	-0.092483	-2.517

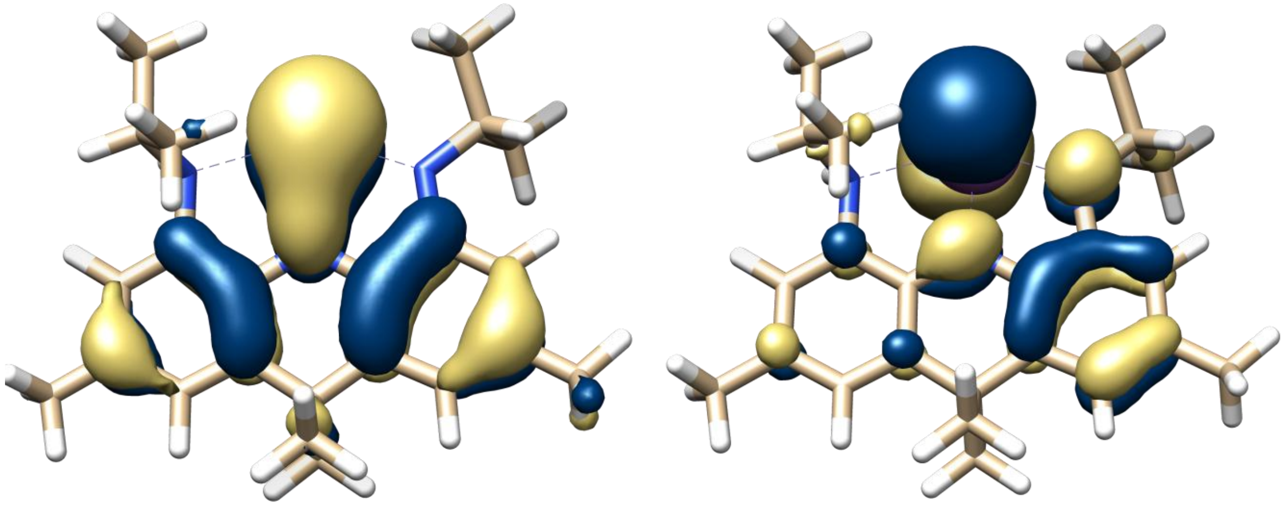

Figure S110. Dominant natural transition orbitals for S1 transition (467 nm) of the protonated T-shaped bismuth(III) complex **2** with HOMO (left) and LUMO (right) at iso-value 0.03 obtained with ORCA 5.0.4 at TDDFT/ZORA-CAM-B3LYP(D4)/Def2-TZVPP/CPCM(DCM) level of theory. Color code: H in white, C in beige, N in blue, Bi in purple.

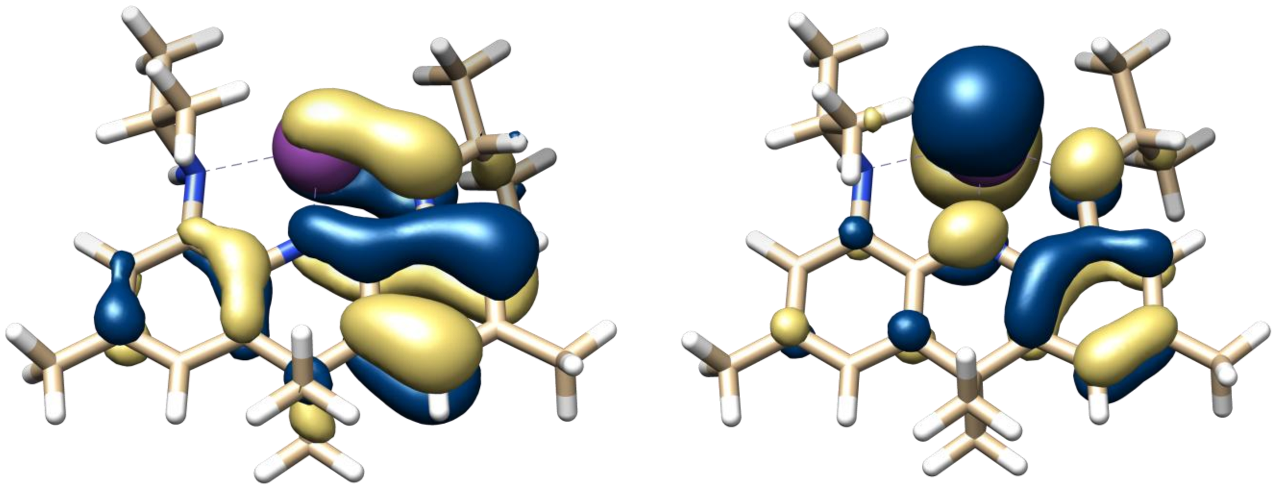

Figure S111. Dominant natural transition orbitals for S2 transition (417 nm) of the protonated T-shaped bismuth(III) complex **2** with HOMO–1 (left) and LUMO (right) at iso-value 0.03 obtained with ORCA 5.0.4 at TDDFT/ZORA-CAM-B3LYP(D4)/Def2-TZVPP/CPCM(DCM) level of theory. Color code: H in white, C in beige, N in blue, Bi in purple.

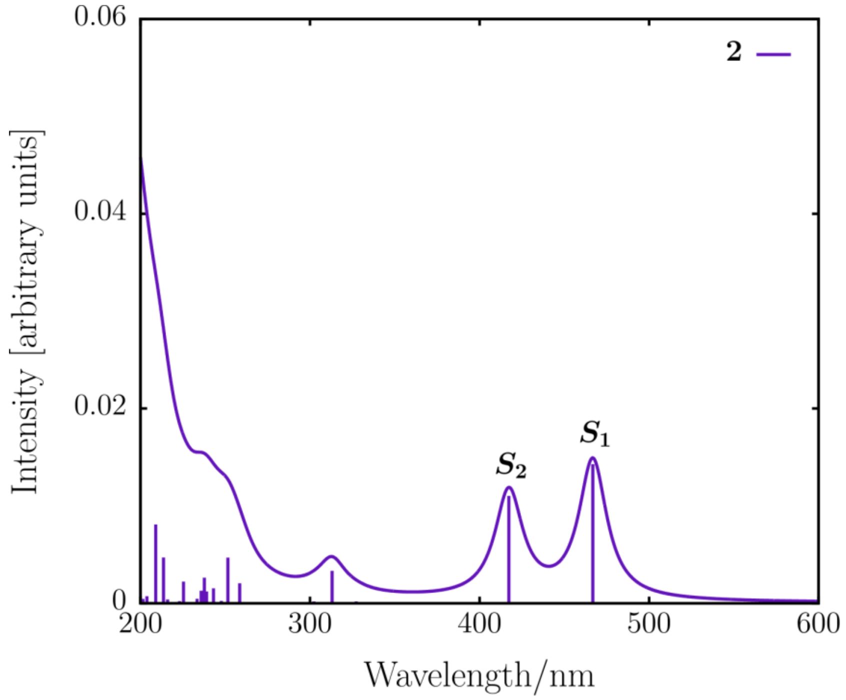

Figure S112. Computational UV/Vis spectrum of the protonated T-shaped bismuth(III) complex **2** obtained with ORCA 5.0.4 at TDDFT/ZORA-CAM-B3LYP(D4)/Def2-TZVPP/CPCM(DCM) level of theory.

3⁺

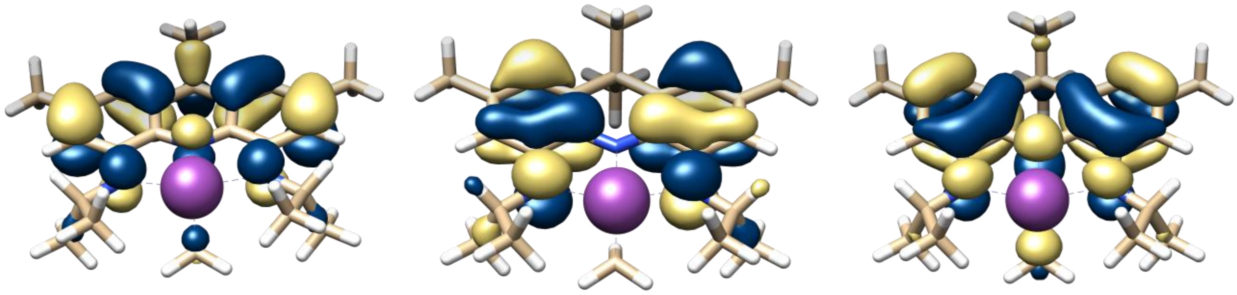

Figure S113. Frontier molecular orbitals of the methylated bismuth complex **3⁺** with HOMO–1 (left), HOMO (center) and LUMO (right) at iso-value 0.03 obtained with ORCA 5.0.4 at TDDFT/ZORA-CAM-B3LYP(D4)/Def2-TZVPP/CPCM(THF) level of theory. Color code: H in white, C in beige, N in blue, Bi in purple.

Table S6. Selected orbital energies of **3⁺**.

Orbital	Energy / Hartree	Energy / eV
**HOMO–1**	-0.2899	-7.882
**HOMO**	-0.249	-6.776
**LUMO**	-0.111	-3.020

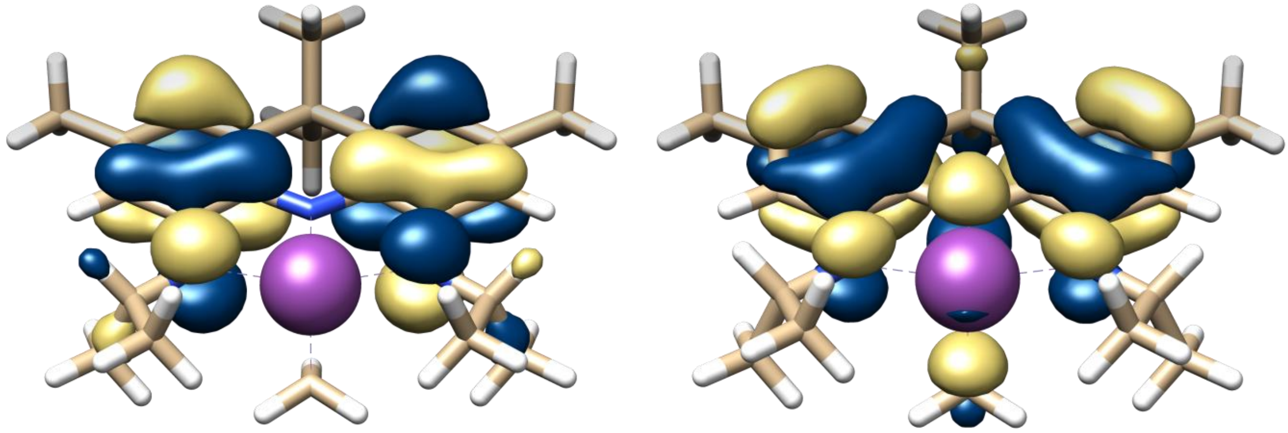

Figure S114. Dominant natural transition orbitals for S1 transition (674 nm) of the methylated bismuth complex **3⁺** with HOMO (left) and LUMO (right) at iso-value 0.03 obtained with ORCA 5.0.4 at TDDFT/ZORA-CAM-B3LYP(D4)/Def2-TZVPP/CPCM(THF) level of theory. Color code: H in white, C in beige, N in blue, Bi in purple.

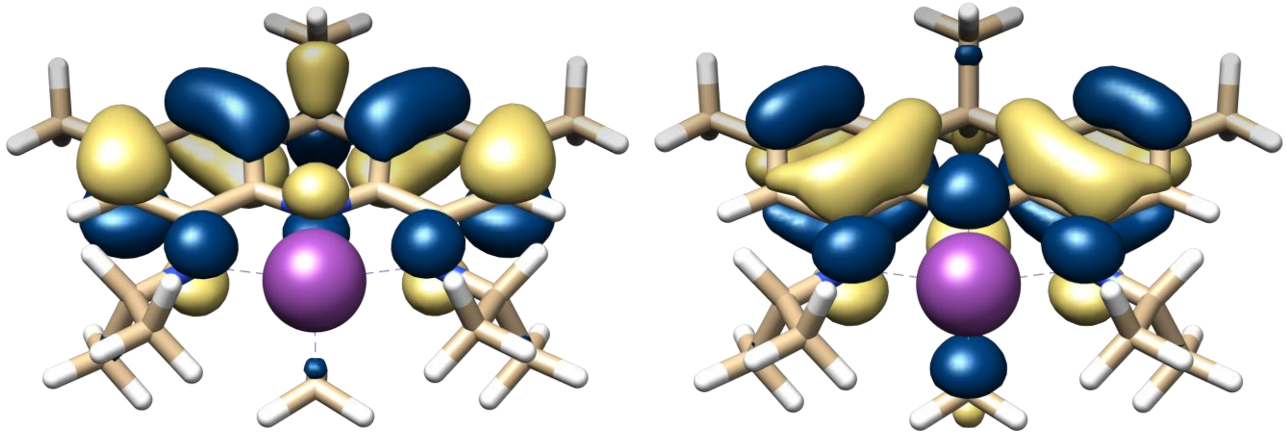

Figure S115. Dominant natural transition orbitals for S2 transition (477 nm) of the methylated bismuth complex cation with HOMO-1 (left) and LUMO (right) at iso-value 0.03 obtained with ORCA 5.0.4 at TDDFT/ZORA-CAM-B3LYP(D4)/Def2-TZVPP/CPCM(THF) level of theory. Color code: H in white, C in beige, N in blue, Bi in purple.

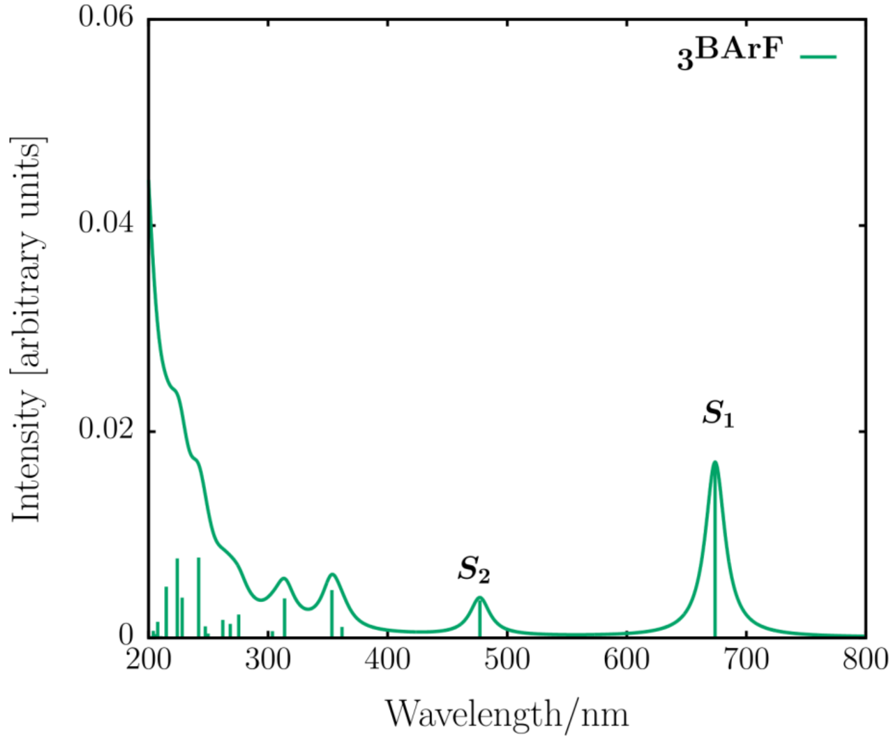

Figure S116. Computational UV/Vis spectrum of the methylated bismuth complex **3**$^+$ obtained with ORCA 5.0.4 at TDDFT/ZORA-CAM-B3LYP(D4)/Def2-TZVPP/CPCM(THF) level of theory.

Transition State of the Methylation of 1^{Bi} by MeOTf

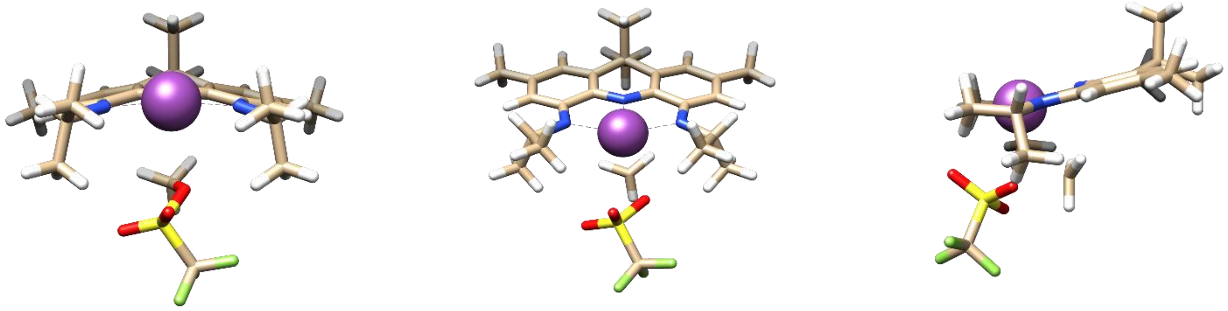

Figure S117. Reactant (left) and transition state structures (middle, right) for methylation of $\mathbf{1^{Bi}}$ complex with triflate counter anion obtained with ORCA 5.0.4 at ZORA-TPSSh(D4)/Def2-TZVPP level of theory. Color code: H in white, C in beige, N in blue, Bi in purple, O in red, S in yellow, F in green.

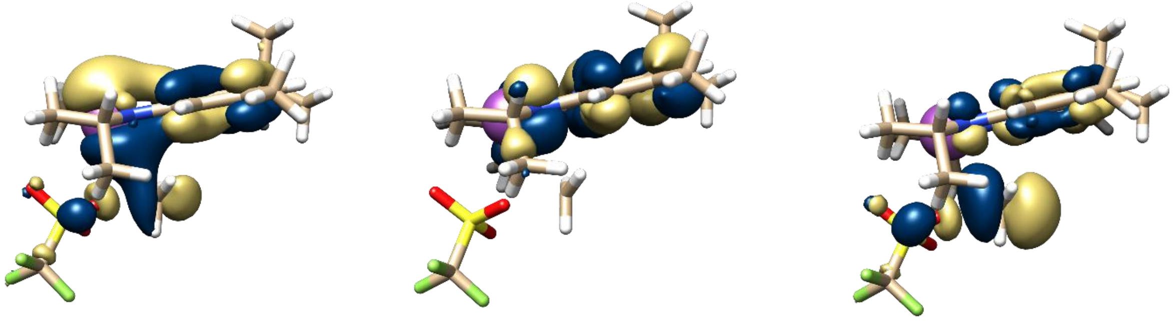

Figure S118. Frontier molecular orbitals of transition state for methylation of $\mathbf{1^{Bi}}$ with triflate counter anion with HOMO–1 (left), HOMO (center) and LUMO (right) at iso-value 0.03 obtained with ORCA 5.0.4 at ZORA-TPSSh(D4)/Def2-TZVPP level of theory. Color code: H in white, C in beige, N in blue, Bi in purple, O in red, S in yellow, F in green.

The transition state for the methylation of the T-shaped bismuth complex $\mathbf{1^{Bi}}$ in gas phase was obtained *via* a *nudge elastic band* (NEB) calculation on ZORA-TPSSh(D4)/Def2-TZVPP level of theory *via* ORCA 5.0.4. A guess structure for the reactant complex was employed as starting point and an optimized structure (same level of theory) of the product complex as end point of the NEB calculation. A subsequent *intrinsic reaction coordinate* calculation followed by an additional optimization on the same level of theory lead to a refinement of the reactant structure. The refined classical activation energy was obtained as $\Delta G^{\ddagger}$ = 32.2 kcal/mol; $\Delta H^{\ddagger}$ = 32.8 kcal/mol. The free reaction enthalpy was determined to be ΔG = –15.8 kcal/mol (ΔH = –13.5 kcal/mol). Solvent effects (DCM, toluene) were found to alter this activation energy only by around 1 kcal/mol.

Frontier molecular orbitals of the TS structure reveal a HOMO–1 with contributions at the $\mathbf{1^{Bi}}$, CH_3 and the triflate fragments, which suggest a related interaction. In contrast, despite the LUMO exhibiting contributions on all fragments, $\mathbf{1^{Bi}}$ seems separated while triflate and CH_3 fragments share orbital contributions. The HOMO is completely located on the NNN pincer ligand of $\mathbf{1^{Bi}}$.

4

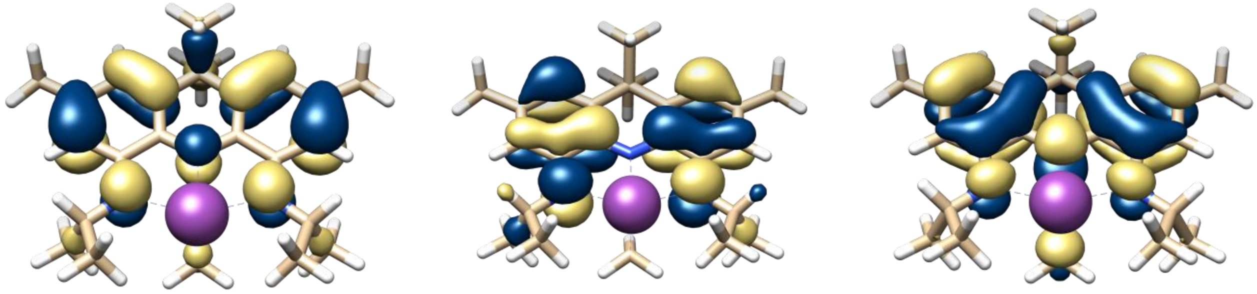

Figure S119. Frontier molecular orbitals of the methylated bismuth radical complex **4** with HOMO–1 (left), HOMO (center) and SUMO (right) at iso-value 0.03 obtained with ORCA 5.0.4 at TDDFT/ZORA-CAM-B3LYP(D4)/Def2-TZVPP/CPCM(THF) level of theory. Color code: H in white, C in beige, N in blue, Bi in purple.

Table S7. Selected orbital (β orbitals) energies of **4**.

Orbital	Energy / Hartree	Energy / eV
**HOMO–1**	-0.248344	-6.757
**HOMO**	-0.211733	-5.768
**SUMO**	-0.071017	-1.933

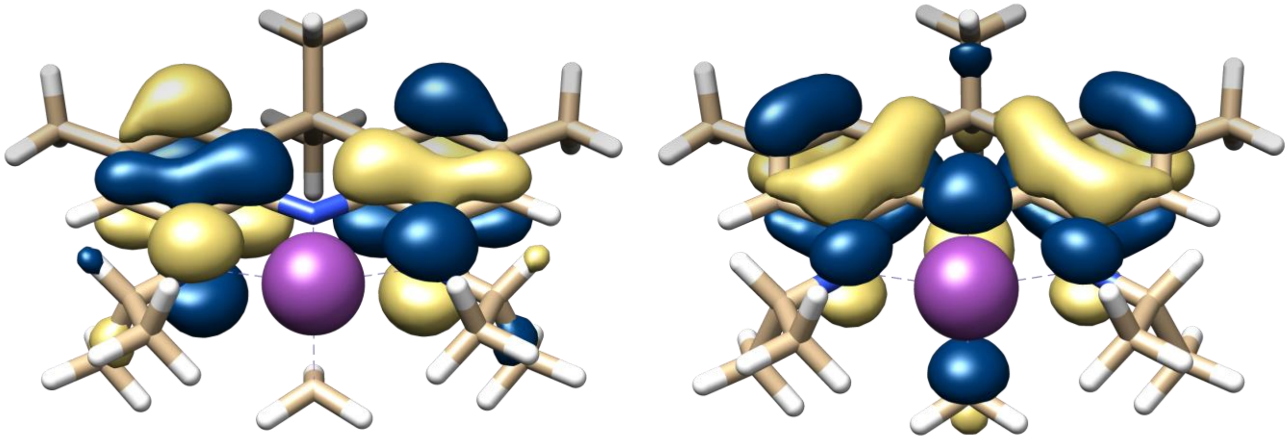

Figure S120. Dominant natural transition orbitals for S2 transition (363 nm) of the methylated bismuth radical complex **4** with HOMO (left) and SUMO (right) at iso-value 0.03 obtained with ORCA 5.0.4 at TDDFT/ZORA-CAM-B3LYP(D4)/Def2-TZVPP/CPCM(THF) level of theory. Color code: H in white, C in beige, N in blue, Bi in purple.

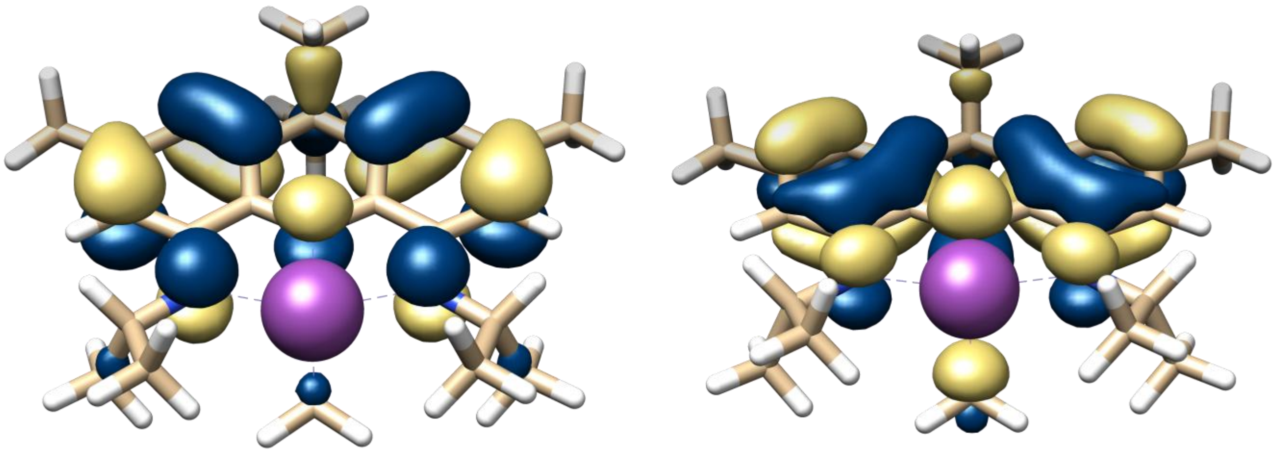

Figure S121. Dominant natural transition orbitals for S3 transition (336 nm) of the methylated bismuth radical complex **4** with HOMO–1 (left) and SUMO (right) at iso-value 0.03 obtained with ORCA 5.0.4 at TDDFT/ZORA-CAM-B3LYP(D4)/Def2-TZVPP/CPCM(THF) level of theory. Color code: H in white, C in beige, N in blue, Bi in purple

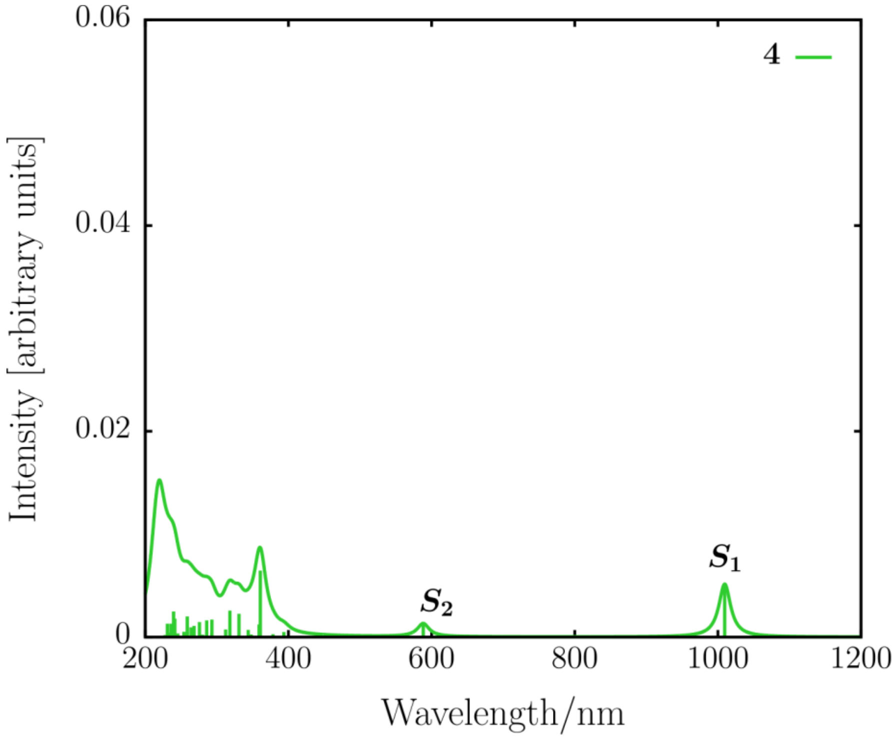

Figure S122. Computational UV/Vis spectrum of the methylated bismuth radical complex **4** obtained with ORCA 5.0.4 at TDDFT/ZORA-CAM-B3LYP(D4)/Def2-TZVPP/CPCM(THF) level of theory.

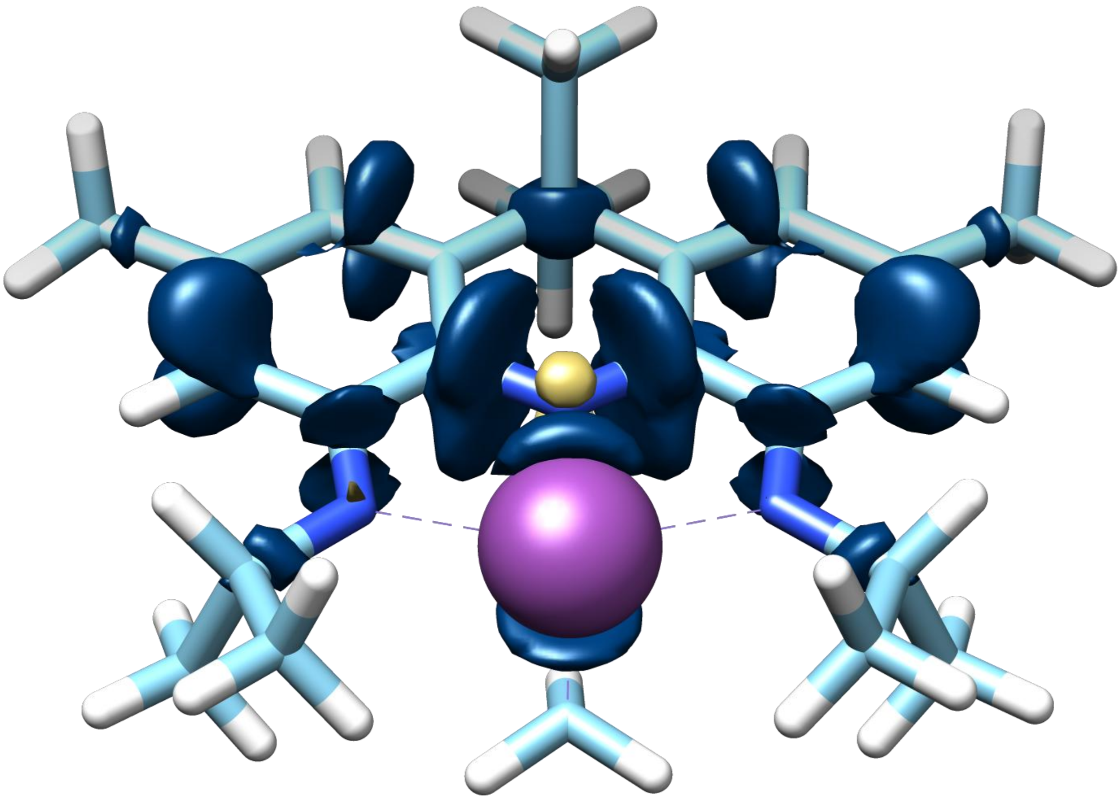

Figure S123. Spin density of **4** (yellow: alpha, blue: beta).

5

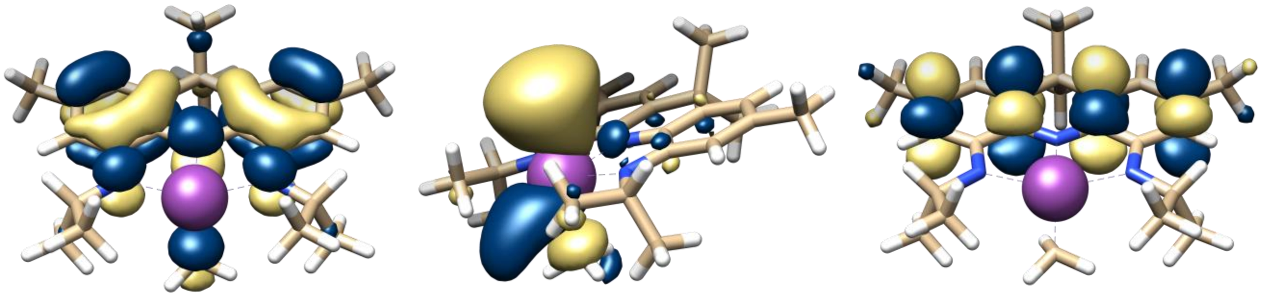

Figure S124. Frontier molecular orbitals of the methylated bismuth complex anion **5** with HOMO (left), LUMO+1 (center) and LUMO+3 (right) at iso-value 0.03 obtained with ORCA 5.0.4 at TDDFT/ZORA-CAM-B3LYP(D4)/Def2-TZVPP/CPCM(THF) level of theory. Color code: H in white, C in beige, N in blue, Bi in purple.

Table S8. Selected orbital energies of **5**.

Orbital	Energy / Hartree	Energy / eV
**HOMO**	-0.161889	-4.408
**LUMO+1**	0.047453	1.292
**LUMO+3**	0.065862	1.793

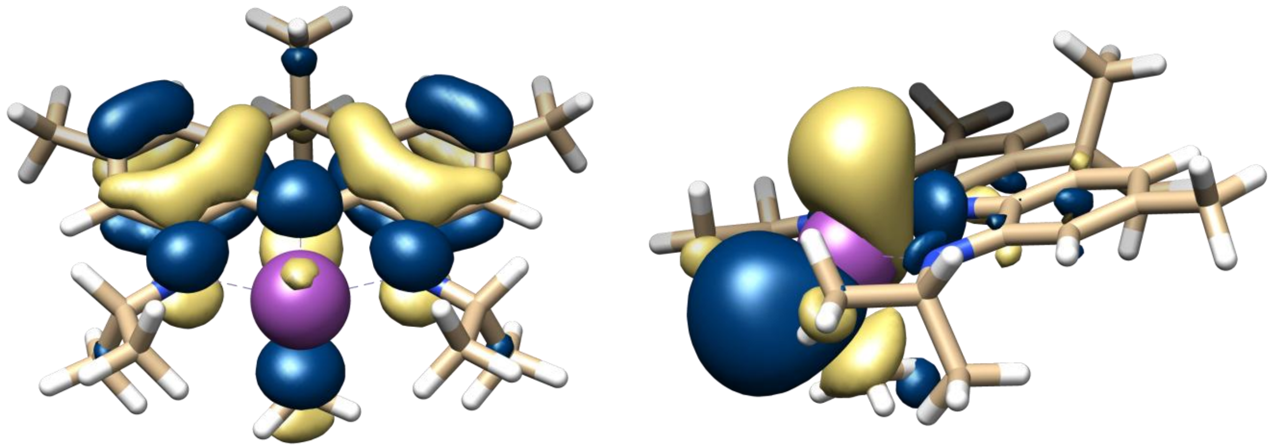

Figure S125. Dominant natural transition orbitals for S2 transition (363 nm) of the methylated bismuth complex anion **5** with HOMO (left) and LUMO+1 (right) at iso-value 0.03 obtained with ORCA 5.0.4 at TDDFT/ZORA-CAM-B3LYP(D4)/Def2-TZVPP/CPCM(THF) level of theory. Color code: H in white, C in beige, N in blue, Bi in purple.

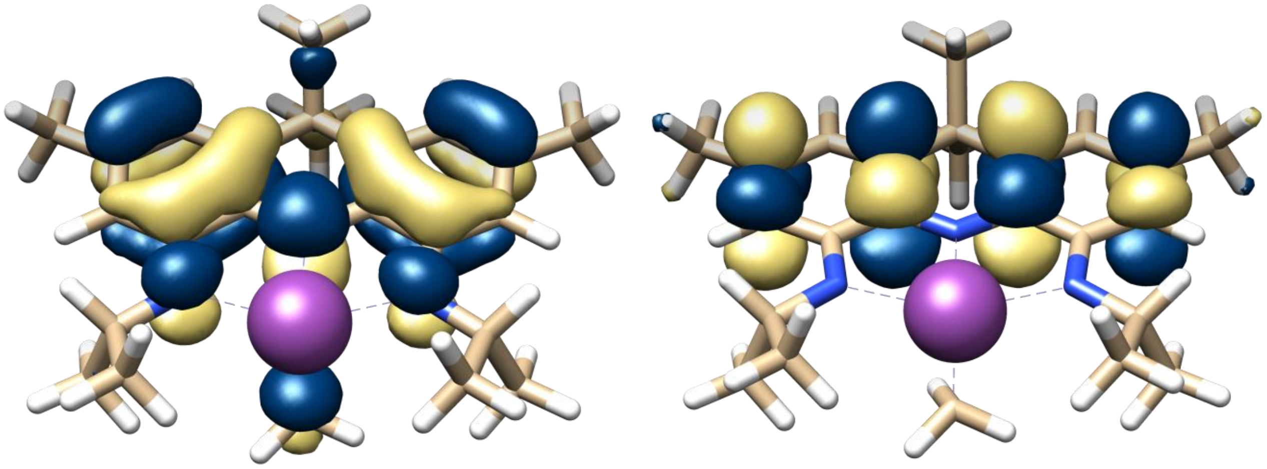

Figure S126. Dominant natural transition orbitals for S3 transition (336 nm) of the methylated bismuth complex anion **5** with HOMO–1 (left) and LUMO (right) at iso-value 0.03 obtained with ORCA 5.0.4 at TDDFT/ZORA-CAM-B3LYP(D4)/Def2-TZVPP/CPCM(THF) level of theory. Color code: H in white, C in beige, N in blue, Bi in purple.

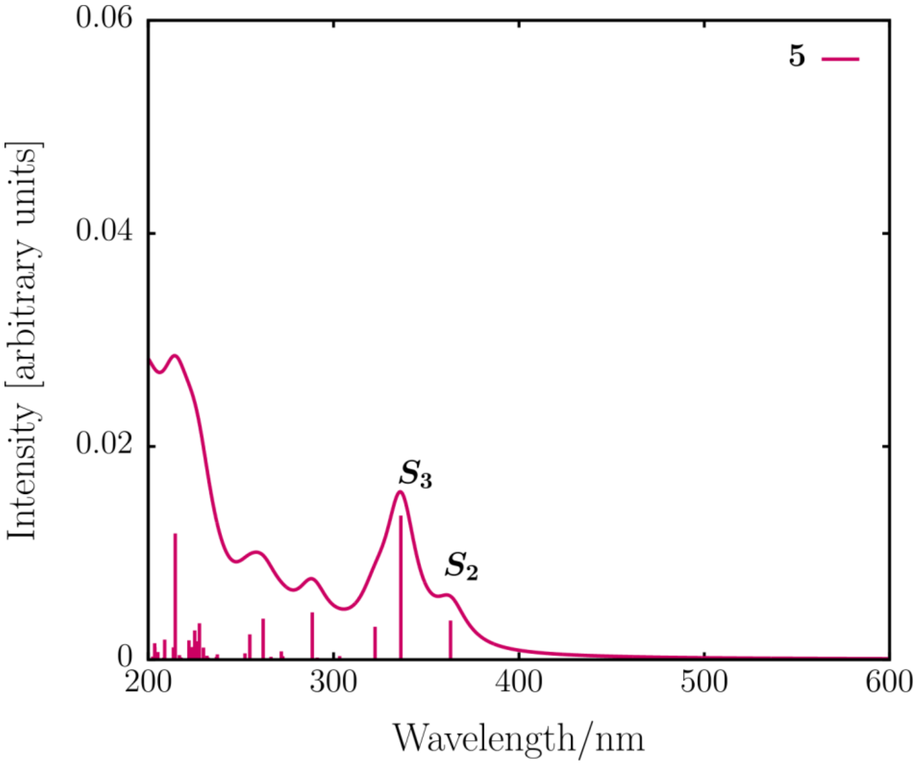

Figure S127. Computational UV/Vis spectrum of the methylated bismuth complex anion **5** obtained with ORCA 5.0.4 at TDDFT/ZORA-CAM-B3LYP(D4)/Def2-TZVPP/CPCM(THF) level of theory.

Spin Properties / Charges of 1 and 3⁺ – 5

Table S9. Loewdin atomic charges and spin populations (ZORA-CAM-B3LYP(D4)/Def2-TZVPP/CPCM(THF)

Atom	**1** / Charge	**3⁺** / Charge	**4**: Charge/Spin Pop.		**5** / Charge
Bi	-0.16	-0.11	-0.21	-0.31	-0.31
N_side	0.18	0.23	0.19	0.15	0.15
N_side	0.18	0.23	0.19	0.15	0.15
central N	0.23	0.33	0.28	0.2	0.2
C at BiMe	-	-0.01	-0.04	-0.08	-0.08

Comparison of Structural Parameters of the Redox Series of 3⁺ - 5

The utilized NNN pincer ligand displays redox non-innocence as recently demonstrated for Ta(V) and P(III) compounds.[8–10,33] Oxidation of the catecholate form *via* the semiquinone radical state to the two-electron oxidized quinone form results in distinct bond alterations within the pincer ligand. The computed structures are fully in line with ligand centered redox events within the series of **3⁺** – **5**, while the bismuth center remains in the +III oxidation state.

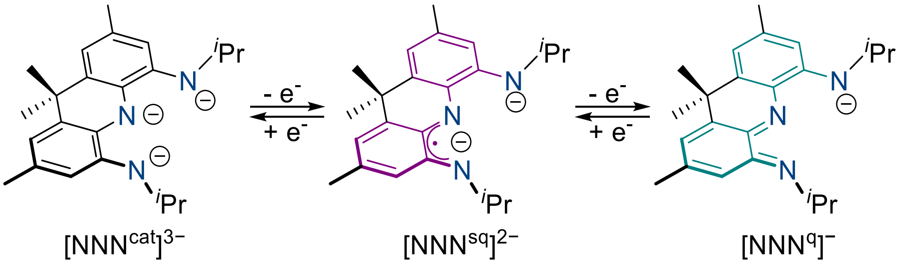

Figure S128. Possible redox states of the NNN pincer ligand.

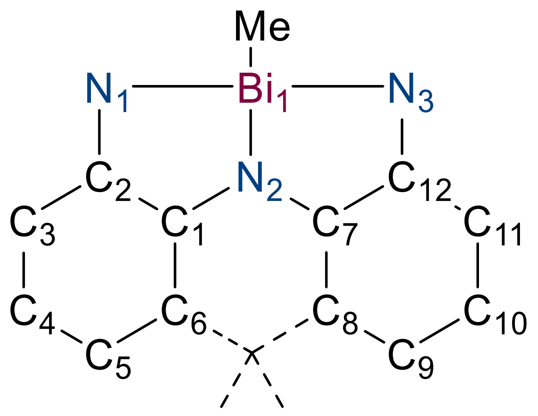

Table S10. Selected calculated bond lengths of **3**$^+$ - **5**.

Bond	3$^+$	4	5
**Bi1–Me**	2.257	2.274	2.311
**Bi1–N1**	2.339	2.334	2.332
**Bi1–N2**	2.270	2.222	2.174
**Bi1–N3**	2.338	2.334	2.333
**N1–C2**	1.326	1.345	1.364
**N2–C1**	1.341	1.362	1.380
**N2–C7**	1.341	1.362	1.380
**N3–C12**	1.326	1.345	1.364
**C1–C2**	1.451	1.438	1.434
**C2–C3**	1.423	1.413	1.401
**C3–C4**	1.370	1.383	1.400
**C4–C5**	1.426	1.404	1.384
**C5–C6**	1.368	1.385	1.406
**C6–C1**	1.418	1.404	1.390
**C7–C8**	1.418	1.404	1.390
**C8–C9**	1.368	1.385	1.406
**C8–C13**	1.426	1.404	1.384
**C9–C10**	1.370	1.383	1.400
**C11–C12**	1.423	1.413	1.402
**C12–C7**	1.451	1.437	1.434

Electrochemistry

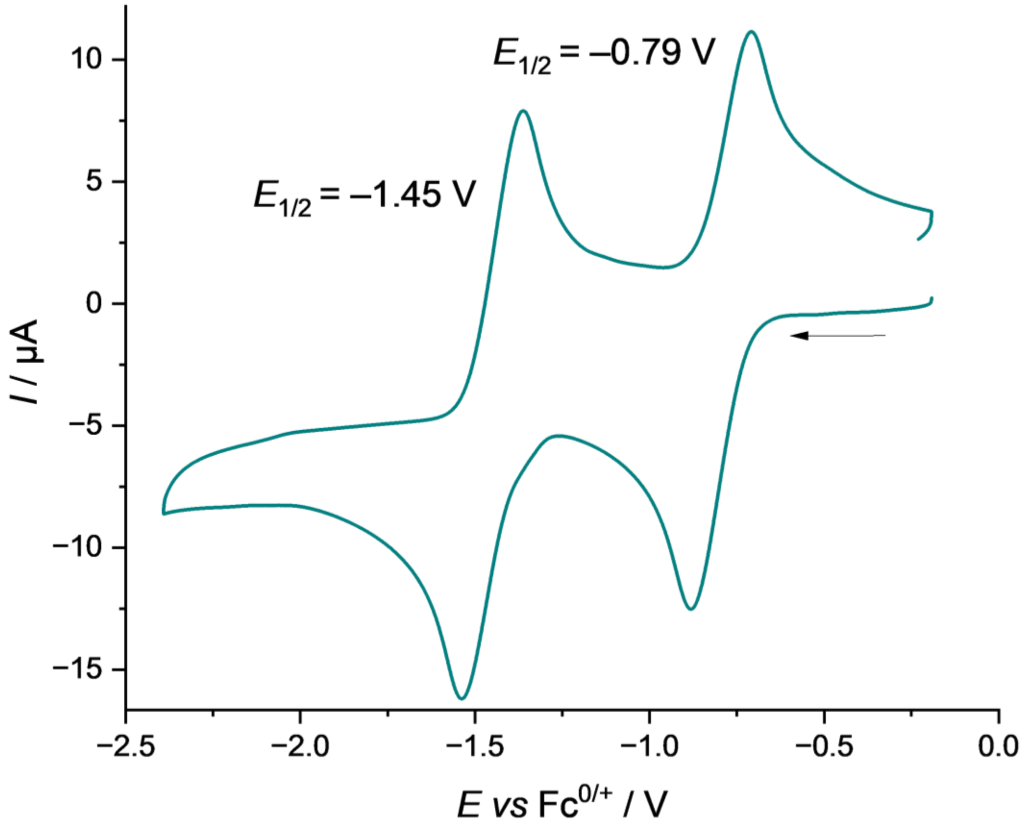

Figure S129. Cyclic voltammogram of $\mathbf{3}^{\mathbf{BArF}}$ in THF, 1 mM, 0.1 M $[NBu_4][PF_6]$, 25 °C, 100 mV/s.

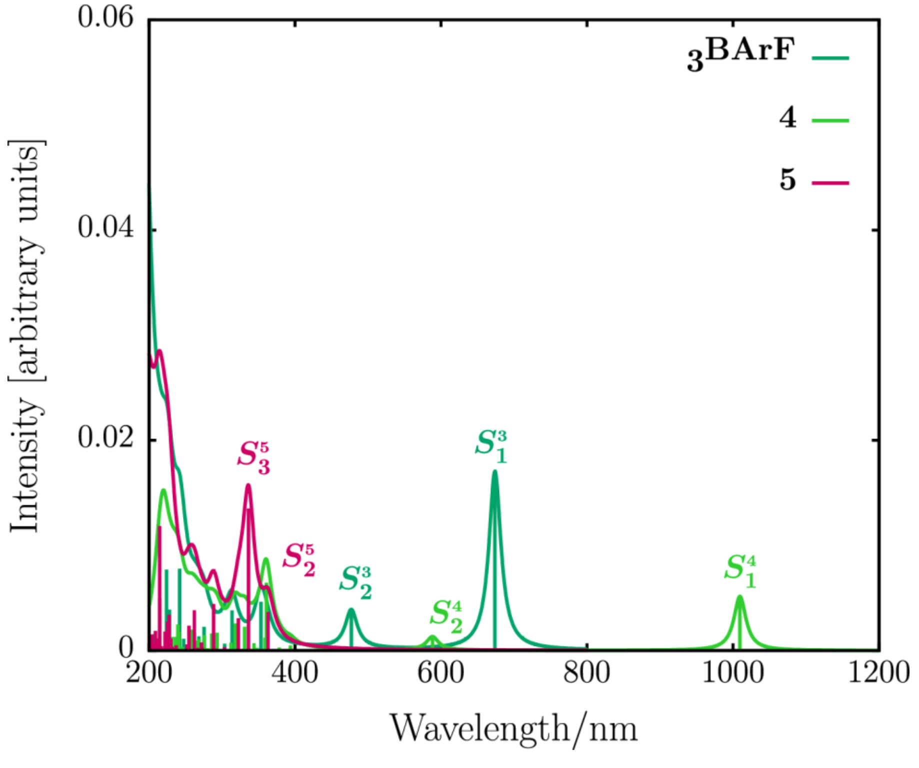

Figure S130. Stacked calculated TDDFT spectra of the Bi(NNN)$Me^{-/0/+}$ redox series.

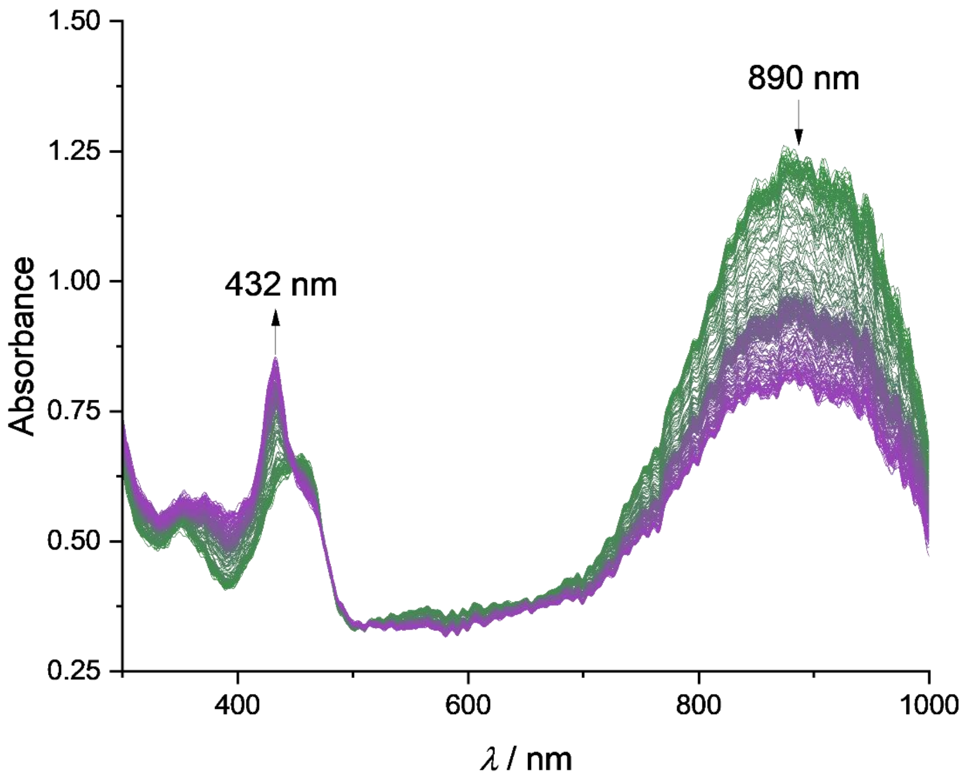

Figure S131. First reduction of 3^{BArF} towards **4** followed by SEC in THF, 1 mM, 0.1 M [NBu4][PF6], 25 °C, 100 mV/s.

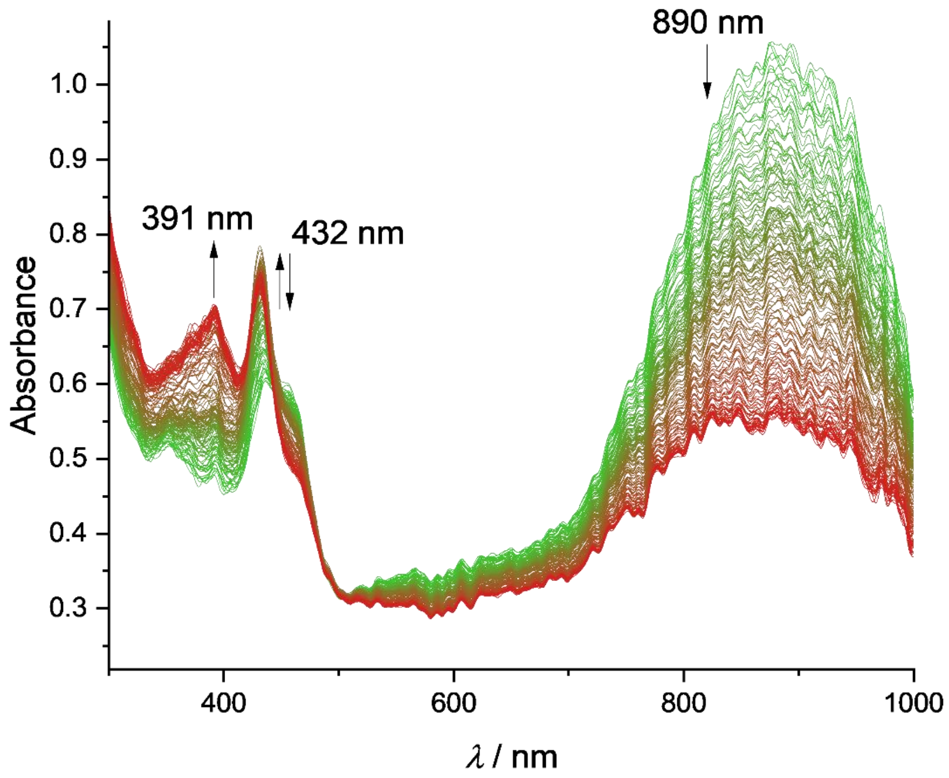

Figure S132. Second reduction of 3^{BArF} towards **5** *via* **4** followed by SEC in THF, 1 mM, 0.1 M [NBu4][PF6], 25 °C, 100 mV/s.

Table S11. Experimental and calculated UV/Vis data of the redox series $\mathbf{3^{BArF}}$ **/ 4 / 5**.

Complex	Exp. / nm	Exp. / cm^{-1}	DFT /nm	DFT /cm^{-1}	Scaling factor
$\mathbf{3^{BArF}}$	890	11236	674	14837	1.32
**4**	431	23202	363	27548	1.19
**5**	391	25575	336	29762	1.16

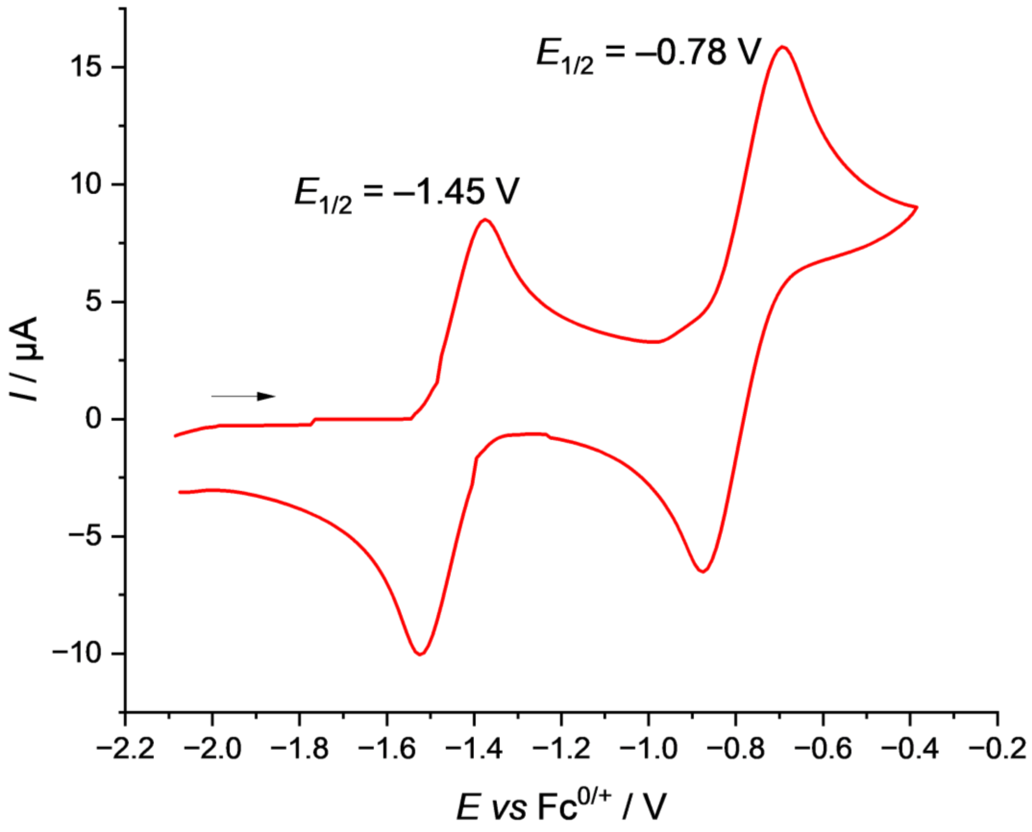

Figure S133. Cyclic voltammogram of **5** in THF, 1 mM, 0.1 M $[NBu_4][PF_6]$, 25 °C, 100 mV/s.

References

(1) Guilherme Buzanich, A.; Radtke, M.; Yusenko, K. V.; M. Stawski, T.; Kulow, A.; Cakir, C. T.; Röder, B.; Naese, C.; Britzke, R.; Sintschuk, M.; Emmerling, F. BAMline—A Real-Life Sample Materials Research Beamline. *J. Chem. Phys.* **2023**, *158* (24), 244202. https://doi.org/10.1063/5.0157194.

(2) Ravel, B.; Newville, M. ATHENA, ARTEMIS, HEPHAESTUS: Data Analysis for X-Ray Absorption Spectroscopy Using IFEFFIT. *J. Synchrotron Radiat.* **2005**, *12* (4), 537–541. https://doi.org/10.1107/S0909049505012719.

(3) Stoll, S.; Schweiger, A. EasySpin, a Comprehensive Software Package for Spectral Simulation and Analysis in EPR. *J. Magn. Reson.* **2006**, *178* (1), 42–55. https://doi.org/10.1016/j.jmr.2005.08.013.

(4) Martínez-Martínez, A. J.; Weller, A. S. Solvent-Free Anhydrous Li+, Na+ and K+ Salts of [B(3,5-(CF3)2C6H3)4]−, [BArF4]−. Improved Synthesis and Solid-State Structures. *Dalton Trans.* **2019**, *48* (11), 3551–3554. https://doi.org/10.1039/C9DT00235A.

(5) Brookhart, M.; Grant, B.; Volpe, A. F. Jr. [(3,5-(CF3)2C6H3)4B]-[H(OEt2)2]+: A Convenient Reagent for Generation and Stabilization of Cationic, Highly Electrophilic Organometallic Complexes. *Organometallics* **1992**, *11* (11), 3920–3922. https://doi.org/10.1021/om00059a071.

(6) Podall, H.; Foster, W. E.; Giraitis, A. P. Catalytic Graphite Inclusion Compounds. I. Potassium Graphite as a Polymerization Catalyst. *J. Org. Chem.* **1958**, *23* (1), 82–85. https://doi.org/10.1021/jo01095a025.

(7) Carmalt, C. J.; Compton, N. A.; Errington, R. J.; Fisher, G. A.; Moenandar, I.; Norman, N. C.; Whitmire, K. H. Homoleptic Bismuth Amides. *Inorg. Synth.* **1996**, 98–101. https://doi.org/10.1002/9780470132623.ch15.

(8) Underhill, J.; Yang, E. S.; Schmidt-Räntsch, T.; Myers, W. K.; Goicoechea, J. M.; Abbenseth, J. Dioxygen Splitting by a Tantalum(V) Complex Ligated by a Rigid, Redox Non-Innocent Pincer Ligand. *Chem. – Eur. J.* **2023**, *29* (5), e202203266. https://doi.org/10.1002/chem.202203266.

(9) Pavlidis, S.; Alasadi, J.; Opis-Basilio, A.; Abbenseth, J. Two-Fold Proton Coupled Electron Transfer of a Ta(V) Aniline Complex Mediated by a Redox Active NNN Pincer Ligand. *Dalton Trans.* **2025**, *54* (6), 2421–2429. https://doi.org/10.1039/D4DT03281K.

(10) King, A. J.; Abbenseth, J.; Goicoechea, J. M. Reactivity of a Strictly T-Shaped Phosphine Ligated by an Acridane Derived NNN Pincer Ligand. *Chem. – Eur. J.* **2023**, *29* (39), e202300818. https://doi.org/10.1002/chem.202300818.

(11) Mayer, U.; Gutmann, V.; Gerger, W. The Acceptor Number — A Quantitative Empirical Parameter for the Electrophilic Properties of Solvents. *Monatshefte Für Chem. Chem. Mon.* **1975**, *106* (6), 1235–1257. https://doi.org/10.1007/BF00913599.

(12) Ramler, J.; Lichtenberg, C. Molecular Bismuth Cations: Assessment of Soft Lewis Acidity. *Chem. – Eur. J.* **2020**, *26* (45), 10250–10258. https://doi.org/10.1002/chem.202001674.

(13) Sheldrick, G. M. SHELXT – Integrated Space-Group and Crystal-Structure Determination. *Acta Crystallogr. Sect. Found. Adv.* **2015**, *71* (1), 3–8. https://doi.org/10.1107/S2053273314026370.

(14) Sheldrick, G. M. Crystal Structure Refinement with SHELXL. *Acta Crystallogr. Sect. C Struct. Chem.* **2015**, *71* (1), 3–8. https://doi.org/10.1107/S2053229614024218.

(15) Groom, C. R.; Bruno, I. J.; Lightfoot, M. P.; Ward, S. C. The Cambridge Structural Database. *Acta Crystallogr. Sect. B Struct. Sci. Cryst. Eng. Mater.* **2016**, *72* (2), 171–179. https://doi.org/10.1107/S2052520616003954.

(16) Pavlidis, S.; Teutloff, C.; Buzanich, A. G.; Krause, K. B.; Emmerling, F.; Bittl, R.; Abbenseth, J. A Crystalline Bismuth(II) Radical Anion: Synthesis, Characterization and Reactivity. *Angew. Chem. Int. Ed.* **2025**, e202515545. https://doi.org/10.1002/anie.202515545.

(17) Coburger, P.; Buzanich, A. G.; Emmerling, F.; Abbenseth, J. Combining Geometric Constraint and Redox Non-Innocence within an Ambiphilic PBiP Pincer Ligand. *Chem. Sci.* **2024**, *15* (16), 6036–6043. https://doi.org/10.1039/D4SC00197D.

(18) Pracht, P.; Bohle, F.; Grimme, S. Automated Exploration of the Low-Energy Chemical Space with Fast Quantum Chemical Methods. *Phys. Chem. Chem. Phys.* **2020**, *22* (14), 7169–7192. https://doi.org/10.1039/C9CP06869D.

(19) Neese, F. The ORCA Program System. *WIRES Comput. Molec. Sci.*, 2012, *2*, 73–78. https://doi.org/10.1002/wcms.81.

(20) Neese, F. Software Update: The ORCA Program System, Version 5.0. *WIRES Comput. Molec. Sci.*, 2022, *12*, e1606. https://doi.org/10.1002/wcms.1606.

(21) Garcia-Rates, M.; Neese, F. Effect of the Solute Cavity on the Solvation Energy and Its Derivatives within the Framework of the Gaussian Charge Scheme. *J. Comput. Chem.*, 2020, *41*, 922–939. https://doi.org/10.1002/jcc.26139.

(22) Tao, J.; Perdew, J. P.; Staroverov, V. N.; Scuseria, G. E. Climbing the Density Functional Ladder: Nonempirical Meta--Generalized Gradient Approximation Designed for Molecules and Solids. *Phys. Rev. Lett.* **2003**, *91* (14), 146401. https://doi.org/10.1103/PhysRevLett.91.146401.

(23) Staroverov, V. N.; Scuseria, G. E.; Tao, J.; Perdew, J. P. Comparative Assessment of a New Nonempirical Density Functional: Molecules and Hydrogen-Bonded Complexes. *J. Chem. Phys.* **2003**, *119* (23), 12129–12137. https://doi.org/10.1063/1.1626543.

(24) Weigend, F.; Ahlrichs, R. Balanced Basis Sets of Split Valence, Triple Zeta Valence and Quadruple Zeta Valence Quality for H to Rn: Design and Assessment of Accuracy. *Phys. Chem. Chem. Phys.* **2005**, *7* (18), 3297–3305. https://doi.org/10.1039/B508541A.

(25) Pantazis, D. A.; Neese, F. All-Electron Scalar Relativistic Basis Sets for the 6p Elements. *Theor. Chem. Acc.* **2012**, *131* (11), 1292. https://doi.org/10.1007/s00214-012-1292-x.

(26) Caldeweyher, E.; Bannwarth, C.; Grimme, S. Extension of the D3 Dispersion Coefficient Model. *J. Chem. Phys.* **2017**, *147* (3), 034112. https://doi.org/10.1063/1.4993215.

(27) Caldeweyher, E.; Ehlert, S.; Hansen, A.; Neugebauer, H.; Spicher, S.; Bannwarth, C.; Grimme, S. A Generally Applicable Atomic-Charge Dependent London Dispersion Correction Scheme. ChemRxiv January 25, 2019. https://doi.org/10.26434/chemrxiv.7430216.v2.

(28) van Lenthe, E.; Snijders, J. G.; Baerends, E. J. The Zero-order Regular Approximation for Relativistic Effects: The Effect of Spin–Orbit Coupling in Closed Shell Molecules. *J. Chem. Phys.* **1996**, *105* (15), 6505–6516. https://doi.org/10.1063/1.472460.

(29) Yanai, T.; Tew, D. P.; Handy, N. C. A New Hybrid Exchange–Correlation Functional Using the Coulomb-Attenuating Method (CAM-B3LYP). *Chem. Phys. Lett.* **2004**, *393* (1), 51–57. https://doi.org/10.1016/j.cplett.2004.06.011.

(30) Riplinger, C.; Sandhoefer, B.; Hansen, A.; Neese, F. Natural Triple Excitations in Local Coupled Cluster Calculations with Pair Natural Orbitals. *J. Chem. Phys.* **2013**, *139* (13), 134101. https://doi.org/10.1063/1.4821834.

(31) Riplinger, C.; Neese, F. An Efficient and near Linear Scaling Pair Natural Orbital Based Local Coupled Cluster Method. *J. Chem. Phys.* **2013**, *138* (3), 034106. https://doi.org/10.1063/1.4773581.

(32) Kindervater, M. B.; Marczenko, K. M.; Werner-Zwanziger, U.; Chitnis, S. S. A Redox-Confused Bismuth(I/III) Triamide with a T-Shaped Planar Ground State. *Angew. Chem. Int. Ed.* **2019**, *58* (23), 7850–7855. https://doi.org/10.1002/anie.201903354.

(33) Pavlidis, S.; Abbenseth, J. Flash Communication: Ligand Centered Cooperative O–H Bond Splitting by a Mo(CO)5(Phosphine) Complex. *Organometallics* **2025**, *44* (3), 483–486. https://doi.org/10.1021/acs.organomet.4c00507.
